# Selective Bacterial Targeting and Infection‐Triggered Release of Antibiotic Colistin Conjugates

**DOI:** 10.1002/anie.202104921

**Published:** 2021-07-05

**Authors:** Werner Tegge, Giulia Guerra, Alexander Höltke, Lauritz Schiller, Ulrike Beutling, Kirsten Harmrolfs, Lothar Gröbe, Hannah Wullenkord, Chunfa Xu, Herbert Weich, Mark Brönstrup

**Affiliations:** ^1^ Department of Chemical Biology Helmholtz Centre for Infection Research Inhoffenstrasse 7 38124 Braunschweig Germany; ^2^ Flow Cytometry and Cell Sorting Platform Helmholtz Centre for Infection Research Inhoffenstrasse 7 38124 Braunschweig Germany; ^3^ German Center for Infection Research (DZIF), Site Hannover-Braunschweig Germany; ^4^ Center of Biomolecular Drug Research (BMWZ) Leibniz Universität 30167 Hannover Germany

**Keywords:** Antibiotics, drug conjugates, drug delivery, immune activation, natural products

## Abstract

In order to render potent, but toxic antibiotics more selective, we have explored a novel conjugation strategy that includes drug accumulation followed by infection‐triggered release of the drug. Bacterial targeting was achieved using a modified fragment of the human antimicrobial peptide ubiquicidin, as demonstrated by fluorophore‐tagged variants. To limit the release of the effector colistin only to infection‐related situations, we introduced a linker that was cleaved by neutrophil elastase (NE), an enzyme secreted by neutrophil granulocytes at infection sites. The linker carried an optimized sequence of amino acids that was required to assure sufficient cleavage efficiency. The antibacterial activity of five regioisomeric conjugates prepared by total synthesis was masked, but was released upon exposure to recombinant NE when the linker was attached to amino acids at the 1‐ or the 3‐position of colistin. A proof‐of‐concept was achieved in co‐cultures of primary human neutrophils and Escherichia coli that induced the secretion of NE, the release of free colistin, and an antibacterial efficacy that was equal to that of free colistin.

## Graphical abstract

A multifunctional peptide–colistin conjugate for the treatment of bacterial infections has been synthesized. The conjugate binds to *E. coli* bacteria by an all‐d sequence of the human peptide ubiquicidin_29–41_. Host immune cells, activated upon infection, secrete the enzyme neutrophil elastase that cleaves an optimized linker sequence to release free colistin, a potent antibiotic that kills the bacteria.

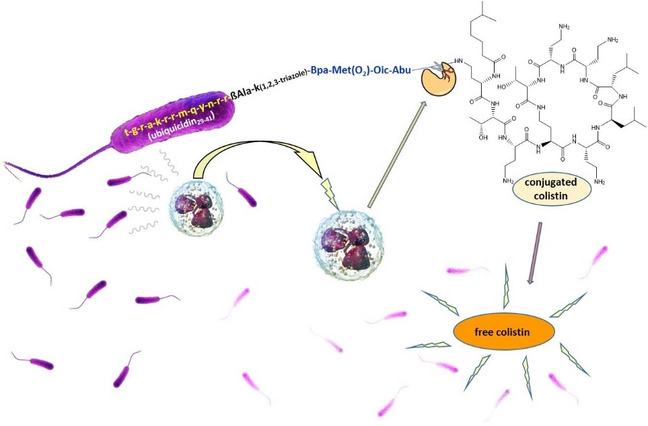

## Introduction

**Figure 1:**
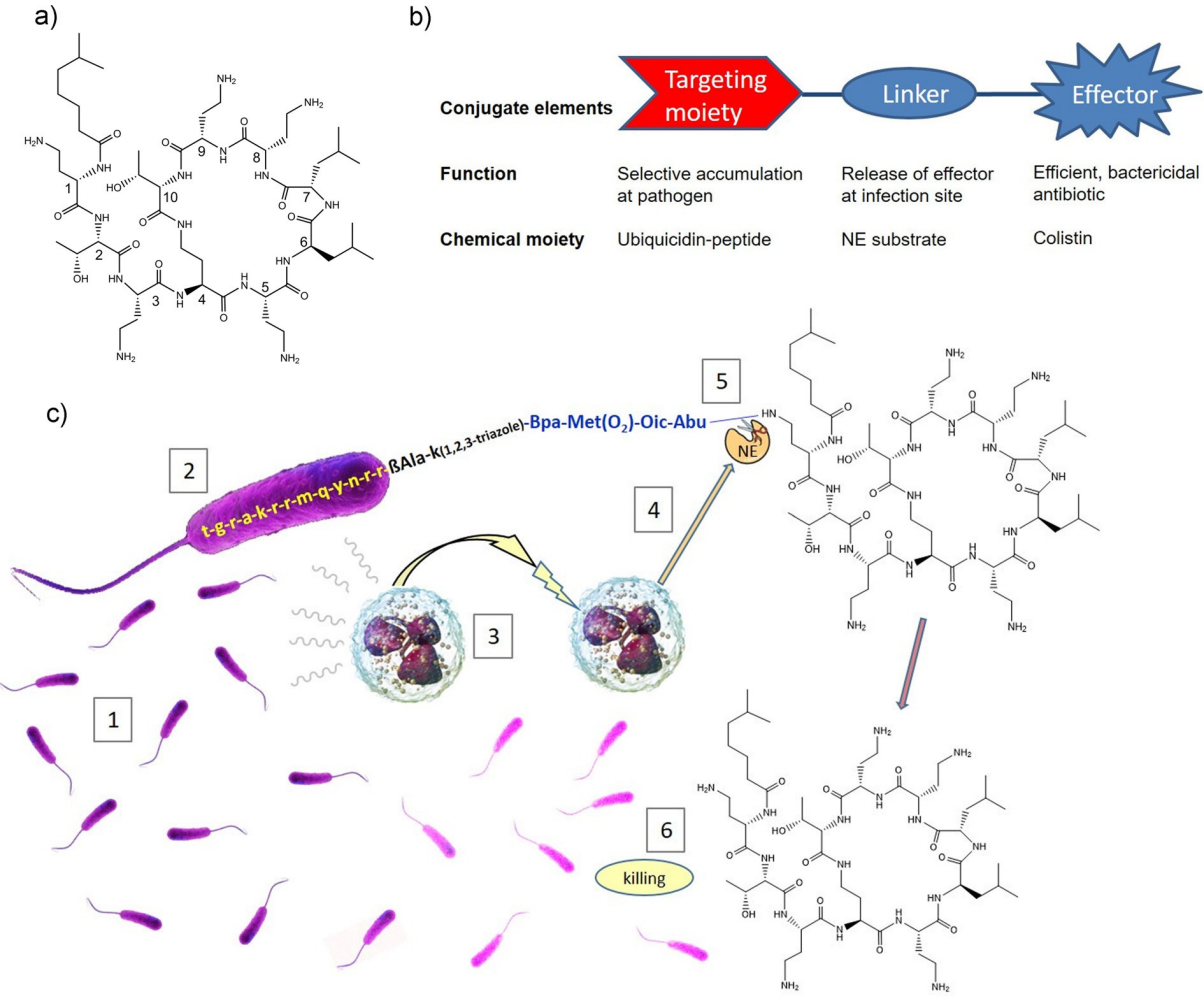
Overview of selective targeting and infection‐triggered release of antibiotics: a) Structure of colistin B (**1**). Numbers indicate the position of amino acids. b) Schematic overview of conjugate design. c) Cellular mechanism of action. 1: Bloodstream infection with Gram‐negative bacteria (in purple); 2: Binding of the peptide–colistin conjugate to a bacterium (enlarged view) via the Ubi_29–41_ sequence (in yellow); 3: Attraction of neutrophil granulocytes to the site of infection; 4: Secretion of neutrophil elastase (NE; in orange) from activated neutrophil granulocytes; 5: Cleavage of the linker (in blue) and release of free **1**; 6: Killing of bacteria. Bpa=4‐benzoyl phenylalanine, Met(O_2_)=l‐methionine sulfone, Oic=octahydro‐1*H* indole‐2‐carboxylic acid, Abu=2‐aminobutyrate. Small letters indicate d‐amino acids.

Infections by multidrug‐resistant bacteria have been recognized as a global threat to human health.^[1]^ In particular, the WHO has emphasized that the arsenal of antibiotics against resistant Gram‐negative pathogens has become alarmingly thin.^[2]^ This led to the decision to reactivate the natural lipopeptide colistin **1** (Figure 1 a, the shown colistin B, also known as polymyxin E2, was used throughout in this study)[^3^, ^4^] as a “last resort” treatment of infections, after its systemic use has been banned for decades due to its toxicity. The strong nephrotoxicity of colistin, leading to acute kidney injury (AKI) in over 30 % of cases,^[5]^ as well as adverse neurological effects make use of this compound a delicate balance between benefit and harm for the patients.^[6]^ However, the potent, broad spectrum activity and the (yet) low level of resistance^[7]^ make colistin an attractive antibiotic scaffold. In consequence, the synthesis of colistin derivatives with improved drug properties or of colistin‐related scaffolds^[8]^ has recently become a topic of intense research activities, with most efforts focused on the empirical replacement of the basic amino acids by other chemical moieties.^[9]^ Binary conjugates of colistin with dextrin,^[10]^ alginate‐oligosaccharide^[11]^ and poly(ethylene glycol) (PEG) derivatives^[12]^ have also been reported, that lead to a liberation of colistin by the action of α‐amylase,^[10]^ esterase or alginate lyase activity,^[11]^ or hydrolysis.^[12]^ A targeting for these macromolecules to inflammation sites can be achieved due to an enhanced leakiness of vessels in inflamed tissue.^[13]^

Herein, we would like to present a novel concept to improve the therapeutic window of highly potent, but also toxic antibiotics like colistin by selectively enhancing their concentration at the pathogen, while minimizing the (toxic) exposure to host cells. For this purpose, a drug conjugation approach^[14]^ was applied to introduce two additional functionalities to **1**:

A targeting moiety should direct the antibiotic effector specifically to bacterial cells
The release of the effector should be triggered selectively at the site of infection

We decided to reduce the concept to practice with components accessible by peptide chemistry (Figure 1 b): Due to the advantages outlined above, colistin was the effector antibiotic of choice. For bacterial targeting, the 13mer fragment Ubi_29–41_ of the human antimicrobial peptide ubiquicidin^[15]^ was selected, because its ability to bind to anionic phospholipid head groups of Gram‐negative bacteria with high affinity and specificity, while showing little binding to mammalian cells, has been validated with a large body of animal and even human imaging data.^[16]^ To protect the targeting peptide from proteolytic degradation under in vivo conditions, the original Ubi_29–41_ was replaced with its *all*‐d‐enantiomer.^[17]^ A corresponding conjugate with a Ubi_29–41_ peptide made of l‐amino acids was also synthesized for comparison. In order to limit the release of the effector only to infection‐related situations, we wanted to take advantage of the fact that neutrophil granulocytes are rapidly recruited at infection sites,^[18]^ concomitant with a local release of neutrophil elastase (NE) by the immune cells to destroy bacteria (Figure 1 c). Thus, a proteolytic cleavage site recognized by NE was incorporated in the linker connecting Ubi_29–41_ and colistin. In order to assure a high cleavage fidelity, an optimized substrate peptide composed of the four non‐proteinogenic amino acids Bpa‐Met(O_2_)‐Oic‐Abu (Bpa=4‐benzoyl phenylalanine, Met(O_2_)=l‐methionine sulfone, Oic=octahydro‐1*H* indole‐2‐carboxylic acid, Abu=2‐aminobutyrate), that is cleaved more than 7000 times faster than the commonly used Ala‐Ala‐Pro‐Val sequence,^[19]^ was incorporated into the conjugate. In addition, we expected the non‐proteinogenic amino acids to be resistant to proteolytic degradation by other plasma enzymes. The fact that unmodified colistin is released upon proteolysis by NE is seen as another advantage, because the antibiotic properties of colistin (e.g. its high potency) are not impaired by residual linker parts. While prodrug‐protected cationic antimicrobial peptides have been reported before,^[20]^ the concept of trifunctional antibiotic conjugates is, to the best of our knowledge, new—and quite complex, as it requires an interplay of at least four components, that is, the trifunctional conjugate, neutrophil elastase, neutrophil granulocytes and bacteria (Figure 1 c). This paper describes the realization and validation of the concept.

## Results and Discussion

**Figure 2:**
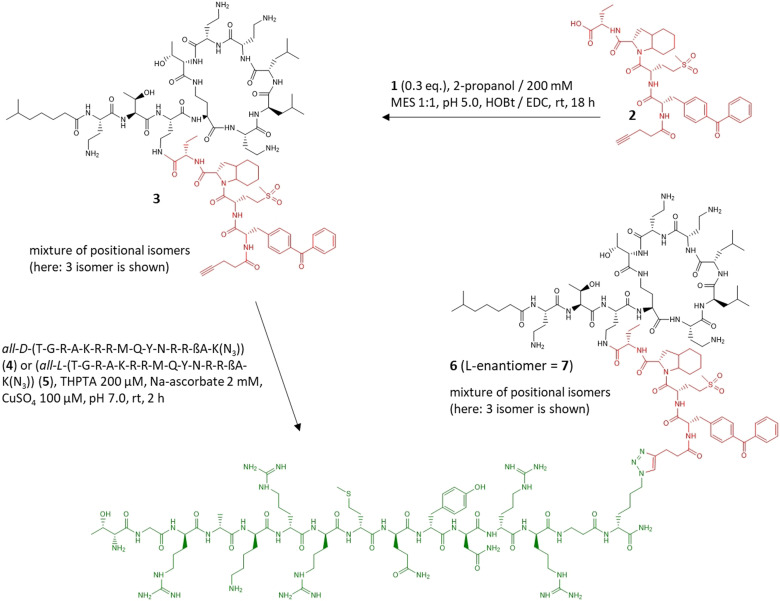
Semisynthesis of **6** and **7**. In green: bacterial binder d‐ubiquicidin_29–41_‐ßAla‐lys(N_3_); in red: NE‐cleavable linker; in black: colistin B. In **5** and **7**, the ubiquicidin_29–41_ part is made entirely of l‐amino acids.

The synthesis of the conjugates was achieved by two different strategies. To satisfy the material demand for the biological characterization experiments, a semisynthetic approach was chosen. Because all attempts to connect Ubi_29–41_ as a C‐terminal thioester to a cysteine‐modified elastase substrate by native chemical ligation failed (data not shown), a copper‐catalyzed alkyne–azide cycloaddition reaction was applied for coupling. The Eastern part of the molecule was assembled by N‐terminally extending the elastase substrate Bpa‐Met(O_2_)‐Oic‐Abu, obtained by peptide synthesis, with 4‐pentinoic acid to give **2**. The alkyne‐modified substrate **2** was subsequently reacted with the amino group(s) of **1** that was isolated from a commercial fermentation product (Figure 2). The monosubstituted isomers **3** were separated from **1** and the higher substitution variants, but no attempt was made to further resolve the positional isomers that eluted with similar retention times from the HPLC column (see the Supporting Information). In parallel, a short spacer consisting of β‐alanine and a C‐terminal azido‐lysine was added to the C‐terminus of the *all‐* d‐enantiomer of Ubi_29–41_ **4**, or to its *all*‐l‐enantiomer **5**, respectively, to build the Western part of the conjugate. Finally, the fully functionalized conjugates **6** and **7** were prepared by coupling **3** to **4** and **5** via a copper‐catalyzed click reaction, respectively, and characterized by HPLC (purities >95 %) and high resolution mass spectrometry.

**Figure 3:**
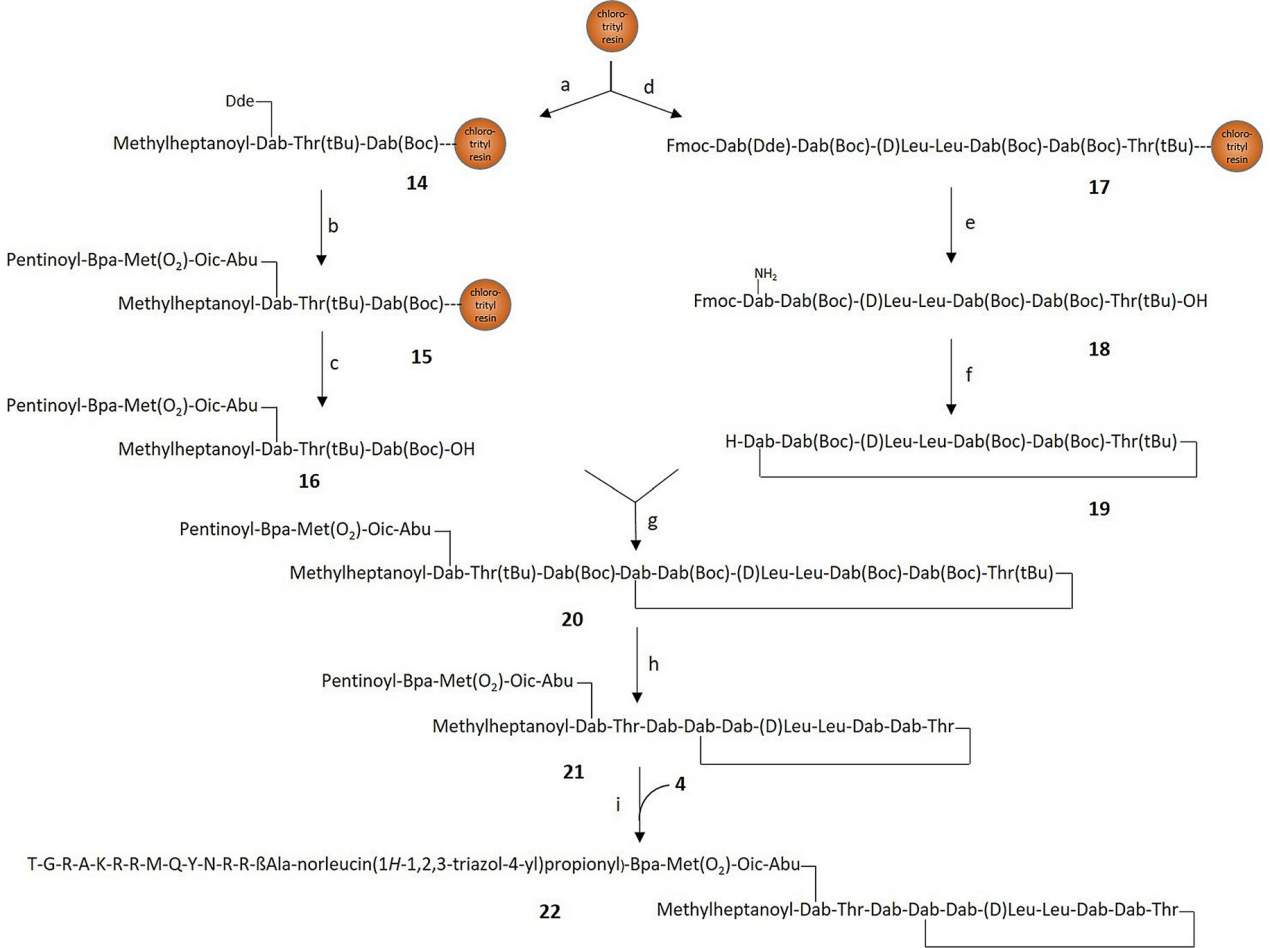
Total synthesis of **22**, modified at position 1 of colistin. a) i) Fmoc‐Dab(Boc)‐OH, DIPEA, DCM, 2 h; ii) piperidine (20 %) in DMF, 10 min; iii) appropriate amino acid and 6‐methyl heptanoic aicd, each with activation by HCTU and DIPEA, DMF, 1 h and followed by piperidine (20 %), DMF, 10 min; b) i) hydrazine (20 mm) in DMF, 3×90 min, ii) Fmoc‐Abu‐OH, Fmoc‐Oic‐OH, and Fmoc‐Met(O_2_)‐OH, each with activation by HCTU and DIPEA, DMF, 1 h, and followed by piperidine (20 %) in DMF, 10 min; iii) Fmoc‐Bpa‐OH, HATU, DIPEA, DMF, 1 h as double coupling, followed by piperidine (20 %), DMF, 10 min, iv) 4‐pentynoic acid, HCTU, DIPEA, DMF, 1 h; c) 1,1,1,3,3,3‐hexafluoro‐2‐propanol (HFIP)/DCM 1:4, 1 h, followed by RP‐HPLC purification; d) i) Fmoc‐Thr(tBu)‐OH, DIPEA, DCM, 2 h; ii) piperidine (20 %) in DMF, 10 min; iii) appropriate amino acid, each with activation by HCTU and DIPEA, DMF, 1 h, and followed by piperidine (20 %) in DMF, 10 min; e) i) hydroxylamine⋅HCl (257 mm), imidazole (193 mm), DCM/NMP 5:1, ii) HFIP/DCM 1:4, 1 h; f) i) DIC, HOBt, DCM/DMF 49:1, 5 h; ii) piperidine (1 %) in DMF, 1 h; g) **19** (1 equiv), PyBOP, HOBt, DIPEA, DMF, 5 h; h) TFA (82.5 %), H_2_O (5 %), phenol (5 %), thioanisol (5 %), dithiothreitol (2.5 %), 90 min, followed by RP‐HPLC purificatin; i) **4** (1 equiv), THPTA (200 μm) Na‐ascorbate (2 mm), CuSO_4_ (100 μm), pH 7.0, 2 h, followed by RP‐HPLC purification.

In the second strategy, also the colistin part was obtained by organic synthesis in order to fully control the position of the linker attachment to any of the five primary amino groups. We devised two different synthetic schemes to derivatize either the two extracyclic diaminobutyric acid (Dab) residues at positions 1 and 3, or the three intracyclic ones at positions 5, 8, and 9 (for atom numbering, see Figure 1 a). The compounds were prepared using solid‐phase peptide synthesis (SPPS) on 2‐chlorotrityl chloride (CTC) resins and a fluorenylmethoxycarbonyl (Fmoc) protocol. The side chain amino groups of Dab were protected as *tert*‐butyloxycarbonyl (Boc), the side chain hydroxy group of Thr as *tert*‐butyl (tBu), while the γ‐amino groups of the Dab involved in cyclization (position 4) or branching carried a 1‐(4,4‐dimethyl‐2,6‐dioxocyclohex‐1‐ylidene)ethyl (Dde) moiety that is orthogonal to Boc and tBu. For the two conjugates modified at extracyclic residues, a convergent approach was selected in which the extracyclic chain was synthesized in parallel to the cyclic core, followed by fragment condensation. The synthesis of **22**, the conjugate modified at position 1, started with the loading of γ‐Boc‐protected Dab onto chlorotrityl resin (Figure 3). After assembly of the extracyclic colistin sequence, the γDde group at Dab‐1 was cleaved, the elastase substrate peptide with N‐terminal 4‐pentinoyl group was coupled, and the protected peptide **16** with a free C‐terminus was cleaved from the support by mild acidic treatment with hexafluoro‐2‐propanol (HFIP). For the cyclic portion of colistin, a linear peptide was obtained by SPPS from Thr (position 10) to the γDde‐protected Dab residue at position 4. After Dde removal and cleavage from the resin, the peptide was cyclized and Fmoc‐deprotected, thereby offering a single amino group at the α‐position of residue 4. The cyclopeptide **19** was then coupled to the free carboxy group of **16**. After a global deprotection, the triple bond of the 4‐pentinoyl moiety was “clicked” to the azido group of the ubiquicidin derivative **4**, affording the final compound **22**. Conjugate **28**, modified at position 3 of **1**, was obtained in a similar manner (Supplementary Figure S1). For the synthesis of three conjugates at intracyclic residues, Fmoc‐Thr(tBu) was attached to 2‐CTC resin, and linear peptide chains with the elastase sequences attached to positions 5, 8, or 9, respectively, were assembled by sequential deprotection/couplings (Supplementary Figures S2, S3, and S4). The branching positions for the elastase substrate peptide were α‐Dde protected, and after completion of the elastase substrate synthesis at the γ‐amino group of the respective Dab, the Dde group was removed, and the remaining colistin sequence was assembled. After cleavage from the resin, the peptides were cyclized in solution and globally deprotected. Finally, the 4‐pentinoyl moiety was coupled by cycloaddition to the ubiquicidin derivative **4**, affording the final compounds **34**, **40**, and **46**. Final conjugates **22**, **28**, **34**, **40**, and **46** were characterized by 1D and 2D NMR spectroscopy, high resolution mass spectrometry, and HPLC. The structure elucidation by NMR spectroscopy was based on a comparative analysis with peptide **50** (Supplementary Figure S18 and S19) and a full assignment of **1** (Supplementary Table S1, Figures S20–S26), which, to the best of our knowledge, has not been reported so far. Even though ^1^H NMR and fragment data are available for a wide range of natural and synthetic derivatives,[^4^, ^21^] only the synthetic octapeptin representative FADDI‐118 was completely assigned.^[8]^ Corroboration of the linker attachment positions was achieved by detailed analysis of specific ^1^H and ^13^C chemical shifts and HMBC correlations to the corresponding Dab moiety (Supplementary Tables S2–S6, Figures S27–S46). Overall, the final products were obtained in amounts of 4.9–6.9 mg using the fully synthetic approach, which enabled the determination of structure–activity relationships in a basic microbiological experiment (see below). However, conjugates required in larger amounts were prepared by semisynthesis.

In order to verify the claim that the Ubi_29–41_‐carrying conjugate indeed accumulates at Gram‐negative bacteria, we prepared an analogue of **6** that was further functionalized with a fluorescent dye for bacterial imaging (Supplementary Figure S5). For this purpose, **4** was C‐terminally elongated with cysteine to **8**, and subsequently reacted with fluorescein maleimide to give **10**. The intermediate **10** was then coupled to **3** to yield the tetrafunctional conjugate **12**. An analogous procedure was conducted with the *all‐* l‐peptide **9**, yielding analogue **13**.

**Figure 4:**
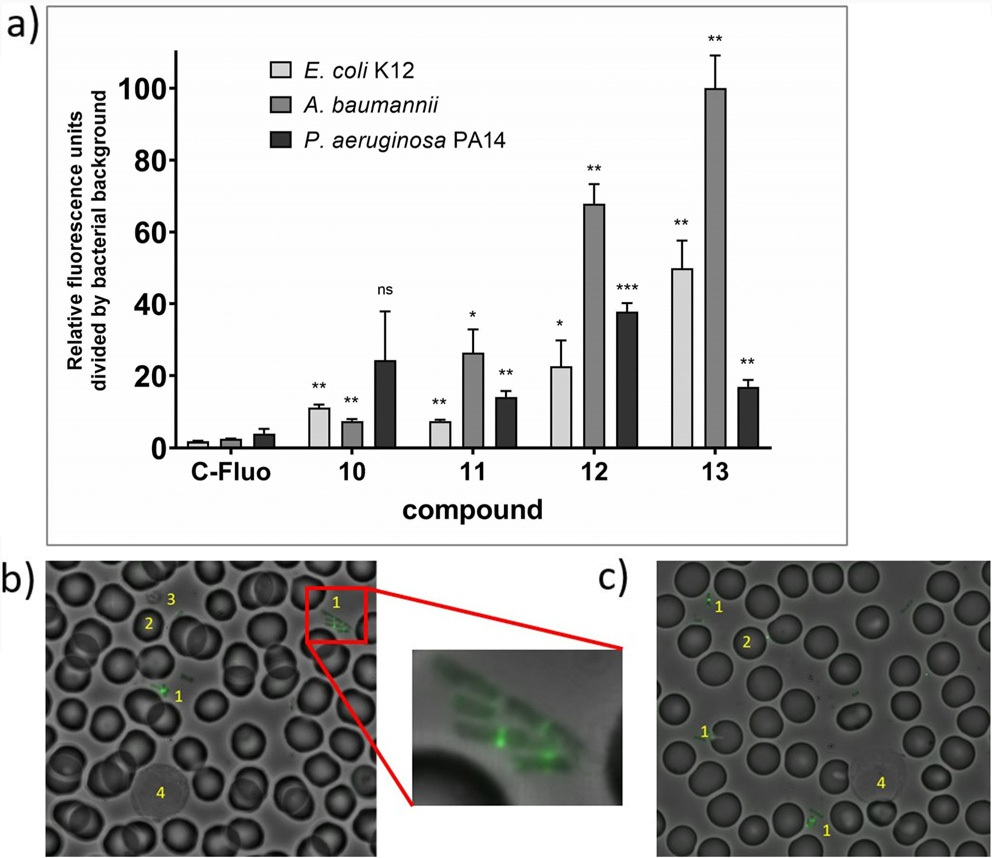
Staining of Gram‐negative bacteria with fluorescently labeled colistin conjugates. a) Three Gram‐negative bacteria (*E. coli* K12, *A. baumannii*, *P. aeruginosa* PA14) were grown in T‐medium, to an OD_600_ of 1.0, and the fluorescently labeled compounds were added (1 μm in PBS, rt, 1 h incubation), followed by washing steps and a quantification of fluorescence in the wells. C‐Fluo: 5(6)‐carboxyfluorescein. Shown is the arithmetric mean of 3 measurements. *p<0.04, **p<0.009, ***p<0.0002, ns: not significant, in comparison to the respective control with 5(6)‐carboxyfluorescein according to Welch's unpaired t‐test; b,c) Microscopy of smears of whole blood after addition of *E. coli* K12 and **12** (b) and **13** (c). An overlay of fluorescence (485/520 nm) and phase contrast pictures is shown. A cluster of bacteria in (b) is magnified in the box. 1=bacteria, 2=erythrocytes, 3=thrombocytes, 4=white blood cells.

The binding properties of **12, 13**, and controls were probed with the three medically relevant Gram‐negative pathogens *Escherichia coli* (*E. coli*), *Pseudomonas aeruginosa* (*P. aeruginosa*), and *Acinetobacter baumannii* (*A. baumannii*) by quantifying the fluorescence of bacteria following a 1 h compound incubation and subsequent washing steps. While 5(6)‐carboxyfluorescein did hardly show binding above background, both **12** and **13** led to fluorescence that was 17–100 fold above background (Figure 4). For *E. coli* and *A. baumannii* the bacterial binding of the *all‐* l‐Ubi_29–41_ conjugate **13** was higher than that of **12**, whereas *P. aeruginosa* preferentially bound the *all*‐d‐Ubi_29–41_ variant **12**. However, the signal strength for both conjugates suggests that chiral recognition plays a limited role for the binding of the peptide to the lipid membrane of bacteria. The fluorescence signals obtained for the *all‐* l‐Ubi_29–41_‐fluorescein and *all‐* d‐Ubi_29–41_‐fluorescein conjugates **10** and **11** were lower compared to **12** and **13**, which demonstrates that also colistin (and not only ubiquicidin) contributed to binding. In fluorescence microscopy images of smears of whole blood after addition of *E. coli* and **12** or **13**, the fluorescence signal was co‐localized with bacteria (with enhanced concentration at the bacterial septum), but not with human erythrocytes, thrombocytes, or white blood cells (Figure 4 b,c). This indicates that the conjugates may indeed target bacteria selectively.

As mentioned above, the *all‐* d‐analogue of Ubi_29–41_ was incorporated in **6** to achieve a better proteolytic stability of the peptide. To verify this assumption, the stability of **6** was tested in phosphate‐buffered saline (PBS) as well as in human plasma at 37 °C in comparison to **7**, the epimer of **6** that carried *all‐* l amino acids in the Ubi_29–41_ part (Supplementary Figure S6). The chemical stability in PBS was comparable for **6** and **7** (40 % and 53 % after 24 h, respectively). In plasma, **6** showed a stability of 44 % after 24 h, a value that is similar to the one obtained in PBS. In stark contrast, we observed that the plasma stability of **7** was only 5 % after 24 h. We suggest that this difference is due to plasma proteases that specifically recognize l‐amino acids, but not d‐amino acids. For this reason, all subsequent experiments were continued with analogue **6**.

As a first proof of concept for antibiotic efficacy, the activity of **6** was tested in two strains of *E. coli* (K12 and DSM 1116) in the presence or absence of neutrophil elastase. As a control, free **1** was applied. Conjugate **6** showed reduced antimicrobial activity with minimal inhibitory concentration (MIC) values of 4 and 16 μm for the two strains compared to **1**, which had MICs of 0.063 μm against both strains (Table 1 and Supplementary Figure S7). However, the activity of colistin was almost completely restored upon addition of NE at 10 mU mL^−1^ to **6**. Neutrophil elastase alone did not display any antimicrobial activity. The microbiological data were supported by LC–MS tracking of the colistin release from **6** upon treatment with NE in RPMI 1640 medium. After an incubation time of 1 h, 10 mU mL^−1^ elastase cleaved approximately 75 % of a 10 μm solution of **6** at 37 °C (Supplementary Figure S8) to generate **1**. The experiments demonstrate that the antibacterial activity of **6** is indeed shielded, but can be liberated by pure, recombinant NE. The role of the optimized NE cleavage peptide was investigated by a comparison with **49**, a conjugate that comprised the commonly used Ala‐Ala‐Pro‐Val peptide substrate for NE (Supplementary Figure S9). The antimicrobial activity of **49** was low (>32 μm) both in the presence and in the absence of neutrophil elastase (Supplementary Figure S10). Also the intermediate **48**, that was devoid of d‐Ubi_29–41_, showed low (MIC=16 μm) antimicrobial activity. These findings demonstrate that utilizing the optimized NE substrate peptide was mandatory for the concept.

**Table 1:** Antibacterial activities of colistin **1** and peptide–colistin conjugate **6** against two strains of *E. coli*. Shown are the MIC values in μm.

Compound	*E. coli* K12	*E. coli* DSM 1116
**1**	0.063	0.063
**6**	4	16
**6** with elastase^[a]^	0.125	0.125

[a] Neutrophil elastase added at 10 mU mL^−1^.

In order to probe whether the attachment position of the elastase‐cleavable linker to colistin impacts the cleavage efficacy of the conjugates, the five structurally defined regioisomers **22, 28, 34, 40**, and **46** were tested against *E. coli* K12 in comparison to free **1**. Remarkably, the intracyclic derivatives **34**, **40**, and **46** showed no activity (MIC>32 μm) in the absence of NE (Table 2 and Supplementary Figure S11). Following activation by elastase, we observed that the microbiological potency of the isomers differed markedly: The highest activity was observed for **22** and **28**, having the elastase substrate at extracyclic positions 1 and 3, respectively. **34**, bearing the elastase substrate at position 5, showed enhancement to a moderate activity (MIC=4 μm). Isomers **40** and **46**, that carried the NE substrate at positions 8 and 9, respectively, showed even lower activities. Because the microbiological activity is mostly due to free **1**, this rank order reflects differences in NE cleavage efficiency. We conclude that the most favorable Dab residue for linker attachment are the ones at positions 1 and 3 (as in **22** and **28**, respectively).

**Table 2:** Antibacterial activity of peptide–colistin isomers obtained by total synthesis against *E. coli* K12.

Compound	MIC without Elastase [μm]^[a]^	MIC with 10 mU mL^−1^ Elastase [μm]^[a]^	MIC with 100 mU mL^−1^ Elastase [μm]^[a]^
**1**	0.031	0.031	0.031
**22**	4	0.125	0.063
**28**	8	0.25	0.125
**34**	>32	4	4
**40**	>32	>32	32
**46**	>32	32	8

[a] Conservative MIC readouts of at least three independent biological experiments.

**Figure 5:**
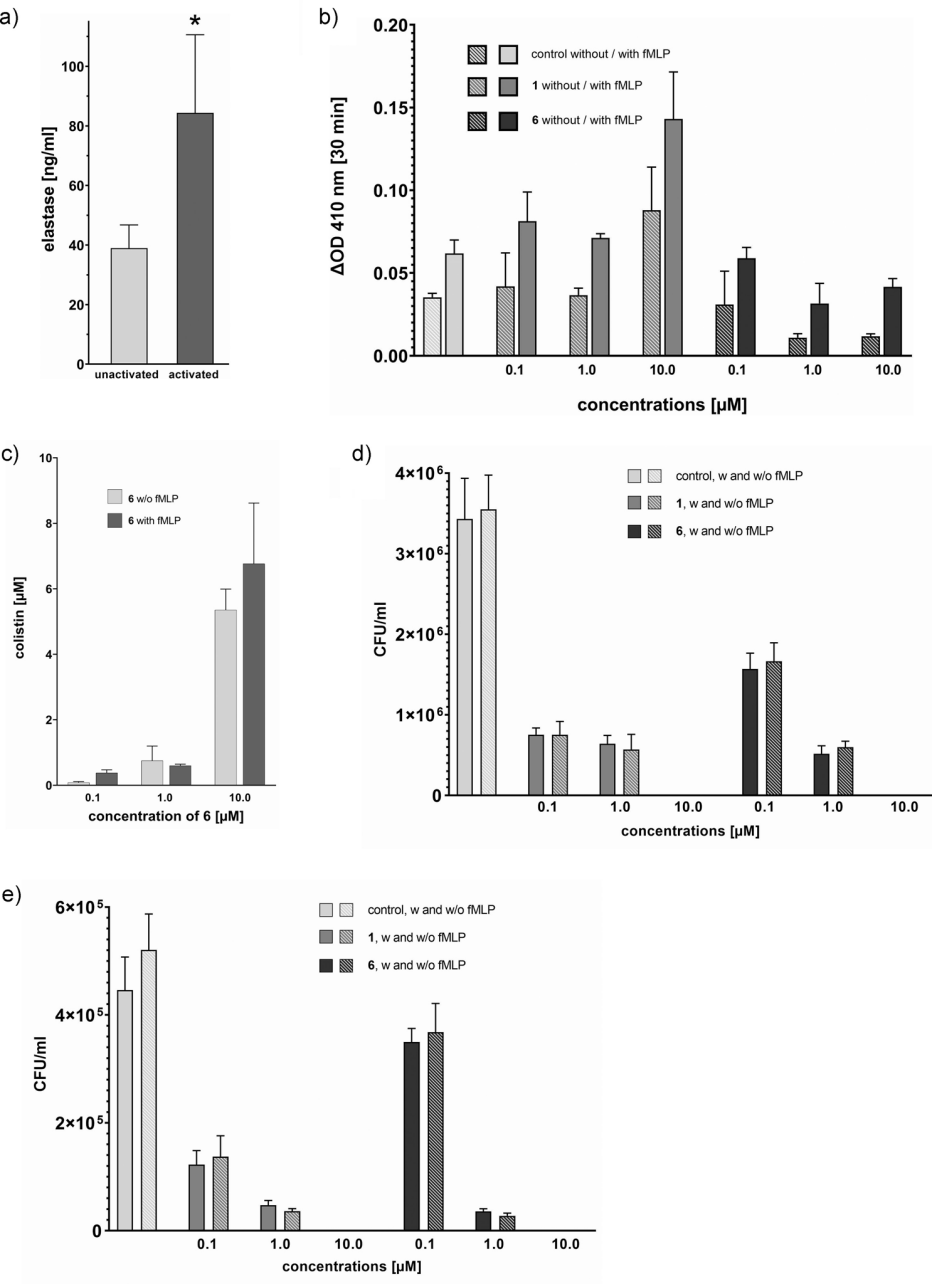
Cellular proof of concept for triggered antibiotic release from **6**. a) Release of neutrophil elastase (NE) from neutrophil granulocytes. PMN were isolated and suspended in RPMI 1640. For activation, 0.1 μm fMLP was added. The samples were incubated for 1 h at 37 °C, centrifuged to remove cells, and the NE concentration was determined in the supernatant by ELISA. Values normalized to 1×10^6^ cells mL^−1^. Data are presented as the mean of 4 separate experiments. *p<0.03 compared to unactivated. b) Activity of NE after co‐cultivation of PMN with *E. coli* K12 in presence of **1** or **6**. 5×10^5^ cells mL^−1^ of PMN were co‐cultivated with 8×10^6^ cells mL^−1^ of *E. coli* K12 (ratio of bacteria : PMN 16:1) for 1 h at 37 °C in RPMI 1640/HEPES‐NaCl 1:1. Data are presented as the mean of two separate experiments. c) Release of colistin from **6**. 5×10^5^ cells mL^−1^ of PMN suspended in RPMI 1640 were co‐cultivated with 8×10^6^ cells mL^−1^ of *E. coli* K12 for 1 h at 37 °C, and **6** was added at three concentrations. After 1 h colistin concentrations were determined by HPLC–MS. Data are presented as the mean of two separate experiments. d,e) Antimicrobial activity of **6** against *E. coli* K12 during co‐cultivation of PMN with the bacteria in RPMI 1640 (d) and in human blood plasma (e). 5×10^5^ cells mL^−1^ of PMN suspended in RPMI 1640 or human blood plasma were co‐cultivated with 8×10^6^ cells mL^−1^ of *E. coli* K12 for 1 h at 37 °C with shaking (ratio bacteria to PMN 16:1). Colistin **1** and the conjugate **6** were added at three concentrations (0.1 μm, 1 μm, and 10 μm). Data are presented as the mean of two separate experiments. *p*<0.05 for all data points compared to control (unpaired Mann–Whitney test). Whiskers present standard deviation.

Next, we addressed the question whether human neutrophil granulocytes (polymorphonuclear neutrophils, PMN) release sufficient amounts of neutrophil elastase upon stimulation for the cleavage of the linker in **6**. According to previous investigations, neutrophil granulocytes contain an average amount of 1.59 pg neutrophil elastase per cell,^[22]^ which corresponds to 1.59 μg mL^−1^ for 1×10^6^ cells mL^−1^, or to 100 mU mL^−1^.^[23]^ Thus, if about 10 % of the NE was released or functionally active on the surface of the cells upon stimulation of the granulocytes, sufficient activity should be available for a measureable cleavage of the conjugates. To probe this experimentally, PMN were isolated from fresh whole blood, taking into account that the cells are very sensitive and readily activated by different stimuli, including their physical handling. We applied a mild, so‐called untouched isolation procedure that removes contaminating cells out of suspension by iron‐conjugated antibodies and magnets.^[24]^ Three sources for the isolation of the PMN were compared: i) buffy coat, ii) the filter content from a plateletpheresis instrument, and iii) fresh venous whole blood (Supplementary Figure S12). Fresh whole blood was found to yield the highest quality PMNs with a purity of >98 % according to FACS analysis after labeling with anti‐CD15‐PE and anti‐CD16‐APC antibodies; therefore, this source was used in further studies. The addition of the chemotactic and neutrophil granulocytes activating peptide formyl‐methionyl‐leucyl‐phenylalanine (fMLP, concentration 0.1 μm) led to a 2.5‐fold increase of the activation state marker CD66b, which is in good agreement with previous data (Supplementary Figure S13).^[24]^ The amount of elastase released by neutrophil granulocytes in RPMI 1640 medium was quantified by an enzyme‐linked immunosorbent assay (ELISA). A basal amount of 39 ng/1×10^6^ cells mL^−1^ was detected, which increased to 84 ng mL^−1^ upon stimulation with 0.1 μm fMLP, corresponding to 2.5 and 5.5 mU mL^−1^, respectively (Figure 5 a). Since a considerable part of the active NE is not secreted but remains associated with the cells,^[25]^ these values are considered sufficient to exert cleavage activities in the range of 10 mU mL^−1^, as used in the antibiotic assays with recombinant NE (Tables 1 and 2). In order to address the question whether NE is not only secreted but also active, we carried out a functional assay which monitored the release of *p*‐nitroaniline at 410 nm following the cleavage of the peptidic substrate MeO‐Suc‐AAPV‐*p*NA by NE (Supplementary Figures S14 and S15).^[26]^ We observed substrate cleavage from both the supernatant or a cell suspension of PMN in RPMI 1640/HEPES medium, indicating elastase activity in the in vivo situation; in line with the literature, the cleavage was stronger (2.5‐fold under unstimulated and 1.8‐fold under stimulated conditions) in the cell suspension as compared to the supernatant.

Another potential confounder was that colistin itself might influence the activity of NE. While Jones et al.^[27]^ reported that colistin had an enhancing effect on the activity of NE in sputum samples, as well as on purified NE in vitro, the study of Hector et al.^[28]^ contradicted these findings and postulated that colistin has an inhibiting effect on the activity of NE in vitro. Since this aspect was important for our concept, we probed the influence of free colistin **1** as well as the conjugate **6** on the activity of NE in co‐cultivations of PMN with *E. coli* K12 in RPMI 1640. Whereas free **1** had an approx. 2.3–2.5‐fold enhancing effect at 10 μm, **6** reduced the activity at 1.0 and 10 μm to approximately 33 % without fMLP and to 50–66 % with fMLP (Figure 5 b). The inhibitory effect of **6** might be due to its competition with the assay substrate peptide MeO‐SucAAV‐pNA for the occupation of the NE active site. On the other hand, the increase of activity by colistin itself was considered to be favorable for our approach, since it may lead to an auto‐enhancement of the liberation of **1** from the conjugate. Next, we checked whether the exposure of PMN to *E. coli* bacteria could trigger the release of colistin from the conjugate **6** (Figure 5 c). In RPMI 1640, cleavage efficiencies of 74 %, 80 %, and 50 % were found at concentrations of **6** of 0.1, 1.0, and 10 μm, respectively. The addition of fMLP had no significant effect. The cleavage efficiency of the two lower concentrations correlates well with the one obtained with pure protein (Supplementary Figure S8); the reduced rate for 10 μm substrate may reflect a limiting concentration of the enzyme. In plasma, free **1** could not be detected under the experimental conditions (for a discussion see below). Overall, we conclude from the ELISA‐based quantification of NE and from the activity‐based functional assays that PMN can indeed provide a sufficient amount of enzyme to cleave NE‐sensitive drug conjugates and to release colistin.

Finally, the crucial proof‐of‐concept experiment addressed the question whether the conjugate **6** can reduce the number of viable bacteria in a co‐culture of PMN with *E. coli*. For this purpose, bacteria and PMN in a ratio of 16:1 were co‐cultivated for 1 h in RPMI 1640 and in blood plasma. We were pleased to observe that 0.1 μm and 1.0 μm of **6** reduced bacterial growth by 53 % and 83 %, respectively, and abolished growth completely at 10 μm in RPMI 1640 (Figure 5 d). This efficacy was equal to that of the positive control **1**. Again, the addition of 0.1 μm fMLP had no effect. To mimic a relevant in vivo matrix, equivalent experiments were performed in blood plasma. Again, **6** reduced growth by 29 % and 95 % at 0.1 and 1.0 μm, respectively, which was comparable to 74 % and 97 % reductions by colistin at equal concentrations (Figure 5 e). Both compounds abolished growth completely at 10 μm.

Despite the fact that we did not detect free **1** in plasma upon addition of **6** to a co‐culture of PMN and *E. coli* K12, the conjugate clearly reduced the number of viable bacteria according to the CFU test. We hypothesize that the released amount of **1** was sufficient to exhibit antibacterial activity, but fell below the limit of detection for colistin in plasma (LOD=50 nm, Supplementary Figure S16).

The released colistin will be eliminated from the body mainly by renal clearance, and the same renal toxicity mechanisms as for unconjugated colistin are operative.^[6a]^ But due to the targeting of the drug to the bacterial membrane, we expect that the systemic dose required to achieve an effective dose at the site of infection can be lowered; in addition, the fraction of conjugate not reaching the infection site is eliminated in a protected prodrug form; both factors should contribute to lower renal exposure to free colistin and lower renal toxicity. However, the proof of these conceptual considerations needs to be given in further in vivo investigations.

## Conclusion

We have validated a new concept for antibiotic conjugates that are activated by the pathogen at the site of infection. Mechanistic experiments demonstrate that **6** can initiate and accomplish the complex sequence of events outlined in Figure 1 c: direction of the antibiotic conjugate to bacterial pathogens, secretion of a proteolytic enzyme from immune cells upon exposure to the pathogen, enzymatic release of antibiotic from the conjugate, and finally bacterial killing. Compared to previous work with bifunctional constructs[^10^, ^11^, ^12^] our trifunctional approach principally enables a more directed targeting to the bacterial surface and more focused release of the drug at the infection site; in addition, the compounds represent defined, single molecular entities. The large molecular size would restrict an application of the conjugates to parenteral modes of administration. However, such antibiotics would be used in hospital settings for the treatment of life‐threatening infections, where the highly polar peptide conjugates are well‐suited for intravenous administrations that constitute the preferred clinical standard.

In future studies, we aim at expanding the concept to additional Gram‐negative, clinically important pathogens, and to probe its in vivo efficacy.

## Conflict of interest

The authors declare no conflict of interest.

## Supporting information

As a service to our authors and readers, this journal provides supporting information supplied by the authors. Such materials are peer reviewed and may be re‐organized for online delivery, but are not copy‐edited or typeset. Technical support issues arising from supporting information (other than missing files) should be addressed to the authors.

Supporting Information
