# Supplementary material for: Selective Bacterial Targeting and Infection‐Triggered Release of Antibiotic Colistin Conjugates
Source: Angew Chem Int Ed Engl. 2021 Jul 5;60(33):17989–97. doi: 10.1002/anie.202104921 (PMC8456958; doi:10.1002/anie.202104921)
Supplement: Supplementary file 1 — Supporting Information [file ANIE-60-17989-s001.pdf]

Angewandte Chemie
Eine Zeitschrift der Gesellschaft Deutscher Chemiker

Supporting Information

Selective Bacterial Targeting and Infection-Triggered Release of Antibiotic Colistin Conjugates

*Werner Tegge, Giulia Guerra, Alexander Höltke, Lauritz Schiller, Ulrike Beutling, Kirsten Harmrolfs, Lothar Gröbe, Hannah Wullenkord, Chunfa Xu, Herbert Weich, and Mark Brönstrup**

anie_202104921_sm_miscellaneous_information.pdf

Author Contributions

W.T. designed the study, performed experiments, analyzed the data, and wrote the manuscript. G.G., A.H., L.S., U.B., K.H., L.G., H.W., C.X., H.W. performed experiments and analyzed the data. M.B. designed the study, analyzed the data, acquired funding, and wrote the manuscript. All authors read and approved the manuscript.

Table of Content

1 **Materials** 3

2 **Methods** 4

3 **Ethical approval** 7

4 **Biological experiments** 8

5 **Supplementary Figures** 14

Figure S1: Total synthesis of 28, modified at position 3 14

Figure S2: Total synthesis of 34, modified at position 5. 15

Figure S3: Total synthesis of 40, modified at position 8. 16

Figure S4: Total synthesis of 46, modified at position 9. 17

Figure S5. Semisynthesis of 12 and 13. 18

Figure S6. Plasma stabilities of 6 and 7. 19

Figure S7. Heat plot of the growth of *E. coli* K12 and *E. coli* DSM 1116 in presence of 6. 20

Figure S8. Cleavage of 6 by neutrophil elastase. 21

Figure S9. Semisynthesis of 49 22

Figure S10. Heat plot of the growth of *E. coli* in presence of 48 and 49 23

Figure S11. Heat plot of the growth of *E. coli* in presence of positional isomers obtained by total chemical synthesis 24

Figure S12. Purity of neutrophil granulocytes after isolation from different sources 25

Figure S13. Activation of human neutrophil granulocytes with fMLP 25

Figure S14. Activity of secreted NE as determined by the release of p-nitroanilin upon cleavage of the substrate peptide MeO-SucAAPV-pNA 26

Figure S15. Activity of neutrophil elastaste in different media as determined by the release of p-nitroanilin upon cleavage of the substrate peptide MeO-SucAAPV-pNA 27

Figure S16. Standard curve for the quantification of colistin B by LC-MS/MS 27

6 **Compound synthesis** 28

7 **Analytical Data** 45

1. HPLC Data 45

2. NMR Data 93

Table S1. NMR data of colistin B (1) in D_2O 93

Table S2. NMR data of fragment of 22 in D_2O 95

Table S3. NMR data of fragment of 28 in D_2O 96

Table S4. NMR data of fragment of 34 in D_2O 97

Table S5. NMR data of fragment of 40 in D_2O... 98
Table S6. NMR data of fragment of 46 in D_2O... 99
Figure S17. Sections of conjugate structures 22, 28, 34, 40 and 46. 100
Figure S18. Overlay of HSQC spectra.. 101
Figure S19. Structure of D-Ubi-Ela-Sub (50). 102

1 Materials

Colistin sulfate was obtained from Cayman Chemical (via Biomol, Hamburg, Germany) and separated into the two major components, colistin A (eluting at 41% acetonitrile (ACN)) and colistin B (the major product, eluting at 40% ACN), by prep. HPLC on C-18 as shown below. Colistin B as the major product was used as a TFA salt as obtained after prep. HPLC, or after converting the TFA salt into the HCl salt by lyophilizing a 1 mM peptide solution three times in 5 mM aqueous HCl, if indicated in the particular procedure.

Amino acid derivatives were obtained from the following suppliers: D- and L-Fmoc-Lys(N_3)-OH, Fmoc-L-Bpa-OH (CAS Nr. 117666-96-3) and Fmoc-L-Oic-OH (CAS Nr. 130309-37-4): Carbolution (St. Ingbert, Germany). 2-Chlorotrityl choride resin, Fmoc-2-Abu-OH, Fmoc-L-Met(O_2)-OH, Fmoc-Dab(Boc)-OH, Fmoc-Dab(Dde)-OH, Dde-Dab(Fmoc)-OH, PyBOP, HATU and HCTU: Iris Biotech (Marktredwitz, Germany). Rapp S RAM resin: Rapp Polymere, (Tübingen, Germany). Other Fmoc-amino acids: MultiSynTech (Witten, Germany). Side chain protections were as follows. Asn and Cys: Trt; Arg: Pbf; Lys: Boc; Thr and Tyr: tBu.

Cedex Trypan Blue solution (Roche Diagnostics, Mannheim, Germany, order No. 05650640001).

The following bacterial strains were used in this study: *Acinetobacter baumannii* DSM 30007 (ATCC 19606); *Escherichia coli* K12 (DSM 498); *E. coli* E2348/69 (serotype O127:H6, EPEC), *E. coli* 536 (serotype 06:H31, UPEC), *Klebsiella pneumoniae* C122 (DSM 11678), *Pseudomonas aeruginosa* PA7 (DSM 24068), *P. aeruginosa* PA14 (DSM 19882).

Media and biological reagents were prepared or purchased as follows:

T-medium: 17 g/l LP 42 (Oxoid), LP44 (Oxoid) 3.0 g/l, Glucose 10.0 g/l, MOPS 10.47 g/l, NaCl 5.84 g/l, KCl 0.149 g/ml, $CaCl_2$x 2 H_2O 0.294 g/l, PH 7.4, sterile filtrated.

RPMI 1640 with phenol red (Gibco/ThermoFisher); HBSS (Hank's Balanced Salt Solution, Gibco/ThermoFisher).

Neutrophil Elastase (human), affinity purified: USBiological (via distributor Biomol GmbH, Hamburg, Germany), No E2230-11. Activity stated: 72 U/ml. On arrival, the solution with the enzyme was divided into aliquots of 5 µl that were stored at -70 °C until use.

MACSxpress® whole Blood Neutrophil Isolation Kit, human and antibodies for FACS analysis: anti-CD15-PE, anti-CD16-APC, anti-CD63-FITC and anti-CD66b-FITC: Miltenyi Biotec (Bergisch-Gladbach, Germany).

N-Formyl-Met-Leu-Phe (fMLP) and N-Methoxysuccinyl-Ala-Ala-Pro-Val-p-nitroanilide (MeOSuc-AAPV-pNA): Sigma-Aldrich.

ELISA for neutrophil elastase: DuoSet ELISA® Human Neutrophil Elastase/ELA2, Streptavidin-HRP, Mouse Anti-Human ELA2 Capture Antibody, Biotinylated Mouse Anti-Human ELA2 Detection Antibody, Recombinant ELA2 Standard, HRP substrate (1:1 mixture of reagent A (H_2O_2) with reagent B (Tetramethylbenzidine), Reagent Diluent (1% BSA in PBS), color reagent A and color reagent B were from R&D Systems (Minneapolis, USA).

2 Methods

Liquid chromatography coupled to mass spectrometry (LC-MS): LC-MS data were recorded on an ion trap mass spectrometer (Amazon SL, Bruker Daltonics, Bremen, Germany) equipped with an Apollo II electrospray source. LC was performed with an Agilent 1200 Series HPLC system (Waldbronn, Germany) equipped with binary pump, autosampler and diode array detector on a Gemini NX 3 µm C18 110 Å, 50×2 mm column (Phenomenex, Aschaffenburg, Germany). The flow rate was 400 µL/min. The solvents (A: water with 0.1% formic acid and B: ACN 0.1% formic acid) were eluted with a gradient from 5% B (0 min), to 80% B (17 min), to 95% B (19 min), to 95% B (21 min). Sample injections were typically 10 µL.

Quantifications of colistin and peptide-colistin constructs were carried out via LC-MS on a triple quadrupole mass spectrometer (AB Sciex Qtrap 6500, Dreieich, Germany) with an UHPLC (Agilent 1290) for separation. LC conditions were as follows: Xselect CSH phenyl-hexyl column (4.6 x 50 mm, 2.5 µm, Waters, Eschborn, Germany), column temperature 30°C, solvent A: water with 0.1% formic acid and B: ACN 0.1% formic acid, flow rate: 700 µl/min. Gradient: 0 min 1% B, 0.5 min 1% B; 4 min 100% B; 4.5 min 100%; followed by reconditioning of the column. The overall run time was 8 min. The mass spectrometer was used in ESI-MRM mode, curtain gas 20 psi (N_2), ion spray voltage 5500 V, temperature 400 °C, gas 1: 45 psi and gas 2: 65 psi (both compressed air). The first transition was used as quantifier, the second as qualifier. Dwell time per transition: 50 msec. Colistin (**1**): 289.7 ($[M+4H]^{4+}$) → 67 (quant, declustering potential 46 V, collision energy 55 V, cell exit potential 10 V) and 289.7 → 44 (qual; declustering potential 46 V, collision energy 63 V, cell exit potential 20 V); Ubi-Ela-Sub-Colistin-construct (**6** and **7**): 544.4 ($[M+7H]^{7+}$) → 400.3 (quant; declustering potential 91 V, collision energy 25 V, cell exit potential 24 V) and 544.4 → 483.2 (qual; declustering potential 91 V, collision energy 19 V, cell exit potential 30 V).

HR-MS data for colistin and colistin conjugates were acquired with an UHR-TOF mass spectrometer (Maxis HD, Bruker, Bremen, Germany), equipped with an Apollo II electrospray source. The samples were directly infused into the mass spectrometer. Na-formiate clusters were used for the internal calibration, and additionally a lock mass calibration was carried out with hexakis(2,2-difluoroethoxy)phosphazene. Internal and lock mass calibration were carried out with the program DataAnalysis (Bruker, Bremen, Germany). MS-acquisition parameters: source type ESI, scan range 50-1500 *m/z*, ion polarity positive, capillary voltage 4500 V, nebulizer pressure 4.0 bar, dry heater 200 °C, dry gas 9.0 l/min. Injection volume 0.5 µl.

Nuclear Magnetic Resonance (NMR) data: NMR experiments were performed on a Bruker Avance III HD 700 MHz spectrometer, equipped with a triple resonance 5 mm Helium cooled CryoProbe (TCI), at 298 K. All data were analyzed with Bruker Topspin 3.6.2. Chemical shift values of ^{1}H- and ^{13}C-NMR

spectra are reported in ppm relative to D_2O δ_H 4.79 and TFA δ_{CF3} 116.6 ppm given as an internal standard. ^{13}C-signals were assigned via 2D-CH and CCH correlations (HSQC and HMBC) for conjugates **22**, **28**, **34**, **40** and **46** and in addition from direct ^{13}C detection for **1**. Multiplicities are described using the following abbreviations: s = singlet, d = doublet, t = triplet, q = quartet, m = multiplet, b = broad; corrected coupling constants are reported in Hz.

Peptide Synthesis General: Fmoc peptide syntheses were carried out on a Syro Multiple Peptide Synthesizer (MultiSynTech, Witten, Germany) with HCTU / diisopropylethyl amine activation with tenfold excess of the amino acids and 1 hour coupling time, if not stated otherwise. Side chain protections of amino acids were as follows: Arg: Pbf, Asn, Cys and Gln: Trt, Dab: Boc or Dde, Lys: Boc, Thr and Tyr: tBu. Dab was also used as Dde-Dab(Fmoc)-OH. Peptide amides were generated on Rapp S RAM resin (loading 220 µmol/g, Rapp Polymere, Tübingen Germany), C-terminal peptide acids were generated on Rapp S PHB resins preloaded with the respective amino acid, as stated in the particular procedure. Unprotected peptides were deprotected and cleaved from the resin by a 3 h treatment with TFA containing 3% triisopropylsilane and 2% H_2O (10 ml/g resin) or with reagent KW (82.5% TFA, 5% H_2O, 5% phenol, 5% thioanisol, 2.5% dithiothreitol (v/w or v/v)) if indicated in the particular procedure. The resin was filtered off, the filtrate was concentrated to a few ml and t-butylmethyl ether was added to precipitate the crude peptides, which were isolated by centrifugation. Side-chain protected peptides were assembled on 2-chlorotrityl chloride resin and cleaved from the solid support by slowly passing 40 ml 1,1,1,3,3,3-hexafluoro-2-propanol (HFIP) / DCM 1:4 (v/v) through the resin over a period of 1 h. The volume of the filtrate was reduced to a few ml, an excess of 1,4-dioxane was added and the solution was lyophilized to yield the crude protected peptides. General procedure for coupling the first amino acid to 2-chlorotrityl chloride resin: 2-Chlorotrityl chloride resin (1.0 g, 1.55 mmol capacity) was suspended in anhydrous DCM (5 mL) in a 10 mL polypropylene syringe fitted with a polyethylene disc. Fmoc-amino acid (1 mmol) and DIPEA (523 µL, 3 mmol) were added and the mixture was gently stirred at RT for 2 h. Afterwards the resin was washed with DCM (3 x 3 mL), DMF

(3 x 3 mL) and DCM again (3 x 3 mL) and allowed to dry. Loading was determined by quantitative photometric determination of the Fmoc group after its cleavage with 20% piperidine in DMF (10 min at rt)(Lit).

For the calculation of yields and the concentration of stock solutions, the counter ion trifluoroacetic acid (mol. weight 114) for every basic amino acid and the free N-terminus was taken into account, where appropriate. Concentrations of stock solutions of the fluorescein-labelled compounds **10-13** were based on photometric determinations in 5% aqueous TFA with the extinction coefficient ε_{440} = 42985, which was predetermined with fluorescein-5-maleimide.

HPLC: Analytical HPLC was carried out on Luna C18, 5µ, 2x50 mm columns (Phenomenex, Aschaffenburg, Germany), flow rate 0.7 ml/min, with 18 min linear gradients of 5% ACN in water to 100% ACN (solvents with 0.1% TFA). Peak detection at 220 nm. Preparative HPLC was carried out on a 4 µm 21.2 x 250 mm Jupiter Proteo column (Phenomenex, Aschaffenburg, Germany) with 60 min linear gradients of 5% ACN in water to 50-80% ACN (depending on the particular product, solvents containing 0.1% TFA). Flow rate 15 ml/min. Fractions containing pure products were lyophilized, protected peptides without prior evaporation.

Statistics: Analysis for significance were carried out with the program GraphPad Prism (Version 8.4.2) by Mann-Whitney test or unpaired two sample Welch t-test, as indicated in the particular graph.

3 Ethical approval

Blood sampling for the project was approved by the ethics committee of the Medical Association Lower Saxony, Germany on July 3, 2018 (reference number BO/22/2018). Informed, signed consent was obtained from the blood donors.

4 Biological experiments

Blood sampling

Peripheral blood of healthy female and male volunteers, aged of 30 to 62 years, was sampled from the arm veins with a Safety Multifly 21G system in 7.5 ml S-Monovette K3E containing EDTA. PMN cells were isolated within 1 h after blood sampling.

Cell counting: 20 µl of a cell suspension were mixed with 20 µl Cedex Tryptan Blue Solution. 10 µl of the mixture were measured automatically in a cell counter (Cedex XS Cell Analyzer, Roche) in duplicate.

Quantification of bacterial binding of fluorescently labelled compounds

The bacterial strains *A. baumannii*, *E. coli* K12 and *P. aeruginosa* PA14 were cultivated in T-medium at 37 °C to exponential growth (OD_{600} = 2-3). The OD_{600} was then adjusted to 1.0 and the cells were collected by centrifugation at 5,000 x g for 5 min at 4 °C. Bacterial pellets were resuspended in 1 ml PBS and compounds 5(6)-carboxyfluorescein, **10**, **11**, **12** and **13** were added at 1 µM, followed by 1 h incubation at rt without shaking. Afterwards, the bacteria were washed twice with PBS, suspended at OD_{600} = 0.2 in PBS and fluorescence was measured at 485/520 nm with 200 µl of bacterial suspensions in black 96-well microtiter plates from above in triplicate.

Microscopic investigation of bacterial staining in whole blood

E.coli K12 was cultivated to exponential growth in T-medium at 37 °C and adjusted to OD_{600} = 1.0. Bacteria from 1 ml of the suspension were collected by centrifugation (5,000 x g, rt, 2 min) and resuspended in 50 µl PBS. The suspension was added to 440 µl whole blood, and 10 µl of aqueous stock solutions of **12** and **13** were added at final concentrations of 1 µM. The mixture was incubated for 10

min at rt, and blood smears were prepared on microscopy slides that were air-dried in the dark. Phase contrast and fluorescence (485/520 nm) microscopy pictures were captured with a Zeiss Axiovert with a 40× APO 1.4 lens. For quantification of fluorescence, pictures were analyzed by using ImageJ (v1.52). Outlines were drawn around each cell and circularity, area and mean fluorescence were determined. In addition, several background readings were taken at empty areas. Total corrected cellular fluorescence (TCCF) = integrated density – (area of selected cell × mean fluorescence of background readings), was calculated for 200 bacteria for each assay.

Determination of antibacterial activities by broth dilution assay

Bacterial suspensions at 5-6 x 10^5 CFU (1:1000 dilution of a bacterial suspension of OD_{600} = 1) in T-medium were incubated in volumes of 200 µl in sterile clear 96-well microtiter plates at 37 °C in a wet chamber with serial dilution of peptide-colistin constructs and controls (32 – 0.016 µM) for 18 h. External neutrophil elastase was added at 10 mU/ml or 100 mU/ml to each well at the start of incubation, where indicated. After the incubation, the plate was placed for 5 min on a MTP shaker to generate homogenous bacterial suspensions. OD_{600} was then determined in a microplate reader. The wells with the lowest compound concentration without growth defined the MICs.

Quantification of 1, 6 and 7

To 50 µl samples 50 µl acetic acid were added and the samples were filtered through 10 kDa ultrafiltration columns (Amicon Ultra 0.5 ml, Millipore) by centrifugation for 60 min at 14.000 x g and 4 °C. The columns were washed 1 x with 300 µl of 50% acetic acid and 1 x with 300 µl of 50% 2-propanol, again followed by centrifugation for 60 min at 14,000 x g at 4°C. The solvents of the combined filtrates were evaporated and the residues were redissolved in 50 µl of 50% aqueous ACN. Colistin was quantified on a Q-Trap 6500 HPLC-MS with samples of 5 µl each as described above. All tests and all

HPLC-MS measurements were carried out in triplicate. Data from MRM measurements were analyzed with Skyline (vers. 18.3, MacCoss Laboratory, University of Washington).

Isolation of Neutrophil Granulocytes from human blood

The MACSxpress Neutrophil Isolation Kit, human from Miltenyi Biotec (Bergisch-Gladbach, Germany) was employed. Fresh venous EDTA blood was processed within 1 h. 65 mg of the MACSxpress whole blood isolation cocktail (lyophilized powder) were mixed with 1 ml of buffer A (from the kit). Then 1 ml of buffer B (from the kit) was added, and the well-mixed cocktail was added to 4 ml of peripheral whole blood in a 15 ml tube. The mixture was gently inverted 3 times and mixed on a tube rotator at 50 rpm for 5 min at rt. The tube was placed, without cap, for 20 min into a strong magnetic field (neodymium magnets). The suspension containing the PMN was collected into a fresh 15 ml tube by careful pipetting. For removal of erythrocytes, the collected supernatant was centrifuged at 200 x g for 5 min at 4 °C. After removal of the supernatant by pipetting, the cells were resuspended in 1 ml H_2O at 4 °C for exactly 20 sec, followed by the addition of 10 ml of RPMI 1640. The suspension was centrifuged at 200 x g for 10 min at 4 °C. The supernatant was removed and the cell pellet was resuspended in an optional volume of buffer or plasma for further experiments.

Flow Cytometry

All steps were carried out with cooled media (4 °C) and the vials were kept on ice. Freshly isolated neutrophil granulocytes in RPMI 1640 were counted, followed by centrifugation at 200 x g for 10 min at 4 °C. The cells were resuspended at $1x10^5$ - $5x10^5$ cells/ml in PBS with 2% FBS (FACS buffer). 5 µl of the antibodies CD15 PE, CD16 APC and CD66b PE (stock solution from the manufacturer) were added to 100 µl of the cell suspension, as desired. The samples were mixed gently and kept for 20 min in the dark. 1 ml of the FACS buffer were added with gentle mixing, followed by centrifugation at 100 x g for

5 min at 4 °C. After repeating the washing, the pellet was suspended in 500 µl FACS buffer and the cells were investigated with a cell analyzer (FACSCanto II, Becton Dickinson, Franklin Lakes, USA).

ELISA for neutrophil elastase

Plate preparation: Clear flat-bottom 96-well half area microtiter plates were coated with 50 µl of the capture antibody (R&D Systems) that was diluted with 1% BSA in PBS to 4.0 µg/ml. The sealed plate was incubated for 18 h at rt without shaking. Afterwards, the capture antibody was aspirated and the wells were washed with 0.1% Tween 20 in PBS (wash buffer). The washing step was repeated twice with manual aspiration. Blocking was carried out by adding 200 µl of Reagent Diluent (1% BSA, Fract. V in PBS) to each well and incubation for 1.5 h at 37 °C. Afterwards, the plates were washed 3 times with wash buffer.

Sample preparation: The samples were centrifuged at 500 x g for 5 min at 4 °C. Next, the supernatant was collected in a fresh 1.5 ml reaction tube and 5 µl of alpha1-antitrypsin solution (1 mg/ml) was added. The mixture was incubated for 40 min at RT on a shaker at 800 rpm.

Assay procedure: The sample and the standard were diluted with Reagent Diluent. 50 µl per well were added into the pre-coated half area microtiter plate. The wells were sealed and incubated for 1.5 h at 37 °C without shaking. After the incubation, the plate was washed 3 times with 200 µl of wash buffer. 50 µl of the diluted detection antibody (diluted with Reagent Diluent) with a final concentration of 250 ng/ml were added and incubated for 1.5 h at 37 °C. After three washing steps with wash buffer 50 µl of the 40-fold diluted streptavidin conjugated HRP was added for 20 min in the dark at rt. The wells were washed 5 times with the wash buffer and 5 times with PBS. Subsequently, 50 µl of the substrate for HRP was added and the mixture was incubated in the dark at RT. The reaction was stopped with 25 µl 2N H_2SO_4 after sufficient color intensity was reached. The absorbance was measured at 450 nm together with a correction wavelength of 570 nm, the value of which was subtracted.

Calculation of results: All samples and standards were measured in duplicate. The standard curve was constructed by plotting the mean absorbance for each standard on the y-axis against the concentration on the x-axis. A linear regression analysis was performed and the results for the samples were calculated. All results were normalized to a concentration of 1x 10^6 cells/ml.

Activity test for neutrophil elastase

For optimization, RPMI 1640, HEPES-NaCl (100 µM HEPES, 0.5 M NaCl, pH 7.4, a 1:1 mixture of RPMI 1640/HEPES-NaCl, HBSS (Hank's Balanced Salt Solution) and PBS were investigated (Figure S15). HEPES-NaCl/RPMI 1640 1:1 was found to give the best results and was used in all experiments further on. Assay procedure: 100 µl sample in RPMI 1640 or blood plasma were added to 100 µl HEPES-NaCl in a clear 96-well flat bottom microtiter plate. MeOSuc-AAPV-pNA from a 10 mM stock in DMSO was added at 100 µM. 100 µM fMLP was added as indicated in the particular experiment. The plate was incubated in a microplate reader for 60 min at 37 °C with shaking for 5 sec at 50 rpm once per minute. Every minute readings were taken at 410 nm. For the calculation of the cleavage velocity, the difference in absorbance after an initialization phase at 15 min and a further incubation time of 30 min was used and related to total cleavage of the substrate. The blank was subtracted from the readings. Results were normalized to 1x 10^6 cells/ml.

Infection assay in RPMI 1640 and in human plasma

5x10^5 cells/ml of PMN suspended in RPMI 1640 or in freshly prepared human EDTA-plasma were co-cultivated with 8x10^6 cells/ml of *E. coli* K12 for 1 h at 37 °C with shaking (ratio bacteria to PMN 16:1). **6** and **1** were added at the concentrations 0.1 µM, 1.0 µM and 10 µM. In parallel, the assays were carried out in presence of 0.1 µM fMLP. 100 µl of the suspensions were used in a CFU test. The samples were diluted several times 10-fold (based on the results from pretests) each with T-medium. 100 µl of the dilutions were spread out on a 10 cm LB agar plate and the plates were incubated for 16 h at 37

°C. Colonies were counted upon visual inspection and the numbers were multiplied with the dilution factor in order to determine the colony-forming units per milliliter (CFU/ml).

Stability test in human plasma

For each biological condition, four individual samples were used (four technical replicates). The compounds were incubated at 10 µM in 100 µl human plasma (from pooled human blood, reconstituted from lyophilisate, Sigma P9523) in quadruplicate at 37 °C with shaking. After the indicated incubation times at 37 °C, 80 µl from each sample were added to 100 µl acetic acid in Amicon Ultra 0.5 ml 10 kDa ultrafiltration membranes (regenerated cellulose, the membranes were pre-washed with 300 µl 50% AcOH and 300 µl water) and filtered by centrifugation at 14,000 x g. Membranes were washed with 200 µl of 50% AcOH and 200 µl of 2-propanol/water 1:1. Solvents from combined filtrates were removed under vacuum and residues were dissolved in 80 µl ACN/water 1:1.

5 Supplementary Figures

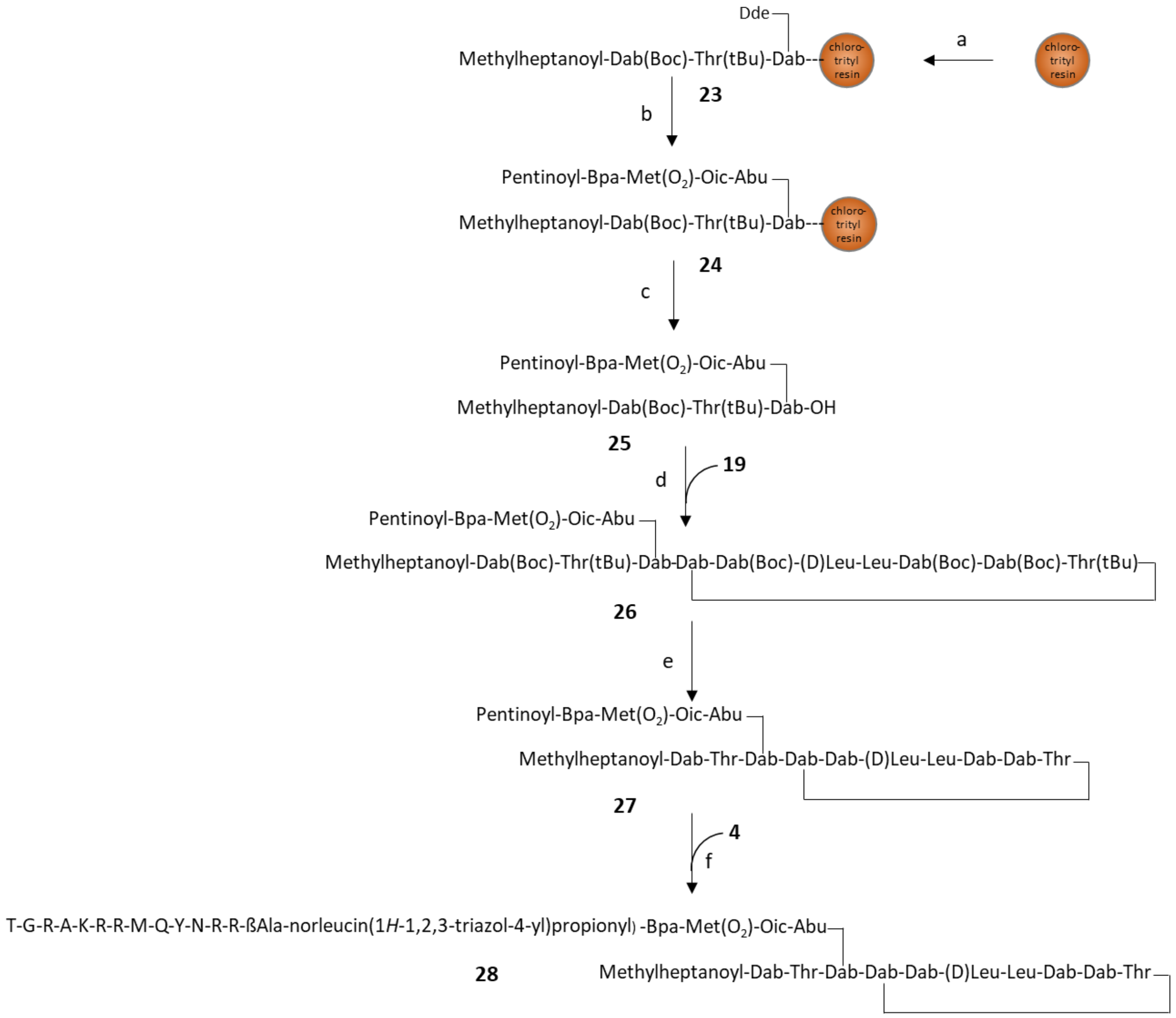

Figure S1: Total synthesis of 28, modified at position 3. (a) i) Fmoc-Dab(Dde)-OH, DIPEA, DCM, 2 h; ii) piperidine (20%) in DMF, 10 min; iii) appropriate amino acid and 6-methyl heptanoic aicd, each with activation by HCTU and DIPEA, DMF, 1 h and followed by piperidine (20%), DMF, 10 min; (b) i) hydrazine (20 mM) in DMF, 3 × 90 min, ii) Fmoc-Abu-OH, Fmoc-Oic-OH and Fmoc-Met(O_2)-OH, each with activation by HCTU and DIPEA, DMF, 1 h, and followed by piperidine (20%) in DMF, 10 min; iii) Fmoc-Bpa-OH, HATU, DIPEA, DMF, 1 h as double coupling, followed by piperidine (20%), DMF, 10 min, iii) 4-pentinoic acid, HCTU, DIPEA, DMF, 1 h; (c) 1,1,1,3,3,3-hexafluoro-2-propanol (HFIP) / DCM 1:4, 1 h, followed by RP-HPLC purification; (d) i) **19** (1 equiv.), PyBOP, HOBt, DIPEA, DMF, 5 h; (e) TFA (82.5%), H_2O (5%), phenol (5%), thioanisol (5%), dithiothreitol (2.5%), 90 min, followed by RP-HPLC purificatin; (f) **4** (1 equiv.), THPTA (200 µM) Na-ascorbate (2 mM), $CuSO_4$ (100 µM), pH 7.0, 2 h, followed by RP-HPLC purification.

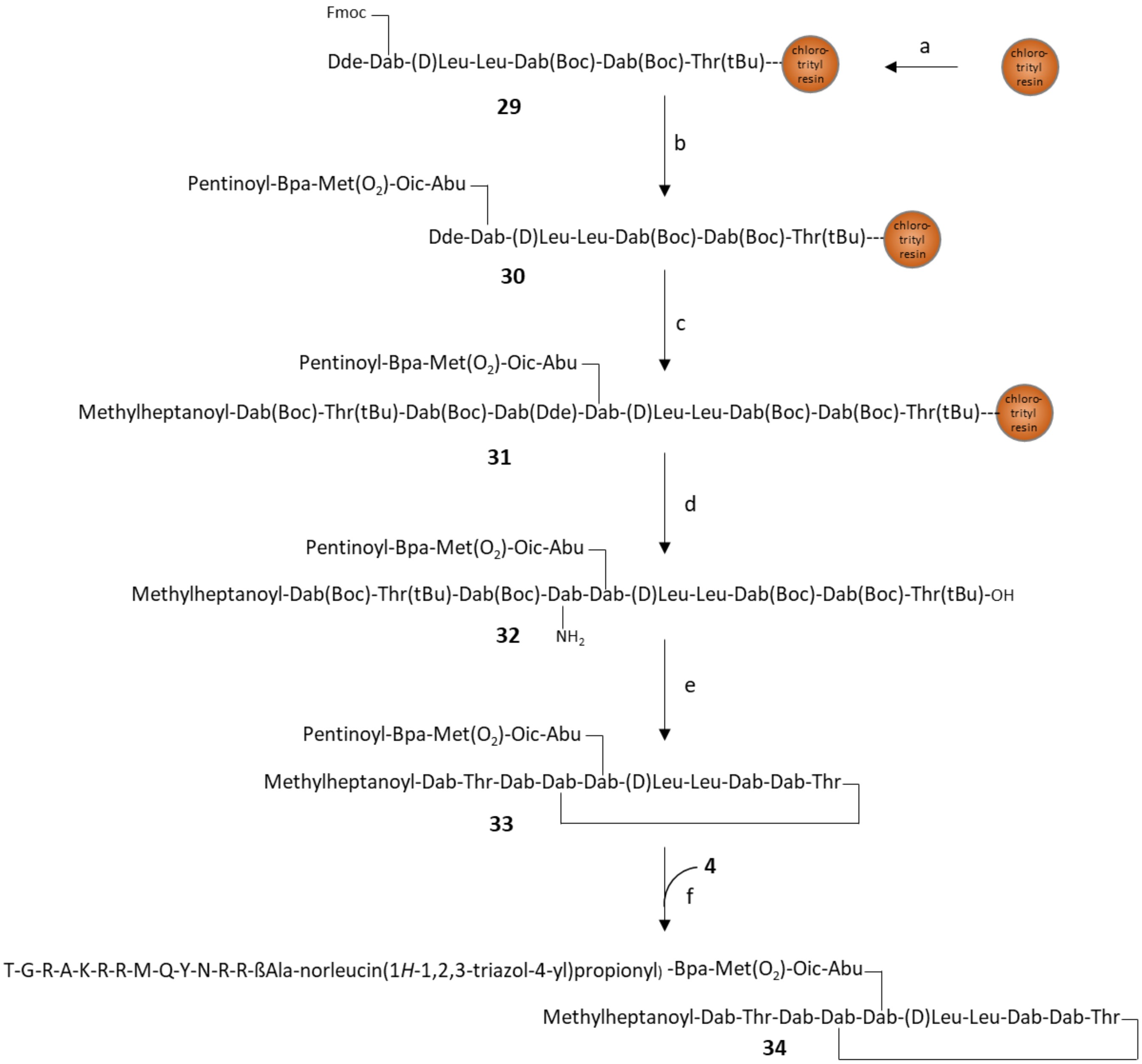

Figure S2: Total synthesis of 34, modified at position 5. (a) i) Fmoc-Thr(tBu)-OH, DIPEA, DCM, 2 h; ii) piperidine (20%) in DMF, 10 min; iii) appropriate amino acid, each with activation by HCTU and DIPEA, DMF, 1 h and followed by piperidine (20%), DMF, 10 min; (b) i) Fmoc-Abu-OH, Fmoc-Oic-OH and Fmoc-Met(O_2)-OH, each with activation by HCTU and DIPEA, DMF, 1 h, and followed by piperidine (20%) in DMF, 10 min; ii) Fmoc-Bpa-OH, HATU, DIPEA, DMF, 1 h as double coupling, followed by piperidine (20%), DMF, 10 min, iii) 4-pentinoic acid, HCTU, DIPEA, DMF, 1 h; (c) i) hydrazine (20 mM) in DMF, 3 × 90 min; ii) appropriate amino acid and 6-methyl heptanoic aicd, each with activation by HCTU and DIPEA, DMF, 1 h and followed by piperidine (20%), DMF, 10 min; (d) i) hydrazine (20 mM) in DMF, 3 × 90 min; ii) 1,1,1,3,3,3-hexafluoro-2-propanol (HFIP) / DCM 1:4; (e) i) DIC, HOBt, DCM/DMF 49:1, 5 h; ii) TFA (82.5%), H_2O (5%), phenol (5%), thioanisol (5%), dithiothreitol (2.5%), 90 min, followed by RP-HPLC purification; (f) **4** (1 equiv.), THPTA (200 µM) Na-ascorbate (2 mM), $CuSO_4$ (100 µM), pH 7.0, 2 h, followed by RP-HPLC purification.

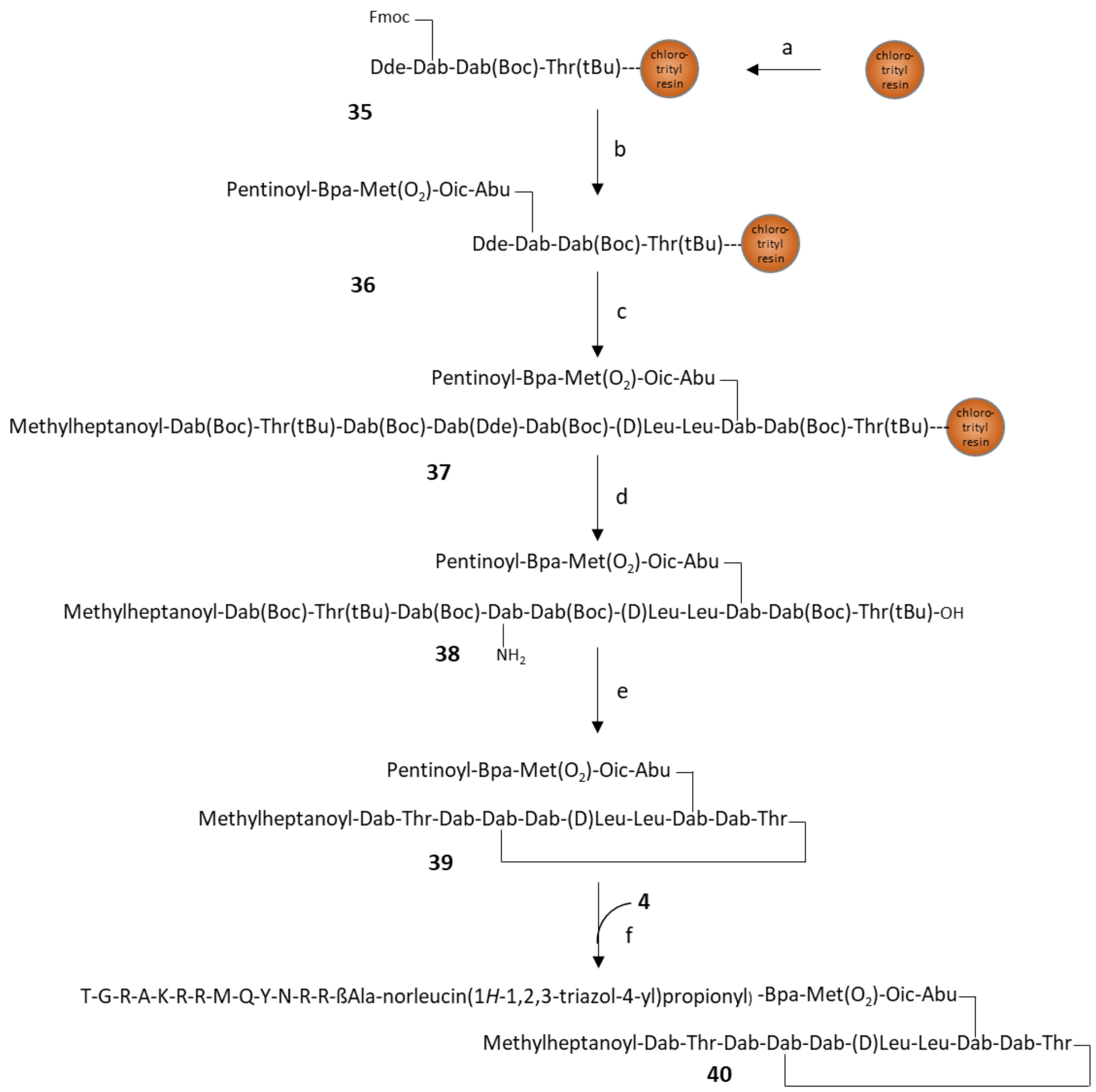

Figure S3: Total synthesis of 40, modified at position 8. (a) i) Fmoc-Thr(tBu)-OH, DIPEA, DCM, 2 h; ii) piperidine (20%) in DMF, 10 min; iii) appropriate amino acid, each with activation by HCTU and DIPEA, DMF, 1 h and followed by piperidine (20%), DMF, 10 min; (b) i) Fmoc-Abu-OH, Fmoc-Oic-OH and Fmoc-Met(O_2)-OH, each with activation by HCTU and DIPEA, DMF, 1 h, and followed by piperidine (20%) in DMF, 10 min; ii) Fmoc-Bpa-OH, HATU, DIPEA, DMF, 1 h as double coupling, followed by piperidine (20%), DMF, 10 min, iii) 4-pentinoic acid, HCTU, DIPEA, DMF, 1 h; (c) i) hydrazine (20 mM) in DMF, 3 × 90 min; ii) appropriate amino acid and 6-methyl heptanoic aicd, each with activation by HCTU and DIPEA, DMF, 1 h and followed by piperidine (20%), DMF, 10 min; (d) i) hydrazine (20 mM) in DMF, 3 × 90 min; ii) 1,1,1,3,3,3-hexafluoro-2-propanol (HFIP) / DCM 1:4; (e) i) DIC, HOBt, DCM/DMF 49:1, 5 h; ii) TFA (82.5%), H_2O (5%), phenol (5%), thioanisol (5%), dithiothreitol (2.5%), 90 min, followed by RP-HPLC purification; (f) **4** (1 equiv.), THPTA (200 µM) Na-ascorbate (2 mM), $CuSO_4$ (100 µM), pH 7.0, 2 h, followed by RP-HPLC purification.

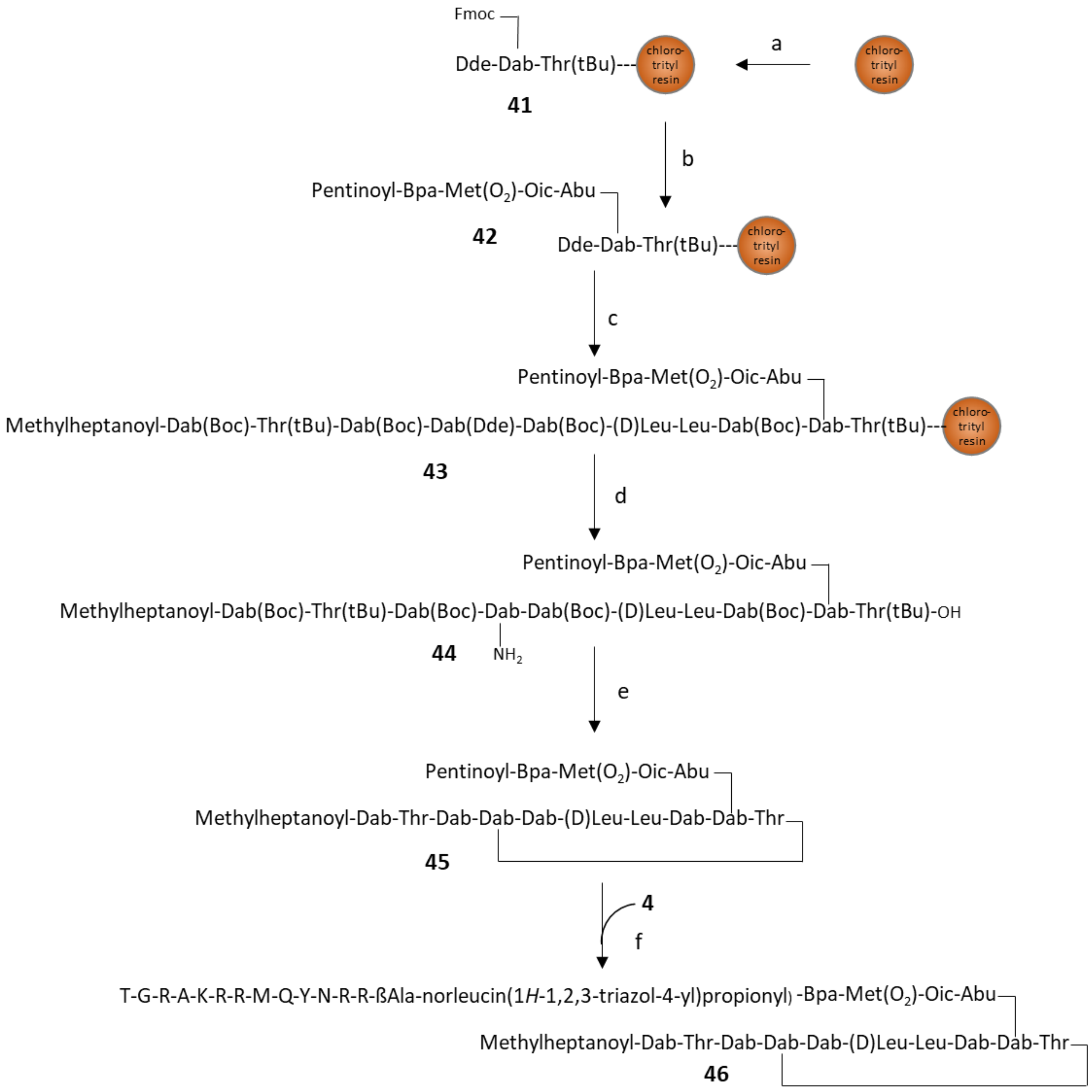

Figure S4: Total synthesis of 46, modified at position 9. (a) i) Fmoc-Thr(tBu)-OH, DIPEA, DCM, 2 h; ii) piperidine (20%) in DMF, 10 min; iii) Dde-Dab(Fmoc)-OH, activation by HCTU and DIPEA, DMF, 1 h and followed by piperidine (20%), DMF, 10 min; (b) i) Fmoc-Abu-OH, Fmoc-Oic-OH and Fmoc-Met(O_2)-OH, each with activation by HCTU and DIPEA, DMF, 1 h, and followed by piperidine (20%) in DMF, 10 min; ii) Fmoc-Bpa-OH, HATU, DIPEA, DMF, 1 h as double coupling, followed by piperidine (20%), DMF, 10 min, iii) 4-pentinoic acid, HCTU, DIPEA, DMF, 1 h; (c) i) hydrazine (20 mM) in DMF, 3 × 90 min; ii) appropriate amino acid and 6-methyl heptanoic aicd, each with activation by HCTU and DIPEA, DMF, 1 h and followed by piperidine (20%), DMF, 10 min; (d) i) hydrazine (20 mM) in DMF, 3 × 90 min; ii) 1,1,1,3,3,3-hexafluoro-2-propanol (HFIP) / DCM 1:4; (e) i) DIC, HOBt, DCM/DMF 49:1, 5 h; ii) TFA (82.5%), H_2O (5%), phenol (5%), thioanisol (5%), dithiothreitol (2.5%), 90 min, followed by RP-HPLC purification; (f) **4** (1 equiv.), THPTA (200 µM) Na-ascorbate (2 mM), $CuSO_4$ (100 µM), pH 7.0, 2 h, followed by RP-HPLC purification.

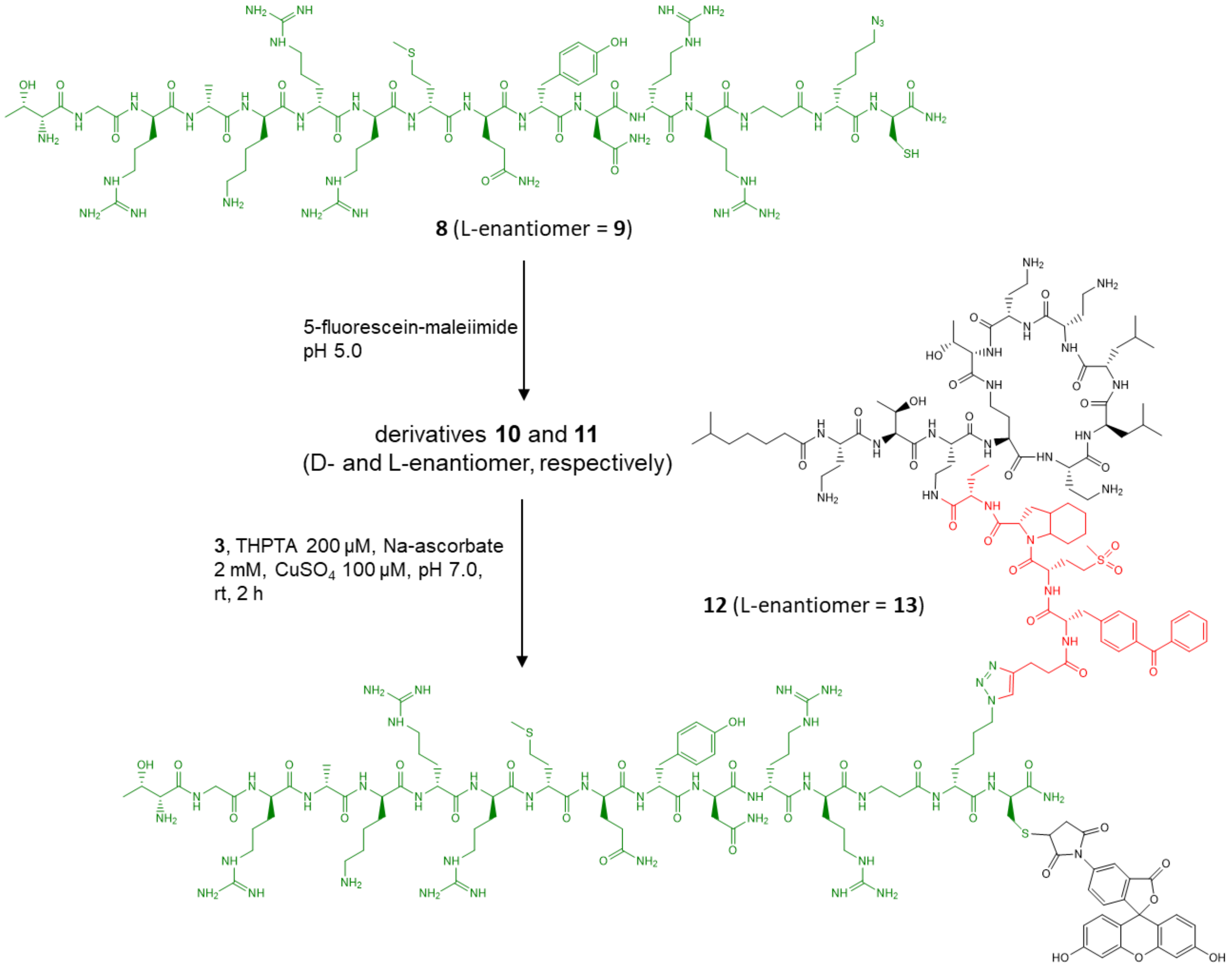

Figure S5. Semisynthesis of 12 and 13. In green: Bacterial binder D-ubiquicidin$_{29\text{-}41}$-ßAla-lys(N_3)-Cys; in red: NE-cleavable linker; in black: colistin B. In structures **9**, **11** and **13** (not shown) the ubiquicidin$_{29\text{-}41}$ derivative is made entirely of L-amino acids

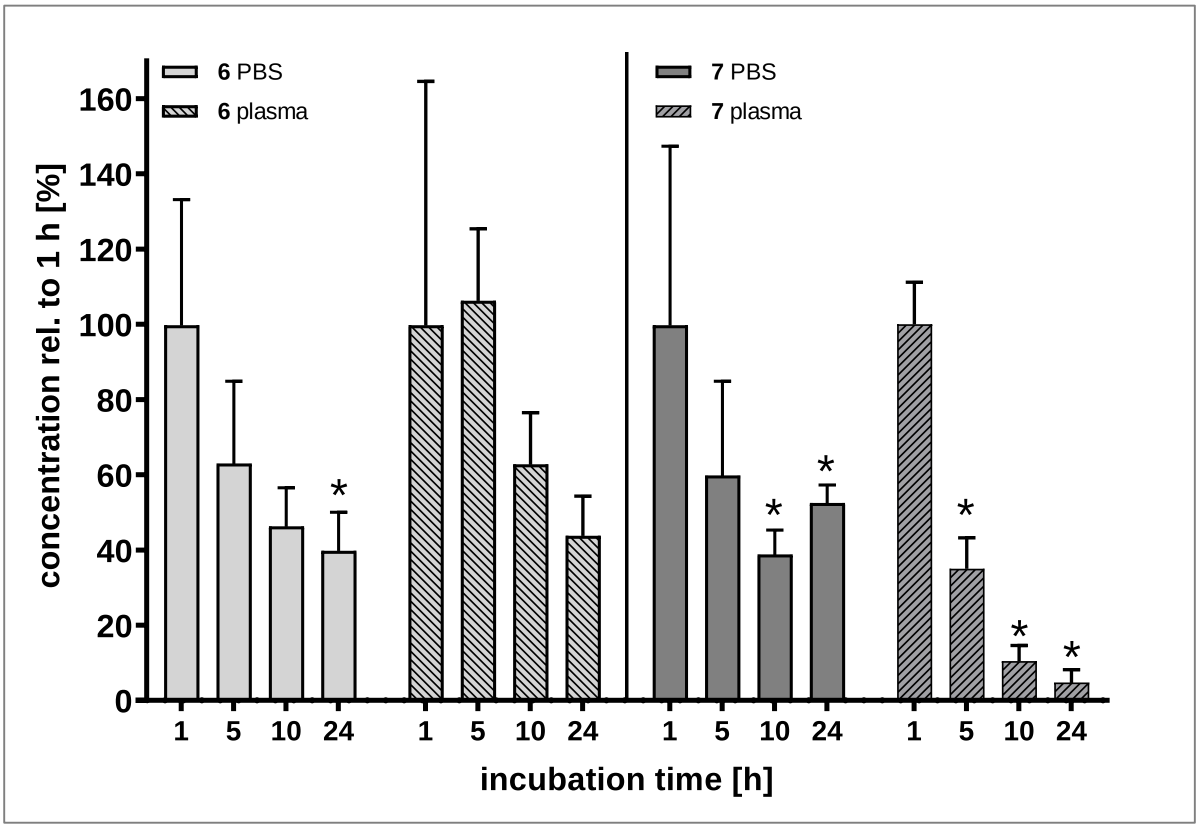

Figure S6. Plasma stabilities of 6 and 7. Compounds **6** (carrying an all-D analog of Ubi29-41) and **7** (carrying an all-L analog of Ubi29-41) were incubated in PBS or human plasma at 10 µM and 37 °C for the times indicated. The remaining quantity of compounds was quantified by LC-MS. Bars represent the averaged peak areas relative to the first time point obtained by LC-MS of four individual samples, and whiskers represent s.d.. * Statistically significant as compared to value at 1 h ($p < 0.05$) according to a Mann-Whitney test.

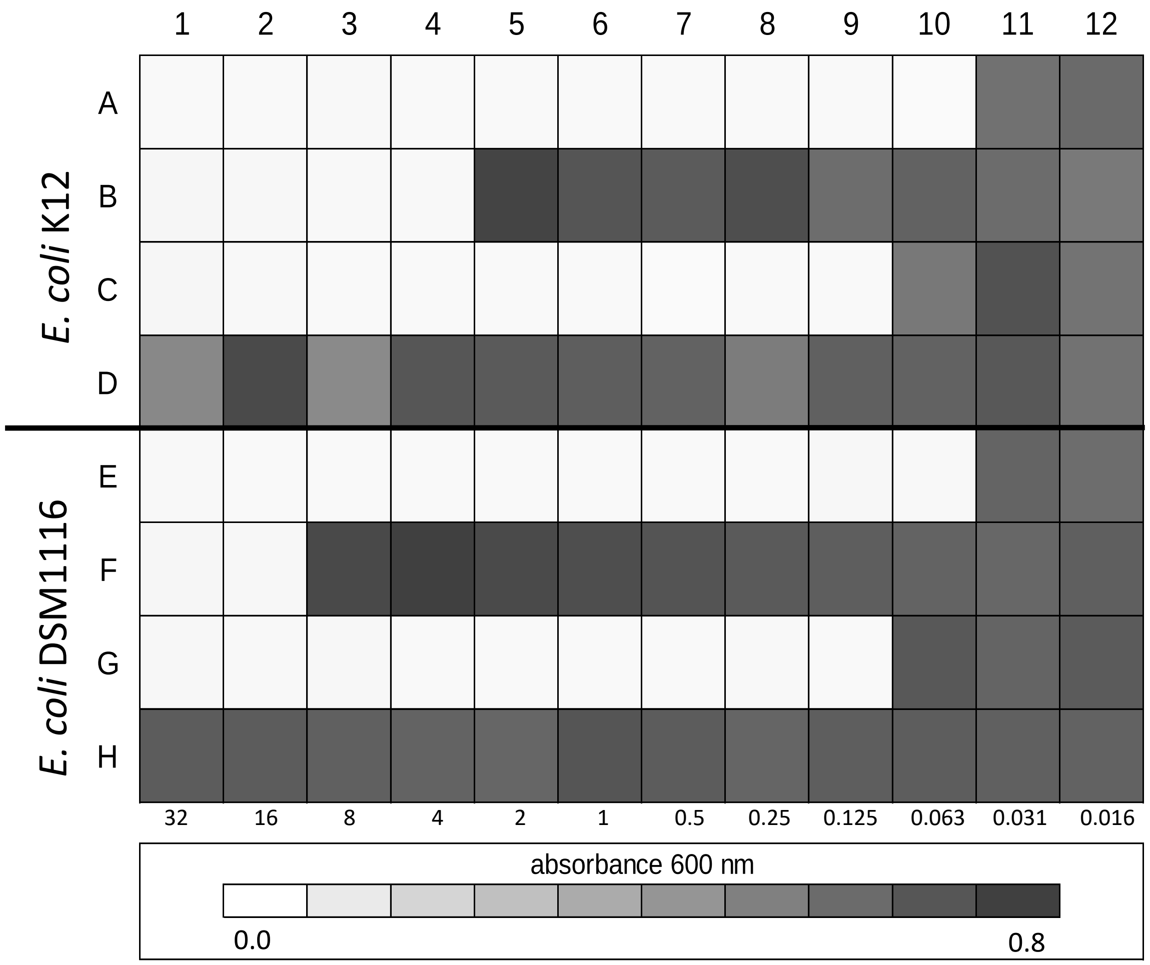

Figure S7. Heat plot of the growth of *E. coli* K12 and *E. coli* DSM 1116 in presence of 6. Rows A-D: *E. coli* K12; Rows E-H: *E. coli* DSM 1116; A + E colistin; B + F peptide-colistin construct **6**; C + G peptide-colistin construct **6** + 10 mU/ml elastase; D + H bacteria without compounds. The numbers below the heatplot depict the concentration of colistin and peptides in µM. Conditions: T medium, incubation 20 h at 37 °C in wet chamber; inoculum: cultures at OD_{600} = 1.0 diluted 1:1000 (5-6 x 10^5 CFU/ml).

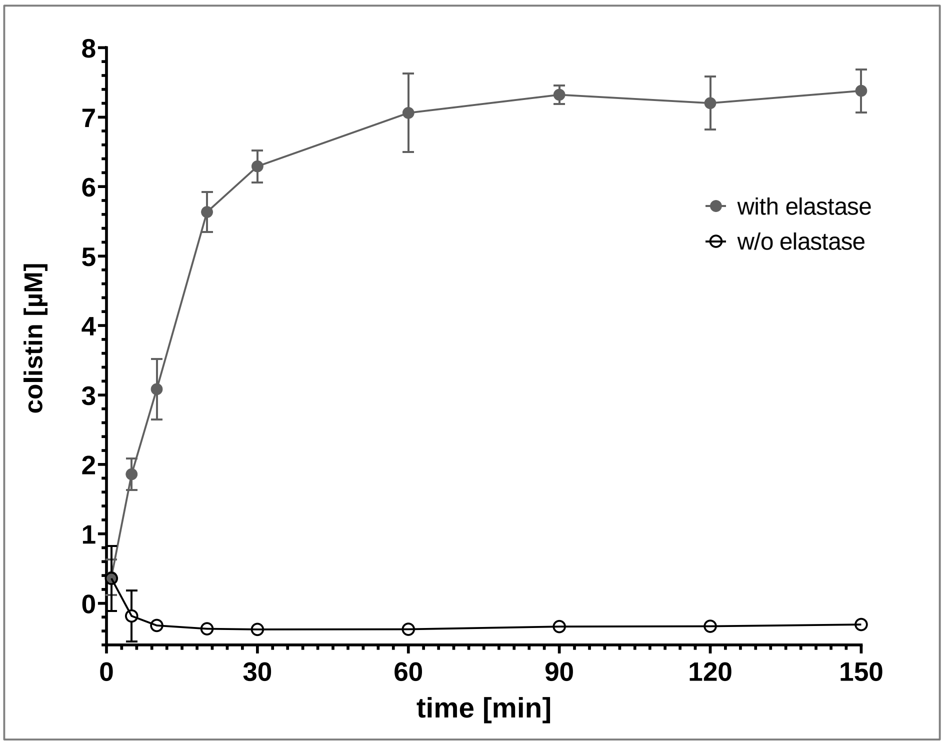

Figure S8. Cleavage of 6 by neutrophil elastase. 10 µM **6** was incubated in RPMI 1640 with and w/o 10 mU/ml neutrophil elastase at 37 °C with shaking. At different time intervals from 1 – 150 min 50 µl samples were taken in triplicate, the reaction was quenched by the addition of 50% acetic acid, and the samples were filtered through 10 kDa ultrafiltration columns. The columns were washed with 300 µl 50% acetic acid and with 300 µl 50% 2-propanol. The solvents of combined filtrates were evaporated, and the residue was redissolved in 50 µl of 50% ACN. Colistin was quantified on a Q-Trap 6500 HPLC-MS with samples of 5 µl each, also in triplicate.

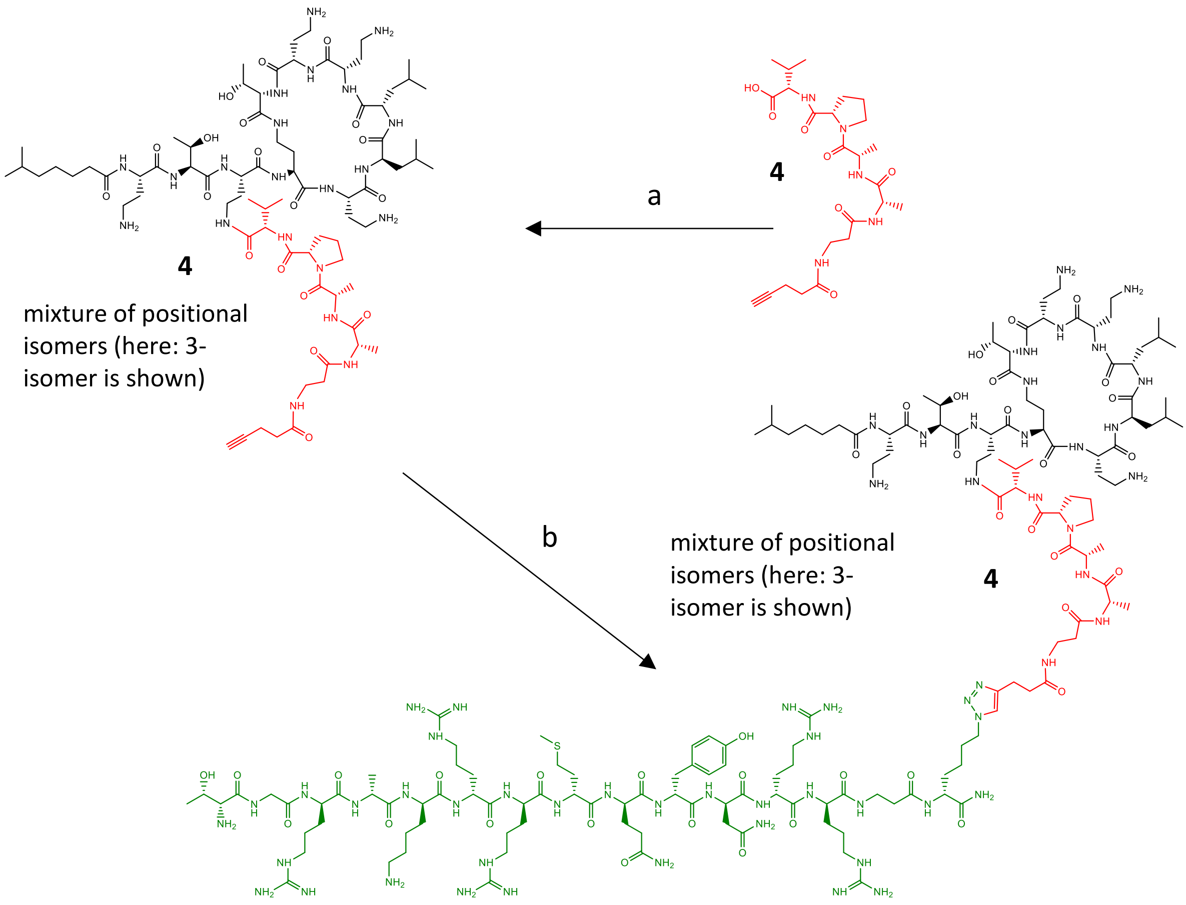

Figure S9. Semisynthesis of 49. (a) **1** (0.3 eq.), 2-propanol / 200 mM MES 1:1, pH 5.0, HOBt / EDC, rt, 18 h; (b) all-D-(T-G-R-A-K-R-R-M-Q-Y-N-R-R-ßA-K(N3)) (**4**) THPTA 200 µM, Na-ascorbate 2 mM, $CuSO_4$ 100 µM, pH 7.0, rt, 2 h; in green: bacterial binder D-ubiquicidin$_{29\text{-}41}$-ßAla-lys(N_3); in red: NE-cleavable linker; in black: colistin B.

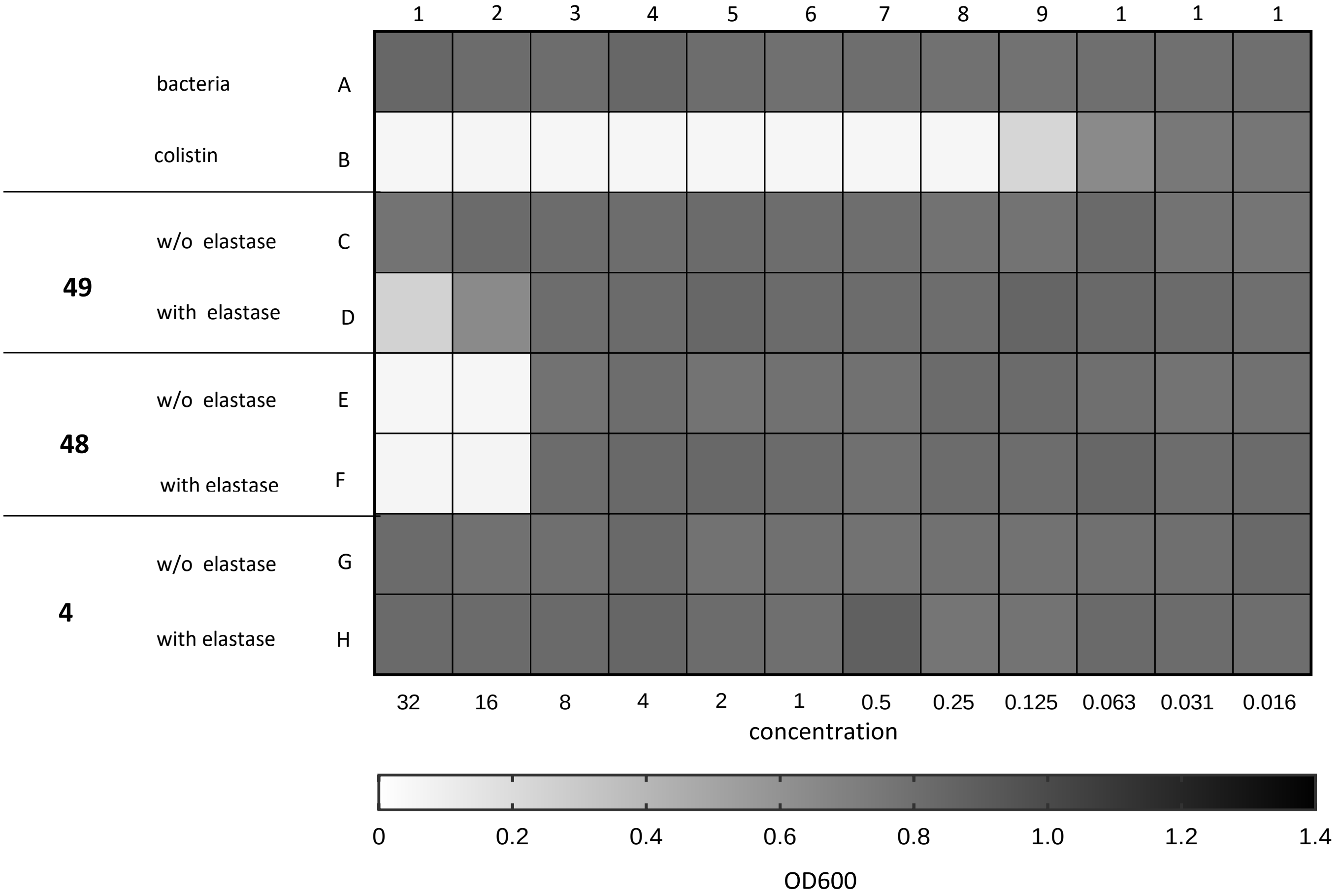

Figure S10. Heat plot of the growth of *E. coli* in presence of 48 and 49. Row A: *E. coli* K12 without added compounds; B colistin B; C peptide-colistin construct **49**; D peptide-colistin construct **49** + 10 mU/ml elastase; E ElsaSub-colistin; F **48** with 10 mU elastase; G **4**; H **4** with 10 mM elastase. The numbers depict the concentration of colistin and peptides in µM. Conditions: T medium, incubation 17.5 h at 37 °C in wet chamber; inoculum 5-6 x 10^5 CFU/ml.

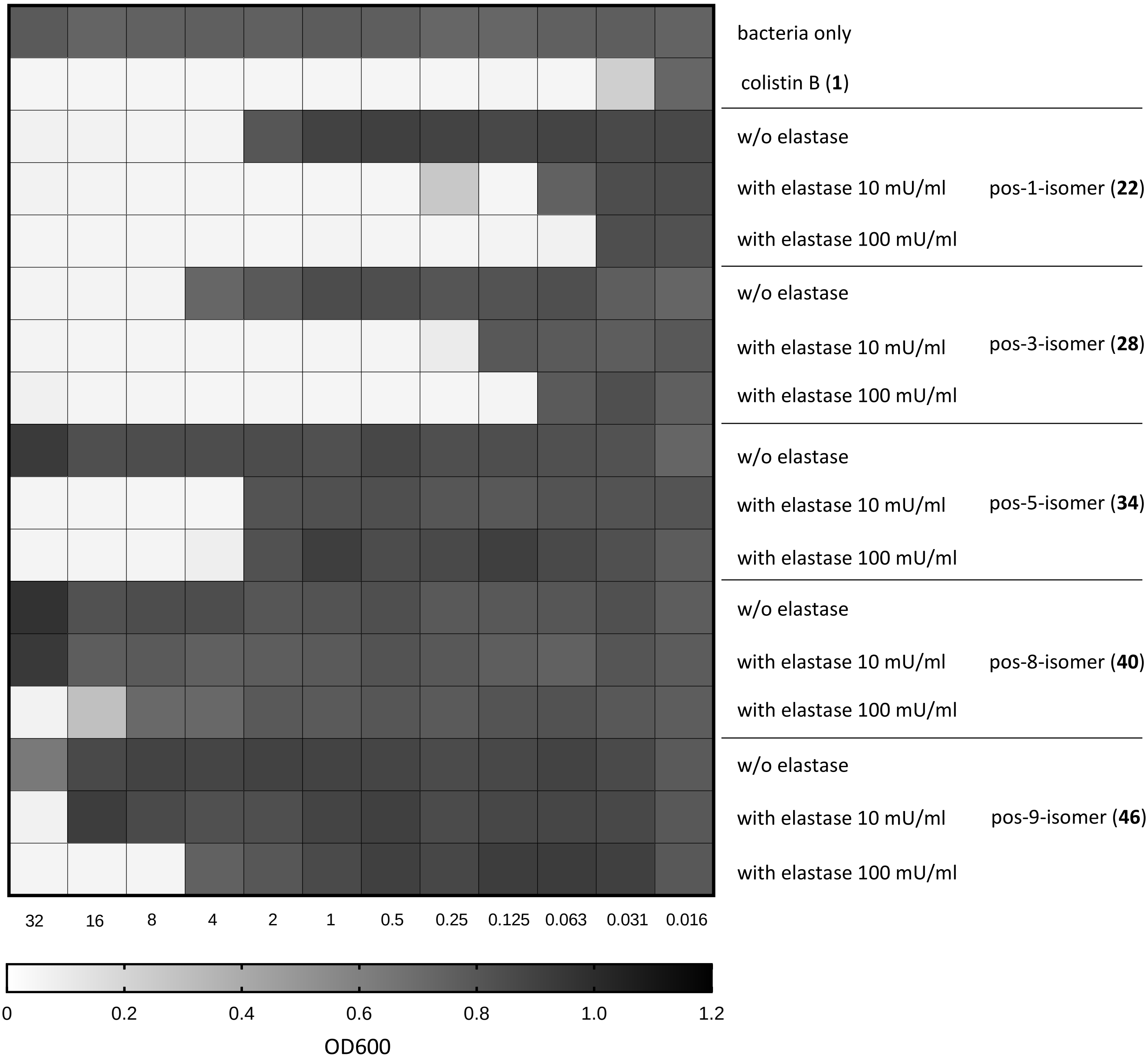

Figure S11. Heat plot of the growth of *E. coli* in presence of positional isomers obtained by total chemical synthesis. Conditions: T medium, incubation for 20 h at 37 °C in wet chamber; inoculum 5-6 x 10^5 CFU/ml. Addition of neutrophil elastase at 10 and 100 mU/ml, as indicated. Concentrations of isomers stated below the heat plot in µg/ml.

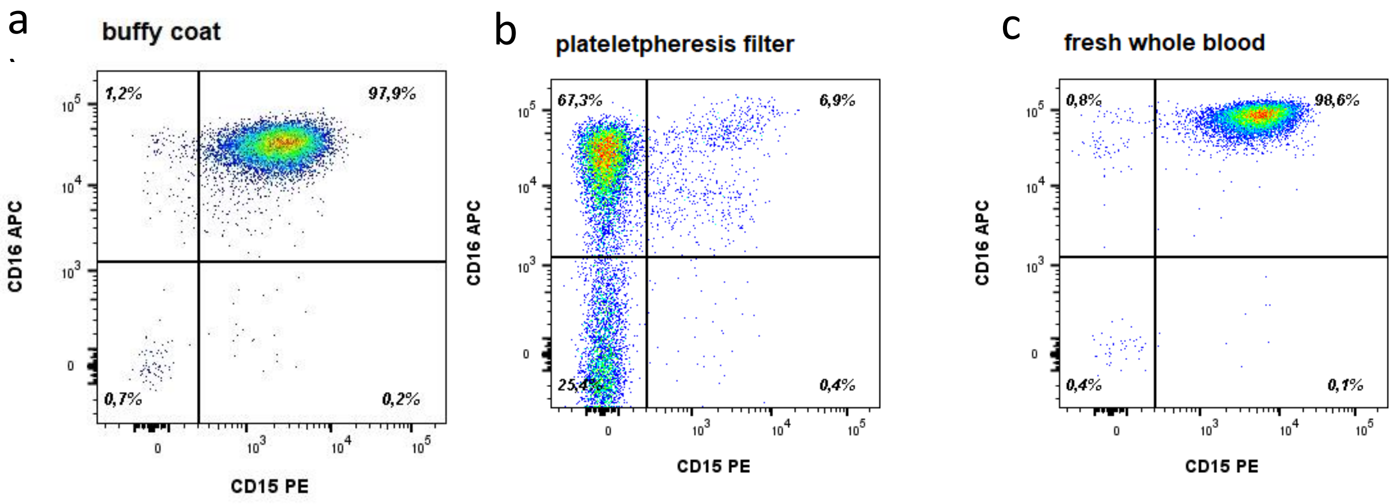

Figure S12. Purity of neutrophil granulocytes after isolation from different sources. Cells were isolated as described in Chapter 4 (Biological Experiments). For the determination of neutrophil granulocytes in a) buffy coat, b) a plateletpheresis filter and c) fresh whole blood, cells were stained with anti-CD15-PE and anti-CD16-APC and analysed by flow cytometry. Anti-CD15 was used to distinguish between granulocytes and other cells. Anti-CD15^{+}/anti-CD16^{+} are markers specific for neutrophil granulocytes.

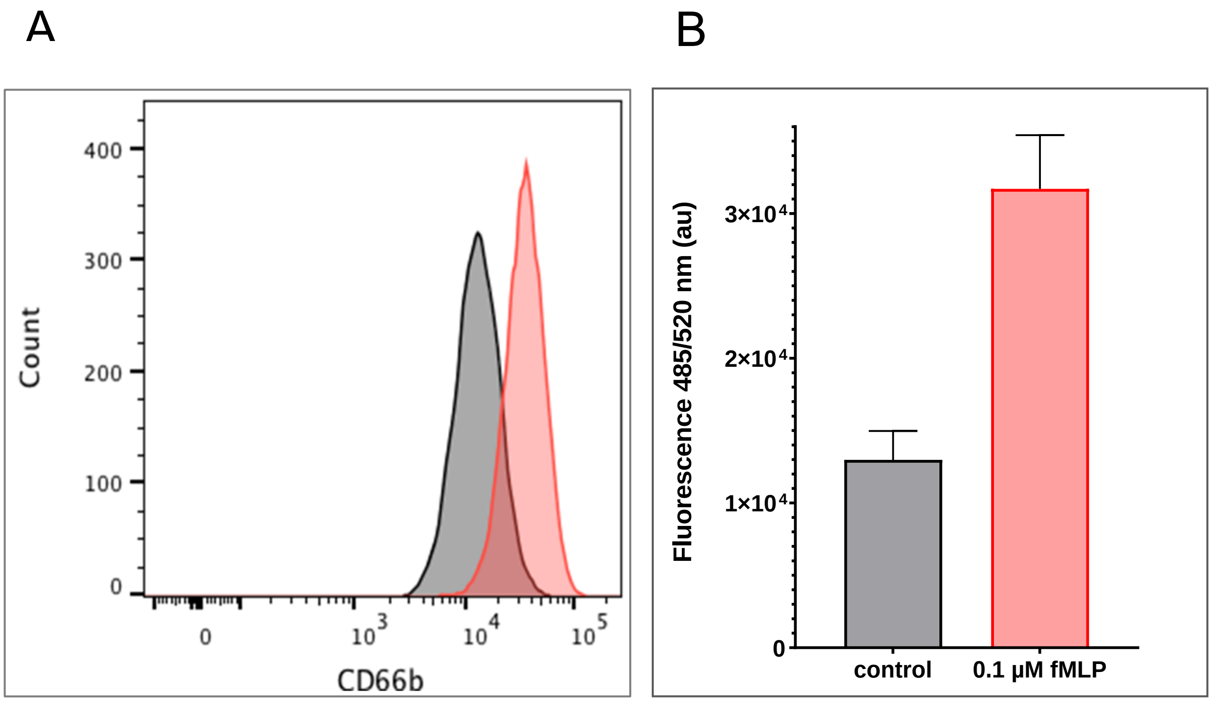

Figure S13. Activation of human neutrophil granulocytes with fMLP. A CD66b expression of fMLP-stimulated PMN in comparison to control as determined by incubation of granulocytes with FITC-labeled anti CD66b antibody. Grey: control; red: 0.1 µM fMLP. The samples were incubated for 1 h at 37 °C with shaking. **B** Mean fluorescence and standard deviation from three independent experiments according to **A**.

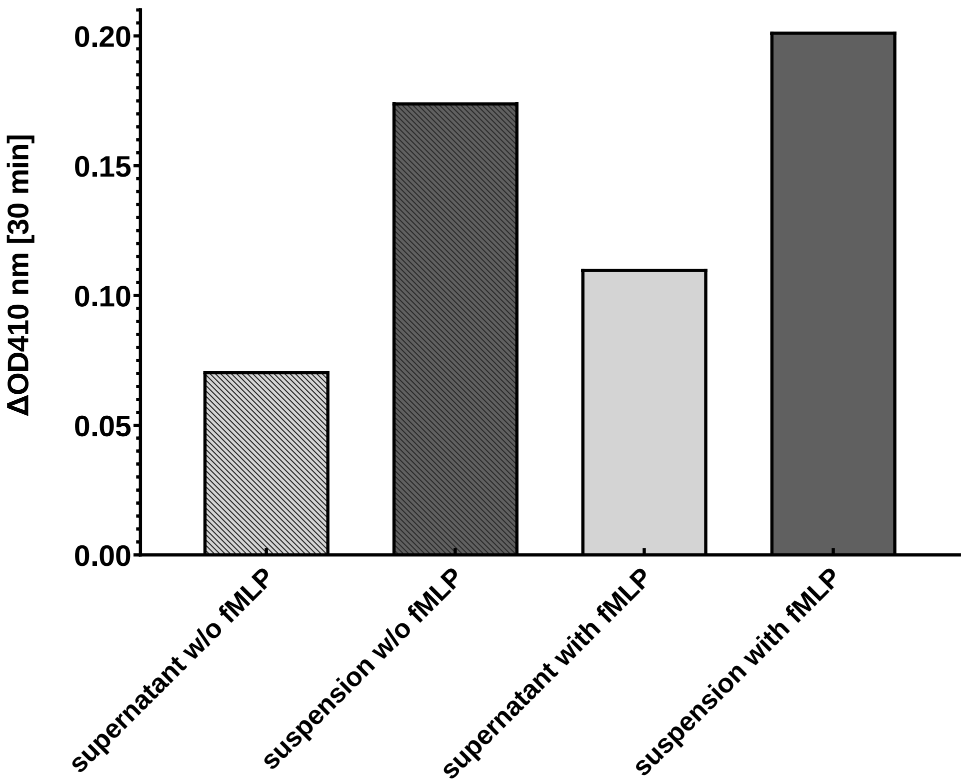

Figure S14. Activity of secreted NE as determined by the release of p-nitroanilin upon cleavage of the substrate peptide MeO-SucAAPV-pNA. PMN were isolated and suspended in RPMI 1640. For activation, 0.1 µM fMLP was added as indicated. For tests with supernatants cells were removed by centrifugation. MeOSuc-AAPV-pNA was used at 100 µM in HEPES-NaCl. After an initiation phase of 15 min at 37 °C the difference in OD410 was determined over a further 30 min incubation and the blanc was subtracted. Results were normalized to $1x10^6$ cells/ml. Orienting experiment with one sample per data point.

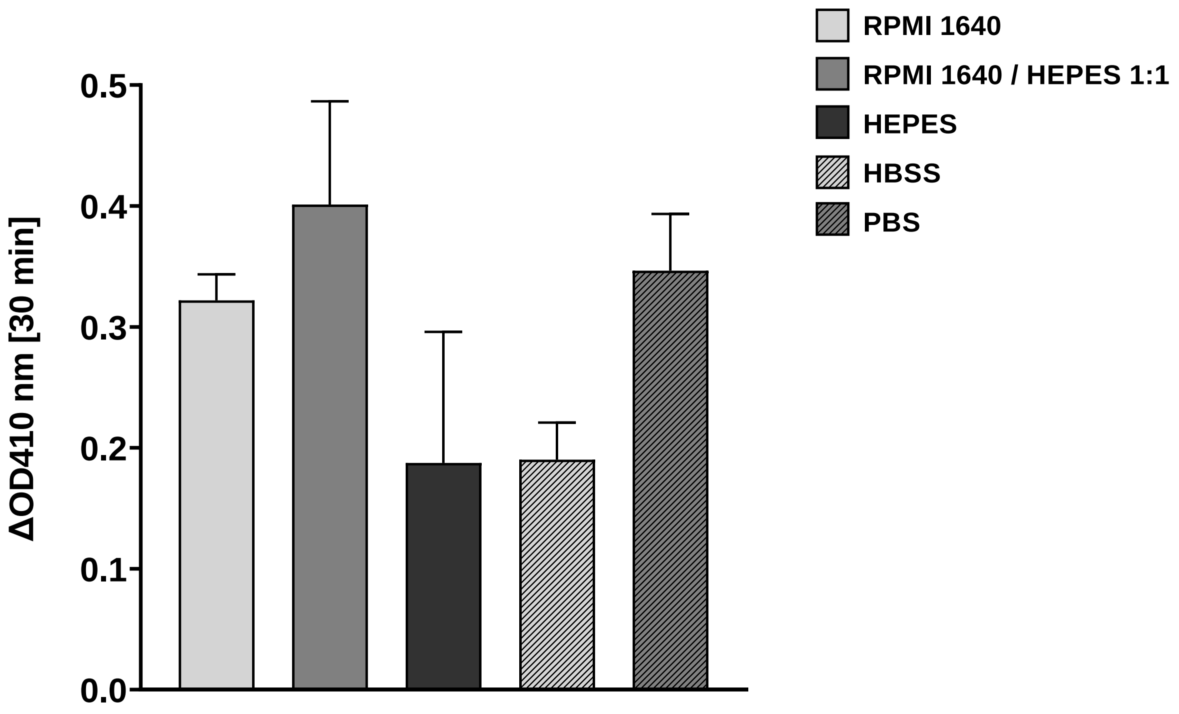

Figure S15. Activity of neutrophil elastaste in different media as determined by the release of p-nitroanilin upon cleavage of the substrate peptide MeO-SucAAPV-pNA. MeOSuc-AAPV-pNA was used at 100 µM in presence of 10 mU/ml externally added neutrophil elastase. After an initiation phase of 15 min at 37 °C, the difference in OD410 was determined over a further 30 min incubation, and the blanc was subtracted. Data presented as the mean of two separate experiments.

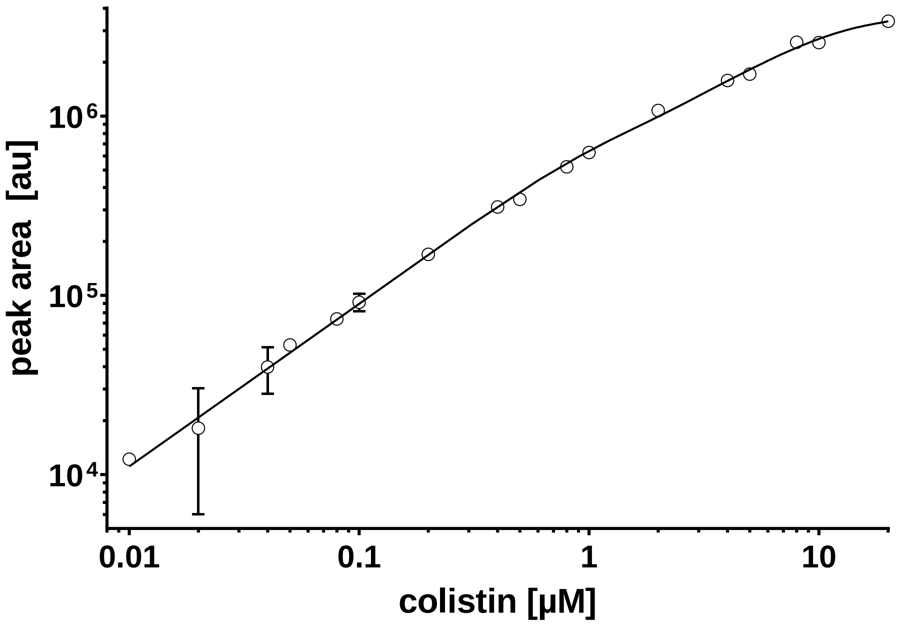

Figure S16. Standard curve for the quantification of colistin B by LC-MS/MS.

5 µl injections, peak at 1.8 min retention time, detected ion $[289.7]^{4+}$.

6 Compound synthesis

(Semi-)Synthesis of mixed positional peptide-colistin isomers

Pentinoyl-Bpa-Met(O$_2$)-Oic-Abu-OH (2)

To 506 mg (200 µmol, capacity 396 µmol/g) chlorotrityl resin with preloaded Fmoc-2-Abu-OH (see Peptide Synthesis General) the amino acids Fmoc-Oic-OH, Fmoc-Met(O$_2$)-OH, Fmoc-Bpa-OH and 4-pentinoic acid were coupled manually in fivefold excess. Fmoc-Bpa-OH, which displayed sluggish reaction, was coupled with HATU/ diisopropylethyl amine as double coupling. After cleavage from the support with HFIP/DCM 1:4, and lyophilization from 1,4-dioxane the product was purified by prep. HPLC with a gradient from 5 to 60% acetonitrile with elution at 53%. Yield: 25.0 mg (33.4 µmol, 16.7%); *m/z* calculated for $C_{39}H_{49}N_4O_9S$ $[M+H]^+$: 749.3 Da; found: 749.4 Da, *m/z* calculated for $C_{39}H_{48}N_4NaO_9S$ $[M+Na]^+$: 771.3 Da; found: 771.4 Da.

Pentinoyl-ßAla-Ala-Ala-Pro-Val-OH (47)

To 652 mg (150 µmol, capacity 230 µmol/g) TentaGel S PHB resin with preloaded Fmoc-Val-OH (see Peptide Synthesis General) the amino acids Fmoc-Pro-OH, Fmoc-Ala-OH (two times), Fmoc-ßAla-OH and 4-pentinoic acid were coupled manually in fivefold excess. After cleavage from the support with 95% TFA, 3% triisopropylsilane, 2% H_2O the crude peptide was purified by prep. HPLC with a gradient from 5 to 40% acetonitrile with elution at 31%. Yield: 48.0 mg (94.7 µmol, 63%); *m/z* calculated for $C_{24}H_{38}N_5O_7$ $[M+H]^+$: 508.3 Da; found: 508.4 Da; *m/z* calculated for $C_{24}H_{37}N_5NaO_7$ $[M+Na]^+$: 530.3 Da; found: 530.3 Da.

Pentinoyl-Bpa-Met(O_2)-Oic-Abu-colistin B (3)

13.1 mg (17.5 µmol) **2** and 70.5 mg (52.5 µmol) colistin B (**1**, HCl-salt) were dissolved in 5 ml 2-propanol / 200 mM MES-buffer 1:1 pH 5.5 and the pH of the solution (which drops upon dissolving the colistin HCl-salt) was adjusted to 5.0 with 1 N NaOH). 3 mg (20 µmol) HOBt and 19.2 mg (100 µmol) EDC x HCl were added and the mixture was stirred at RT for 18 h with occasional control of the progress of the reaction by anal. HPLC. Additional EDC was added if considered necessary, until the HPLC indicated complete consumption of the elastase substrate peptide **2**. At this point, some unreacted colistin and some disubstituted product was present, but the peak group of the monoacylated product dominated. The mixture was separated without previous workup by prep. HPLC. Monoacylated colistin derivative **3** eluted at 48-52% ACN. The fractions were pooled and lyophilized. Yield: 7.40 mg (3.2 µmol (with 4 x TFA), 18.3% based on **2**); *m/z* calculated for $C_{91}H_{147}N_{20}O_{21}S$ $[M+3H]^{3+}$: 629.3585 Da; found: 629.3584 Da; *m/z* calculated for $C_{91}H_{148}N_{20}O_{21}S$ $[M+4H]^{4+}$: 472.2707 Da; found: 472.2708 Da.

Pentinoyl-ßAla-Ala-Ala-Pro-Val-colistin B (48)

5.8 mg (11.4 µmol) **47** and 67.6 mg (39.2 µmol) colistin B (**1**, TFA-salt) were dissolved in 6.7 ml 2-propanol / 200 mM MES-buffer 1:1 pH 5.5. 4.1 mg (26.8 µmol) HOBt and 26.7 mg (139 µmol) EDC x HCl were added and the mixture was stirred at RT for 18 h with occasional control of the progress of the reaction by anal. HPLC. The mixture was separated without previous workup by prep. HPLC. Monoacylated colistin derivative **48** eluted at 41-46% ACN. The fractions were pooled and lyophilized. Yield: 16.0 mg (7.6 µmol, with 4 x TFA, 67% based on **47**); *m/z* calculated for $C_{76}H_{134}N_{21}O_{19}$ $[M+H]^{+}$: 1645.0 Da; found: 1645.0 Da; *m/z* calculated for $C_{76}H_{133}N_{21}NaO_{19}$ $[M+Na]^{+}$: 1667.0 Da; found: 1667.0 Da.

D- and L-ubiquicidin$_{29-41}$ derivatives T-G-R-A-K-R-R-M-Q-Y-N-R-R-ßAla-Lys(N_3) (4 and 5, respectively)

The peptides were assembled on a scale of 150 µmol as C-terminal amides from the D- and L-Fmoc-amino acids, respectively. The crude peptides were purified by preparative HPLC with elution at 32% ACN. Yield **4**: 114,5 mg (42.2 µmol (with 7 x TFA), 28.1%), yield **5**: 87.7 mg (32.2 µmol (with 7 x TFA), 21.5%); *m/z* calculated for $C_{77}H_{139}N_{37}O_{19}S$ $[M+2H]^{2+}$: 959.0 Da; found for **4**: 959.1 Da; found for **5**: 959.5 Da; *m/z* calculated for $C_{77}H_{140}N_{37}O_{19}S$ $[M+3H]^{3+}$: 639.7 Da; found for **4**: 640.0 Da; *m/z* calculated for $C_{77}H_{141}N_{37}O_{19}S$ $[M+4H]^{4+}$: 480.0 Da; found for **4**: 480.3 Da; *m/z* calculated for $C_{77}H_{142}N_{37}O_{19}S$ $[M+5H]^{5+}$: 384.2 Da; found for **4**: 384.5 Da.

(D- and L-) T-G-R-A-K-R-R-M-Q-Y-N-R-R-ßAla-norleucinamide(1*H*-1,2,3-triazol-4-yl)propionyl-Bpa-Met(O_2)-Oic-Abu-colistin (6 and 7, respectively)

The D- and L-ubiquicidin peptide derivatives **4** (5.7 mg, 2.1 µmol) and **5** (10.0 mg, 3.7 µmol) were coupled to **3** (5.0 mg, 2.1 µmol and 8.7 mg, 3.7 µmol, respectively) in a click reaction. General procedure: 1.0 µmol **3** and ubiquicidin derivative **4** or **5** were dissolved in 950 µl 200 mM MOPS buffer pH 7.0 under argon atmosphere. 20 µl of a 10 mM aqueous stock solution of THPTA (resulting in 200 µM), 20 µl of a fresh aqueous solution of 20 mg/ml sodium ascorbate (resulting in 2 mM) and 10 µl of 10 mM aqueous $CuSO_4$ (resulting in 100 µM) were added. After 1-2 h progress of the reaction was monitored by analytical HPLC. If required, another 10 µl of the $CuS0_4$ solution was added. After completion of the reaction, the products were purified without workup by prep. HPLC. Elution of the product peaks (mixture of isomers) at 43-46% ACN; yield for **6**: 9.2 mg (1.8 µmol (with 11 x TFA), 86%); yield for **7**: 13.2 mg (2.61 µmol (with 11 x TFA), 71%); *m/z* calculated for $C_{168}H_{286}N_{57}O_{40}S_2$ $[M+5H]^{5+}$: 761.2302 Da; found for **6**: 761.2333 Da; *m/z* calculated for $C_{168}H_{287}N_{57}O_{40}S_2$ $[M+6H]^{6+}$: 634.5264 Da; found for **6**: 634.5205 Da; found for **7**: 634.5271 Da; *m/z* calculated for $C_{168}H_{288}N_{57}O_{40}S_2$ $[M+7H]^{7+}$: 544.0237 Da; found for **6**: 544.0237 Da; found for **7**: 544.0244 Da; *m/z* calculated for $C_{168}H_{289}N_{57}O_{40}S_2$ $[M+8H]^{8+}$: 476.1466 Da; found for **6**: 476.1423 Da; found for **7**: 476.1473 Da.

D- and L-ubiquicidin$_{29-41}$ derivatives T-G-R-A-K-R-R-M-Q-Y-N-R-R-ßAla-Lys(N_3)-Cys (8 and 9, respectively)

The peptides were assembled on a scale of 100 µmol as C-terminal amides from the D- and L-Fmoc-amino acids, respectively. The crude peptides were purified by preparative HPLC with gradients of 10%-50% ACN and elution at 30%. Yield **8**: 54.8 mg (19.5 µmol (with 7 x TFA), 19.5%); yield **9**: 12.3 mg (4.4 µmol (with 7 x TFA), 4.4%); *m/z* calculated for $C_{80}H_{143}N_{38}O_{20}S_2$ $[M+H]^+$: 2020.1 Da; found for **8**: 2020.0 Da; *m/z* calculated for $C_{80}H_{144}N_{38}O_{20}S_2$ $[M+2H]^{2+}$: 1010.5 Da; found for **9**: 1011.1 Da.

D- and L-ubiquicidin$_{29-41}$ derivatives T-G-R-A-K-R-R-M-Q-Y-N-R-R-ßAla-Lys(N_3)-Cys(3-succinimidyl-N-5-fluorescein) (10 and 11, respectively)

Compounds **8** (15.4 mg, 5.5 µmol) or **9** (12.26 mg, 4.35 µmol) were dissolved in 3 ml 100 mM acetate buffer containing 10% (v/v) DMSO, pH 5.0. 5.5 µl or 4.4 µl (corresponding to 5.5 or 4.4 µmol, respectively) from a 100 mM stock solution of fluorescein-5-maleimide in DMSO were added in portions of 1 µl each over a period of 4 h at rt. After anal. HPLC indicated complete conversion to the products (peak eluting at 38% ACN), the mixtures were separated by prep. HPLC without previous workup. The fractions containing the product were lyophilized. Yield **10**: 3.6 mg (1.1 µmol (with 7 x TFA), 20%), yield **11**: 5.1 mg (1.56 µmol (with 7 x TFA), 36%); *m/z* calculated for $C_{104}H_{159}N_{39}O_{27}S_2$ $[M+4H]^{4+}$: 612.5422 Da; found for **10**: 612.5422 Da; found for **11**: 612.5408 Da; *m/z* calculated for $C_{104}H_{159}N_{39}O_{28}S_2$ $[M+O+4H]^{4+}$: 616.5409 Da; found for **10**: 616.5411 Da; *m/z* calculated for $C_{104}H_{160}N_{39}O_{27}S_2$ $[M+5H]^{5+}$: 490.2352 Da; found for **10**: 490.2351 Da; found for **11**: 490.2345 Da; *m/z* calculated for $C_{104}H_{160}N_{39}O_{28}S_2$ $[M+O+5H]^{5+}$: 493.4347 Da; found for **10**: 493.4343 Da; *m/z* calculated for $C_{104}H_{161}N_{39}O_{27}S_2$ $[M+6H]^{6+}$: 408.6972 Da; found for **10**: 408.6972 Da; found for **11**: 408.6967 Da; *m/z* calculated for $C_{104}H_{161}N_{39}O_{28}S_2$ $[M+O+6H]^{6+}$: 411.3630 Da; found for **10**: 411.3630 Da.

(D- and L-) T-G-R-A-K-R-R-M-Q-Y-N-R-R-ßAla-norleucinamide(1*H*-1,2,3-triazol-4-yl)propionyl-(Cys(3-succinimidyl-N-5-fluorescein))-Bpa-Met(O_2)-Oic-Abu-colistin (12 and 13, respectively)

Compound **3** (5 mg, 2.14 µmol and 3 mg, 1.28 µmol, respectively) were reacted with **10** (3.6 mg, 1.11 µmol and **11** (4.6 mg, 1.41 µmol), respectively, according to the general click-reaction procedure described for **6** and **7**. Elution of the products at 45-46% ACN; yield for **12**: 0.98 mg (0.175 µmol, with 11 x TFA), 16% based on **10**, yield for **13**: 4.3 mg (0.77 µmol, with 11 x TFA), 60% based on **3**; *m/z* calculated for $C_{195}H_{305}N_{59}O_{48}S_3$: $[M+6H]^{6+}$: 722.8728 Da; found for **13**: 722.8681 Da; *m/z* calculated for $C_{195}H_{305}N_{59}O_{49}S_3$: $[M+O+6H]^{6+}$: 725.5386 Da; found for **12**: 725.5336 Da; *m/z* calculated for $C_{195}H_{307}N_{59}O_{49}S_3$: $[M+H_2O+6H]^{6+}$: 725.8746 Da; found for **13**: 725.8693 Da; *m/z* calculated for $C_{195}H_{307}N_{59}O_{50}S_3$: $[M+O+H_2O+6H]^{6+}$: 728.5404 Da; found for **12**: 728.5362 Da; found for **13**: 728.5354 Da; *m/z* calculated for $C_{195}H_{306}N_{59}O_{48}S_3$: $[M+7H]^{7+}$: 619.7492 Da; found for **13**: 619.7438 Da; *m/z* calculated for $C_{195}H_{306}N_{59}O_{49}S_3$: $[M+O+7H]^{7+}$: 622.0342 Da; found for **12**: 622.0290 Da; *m/z* calculated for $C_{195}H_{308}N_{59}O_{49}S_3$: $[M+H_2O+7H]^{7+}$: 622.3221 Da; found for **13**: 622.3171 Da; *m/z* calculated for $C_{195}H_{308}N_{59}O_{50}S_3$: $[M+O+H_2O+7H]^{7+}$: 624.6071 Da; found for **12**: 624.6035 Da; found for **13**: 624.6033 Da; *m/z* calculated for $C_{195}H_{307}N_{59}O_{48}S_3$: $[M+8H]^{8+}$: 542.4064 Da; found for **13**: 542.4028 Da; *m/z* calculated for $C_{195}H_{307}N_{59}O_{49}S_3$: $[M+O+8H]^{8+}$: 544.4058 Da; found for **12**: 544.4025 Da; *m/z* calculated for $C_{195}H_{309}N_{59}O_{49}S_3$: $[M+H_2O+8H]^{8+}$: 544.6577 Da; found for **13**: 544.6537 Da; *m/z* calculated for $C_{195}H_{309}N_{59}O_{50}S_3$: $[M+O+H_2O+8H]^{8+}$: 546.6571 Da; found for **12**: 546.6539 Da; found for **13**: 546.6532 Da; *m/z* calculated for $C_{195}H_{308}N_{59}O_{48}S_3$: $[M+9H]^{9+}$: 482.2510 Da; found for **13**: 482.2477 Da; *m/z* calculated for $C_{195}H_{308}N_{59}O_{49}S_3$: $[M+O+9H]^{9+}$: 484.0282 Da; found for **12**: 484.0256 Da; *m/z* calculated for $C_{195}H_{310}N_{59}O_{49}S_3$: $[M+H_2O+9H]^{9+}$: 484.2521 Da; found for **13**: 484.2488 Da; *m/z* calculated for $C_{195}H_{310}N_{59}O_{50}S_3$: $[M+O+H_2O+9H]^{9+}$: 486.0294 Da; found for **12**: 486.0269 Da; found for **13**: 486.0247 Da.

(*all*-D) T-G-R-A-K-R-R-M-Q-Y-N-R-R-ßAla-norleucinamide(1*H*-1,2,3-triazol-4-yl)propionyl-ßAla-L-Ala-L-Ala-L-Pro-L-Val-colistin B (49)

The D-ubiquicidin peptide derivative **4** (7.5 mg, 2.8 µmol) was coupled to 4-pentinoyl-ßAla-Ala-Ala-Pro-Val-colistin B **48** (5.0 mg, 2.4 µmol) in a click reaction according to the general procedure described for **6** and **7**. After completion of the reaction, the product was purified by prep. HPLC. Elution of the product peak (mixture of isomers) at 34-38% ACN; yield: 2.5 mg (0.5 µmol, 21% (with 11 x TFA)); *m/z* calculated for $C_{153}H_{276}N_{58}O_{38}S$ $[M+6H]^{6+}$: 594.3523 Da; found: 594.3547 Da; *m/z* calculated for $C_{153}H_{276}N_{58}O_{39}S$ $[M+O+6H]^{6+}$: 597.0181 Da; found: 597.0212 Da; *m/z* calculated for $C_{153}H_{277}N_{58}O_{38}S$ $[M+7H]^{7+}$: 509.5887 Da; found: 509.5911 Da; *m/z* calculated for $C_{153}H_{277}N_{58}O_{39}S$ $[M+O+7H]^{7+}$: 511.8737 Da; found: 511.8764 Da;*m/z* calculated for $C_{153}H_{278}N_{58}O_{38}S$ $[M+8H]^{8+}$: 446.0160 Da; found: 446.0180 Da; *m/z* calculated for $C_{153}H_{278}N_{58}O_{39}S$ $[M+O+8H]^{8+}$: 448.0154 Da; found: 448.0178 Da.

(D-) T-G-R-A-K-R-R-M-Q-Y-N-R-R-ßAla-norleucinamide(1*H*-1,2,3-triazol-4-yl)propionyl-Bpa-Met(O_2)-Oic-Abu (50)

The D-peptide derivative **4** (10.9 mg, 4.0 µmol) was coupled to **2** (3.0 mg, 4.0 µmol) in a click reaction according to the general procedure described for **6** and **7**. After completion of the reaction, the product was purified by prep. HPLC. Elution of the product peak at 44-46% ACN; *m/z* calculated for $C_{116}H_{187}N_{41}O_{28}S_2$ $[M+2H]^{2+}$: 1333.20 Da; found: 1334.21 Da; *m/z* calculated for $C_{116}H_{188}N_{41}O_{28}S_2$ $[M+3H]^{3+}$: 889.13 Da; found: 889.72 Da; *m/z* calculated for $C_{116}H_{189}N_{41}O_{28}S_2$ $[M+4H]^{4+}$: 667.10 Da; found: 667.52 Da; *m/z* calculated for $C_{116}H_{190}N_{41}O_{28}S_2$ $[M+5H]^{5+}$: 533.88 Da; found: 534.22 Da.

Synthesis of defined isomers

Extracyclic branching at position 1 of colistin

6-Methylheptanoyl-Dab(4-pentinoyl-Bpa-Met(O_2)-Oic-Abu)-Thr(tBu)-Dab(Boc)-OH (16)

Fmoc-Thr(tBu)-OH, Fmoc-Dab(Dde)-OH and 6-methylheptanoic were coupled manually in fivefold excess to 2-chlorotrityl resin that has been preloaded with Fmoc-Dab(Boc)-OH (374 mg resin, 100 µmol, see Peptide Synthesis General) to yield **14**. She side chain Dde group was cleaved on-resin by treatment with hydrazine in DMF (40 µl of a 1 M solution of hydrazine in THF added to 2 ml DMF, 20 mM). The mixture was stirred at RT for 1.5 h. Two repetitions of the hydrazine treatment were carried out, with washing with DMF in between. After another washing with DMF the amino acids Fmoc-2-Abu-OH, Fmoc-Oic-OH, Fmoc-Met(O_2)-OH, Fmoc-Bpa-OH and 4-pentinoic acid were coupled manually in fivefold excess. As in the synthesis of **2**, Fmoc-Bpa-OH was coupled with HATU/ diisopropylethyl amine as double coupling. After final assembly of the intermediate **15**, the resin was washed with DMF and finally DCM and allowed to dry. After cleavage from the support with HFIP/DCM 1:4 and lyophilization from 1,4-dioxane the product **16** was purified by prep. HPLC with gradients from 10% to 75% ACN. Yield: 28.8 mg (assuming 21.6 µmol, 22%). Analytical HPLC: peak eluting at 71% ACN; *m/z* calculated for $C_{68}H_{102}N_9O_{16}S$ $[M+H]^+$: 1332.7 Da; found: 1332.7 Da; *m/z* calculated for $C_{68}H_{101}N_9NaO_{16}S$ $[M+Na]^+$: 1354.7 Da; found: Da 1354.7 Da.

Fmoc-Dab(NH$_2$)-Dab(Boc)-DLeu-Leu-Dab(Boc)-Dab(Boc)-Thr(tBu)-OH (18)

To 126 mg 2-chlorotrityl resin (50 µmol, capacity 397 µmol/g) with pre-coupled Fmoc-Thr(tBu)-OH (see Peptide Synthesis General) were coupled Fmoc-Dab(Boc)-OH (two times), Fmoc-Leu-OH, Fmoc-D-Leu-OH, Fmoc-Dab(Boc) and Fmoc-Dab(Dde)-OH. The N-terminal Fmoc group was not removed. Control cleavage of a small amount of the resin (1 mg) and analysis of the product showed sufficient quality of the intermediate product, which eluted at 84% ACN. *m/z* Calculated for $C_{76}H_{118}N_{11}O_{19}$ $[M+H]^+$: 1488.9 Da; found: 1488.9 Da; *m/z* calculated for $C_{76}H_{119}N_{11}O_{19}$ $[M+2H]^{2+}$: 744.9 Da; found: 744.9 Da.

The γDde group was cleaved on-resin by three consecutive treatments with 3 ml each of a solution of hydroxylamine x HCl and imidazole in NMP (257 and 193 mM, respectively), to which immediately before use DCM was added at a ratio of 5:1 (v/v), with washing with NMP between the cycles.

Afterwards the resin was washed with DMF (3 x 5 ml) and DCM (3 x 5 ml) and allowed to dry. The crude peptide was cleaved from the resin by treatment with HFIP/DCM 1:4, lyophilized from 1,4-dioxane and used in the next step without purification. Yield: 50.83 mg (assumed 38.37 µmol, 77%). HPLC: elution of product peak at 70% ACN; *m/z* calculated for $C_{66}H_{106}N_{11}O_{17}$ $[M+H]^{+}$: 1324.8 Da; found: 1324.8 Da; *m/z* calculated for $C_{66}H_{107}N_{11}O_{17}$ $[M+2H]^{2+}$: 662.9 Da; found: 662.9 Da.

H-cyclo[Dab-Dab(Boc)-DLeu-Leu-Dab(Boc)-Dab(Boc)-Thr(tBu)] (19)

Crude **18** (50.83 mg, 38.37 µmol) was dissolved in 50 ml dry DMF/DCM 1:50 (peptide concentration 767 mM). PyBOP (39.93 mg, 76.74 µmol), HOBt (10.37 mg, 76.74 µmol) and DIPEA (26.73 µL, 153.48 µmol) were sequentially added under inert atmosphere and the reaction was stirred at rt overnight. The DCM was removed by evaporation at <30 °C. 10% Piperidin in DMF (2 mL) was added to the residue and the mixture was stirred for 1 h at rt. Afterwards, the product was isolated without workup by prep. HPLC with a linear gradient of 30-80% ACN with the product eluting at 63%. The fractions with the product were lyophilized and the product **19** was obtained in 61% yield (25.38 mg, 23.41 µmol) as white solid. *m/z* Calculated for $C_{51}H_{94}N_{11}O_{14}$ $[M+H]^{+}$: 1084.7 Da; found: 1084.7 Da; *m/z* calculated for $C_{102}H_{187}N_{22}O_{28}$ $[2M+H]^{+}$: 2168.4 Da; found: 2168.5 Da.

6-Methylheptanoyl-Dab(4-pentinoyl-Bpa-Met(O_2)-Oic-Abu)-Thr(tBu)-Dab(Boc)-cyclo[Dab-Dab(Boc)-DLeu-Leu-Dab(Boc)-Dab(Boc)-Thr(tBu)] (20)

Crude **16** (15 mg, 11.26 µmol) was dissolved in 3 ml anhydrous DMF and PyBOP (8.8 mg, 16.9 µmol), anhydrous HOBt (1.5 mg, 11.3 µmol) and DIPEA (5.9 µL, 33.8 µmol) were sequentially added. To this, fragment **19** (14.4 mg, 12 µmol) was added and the mixture was stirred at 0°C for 2 h. Reaction control by anal. HPLC showed two product peaks with elution at 92 and 96% ACN, respectively, which both showed the expected mass by LC-MS. After conversion, 100 ml 1,4-dioxane were added to the mixture and the solution was lyophilized to yield a light yellow solid that was used in the next step without

purification. *m/z* Calculated for $C_{119}H_{194}N_{20}O_{29}S$ $[M+2H]^{2+}$: 1200.2 Da; found: 1200.5 Da; *m/z* calculated for $C_{119}H_{193}N_{20}NaO_{29}S$ $[M+H+Na]^{2+}$: 1211.2 Da; found: 1211.5 Da; *m/z* calculated for $C_{119}H_{192}N_{20}Na_2O_{29}S$ $[M+2Na]^{2+}$: 1222.2 Da; found: 1222.5 Da.

6-Methylheptanoyl-Dab(4-pentinoyl-Bpa-Met(O$_2$)-Oic-Abu)-Thr-Dab-cyclo[Dab-Dab-DLeu-Leu-Dab-Dab-Thr] (21)

The crude product from the procedure described before was deprotected with reagent KW (see Peptide Synthesis General). Purification by prep. HPLC eluted pure product at 53% ACN and resulted in 3.9 mg **21** (2.07 µmol, 18% based on **16**). *m/z* Calculated for $C_{91}H_{145}N_{20}O_{21}S$ $[M+H]^{+}$: 1886.1 Da; found: 1886.1 Da; *m/z* calculated for $C_{91}H_{146}N_{20}O_{21}S$ $[M+2H]^{2+}$: 943.5 Da; found: 943.7 Da; *m/z* calculated for $C_{91}H_{147}N_{20}O_{21}S$ $[M+3H]^{3+}$: 629.4 Da; found: 629.5 Da; *m/z* calculated for $C_{91}H_{148}N_{20}O_{21}S$ $[M+4H]^{4+}$: 472.3 Da; found: 472.5 Da.

6-Methylheptanoyl-Dab(T-G-R-A-K-R-R-M-Q-Y-N-R-R-ßAla-norleucinamide(1*H*-1,2,3-triazol-4-yl)propionyl-Bpa-Met(O$_2$)-Oic-Abu)-Thr-Dab-cyclo[Dab-Dab-DLeu-Leu-Dab-Dab-Thr] (22)

Compound **21** (4.01 mg, 2.12 µmol and 6.8 mg (2.5 µmol) D-ubiquicidin derivative **4** were coupled by click-reaction according to the procedure described for **6**. The mixture was separated by prep. HPLC without workup and the fractions with the product which eluted at 55% ACN were lyophilized to yield 5.48 mg (1.08 µmol (with 11 x TFA), 51%) **22** as a white solid. *m/z* Calculated for $C_{168}H_{287}N_{57}O_{40}S_2$ $[M+6H]^{6+}$: 634.5264 Da; found: 634.5265 Da; *m/z* calculated for $C_{168}H_{287}N_{57}O_{41}S_2$ $[M+O+6H]^{6+}$: 637.1922 Da; found: 637.1912 Da; *m/z* calculated for $C_{168}H_{288}N_{57}O_{40}S_2$ $[M+7H]^{7+}$: 544.0237 Da; found: 544.0238 Da; *m/z* calculated for $C_{168}H_{288}N_{57}O_{41}S_2$ $[M+O+7H]^{7+}$: 546.3087 Da; found: 546.3083 Da; *m/z* calculated for $C_{168}H_{289}N_{57}O_{40}S_2$ $[M+8H]^{8+}$: 476.1466 Da; found: 476.1469 Da; *m/z* calculated for $C_{168}H_{289}N_{57}O_{41}S_2$ $[M+O+8H]^{8+}$: 478.1460 Da; found: 478.1458 Da.

Extracyclic branching at position 3 of colistin

6-Methylheptanoyl-Dab(Boc)-Thr(tBu)-Dab(4-pentinoyl-Bpa-Met(O_2)-Oic-Abu)-OH (25)

Fmoc-Thr(tBu)-OH, Fmoc-Dab(Boc)-OH and 6-methylheptanoic were coupled manually in fivefold excess to 2-chlorotrityl resin that has been preloaded with Fmoc-Dab(Dde)-OH (181 mg resin, 50 µmol, capacity 277 µmol/g, see Peptide Synthesis General) to yield **23**. Afterwards, the side chain Dde group was cleaved on-resin and the elastase substrate peptide was assembled as stated in the synthesis of **15**. The product was cleaved from the support with HFIP/DCM 1:4 and crude **25** was obtained after lyophilization from 1,4-dioxane and used in the next step without purification. Analytical HPLC: peak eluted at 72% ACN; *m/z* calculated for $C_{68}H_{102}N_9O_{16}S$ $[M+H]^+$: 1332.7 Da; found: 1332.7 Da; *m/z* calculated for $C_{68}H_{101}N_9NaO_{16}S$ $[M+Na]^+$: 1354.7 Da; found: 1354.7 Da.

6-Methylheptanoyl-Dab(Boc)-Thr(tBu)-Dab(4-pentinoyl-Bpa-Met(O_2)-Oic-Abu)-cyclo[Dab-Dab(Boc)-DLeu-Leu-Dab(Boc)-Dab(Boc)-Thr(tBu)] (26)

Crude **25** (13.10 mg, 9.83 µmol) was dissolved in 3 ml anhydrous DMF and PyBOP (7.7 mg, 14.8 µmol), anhydrous HOBt (1.3 mg, 9.8 µmol) and DIPEA (5.1 µL, 29.5 µmol) were sequentially added. To this, fragment **19** (11.8 mg, 9.83 µmol) was added and the mixture was stirred at 0°C for 2 h. After addition of 100 ml 1,4-dioxane the solution was lyophilized to yield a light yellow solid that was used in the next step without purification. Yield: 21.22 mg (assuming 8.85 µmol). Anal. HPLC: Elution of **26** at 92% ACN. *m/z* Calculated for $C_{119}H_{194}N_{20}O_{29}S$ $[M+2H]^{2+}$: 1199.7 Da; found: 1200.5 Da; *m/z* calculated for $C_{119}H_{193}N_{20}NaO_{29}S$ $[M+H+Na]^{2+}$: 1210.7 Da; found: 1211.5 Da; *m/z* calculated for $C_{119}H_{192}N_{20}Na_2O_{29}S$ $[M+2Na]^{2+}$: 1221.7 Da; found: 1222.5 Da.

6-Methylheptanoyl-Dab-Thr-Dab(4-pentinoyl-Bpa-Met(O_2)-Oic-Abu)-cyclo[Dab-Dab-DLeu-Leu-Dab-Dab-Thr] (27)

Crude **26** (21.22 mg, assuming 8.85 µmol) was deprotected with reagent KW (see Peptide Synthesis General). Purification by prep. HPLC eluted pure **27** at 50% ACN and resulted in 4.0 mg (1.7 µmol (with 4 x TFA), 19% based on **26**). *m/z* Calculated for $C_{91}H_{145}N_{20}O_{21}S$ $[M+H]^+$: 1886.1 Da; found: 1886.1 Da; *m/z* calculated for $C_{91}H_{146}N_{20}O_{21}S$ $[M+2H]^{2+}$: 943.5 Da; found: 943.7 Da; *m/z* calculated for $C_{91}H_{147}N_{20}O_{21}S$ $[M+3H]^{3+}$: 629.4 Da; found: 629.5 Da; *m/z* calculated for $C_{91}H_{148}N_{20}O_{21}S$ $[M+4H]^{4+}$: 472.3 Da; found: 472.5 Da.

6-Methylheptanoyl-Dab-Thr-Dab(T-G-R-A-K-R-R-M-Q-Y-N-R-R-ßAla-norleucinamide(1*H*-1,2,3-triazol-4-yl)propionyl-Bpa-Met(O_2)-Oic-Abu)-cyclo[Dab-Dab-DLeu-Leu-Dab-Dab-Thr] (28)

27 (4.0 mg, 2.1 µmol and 6.8 mg (2.5 µmol) **4** were coupled by click-reaction according to the procedure described for **6**. The mixture was separated by prep. HPLC without workup and the fractions with the product which eluted at 43% ACN were lyophilized to yield **28** (5.65 mg (1.12 µmol (with 11 x TFA), 53% based on **27**) as a white solid. *m/z* Calculated for $C_{168}H_{287}N_{57}O_{40}S_2$ $[M+6H]^{6+}$: 634.5264 Da; found: 634.5278 Da; *m/z* calculated for $C_{168}H_{287}N_{57}O_{41}S_2$ $[M+O+6H]^{6+}$: 637.1922 Da; found: 637.1947 Da; *m/z* calculated for $C_{168}H_{288}N_{57}O_{40}S_2$ $[M+7H]^{7+}$: 544.0237 Da; found: 544.0249 Da; *m/z* calculated for $C_{168}H_{288}N_{57}O_{41}S_2$ $[M+O+7H]^{7+}$: 546.3087 Da; found: 546.3107 Da; *m/z* calculated for $C_{168}H_{289}N_{57}O_{40}S_2$ $[M+8H]^{8+}$: 476.1466 Da; found: 476.1477 Da; *m/z* calculated for $C_{168}H_{289}N_{57}O_{41}S_2$ $[M+O+8H]^{8+}$: 478.1460 Da; found: 478.1478 Da.

Intracyclic branching

Intracyclic branching at position 5 of colistin

6-Methylheptanoyl-Dab(Boc)-Thr(tBu)-Dab(Boc)-Dab(NH_2)-Dab(4-pentinoyl-Bpa-Met(O_2)-Oic-Abu)-DLeu-Leu-Dab(Boc)-Dab(Boc)-Thr(tBu)-OH (32)

Dde-Dab(Fmoc)-DLeu-Leu-Dab(Boc)-Dab(Boc)-Thr(tBu)-resin (**29**) was assembled manually on a scale of 50 µmole on Fmoc-Thr(tBu) 2-chlorotrityl resin. A fivefold excess of the Fmoc amino acids was used. After a control cleavage of a small amount of the solid support revealed sufficient quality of the intermediate structure **29**, the Fmoc group was removed and the elastase substrate sequence was assembled manually to yield Dde-Dab(4-pentinoyl-Bpa-Met(O_2)-Oic-Abu)-DLeu-Leu-Dab(Boc)-Dab(Boc)-Thr(tBu)-resin (**30**). As in the case of the synthesis of the elastase substrate **2**, for Fmoc-Bpa-OH a double coupling with HATU activation was carried out. The αDde group from **30** was cleaved on-resin with hydrazine. Test cleavage of a small amount of the peptide from the resin with HFIP/DCM 1:4 and analysis by HPLC (peak eluted at 84% ACN) and LC-MS revealed complete removal of the Dde group. *m/z* Calculated for $C_{81}H_{126}N_{13}O_{20}S$ $[M+H]^+$: 1632.9 Da; found: 1632.8 Da; *m/z* calculated for $C_{81}H_{125}N_{13}NaO_{20}S$ $[M+Na]^+$: 1654.9 Da; found: 1654.8 Da.

Further N-terminal extension of the sequence resulted in resin-bound 6-methylheptanoyl-Dab(Boc)-Thr(tBu)-Dab(Boc)-Dab(Dde)-Dab(4-pentinoyl-Bpa-Met(O_2)-Oic-Abu)-DLeu-Leu-Dab(Boc)-Dab(Boc)-Thr(tBu)-resin (**31**). The side-chain Dde group was removed with hydrazine and the resin was washed with DMF and DCM and allowed to dry. The linear peptide was cleaved from the support with HFIP/DCM 1:4. After lyophilization from 1,4-dioxane the crude product **32** was obtained as a white solid that was used in the next step without purification. Yield 73.4 mg (30.0 µmole, 60%). Analytical HPLC: Major peak with elution at 82% ACN; *m/z* calculated for $C_{119}H_{195}N_{20}O_{30}S$ $[M+H]^+$: 2417.4 Da; found: 2416.4 Da; *m/z* calculated for $C_{119}H_{196}N_{20}O_{30}S$ $[M+2H]^{2+}$: 1209.2 Da; found: 1209.2 Da; *m/z* calculated for $C_{119}H_{195}N_{20}NaO_{30}S$ $[M+H+Na]^{2+}$: 1220.2 Da; found: 1220.2 Da.

6-Methylheptanoyl-Dab-Thr-Dab-cyclo[Dab-Dab(4-pentinoyl-Bpa-Met(O_2)-Oic-Abu)-DLeu-Leu-Dab-Dab-Thr] (33)

Crude **32** (73.4 mg, assuming 30 µmole) was dissolved in dry DMF/DCM 1:49 at 0.5 µmol/mL. PyBOP (23.4 mg, 90 µmol), anhydrous HOBt (12.2 mg, 90 µmol) and DIPEA (31.4 µL, 180 µmol) were added under argon and the reaction was stirred for 18 h at RT. The DCM was removed under vacuum, 40 ml 1,4-dioxane were added and the solution was lyophilized to result in crude **cyclized 32** that was used in the next step without purification. Anal. HPLC showed major peak with elution at 93% ACN.

Crude **cyclized 32** was dissolved in dry DCM (20 mL), TFA (2.5 mL) was added and the mixture was stirred for 90 min at rt. Volatiles were removed under vacuum at <30 °C. Crude peptide **33** was precipitated with cold diethyl ether, dissolved in water and purified by prep. HPLC with elution at 51% ACN and lyophilized. Yield 4.13 mg (1.76 µmol (with 4 x TFA), 5.9% based on crude **32**). *m/z* Calculated for $C_{91}H_{144}N_{20}O_{21}S$ $[M+H]^{+}$: 1886.1 Da; found: 1886.1 Da; *m/z* calculated for $C_{91}H_{145}N_{20}O_{21}S$ $[M+2]^{2+}$: 943.0 Da; found: 943.7 Da; *m/z* calculated for $C_{91}H_{146}N_{20}O_{21}S$ $[M+3H]^{3+}$: 629.0 Da; found: 629.5 Da; *m/z* calculated for $C_{91}H_{147}N_{20}O_{21}S$ $[M+4H]^{4+}$: 472.0 Da; found: 472.5 Da.

6-Methylheptanoyl-Dab-Thr-Dab-cyclo[Dab-Dab(T-G-R-A-K-R-R-M-Q-Y-N-R-R-ßAla-norleucinamide(1*H*-1,2,3-triazol-4-yl)propionyl-Bpa-Met(O_2)-Oic-Abu))-DLeu-Leu-Dab-Dab-Thr] (34)

33 (3.80 mg, 1.6 µmol) and **4** (5.3 mg, 1.95 µmol) were coupled by click reaction as described for the synthesis of **6** (and **7**). Purification by prep. HPLC with elution at 44% ACN. Yield 4.96 mg (0.98 µmol (with 11 x TFA), 61% based on **33**. *m/z* Calculated for $C_{168}H_{287}N_{57}O_{40}S_2$ $[M+6H]^{6+}$: 634.5264 Da; found: 634.5280 Da; *m/z* calculated for $C_{168}H_{287}N_{57}O_{41}S_2$ $[M+O+6H]^{6+}$: 637.1922 Da; found: 637.1935 Da; *m/z* calculated for $C_{168}H_{288}N_{57}O_{40}S_2$ $[M+7H]^{7+}$: 544.0237 Da; found: 544.0251 Da; *m/z* calculated for $C_{168}H_{288}N_{57}O_{41}S_2$ $[M+O+7H]^{7+}$: 546.3087 Da; found: 546.3109 Da; *m/z* calculated for $C_{168}H_{289}N_{57}O_{40}S_2$ $[M+8H]^{8+}$: 476.1466 Da; found: 476.1479 Da; *m/z* calculated for $C_{168}H_{289}N_{57}O_{41}S_2$ $[M+O+8H]^{8+}$: 478.1460 Da; found: 478.1473 Da.

Intracyclic branching at position 8 of colistin

6-Methylheptanoyl-Dab(Boc)-Thr(tBu)-Dab(Boc)-Dab(NH_2)-Dab(Boc)-DLeu-Leu-Dab(4-pentinoyl-Bpa-Met(O_2)-Oic-Abu)-Dab(Boc)-Thr(tBu)-OH (38)

Dde-Dab(Fmoc)-Dab(Boc)-Thr(tBu)-resin (**35**) was assembled manually on a scale of 50 µmole on 2-chlorotrityl resin. The Fmoc group was removed and the elastase substrate sequence was assembled manually to yield Dde-Dab(4-pentinoyl-Bpa-Met(O_2)-Oic-Abu)-Dab(Boc)-Thr(tBu)-resin (**36**). The αDde group from **36** was cleaved on-resin with hydrazine. Test cleavage of a small amount of the peptide from the resin with HFIP/DCM 1:4 and analysis by HPLC (peak eluted at 66% ACN) and LC-MS revealed complete removal of the Dde group. *m/z* Calculated for $C_{60}H_{88}N_9O_{15}S$ $[M+H]^+$: 1206.6 Da; found: 1206.6 Da; *m/z* calculated for $C_{60}H_{87}N_9NaO_{15}S$ $[M+Na]^+$: 1228.6 Da; found: 1228.6 Da.

Further N-terminal extension of the sequence resulted in 6-methylheptanoyl-Dab(Boc)-Thr(tBu)-Dab(Boc)-Dab(Dde)-Dab(Boc)-DLeu-Leu-Dab(4-pentinoyl-Bpa-Met(O_2)-Oic-Abu)-Dab(Boc)-Thr(tBu)-resin (**37**). The side-chain Dde group was removed with hydrazine and the resin was washed with DMF and DCM and allowed to dry. The linear peptide was cleaved from the support with HFIP/DCM 1:4. After lyophilization from 1,4-dioxane crude **38** was obtained as a white solid that was used in the next step without purification. Yield 84.81 mg (assuming 35.1 µmol, 70%). Analytical HPLC: Major peak with elution at 81% ACN; *m/z* calculated for $C_{119}H_{195}N_{20}O_{30}S$ $[M+H]^+$: 2416.4 Da; found: 2416.4 Da; ; *m/z* calculated for $C_{119}H_{196}N_{20}O_{30}S$ $[M+2H]^{2+}$: 1208.7 Da; found: 1209.2; *m/z* calculated for $C_{119}H_{195}N_{20}NaO_{30}S$ $[M+H+Na]^{2+}$: 1219.7 Da; found: 1220.2 Da.

6-Methylheptanoyl-Dab-Thr-Dab-cyclo[Dab-Dab-DLeu-Leu-Dab(4-pentinoyl-Bpa-Met(O_2)-Oic-Abu)-Dab-Thr] (39)

Crude **38** (30 mg, assuming 12.4 µmol) was cyclized following the procedure described for **32** and the product was obtained in 19.66 mg (assuming 8.2 µmol, 66%) crude yield as a light yellow waxy solid that was used in the next step without purification. Anal. HPLC: Major peak with elution at 92% ACN. m/z Calculated for $C_{119}H_{194}N_{20}O_{29}S$ $[M+2H]^{2+}$: 1199.7 Da; found: 1200.5 Da; m/z calculated for

$C_{119}H_{193}N_{20}NaO_{29}S$ [M+H+Na]$^{2+}$: 1210.7 Da; found: 1211.5 Da; m/z calculated for $C_{119}H_{192}N_{20}O_{29}Na_2S$ [M+2Na]$^{2+}$: 1221.7 Da; found: 1222.5 Da.

Deprotection was carried out as described for **cyclized 32** and the product **39** was purified by prep. HPLC with elution at 52% ACN and lyophilized. Yield 7.0 mg (3.0 µmol (with 4 x TFA), 24% based on crude **38**). m/z Calculated for $C_{91}H_{145}N_{20}O_{21}S$ [M+H]$^{+}$: 1886.1 Da; found: 1886.1 Da; m/z calculated for $C_{91}H_{146}N_{20}O_{21}S$ [M+2H]$^{2+}$: 943.5 Da; found: 943.7 Da; m/z calculated for $C_{91}H_{147}N_{20}O_{21}S$ [M+3H]$^{3+}$: 629.4 Da; found: 629.5 Da; m/z calculated for $C_{91}H_{148}N_{20}O_{21}S$ [M+4H]$^{4+}$: 472.3 Da; found: 472.5 Da.

6-Methylheptanoyl-Dab-Thr-Dab-cyclo[Dab-Dab-DLeu-Leu-Dab(T-G-R-A-K-R-R-M-Q-Y-N-R-R-ßAla-norleucinamide(1*H*-1,2,3-triazol-4-yl)propionyl-Bpa-Met(O$_2$)-Oic-Abu)-Dab-Thr] (40)

39 (4.5 mg, 1.9 µmol) and **4** (5.3 mg, 1.95 µmol) were coupled by click reaction as described for the synthesis of **6**. Purification by prep. HPLC with elution at 44% ACN. Yield 6.89 mg (1.36 µmol (with 11 x TFA), 72% based on **39**). *m/z* calculated for $C_{168}H_{287}N_{57}O_{40}S_2$ [M+6H]$^{6+}$: 634.5264 Da; found: 634.5271 Da; *m/z* calculated for $C_{168}H_{287}N_{57}O_{41}S_2$ [M+O+6H]$^{6+}$: 637.1922 Da; found: 637.1945 Da; *m/z* calculated for $C_{168}H_{288}N_{57}O_{40}S_2$ [M+7H]$^{7+}$: 544.0237 Da; found: 544.0232 Da; *m/z* calculated for $C_{168}H_{288}N_{57}O_{41}S_2$ [M+O+7H]$^{7+}$: 546.3087 Da; found: 546.3090 Da; *m/z* calculated for $C_{168}H_{289}N_{57}O_{40}S_2$ [M+8H]$^{8+}$: 476.1466 Da; found: 476.1470 Da; *m/z* calculated for $C_{168}H_{289}N_{57}O_{41}S_2$ [M+O+8H]$^{8+}$: 478.1460 Da; found: 478.1470 Da.

Intracyclic branching at position 9 of colistin

6-Methylheptanoyl-Dab(Boc)-Thr(tBu)-Dab(Boc)-Dab(NH$_2$)-Dab(Boc)-DLeu-Leu-Dab(Boc)-Dab(4-pentinoyl-Bpa-Met(O$_2$)-Oic-Abu)-Thr(tBu)-OH (44)

Dde-Dab(Fmoc)-Thr(tBu)-resin (**41**) was assembled manually on a scale of 50 µmole on 2-chlorotrityl resin. The Fmoc group was removed and the elastase substrate sequence was assembled manually to

yield Dde-Dab(4-pentinoyl-Bpa-Met(O_2)-Oic-Abu)-Thr(tBu)-resin (**42**). The αDde group was cleaved on-resin with hydrazine and a control cleavage of a small amount of the resin (1 mg) was carried out that revealed complete removal of the Dde group. Anal. HPLC: major peak with elution at 52% ACN; *m/z* calculated for $C_{51}H_{72}N_7O_{12}S$ $[M+H]^+$: 1006.5 Da; found: 1006.5 Da; *m/z* calculated for $C_{51}H_{71}N_7NaO_{12}S$ $[M+Na]^+$: 1028.5 Da; found: 1028.5 Da.

Further N-terminal extension of the sequence resulted in 6-methylheptanoyl-Dab(Boc)-Thr(tBu)-Dab(Boc)-Dab(Dde)-Dab(Boc)-DLeu-Leu-Dab(Boc)-Dab(4-pentinoyl-Bpa-Met(O_2)-Oic-Abu)-Thr(tBu)-resin (**43**). The side-chain Dde group was removed with hydrazine and the linear peptide was cleaved from the support with HFIP/DCM 1:4. After lyophilization from 1,4-dioxane crude **44** was obtained as a white solid that was used in the next step without purification. Yield 80.0 mg (assuming 33.1 µmol, 66%). Analytical HPLC: Major peak with elution at 84% ACN; m/z calculated for $C_{119}H_{195}N_{20}O_{30}S$ $[M+H]^+$: 2416.4 Da; found: 2416.4 Da; *m/z* calculated for $C_{119}H_{196}N_{20}O_{30}S$ $[M+2H]^{2+}$: 1208.7 Da; found: 1209.2 Da; *m/z* calculated for $C_{119}H_{195}N_{20}NaO_{30}S$ $[M+H+Na]^{2+}$: 1219.7 Da; found: 1220.2 Da.

6-Methylheptanoyl-Dab-Thr-Dab-cyclo[Dab-Dab-DLeu-Leu-Dab-Dab(4-pentinoyl-Bpa-Met(O_2)-Oic-Abu)-Thr] (45)

Crude **44** (25 mg, assuming 10.4 µmol) was cyclized following the procedure described for **32** and the product was obtained in 16.9 mg (assuming 7.0 µmol, 67%) crude yield as a light yellow waxy solid that was used in the next step without purification. Anal. HPLC: Major peak with elution at 95% ACN. *m/z* Calculated for $C_{119}H_{194}N_{20}O_{29}S$ $[M+2H]^{2+}$: 1199.7 Da; found: 1200.5 Da; *m/z* calculated for $C_{119}H_{193}N_{20}NaO_{29}S$ $[M+H+Na]^{2+}$: 1210.7 Da; found: 1211.5 Da; *m/z* calculated for $C_{119}H_{192}N_{20}NaO_{29}S$ $[M+2Na]^{2+}$: 1221.7 Da; found: 1222.5 Da.

Deprotection of **cyclized 44** (20 mg, assuming 8.3 µmol) was carried out as described for **cyclized 32** and the product **45** was purified by prep. HPLC with elution at 50% ACN and lyophilized. Yield 3.8 mg (1.6 µmol (with 4 x TFA), 19% based on crude **cyclized 44**). *m/z* Calculated for $C_{91}H_{145}N_{20}O_{21}S$ $[M+H]^+$:

1886.1 Da; found: 1886.1 Da; *m/z* calculated for $C_{91}H_{146}N_{20}O_{21}S$ $[M+2H]^{2+}$: 943.5 Da; found: 943.7 Da; *m/z* calculated for $C_{91}H_{147}N_{20}O_{21}S$ $[M+3H]^{3+}$: 629.4 Da; found: 629.5 Da; *m/z* calculated for $C_{91}H_{148}N_{20}O_{21}S$ $[M+4H]^{4+}$: 472.3 Da; found: 472.5 Da.

6-Methylheptanoyl-Dab-Thr-Dab-cyclo[Dab-Dab-DLeu-Leu-Dab(T-G-R-A-K-R-R-M-Q-Y-N-R-R-ßAla-norleucinamide(1*H*-1,2,3-triazol-4-yl)propionyl-Bpa-Met(O$_2$)-Oic-Abu)-Dab-Thr] (46)

45 (3.80 mg, 1.6 µmol) and **4** (5.2 mg, 1.9 µmol) were coupled by click reaction as described for the synthesis of **6**. Purification by prep. HPLC with elution at 43% ACN gave **46** (5.4 mg, 1.1 µmol (with 11 x TFA), 69% based on **45**). *m/z* calculated for $C_{168}H_{287}N_{57}O_{40}S_2$ $[M+6H]^{6+}$: 634.5264 Da; found: 634.5284 Da; *m/z* calculated for $C_{168}H_{287}N_{57}O_{41}S_2$ $[M+O+6H]^{6+}$: 637.1922 Da; found: 637.1940 Da; *m/z* calculated for $C_{168}H_{288}N_{57}O_{40}S_2$ $[M+7H]^{7+}$: 544.0237 Da; found: 544.0254 Da; *m/z* calculated for $C_{168}H_{288}N_{57}O_{41}S_2$ $[M+O+7H]^{7+}$: 546.3087 Da; found: 546.3104 Da; *m/z* calculated for $C_{168}H_{289}N_{57}O_{40}S_2$ $[M+8H]^{8+}$: 476.1466 Da; found: 476.1481 Da; *m/z* calculated for $C_{168}H_{289}N_{57}O_{41}S_2$ $[M+O+8H]^{8+}$: 478.1460 Da; found: 478.1478 Da.

7 Analytical Data

1. **HPLC** Data

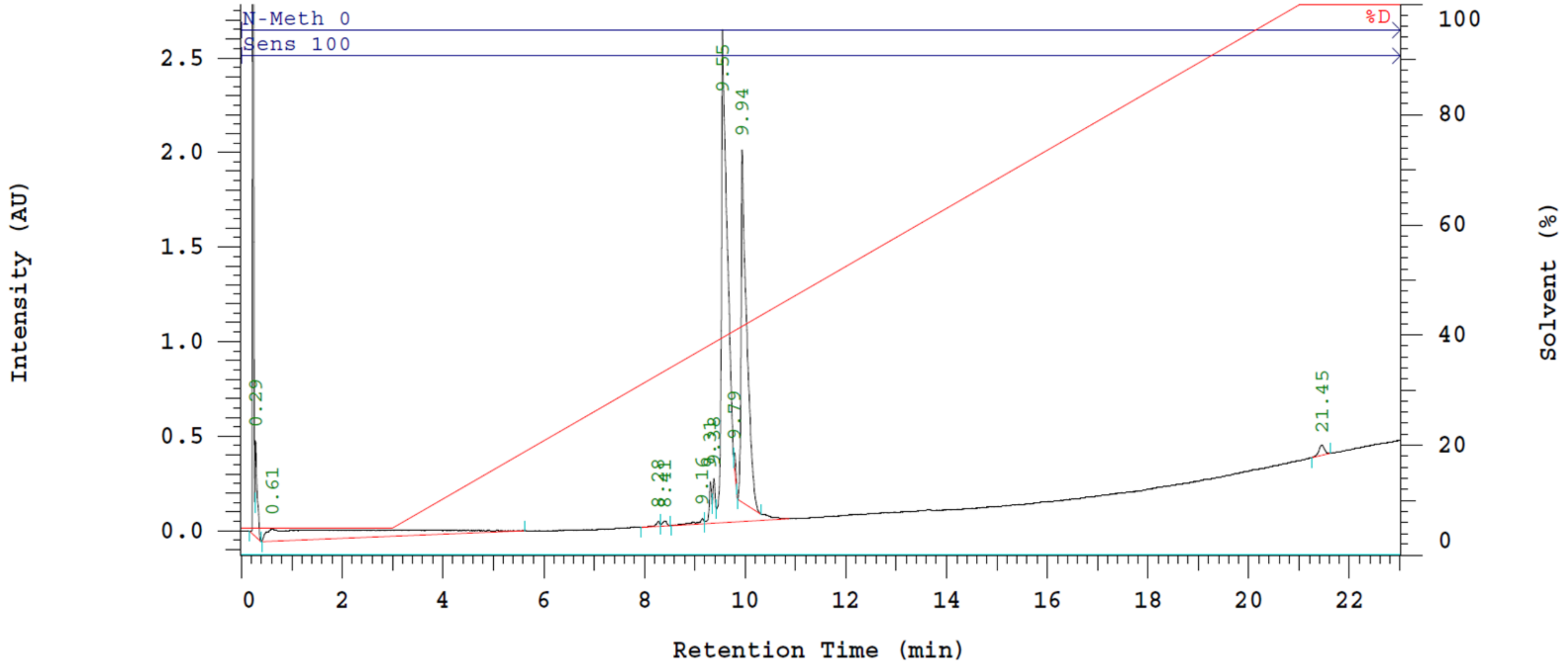

Analytical HPLC of colistin sulfate from Cayman Inc. The signal at 9.55 min shows colistin B and the one at 9.94 min colistin A.

A

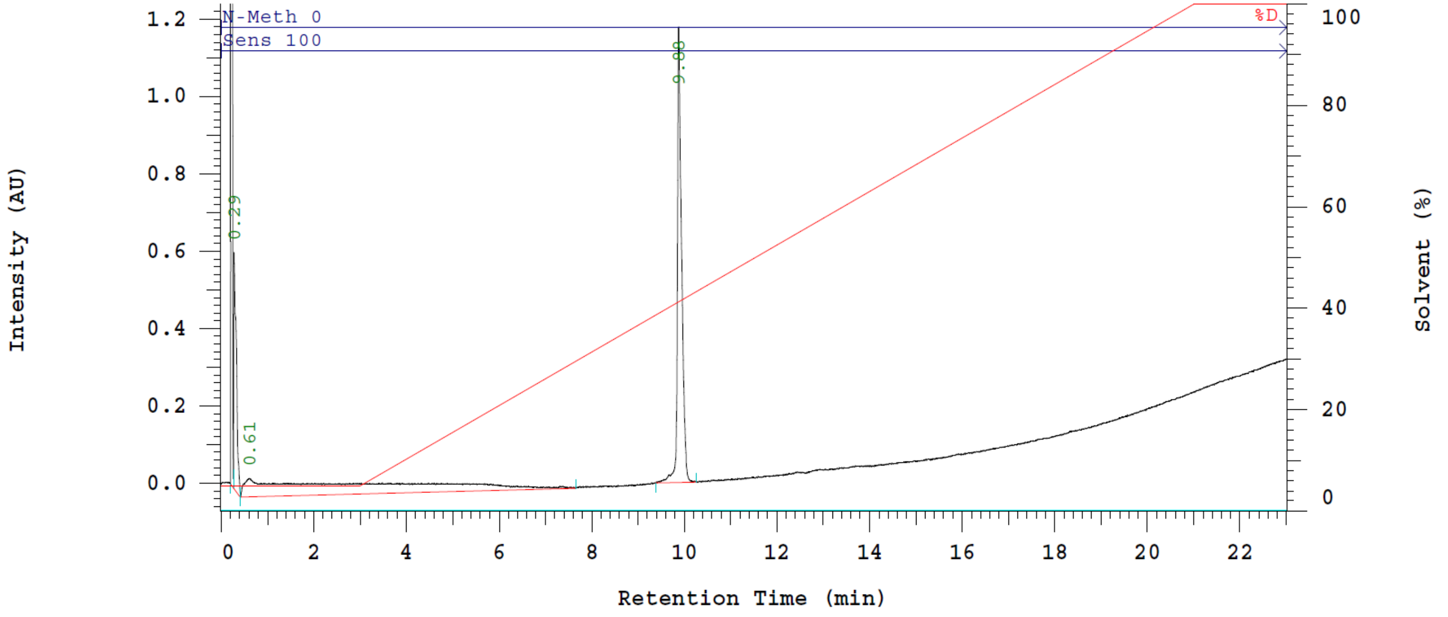

B

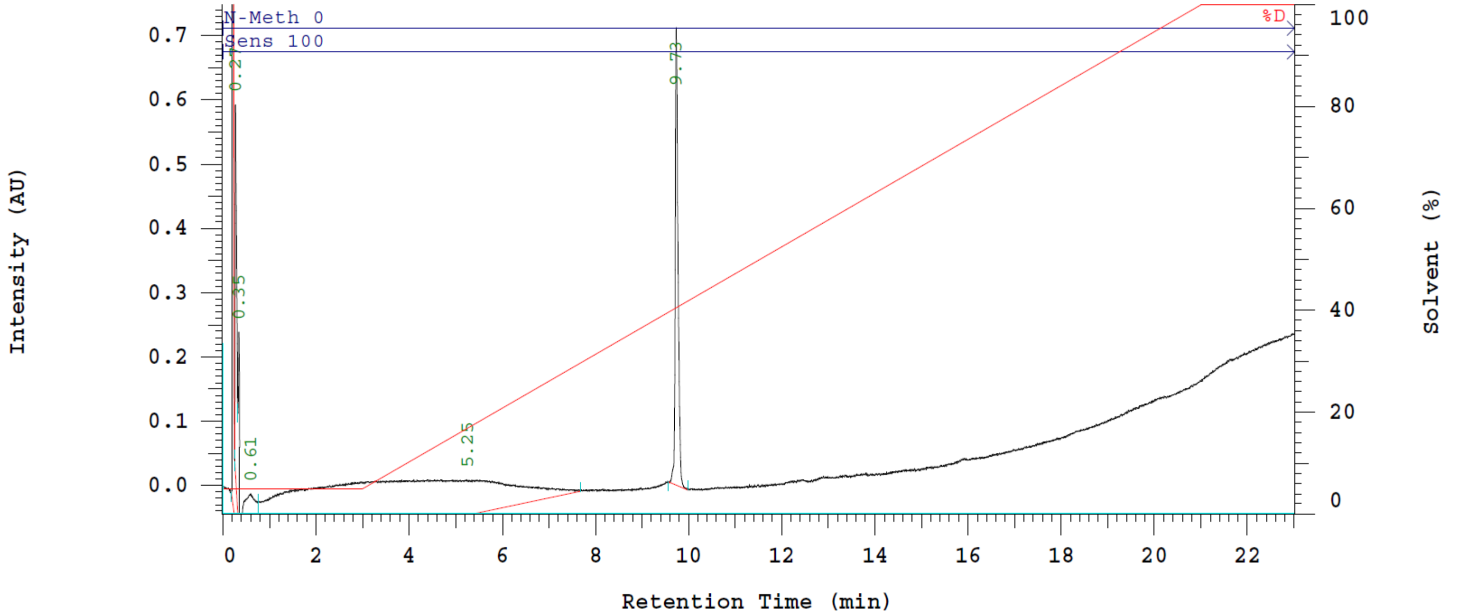

Analytical HPLC after prep. HPLC-purification of **A**: colistin B (**1**) and **B**: colistin A

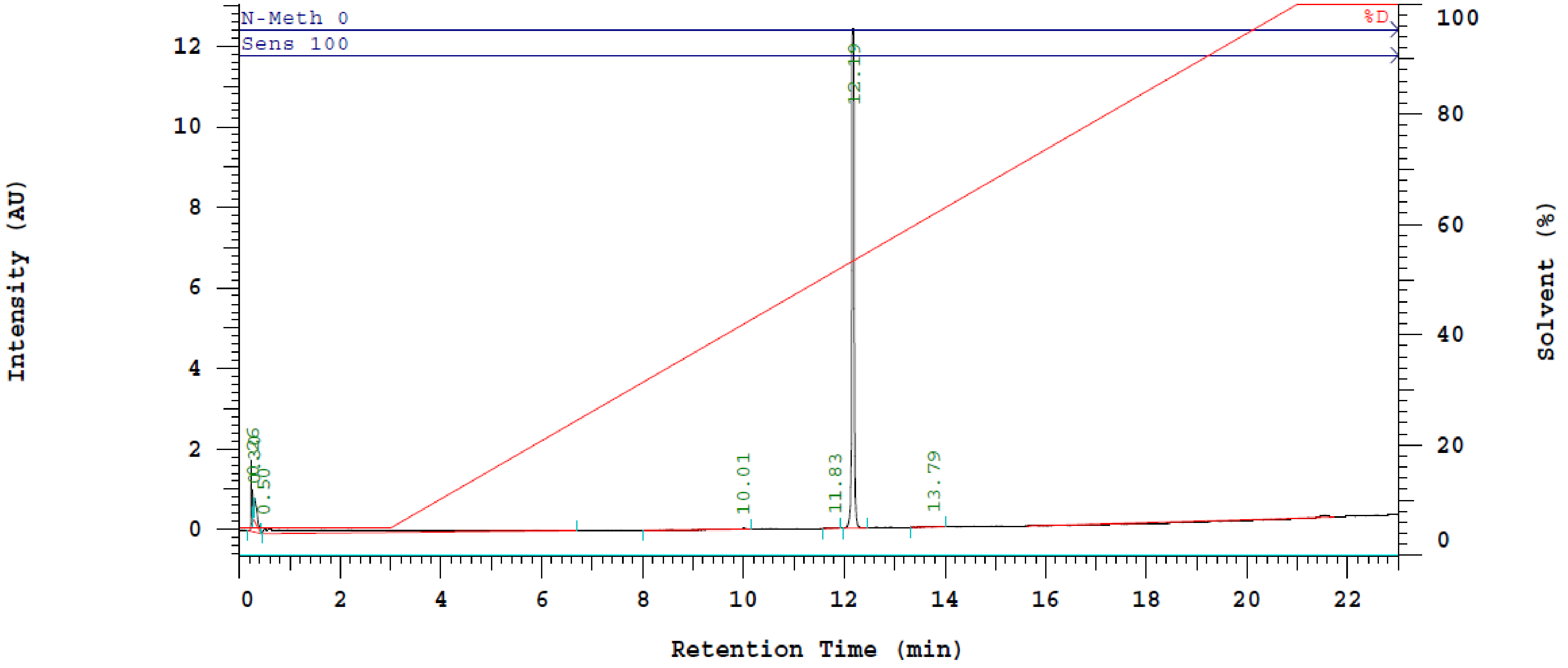

Analytical HPLC of purified **2**

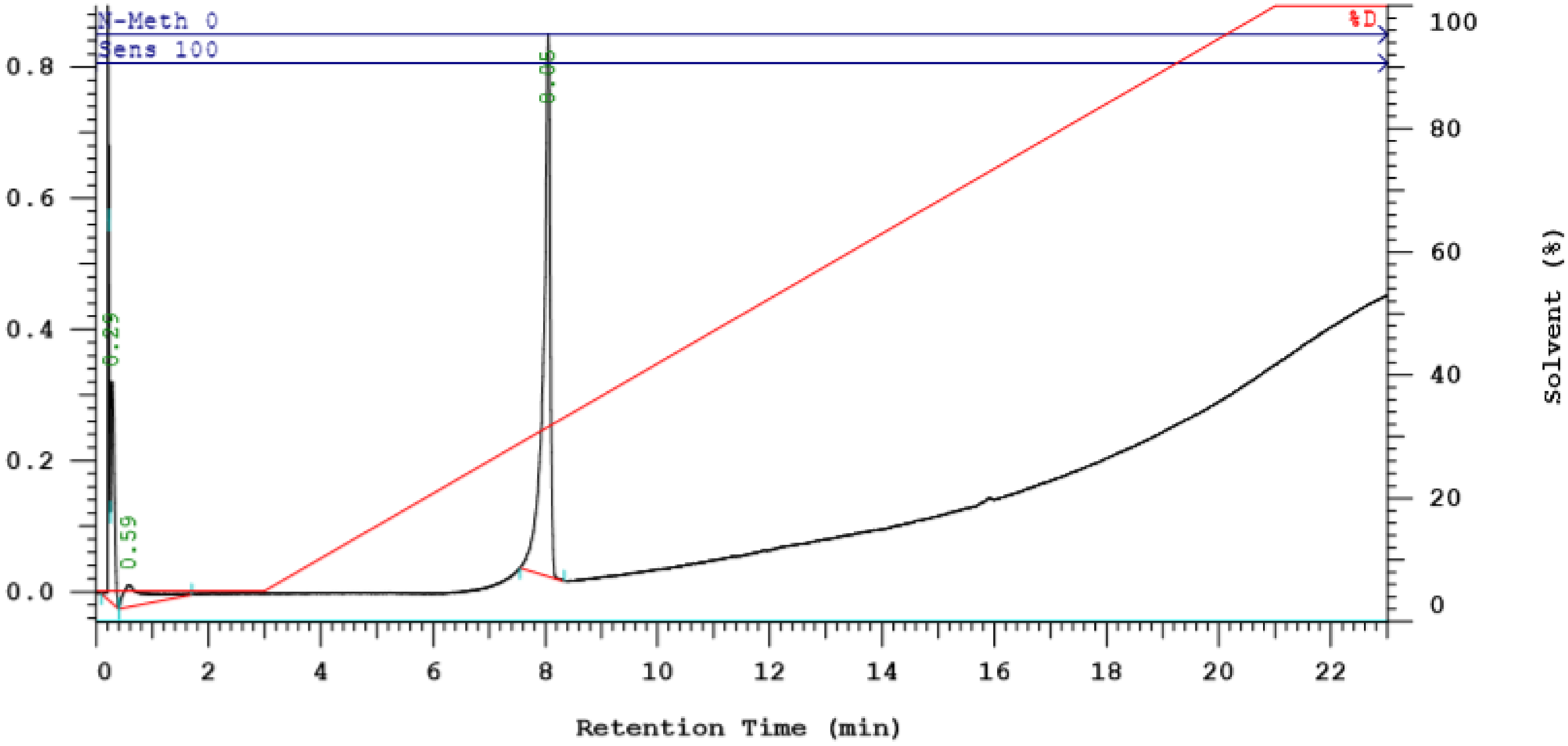

Analytical HPLC of purified **47**.

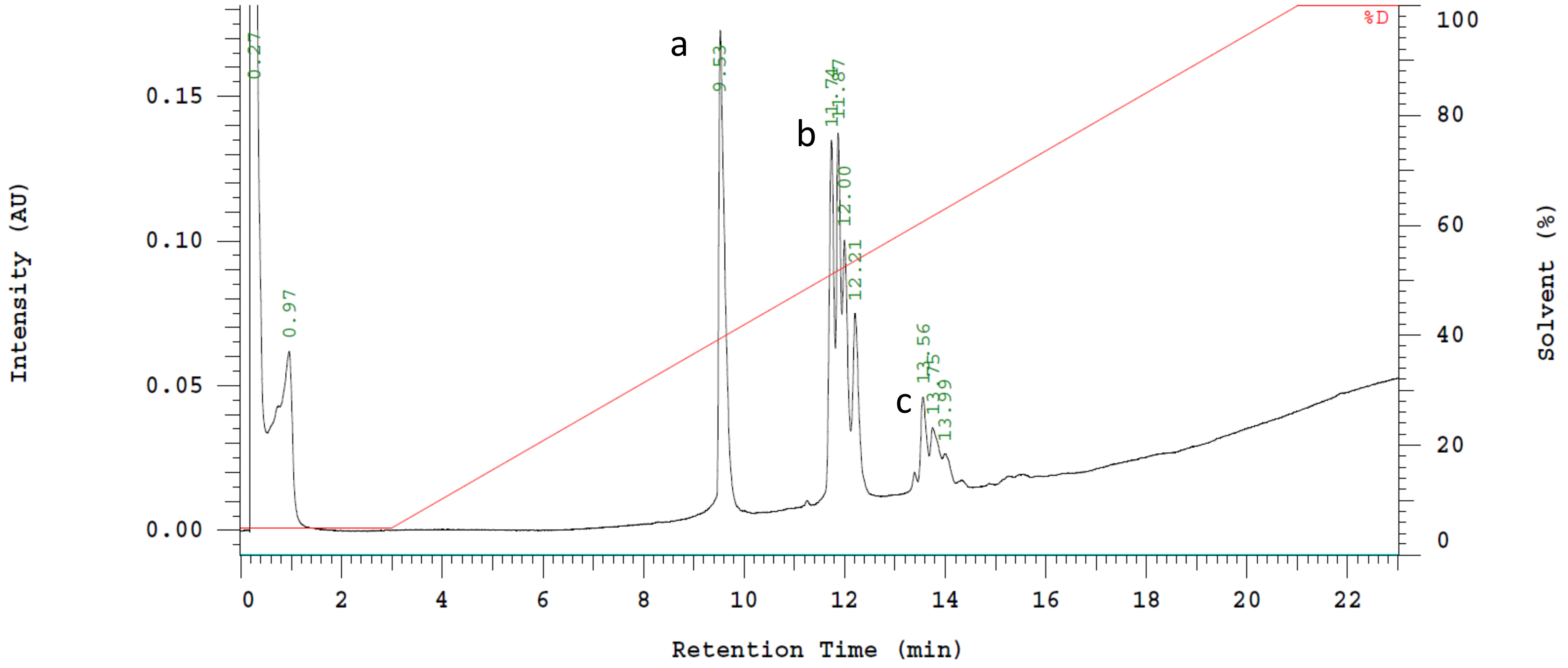

Formation of **3** after complete consumption of **2**; a: excess colistin **1**; b: **3**; c: **3** with additional attachment of a second molecule of **2**.

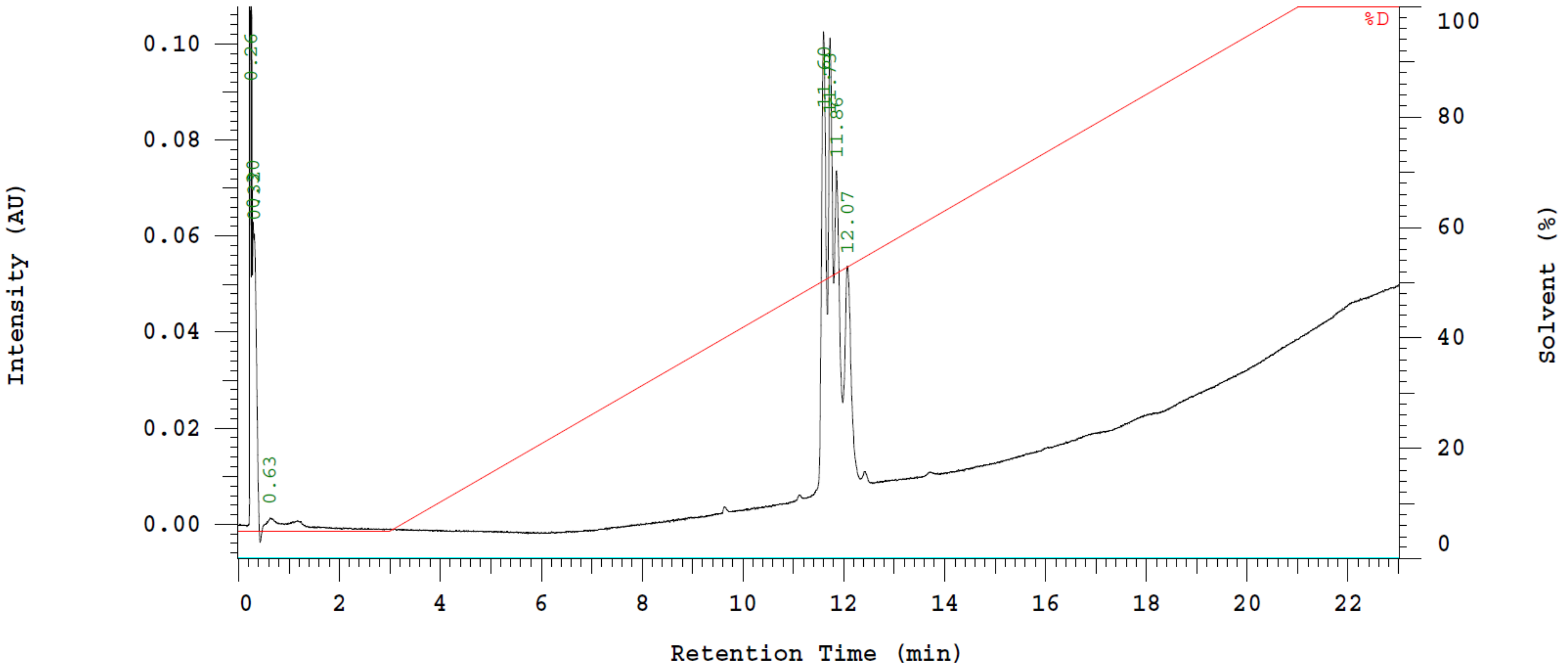

Analytical HPLC of purified **3**.

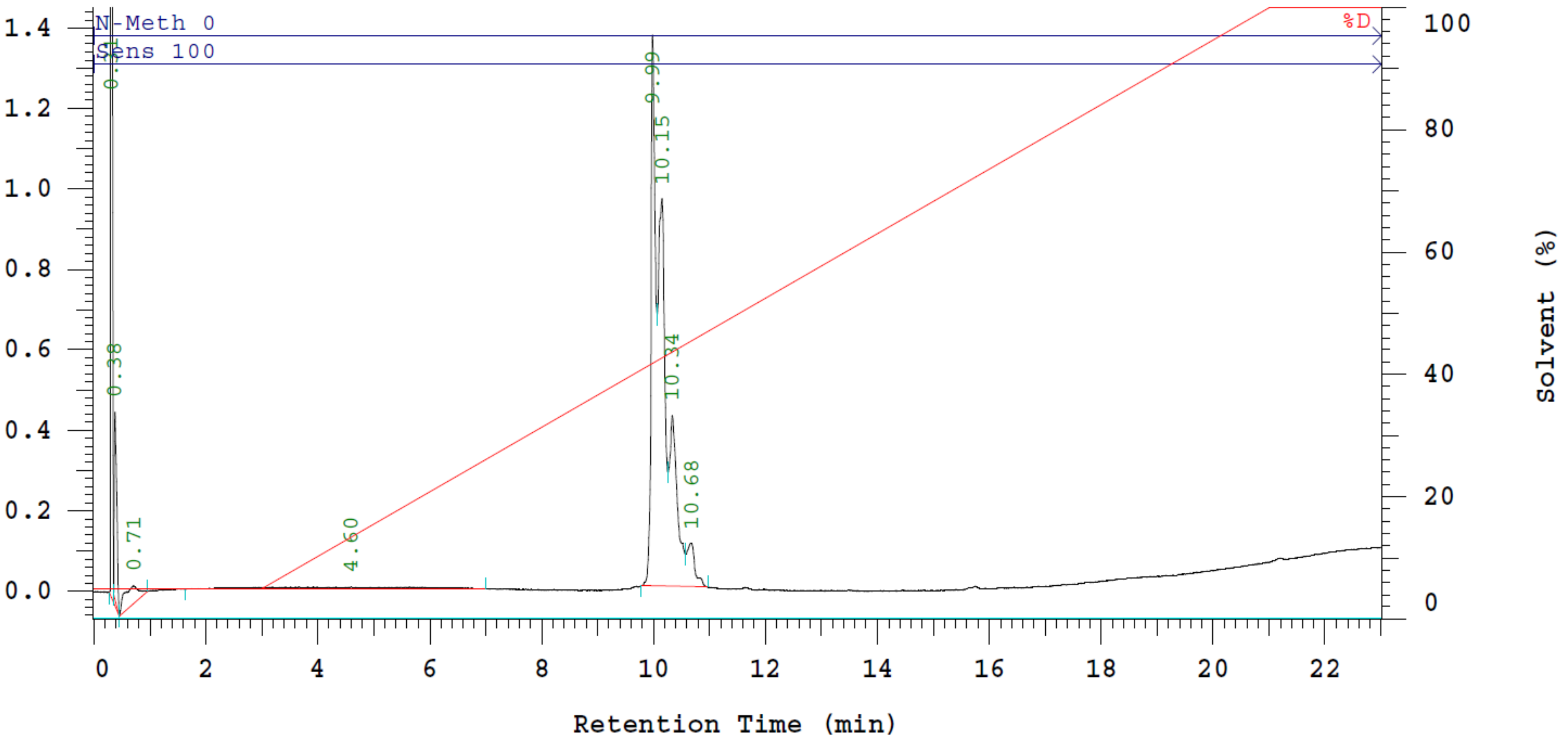

Analytical HPLC of purified **48**.

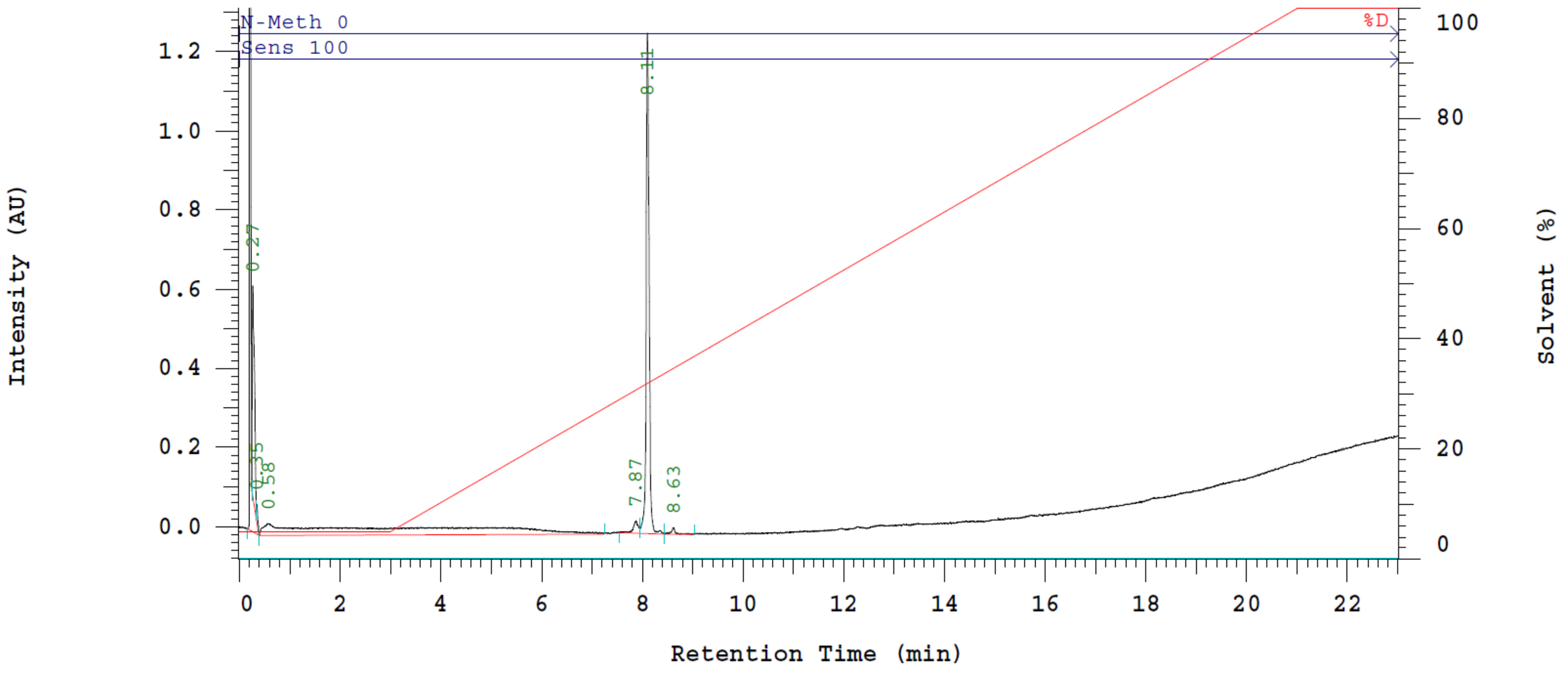

Analytical HPLC of purified **4**.

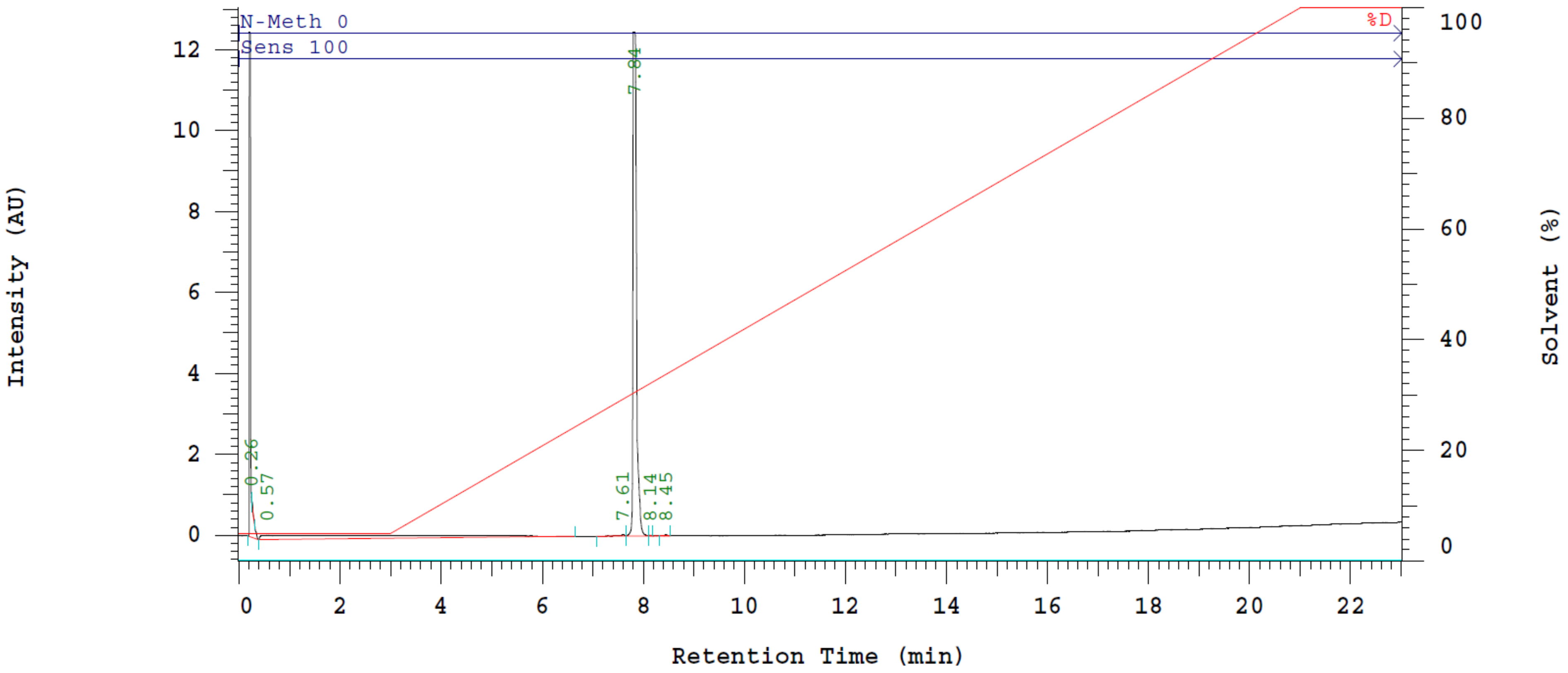

Analytical HPLC of purified **5**.

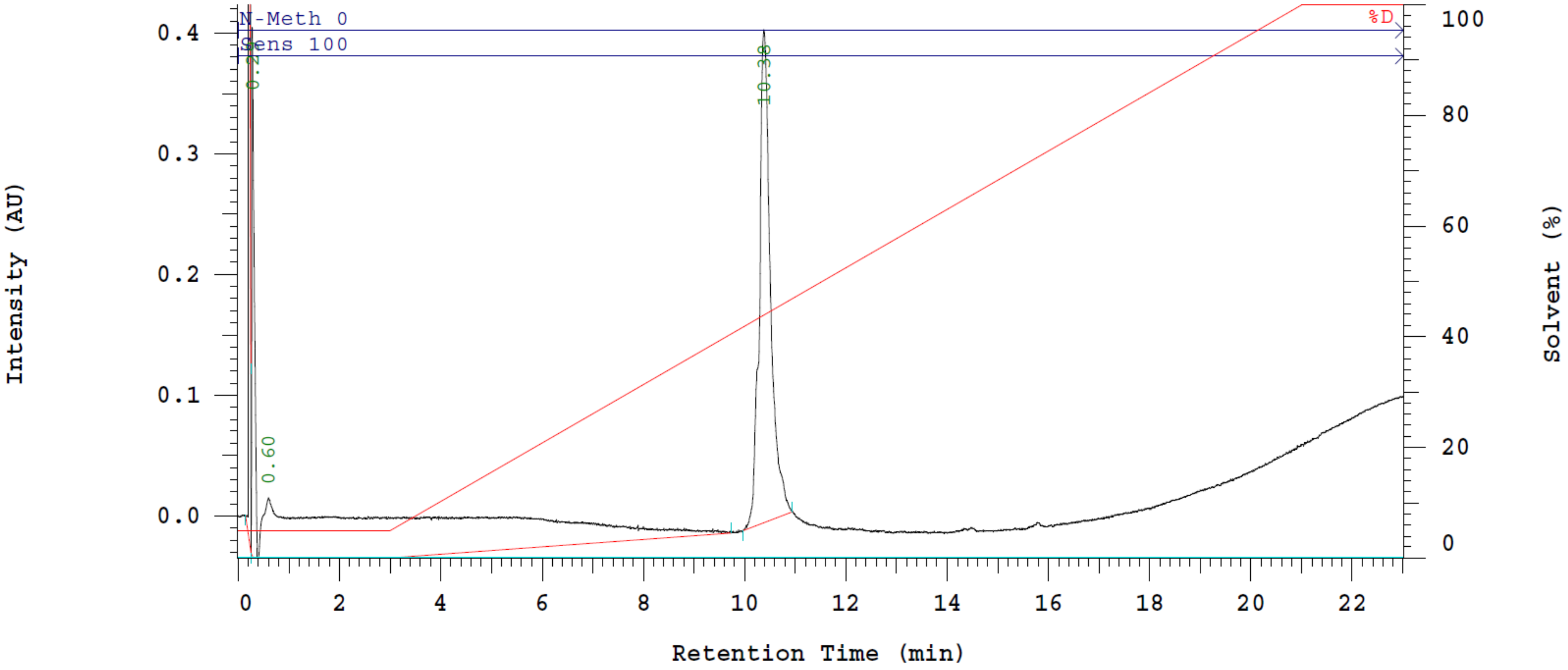

Analytical HPLC of purified **6**.

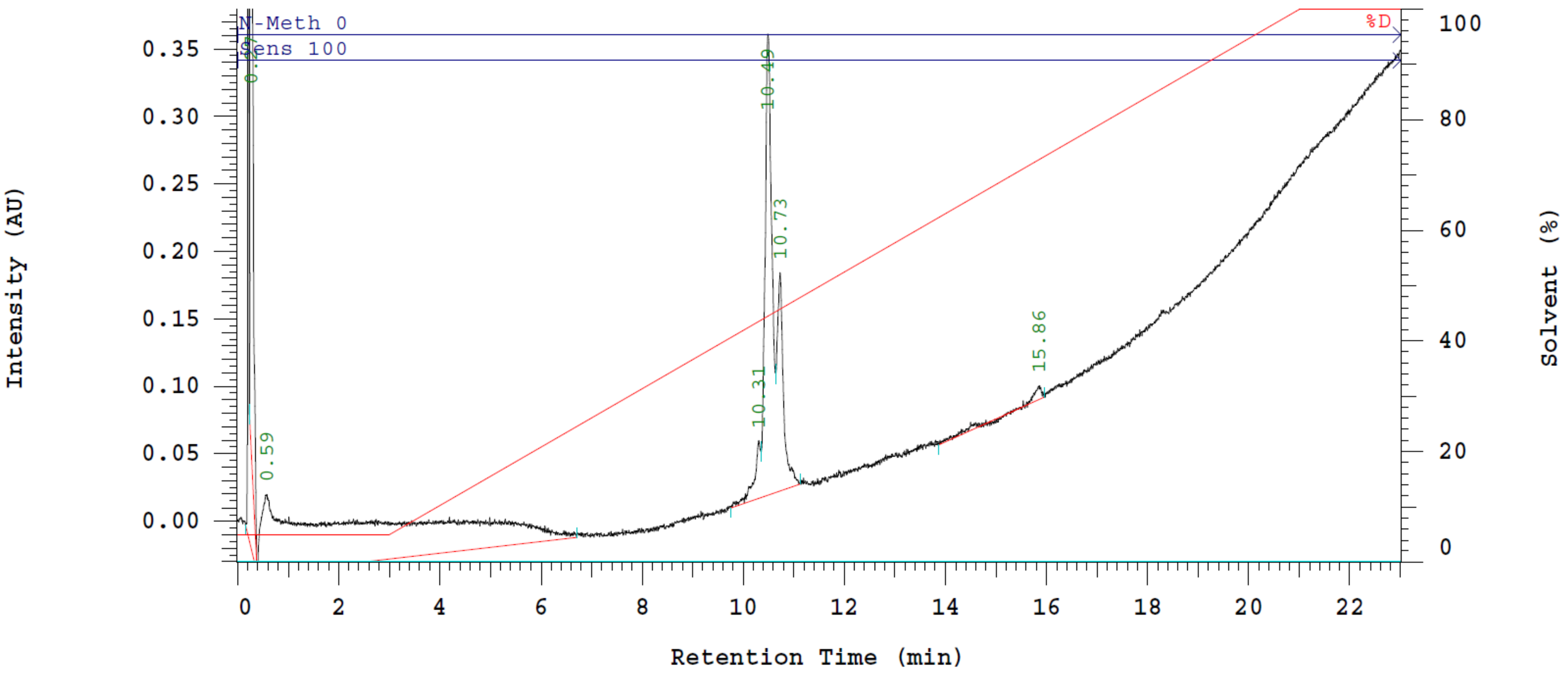

Analytical HPLC of purified **7**.

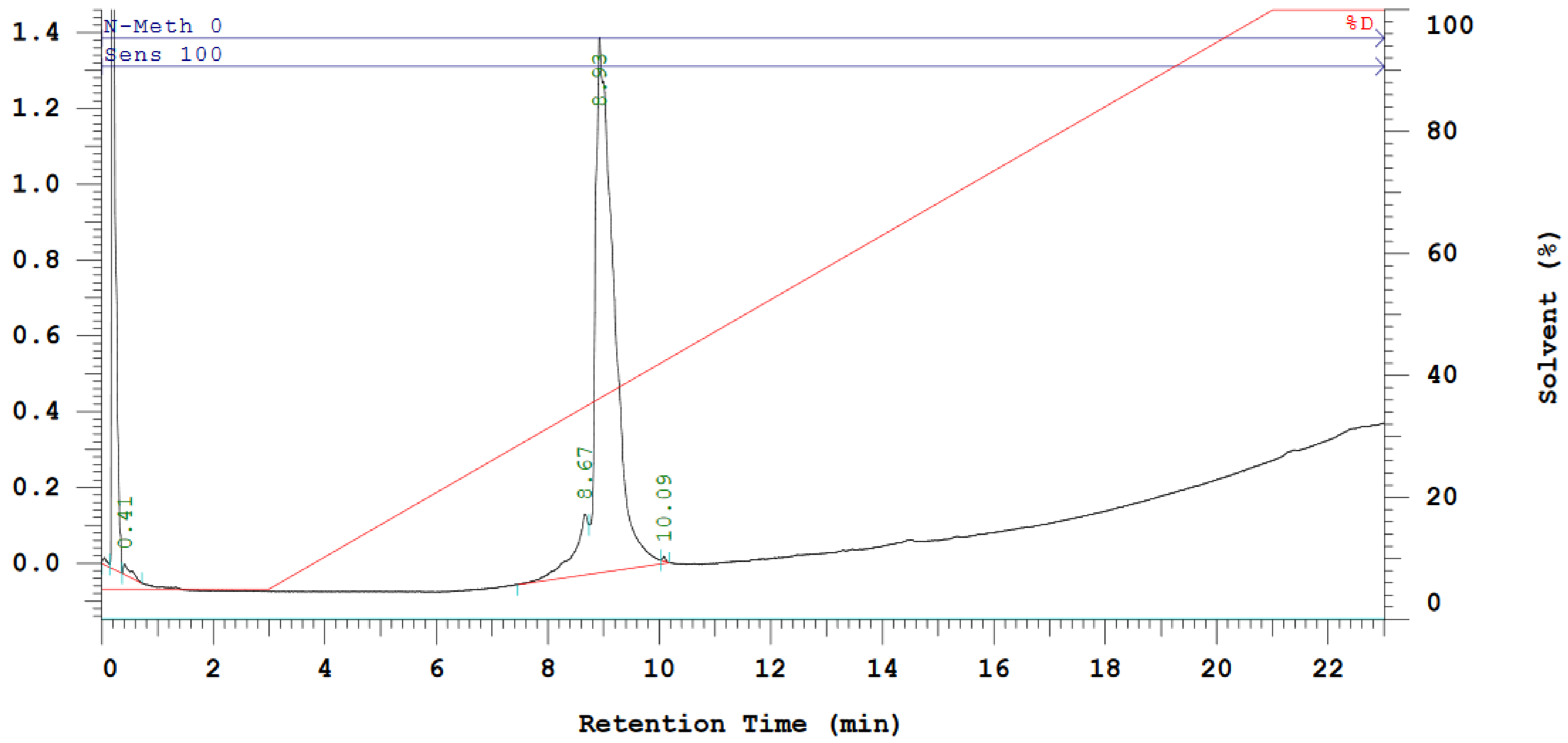

Analytical HPLC of purified **49**.

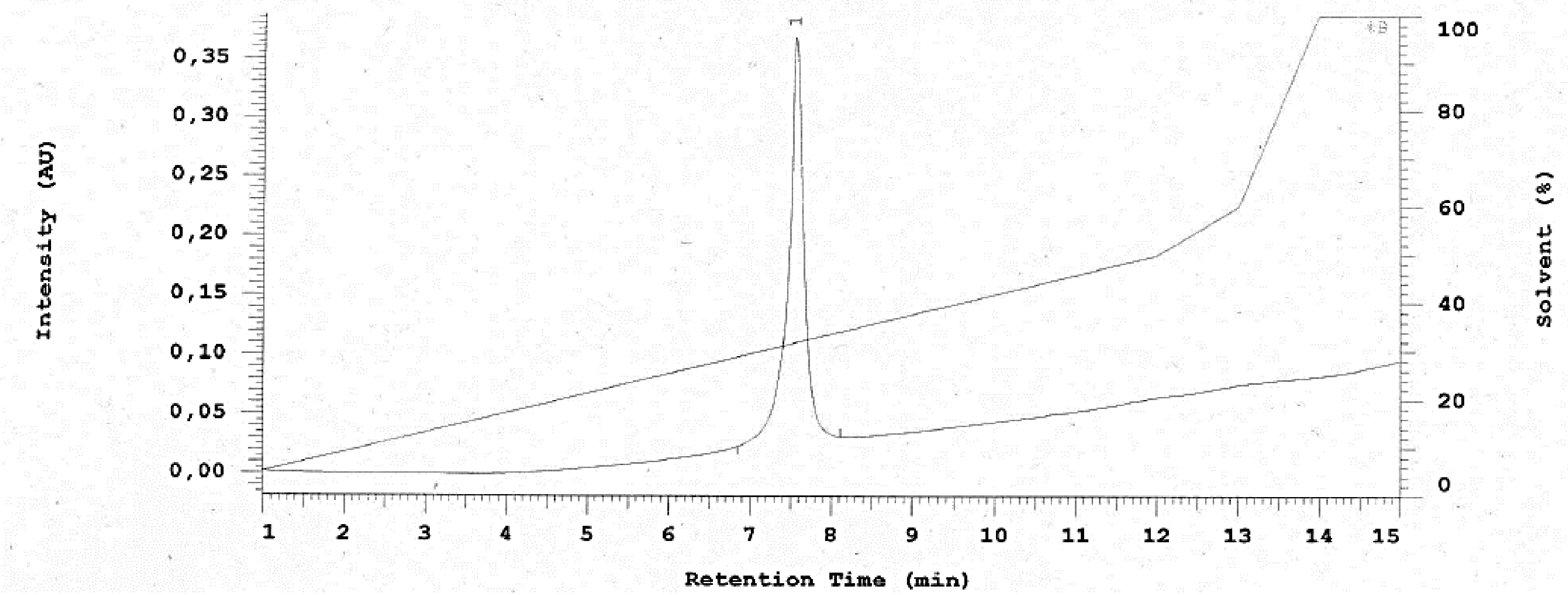

Analytical HPLC of purified **8**.

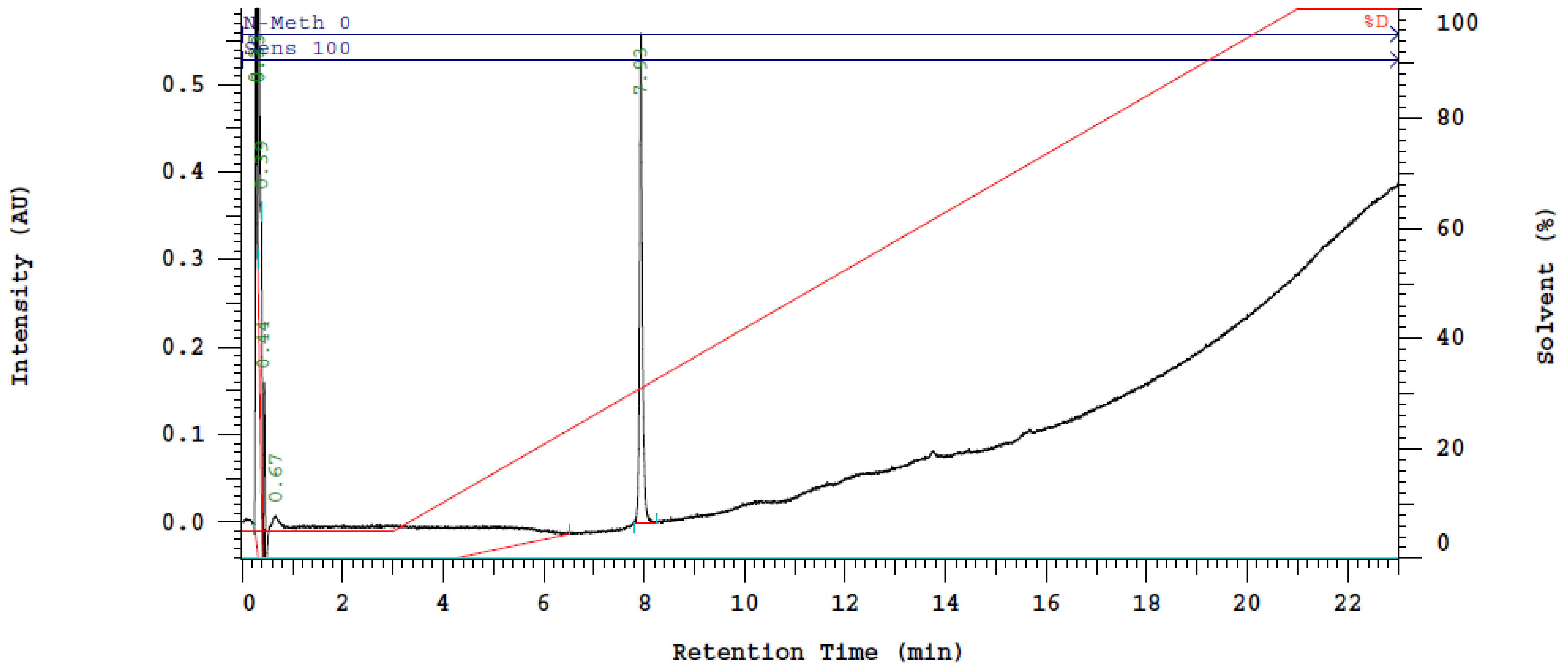

Analytical HPLC of purified **9**.

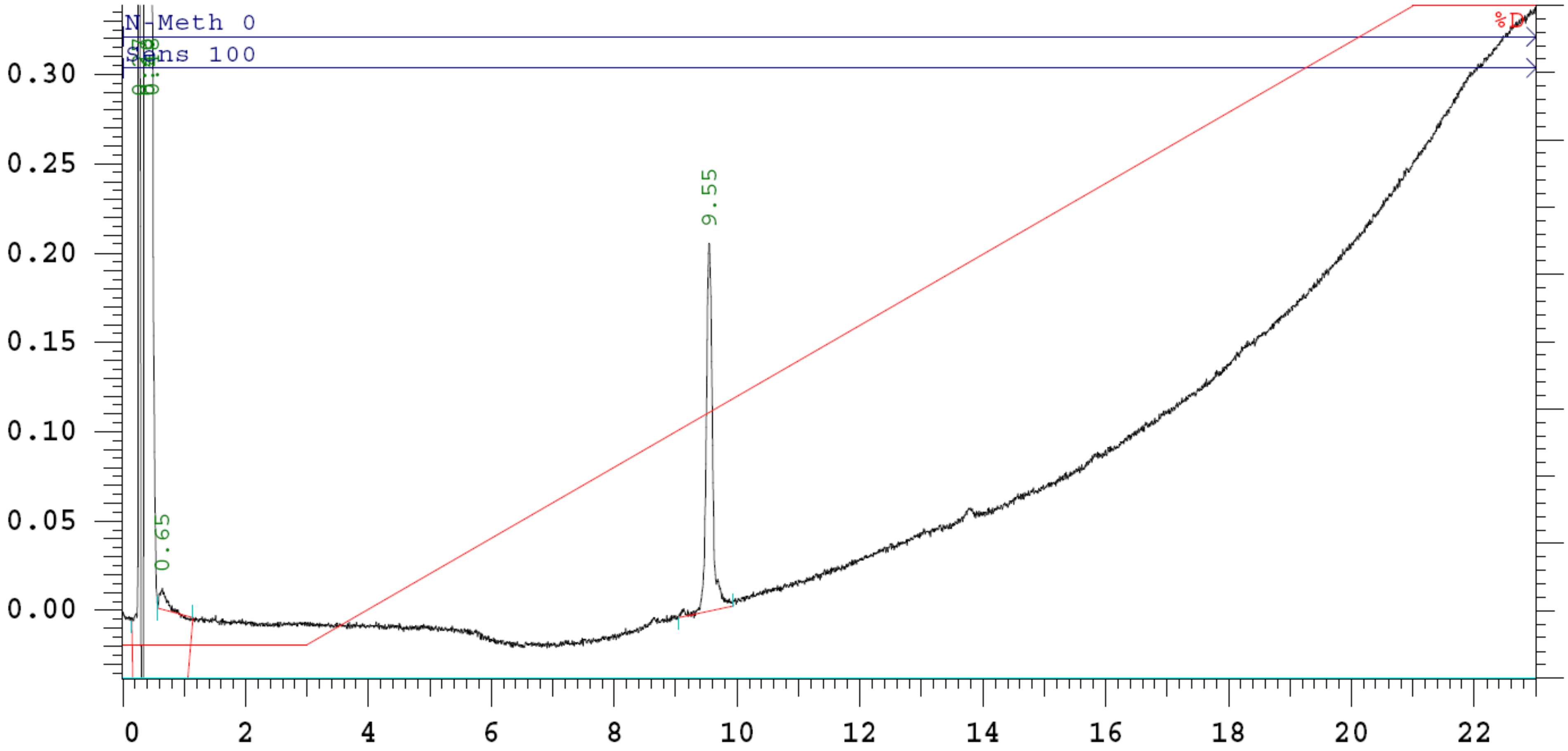

Analytical HPLC of purified **10**.

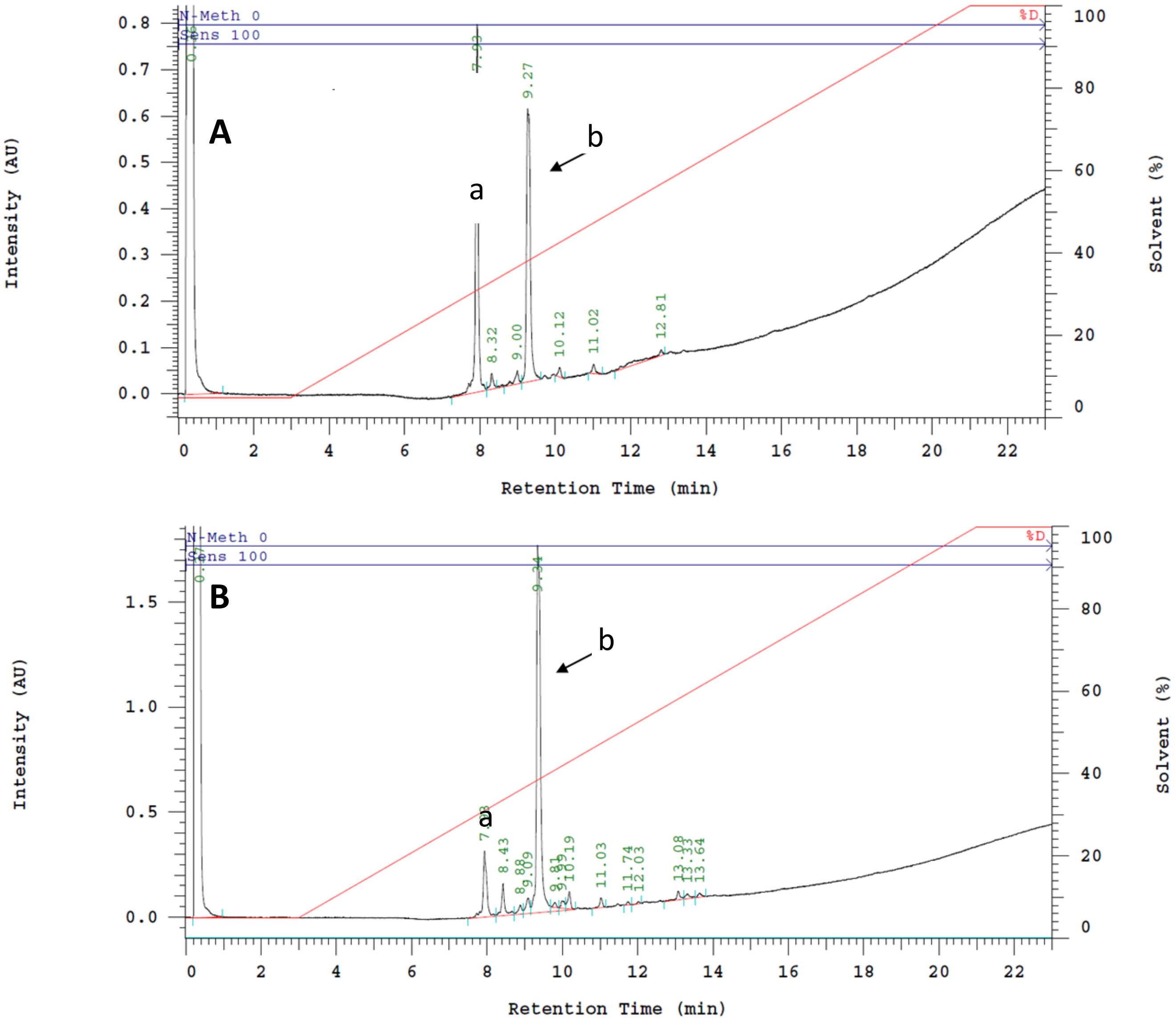

Analytical HPLC of the formation of **11** (reaction control); A: 5 min reaction time; B: 3 h reaction time; a: compound **9**, b: compound **11**.**A**: 5 min reaction time, 1 µmol F5M; **B**: 3 h reaction time, 3 µmol F5M.

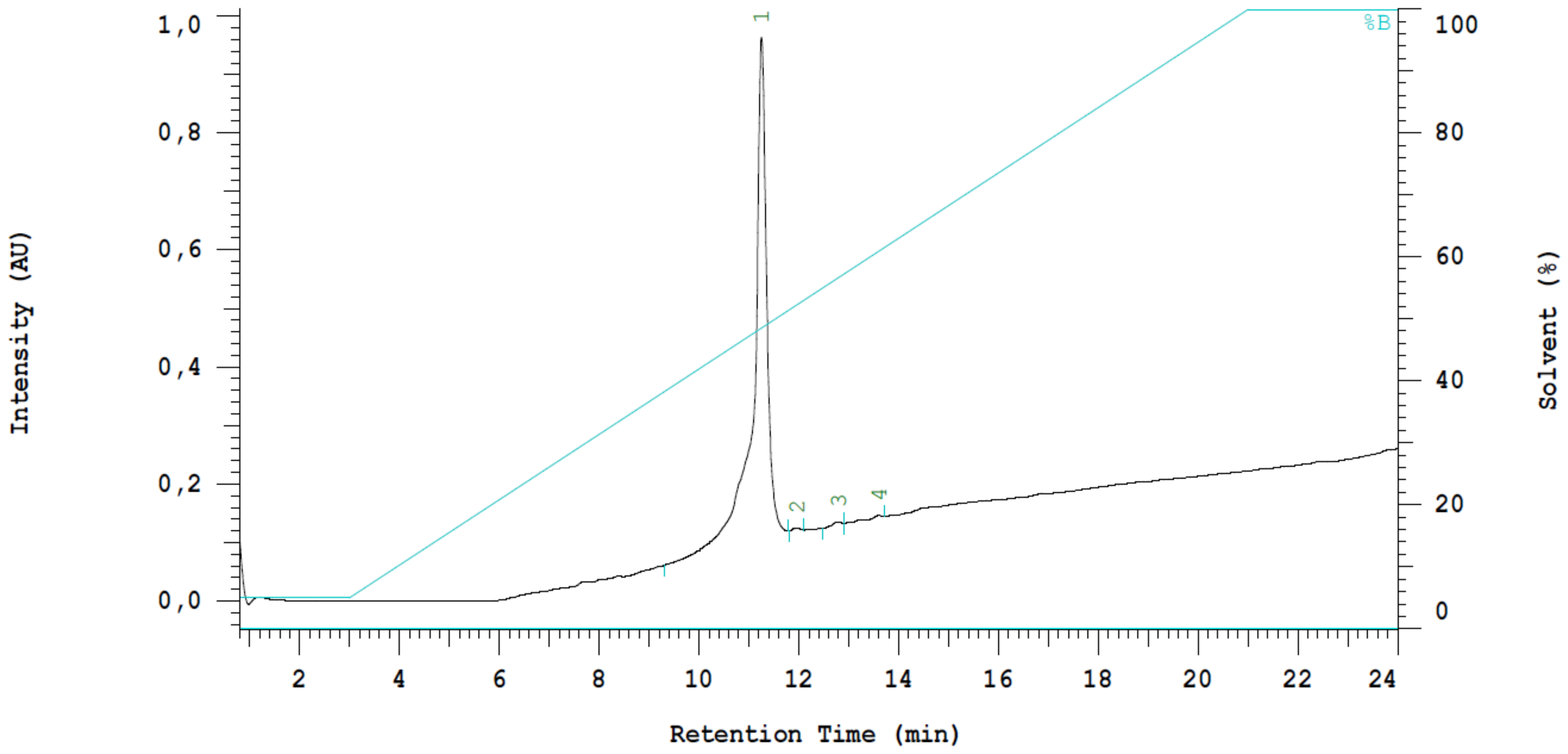

Analytical HPLC of purified **11**. Solvents with 0.1% formic acid.

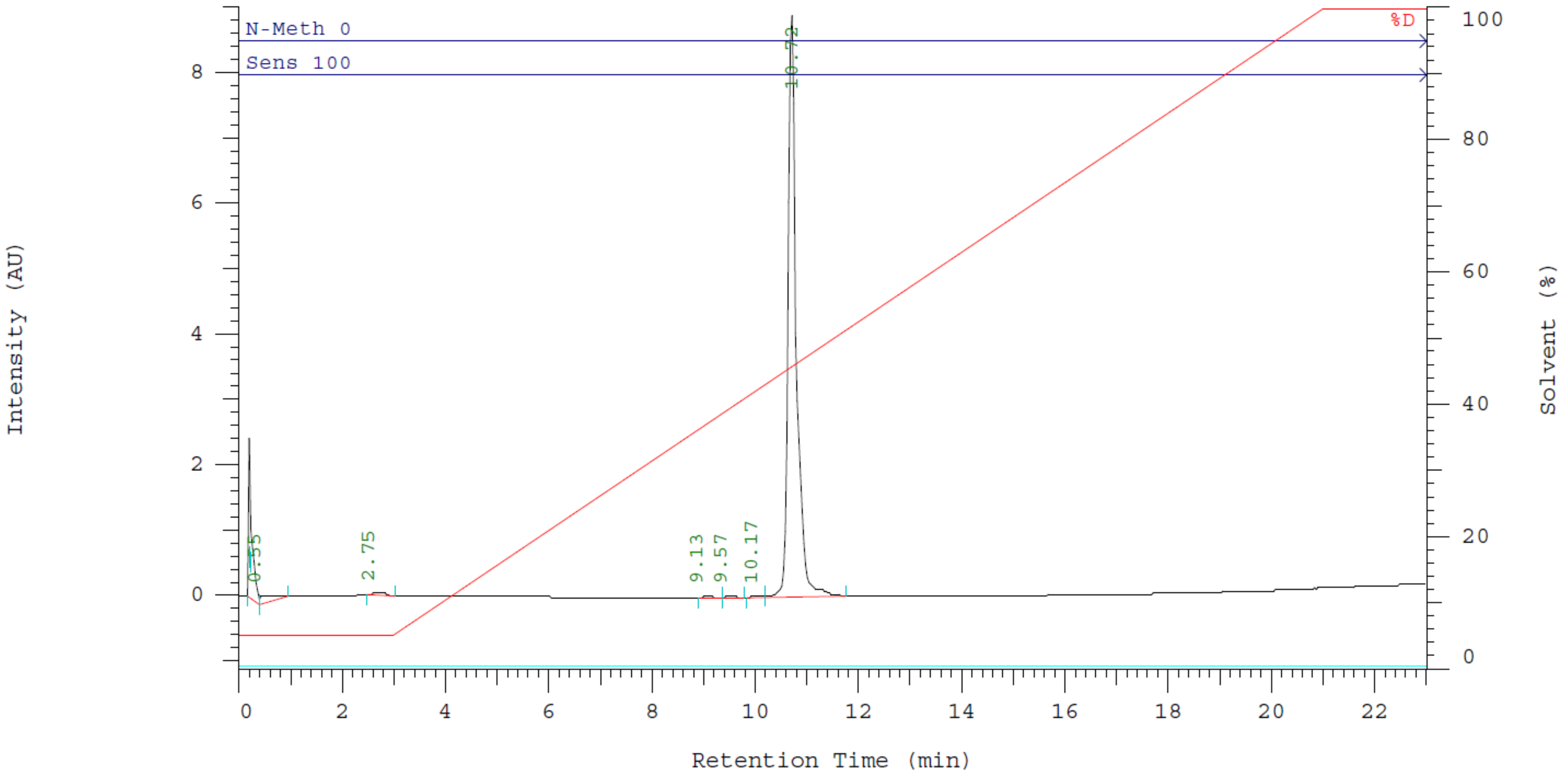

Analytical HPLC of purified **12**.

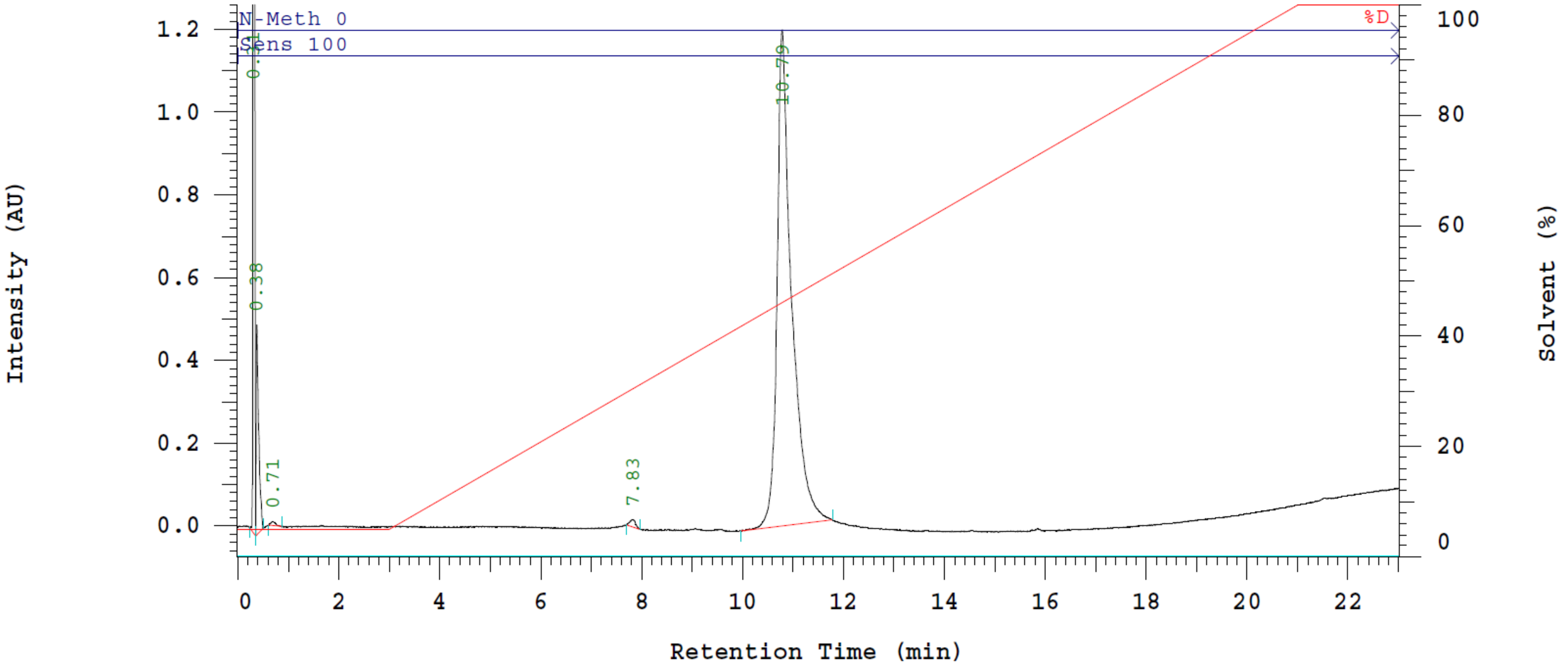

Analytical HPLC of purified **13**.

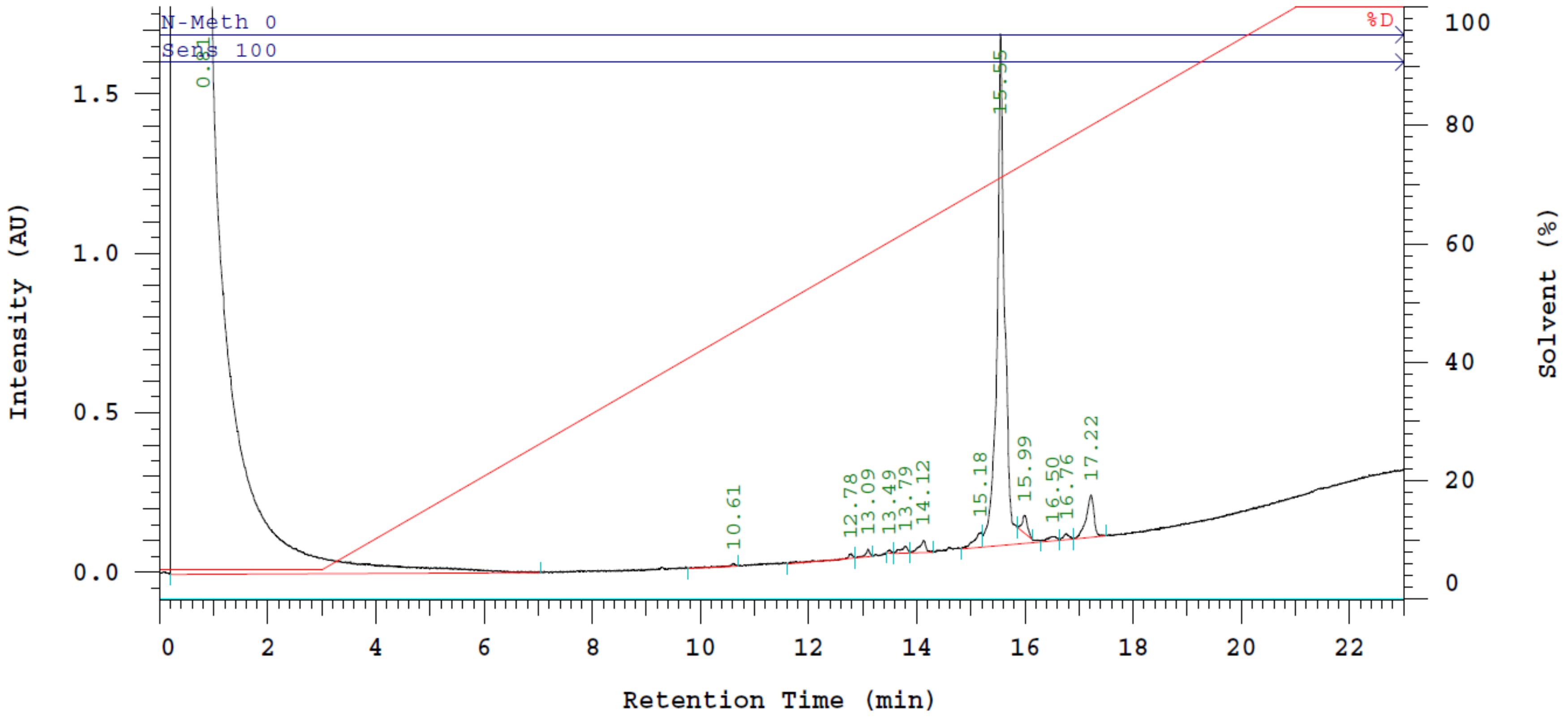

Analytical HPLC of crude **16**.

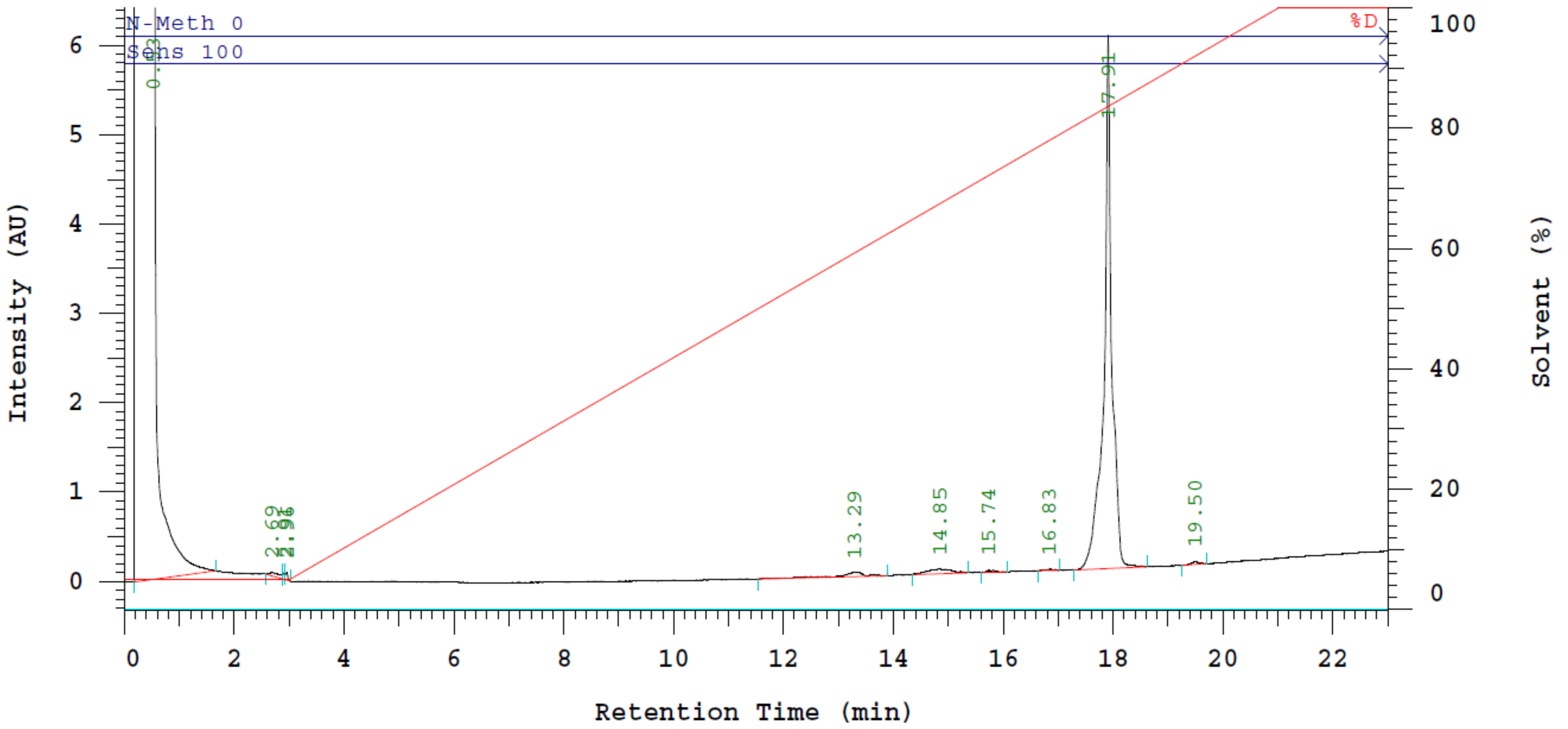

Analytical HPLC of crude **17** from trial cleavage.

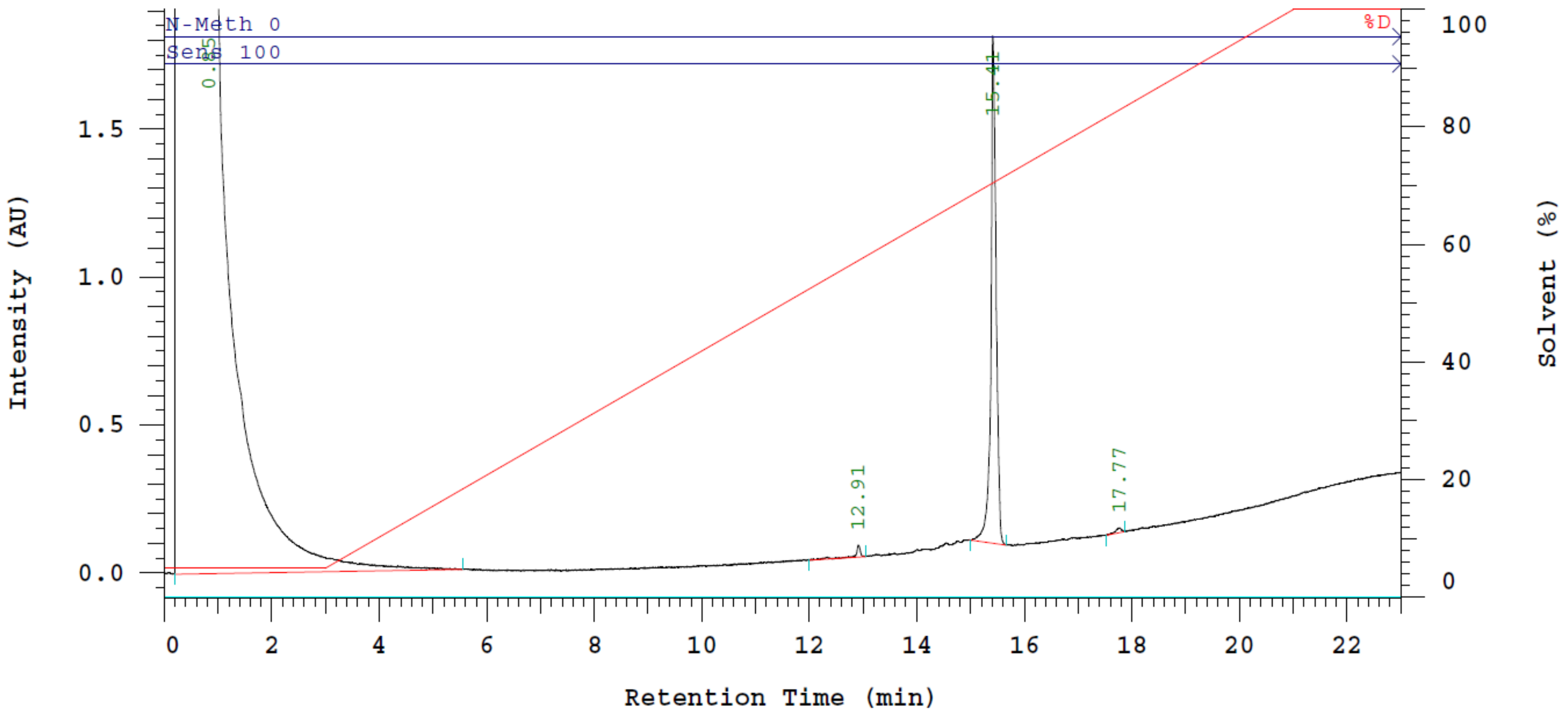

Analytical HPLC of crude **18**.

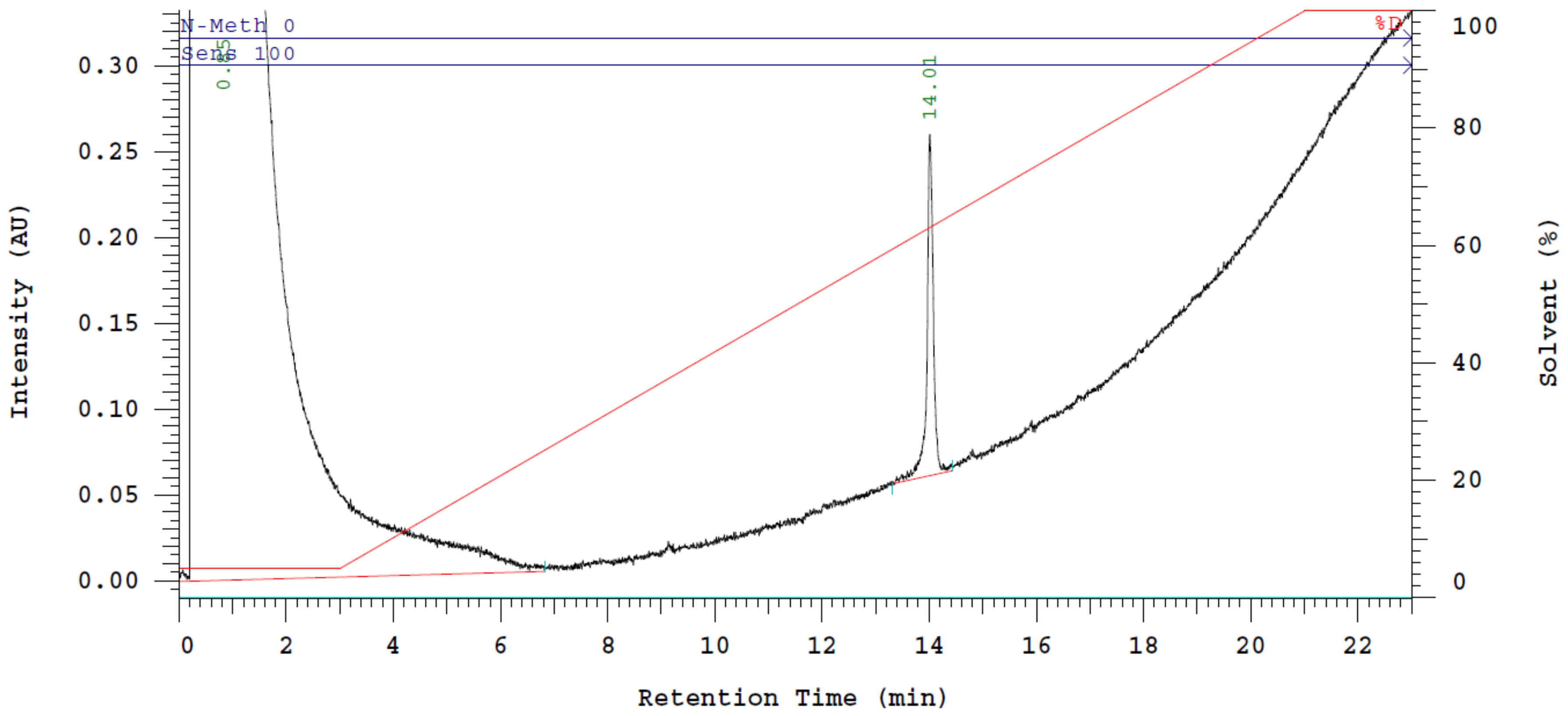

Analytical HPLC of purified **19**.

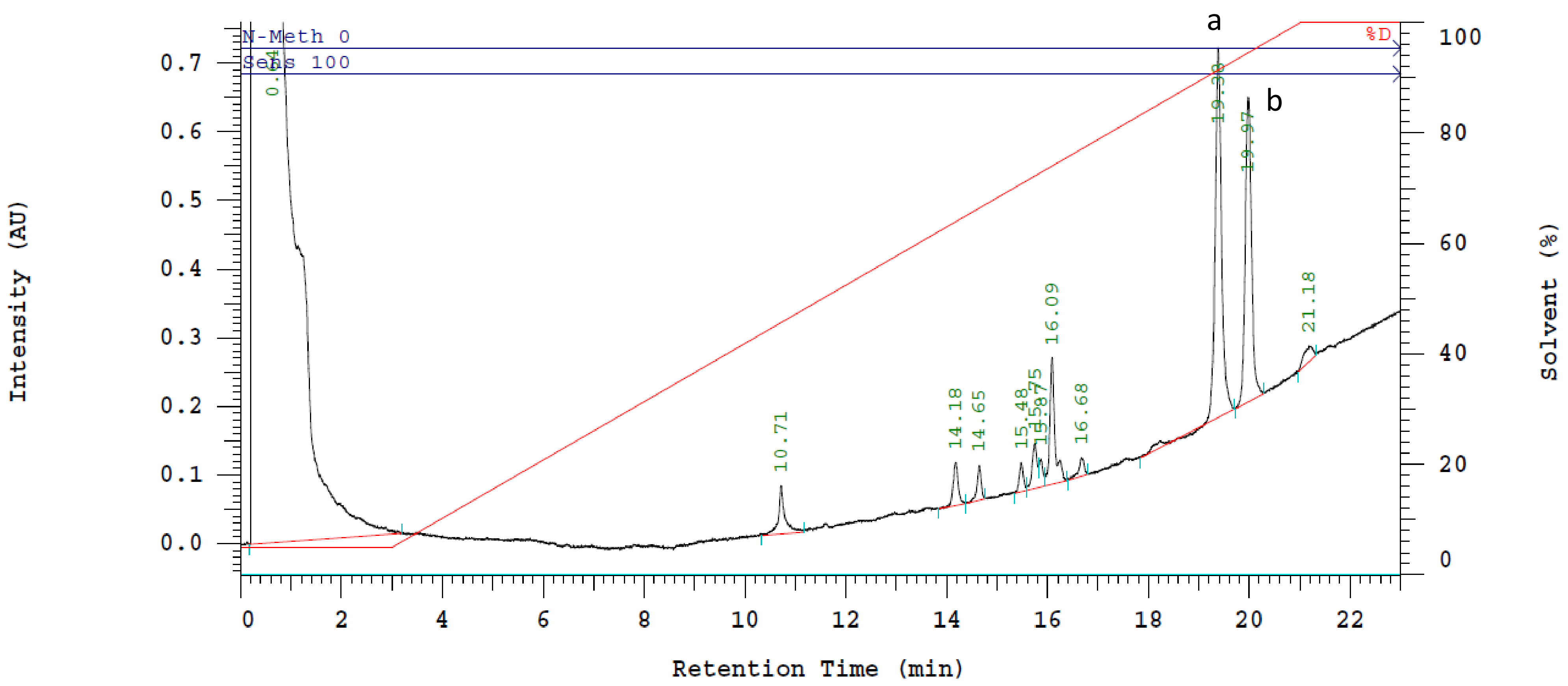

Analytical HPLC of the formation of **20** (reaction control). Peaks a and b showed the expected mass.

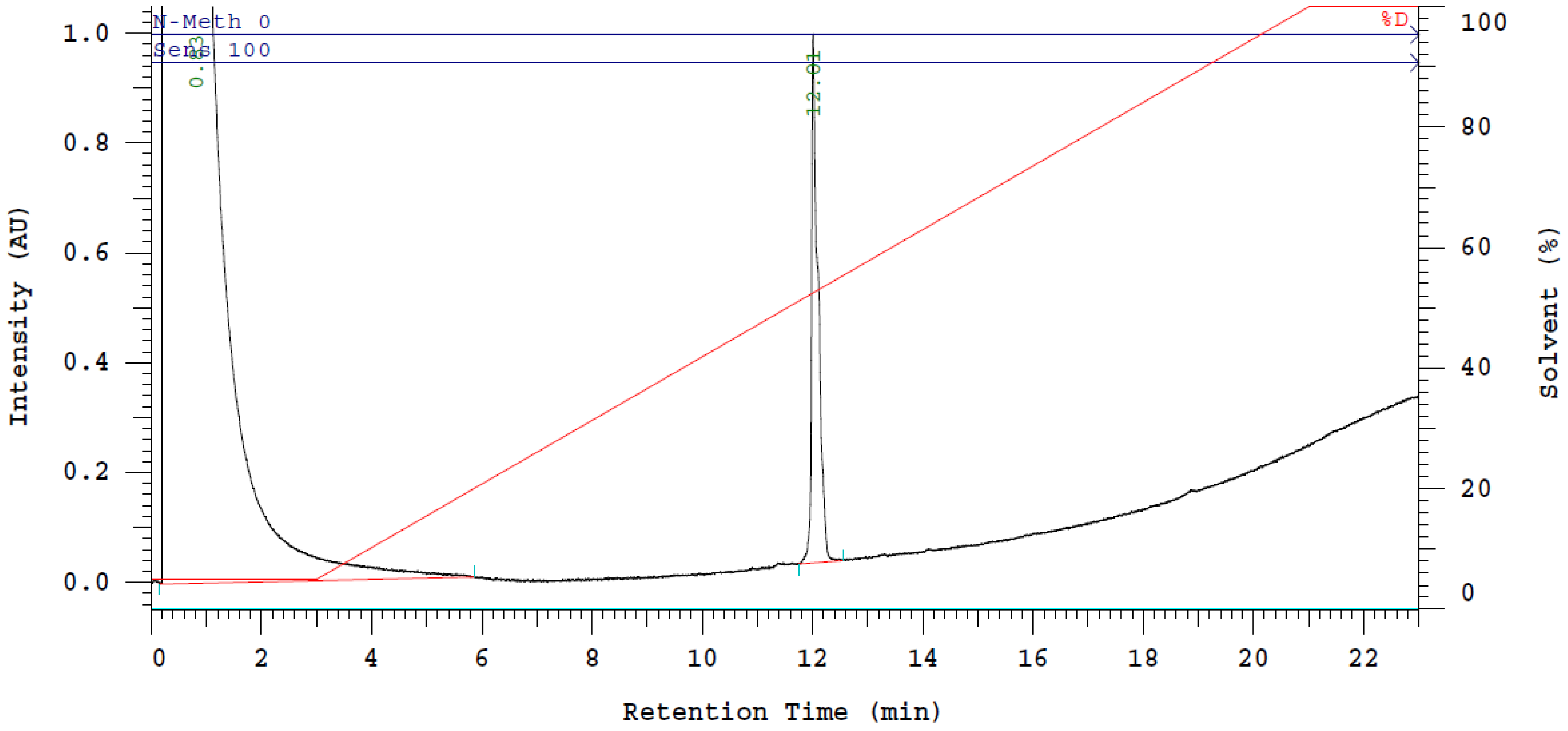

Analytical HPLC of purified **21**.

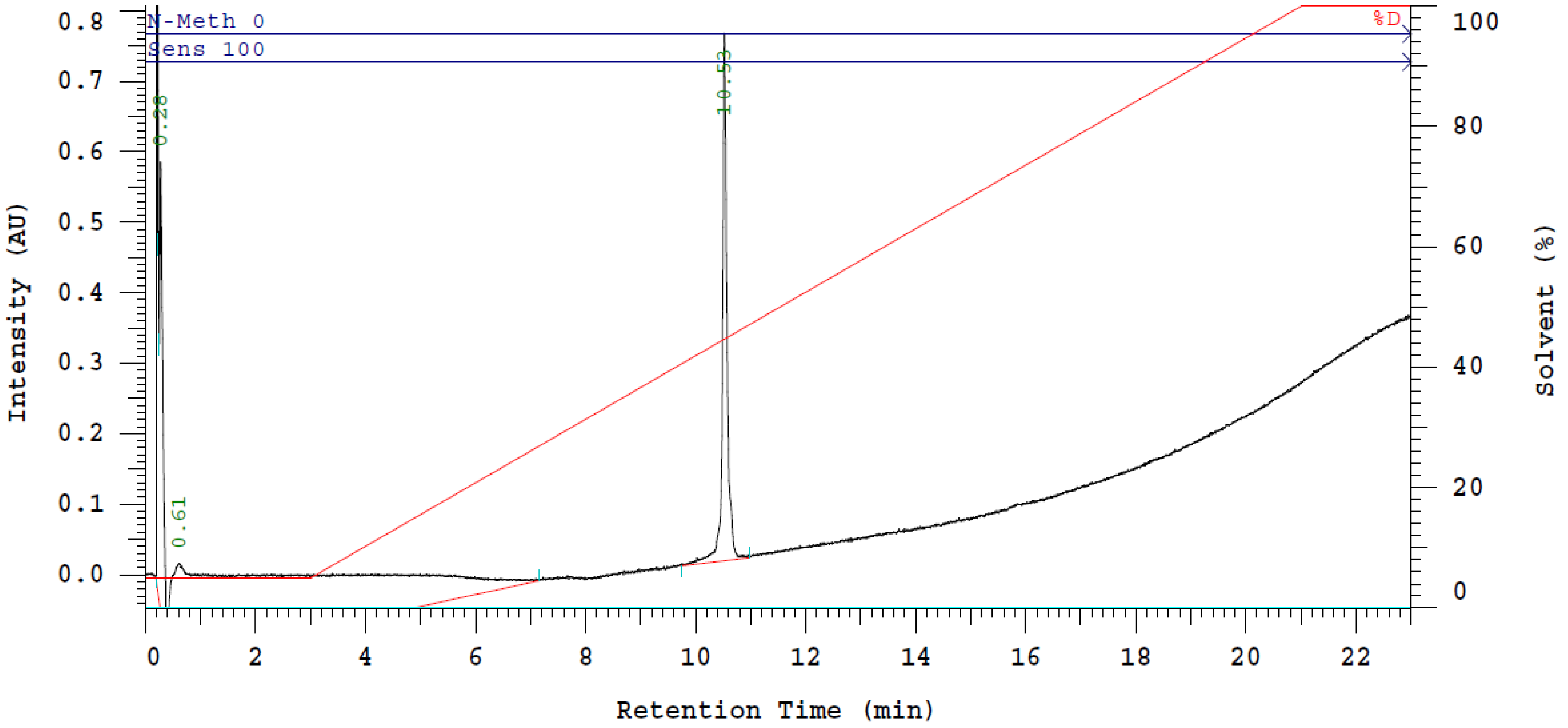

Analytical HPLC of purified **22**.

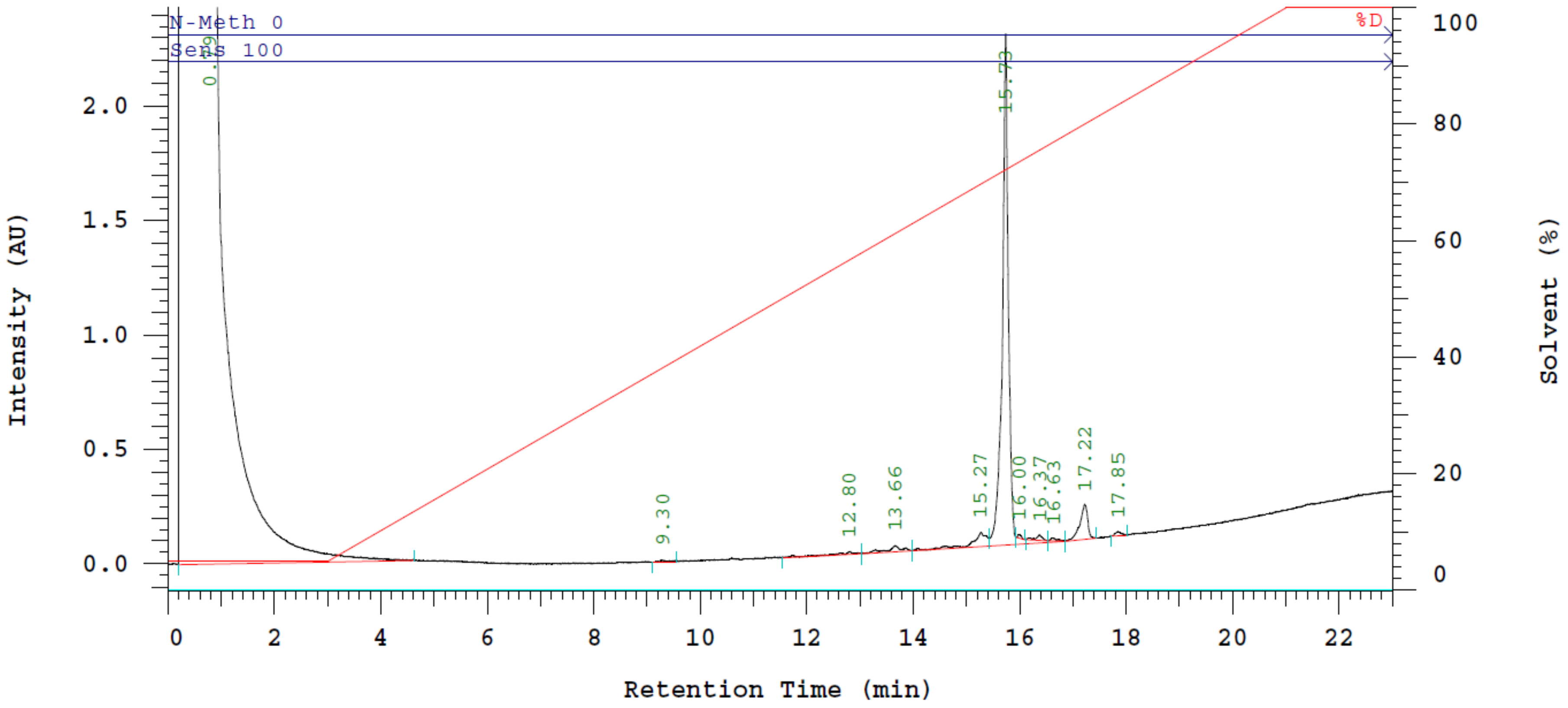

Analytical HPLC of crude **25**.

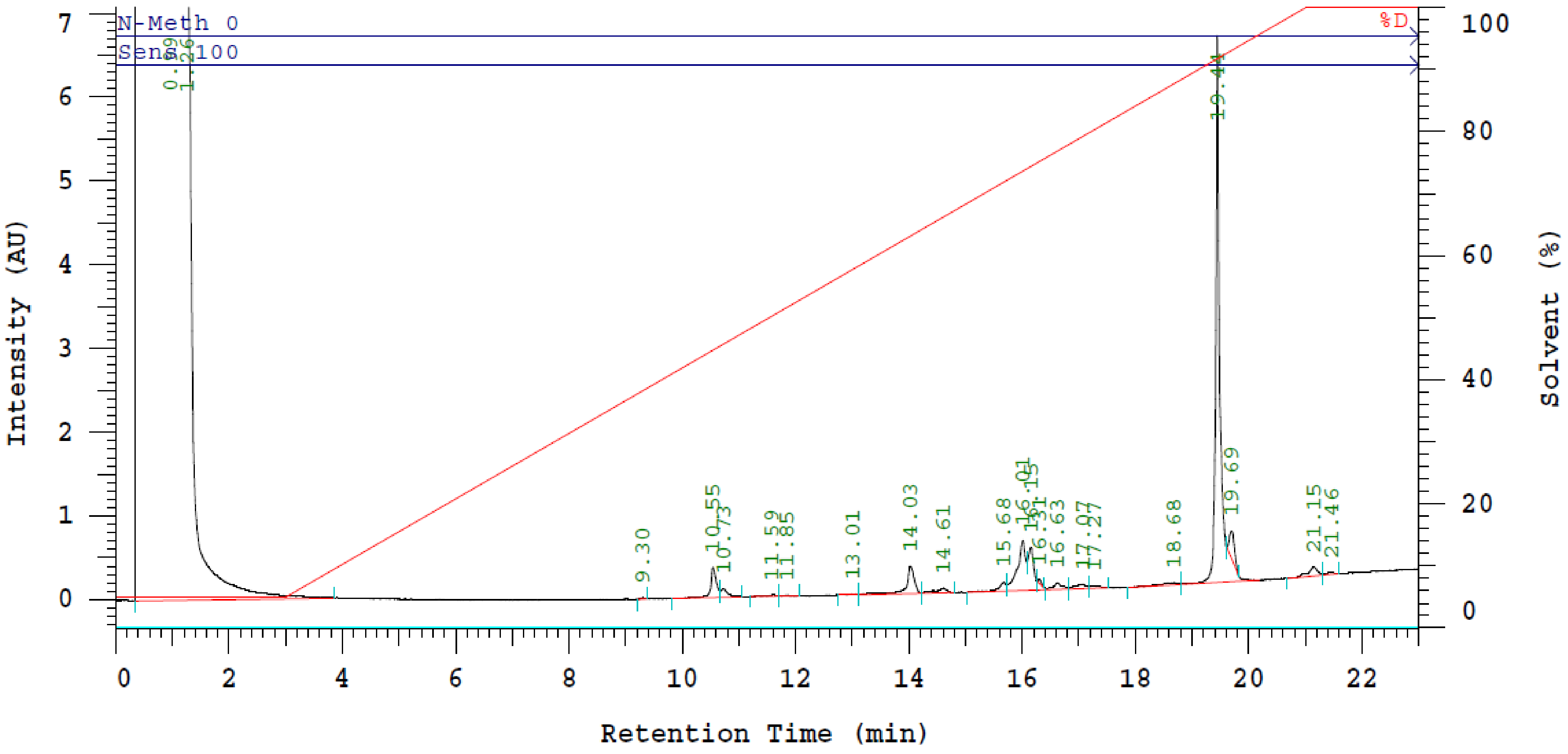

Analytical HPLC of crude **26**.

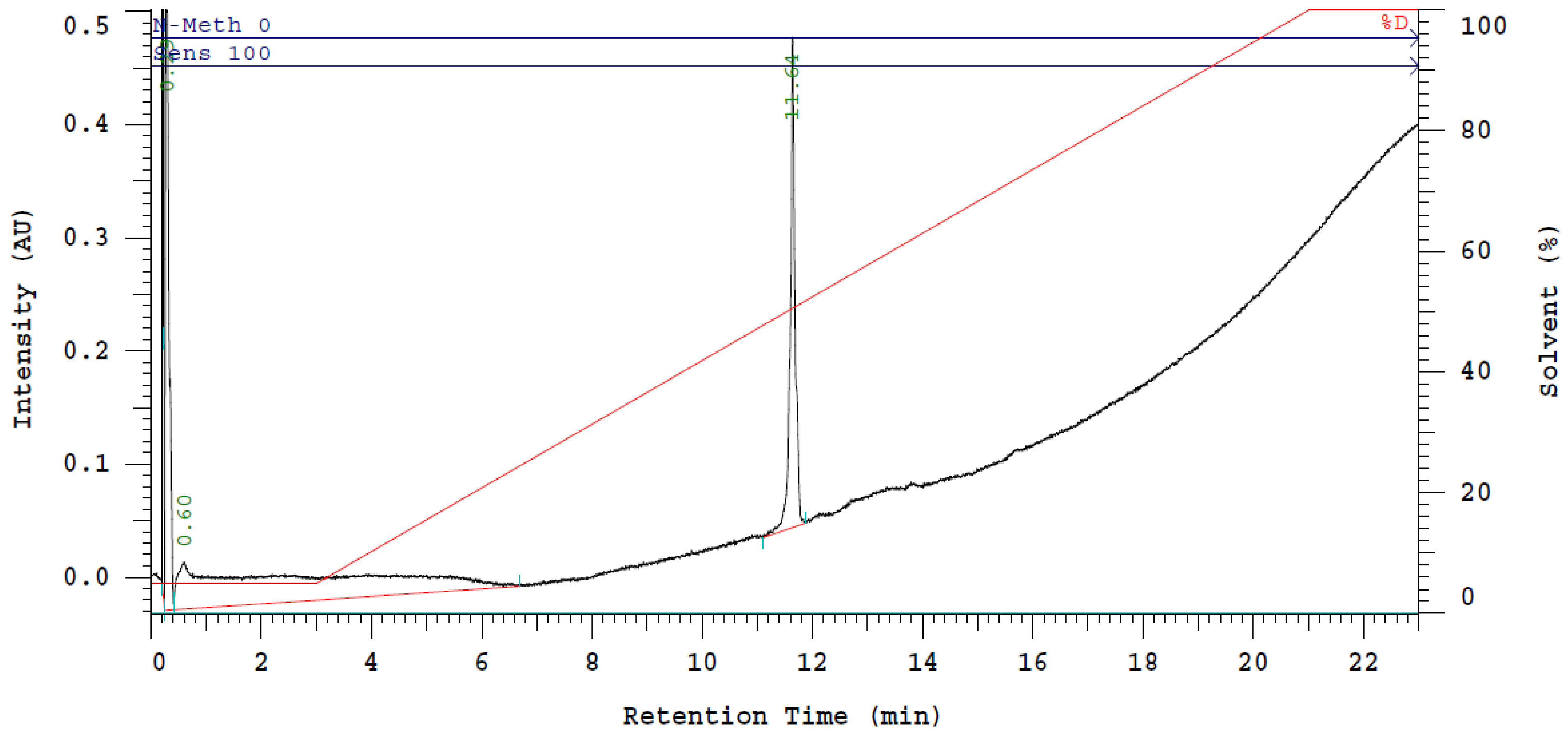

Analytical HPLC of purified **27**.

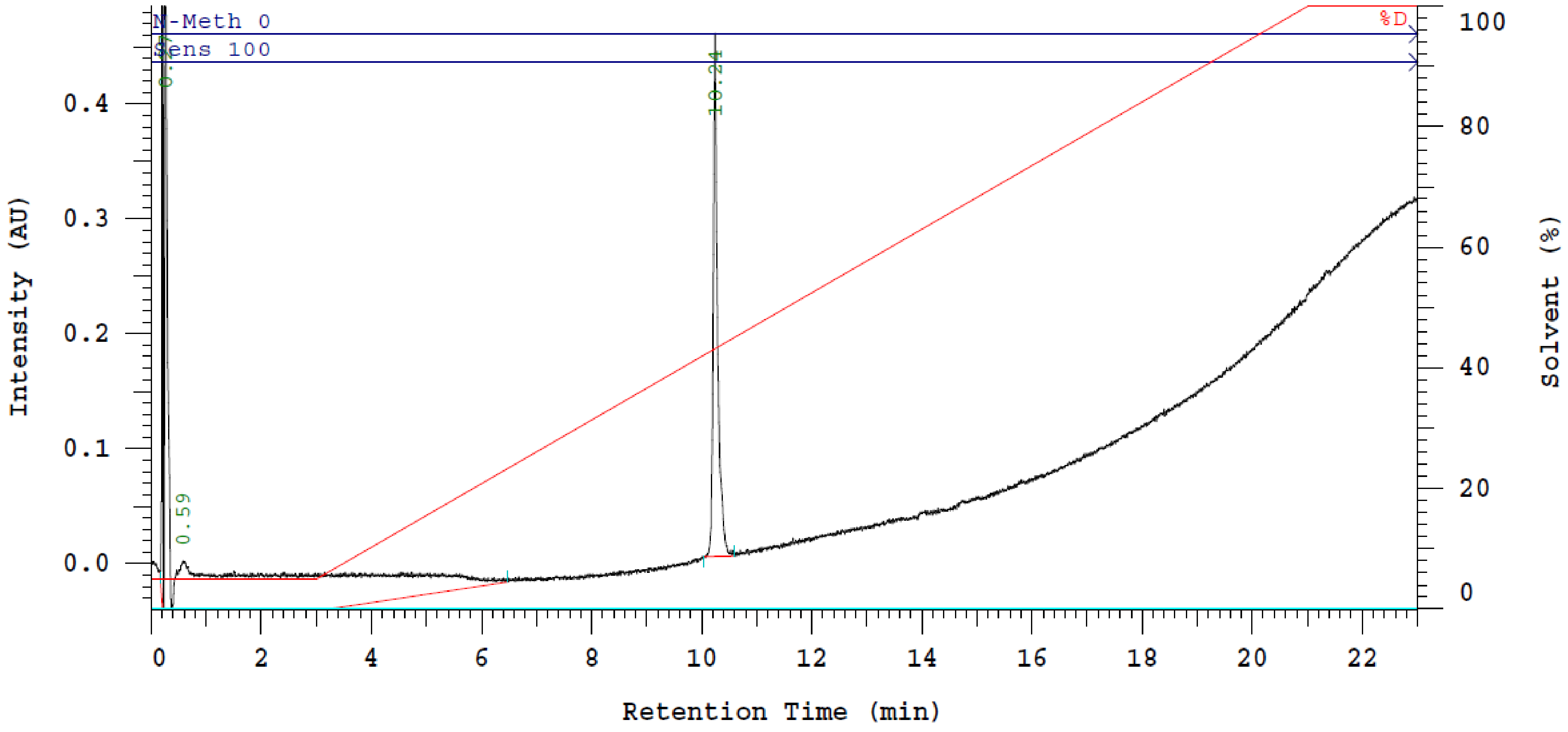

Analytical HPLC of purified **28**.

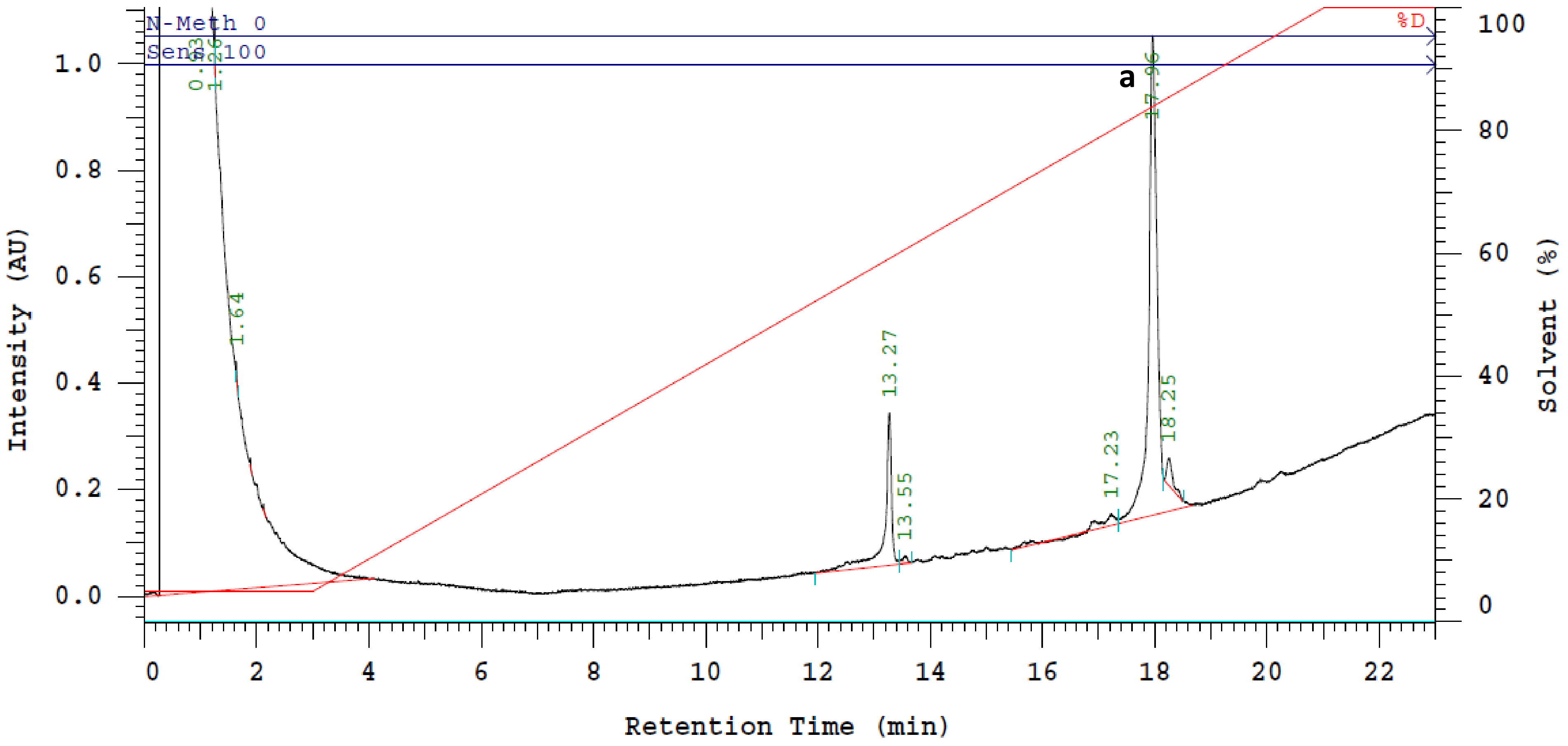

Analytical HPLC of **30** after Dde removal and trial cleavage from resin. Peak **a** showed the expected mass.

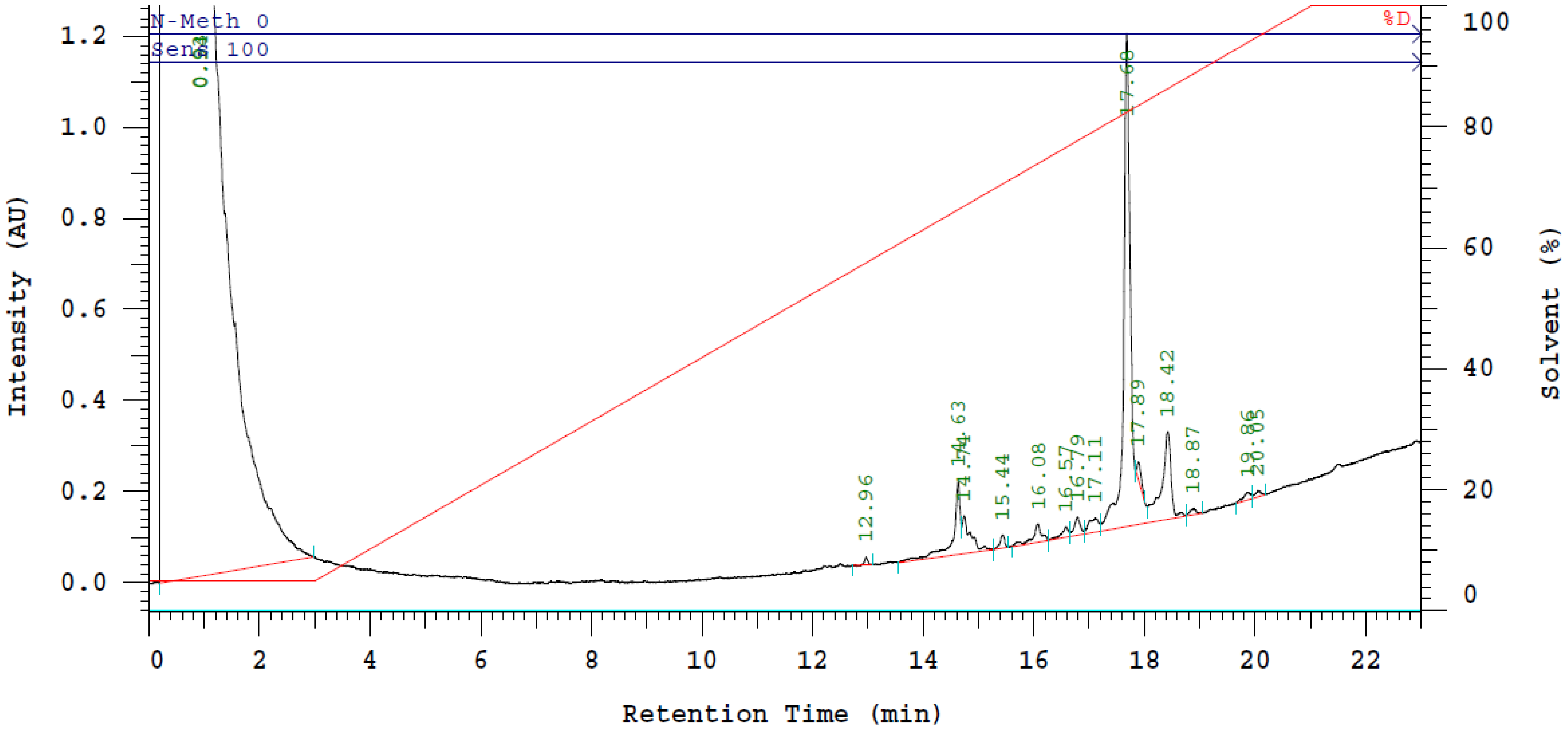

Analytical HPLC of crude linear **32**.

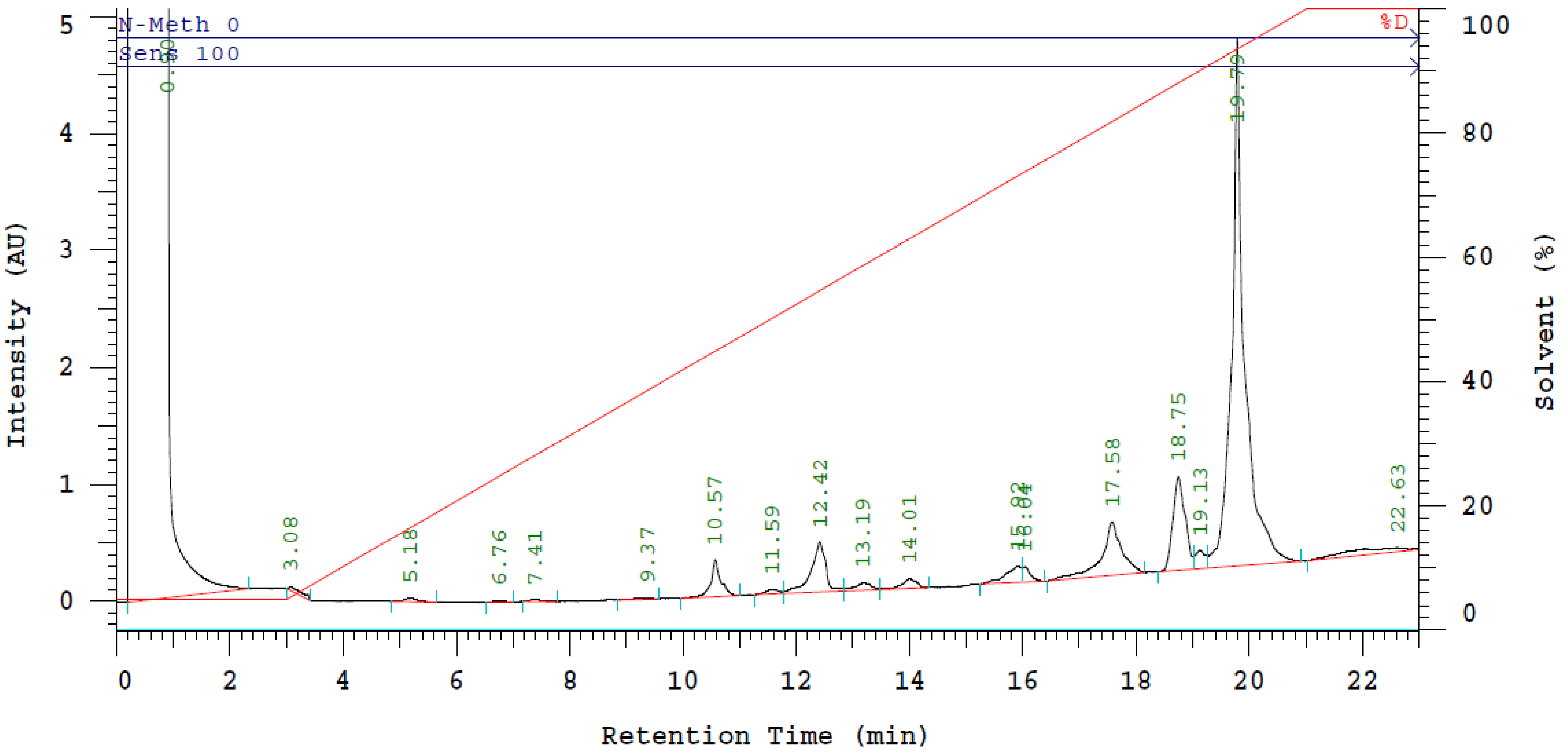

Analytical HPLC of crude cyclized **32**.

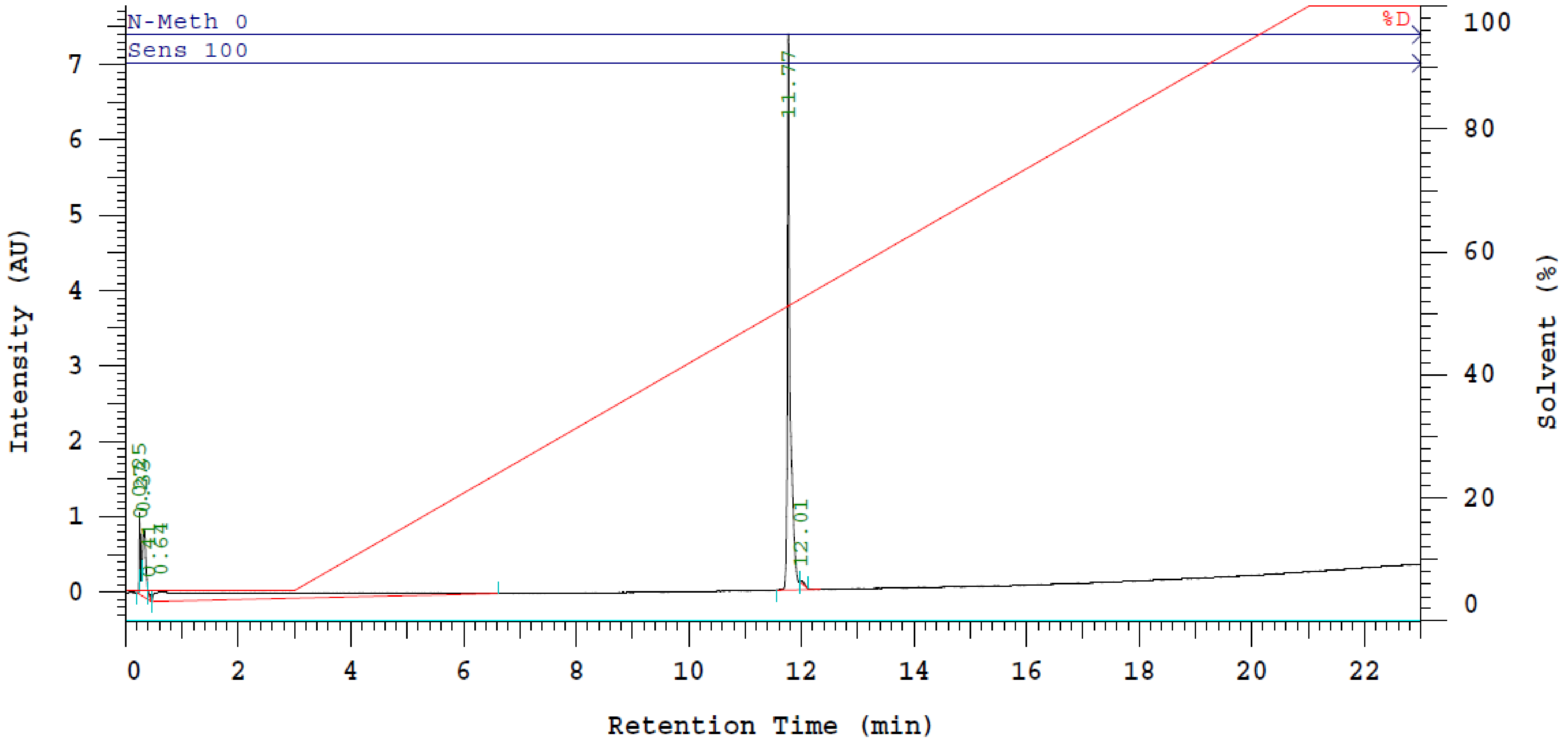

Analytical HPLC of purified **33**.

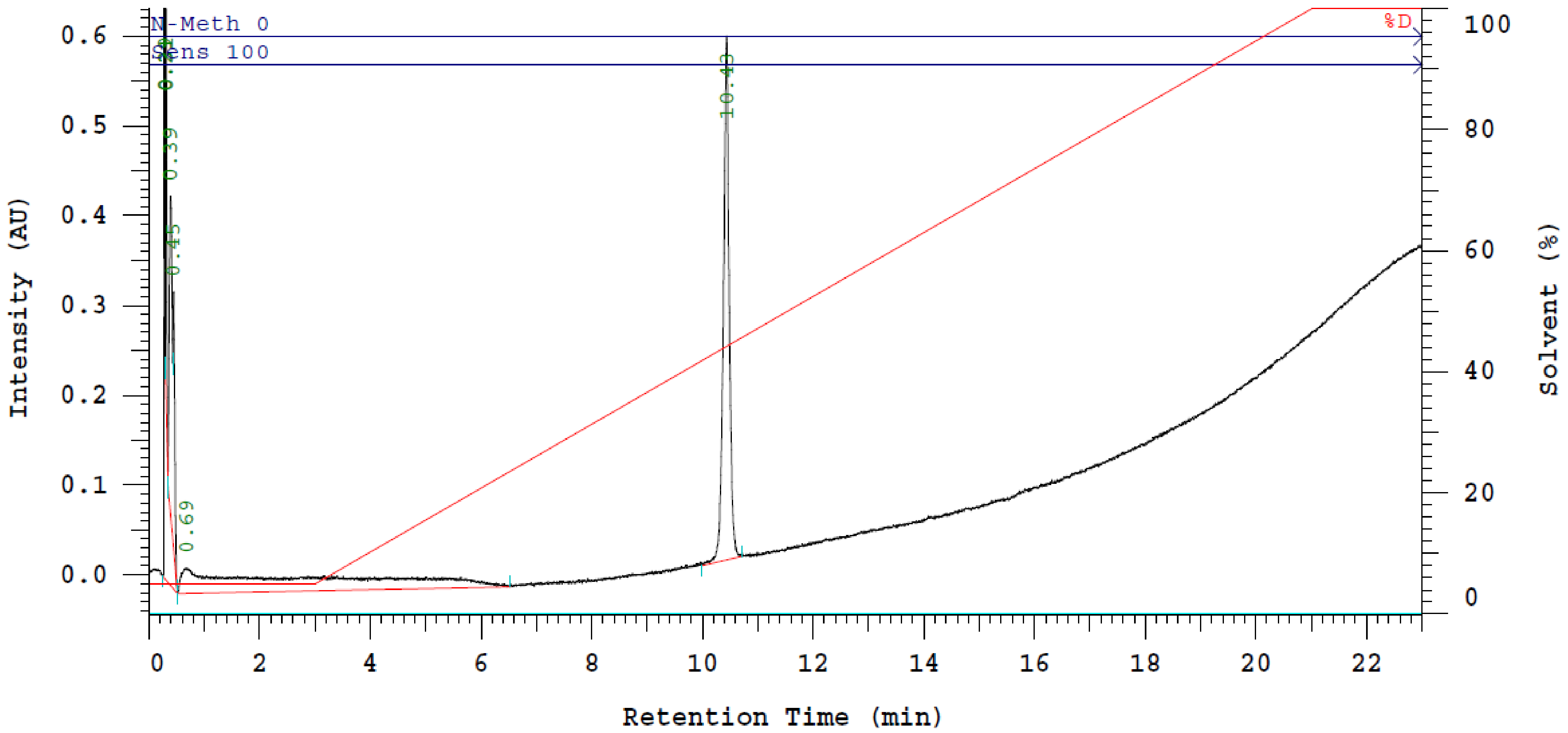

Analytical HPLC of purified **34**.

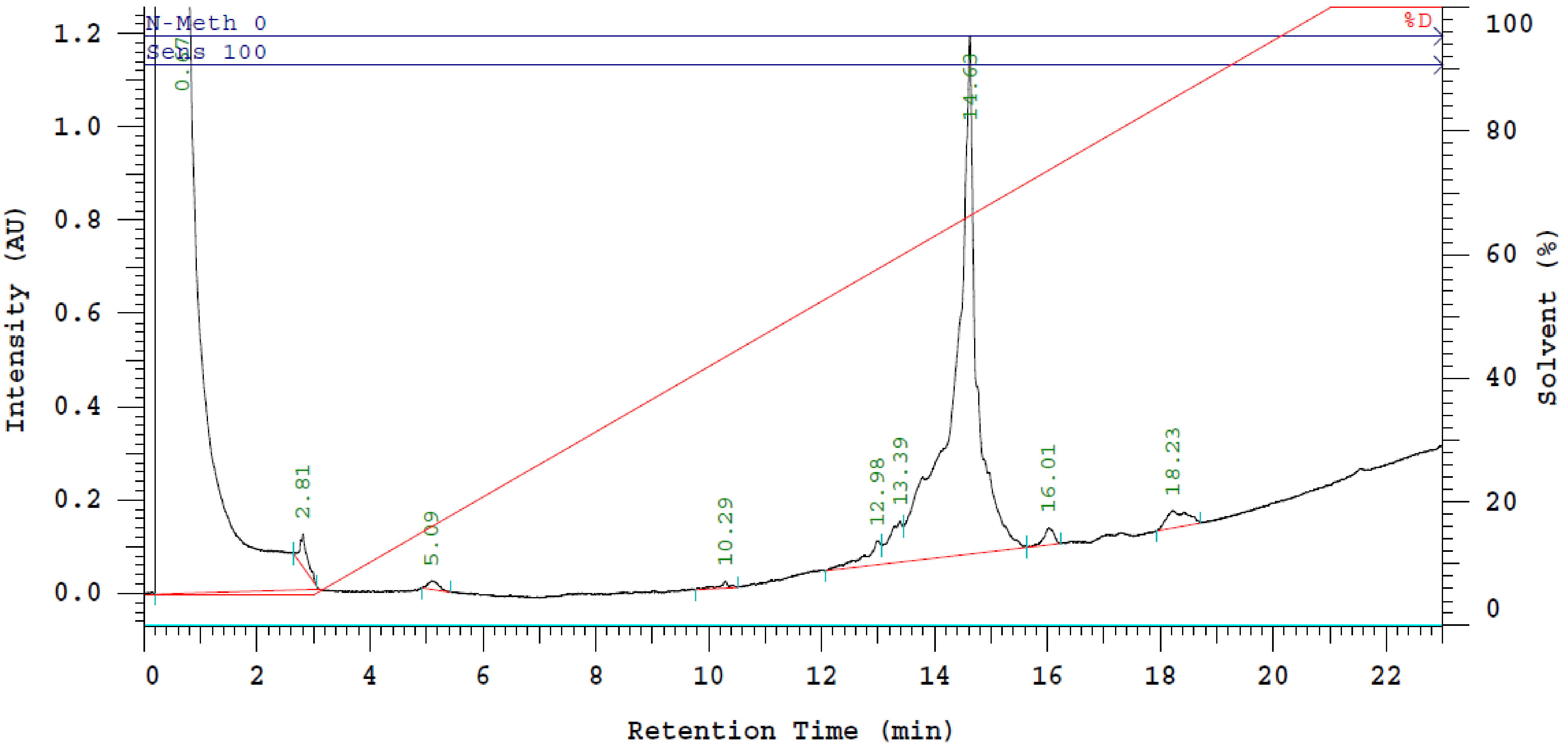

Analytical HPLC of crude **36** after Dde removal and trial cleavage from resin.

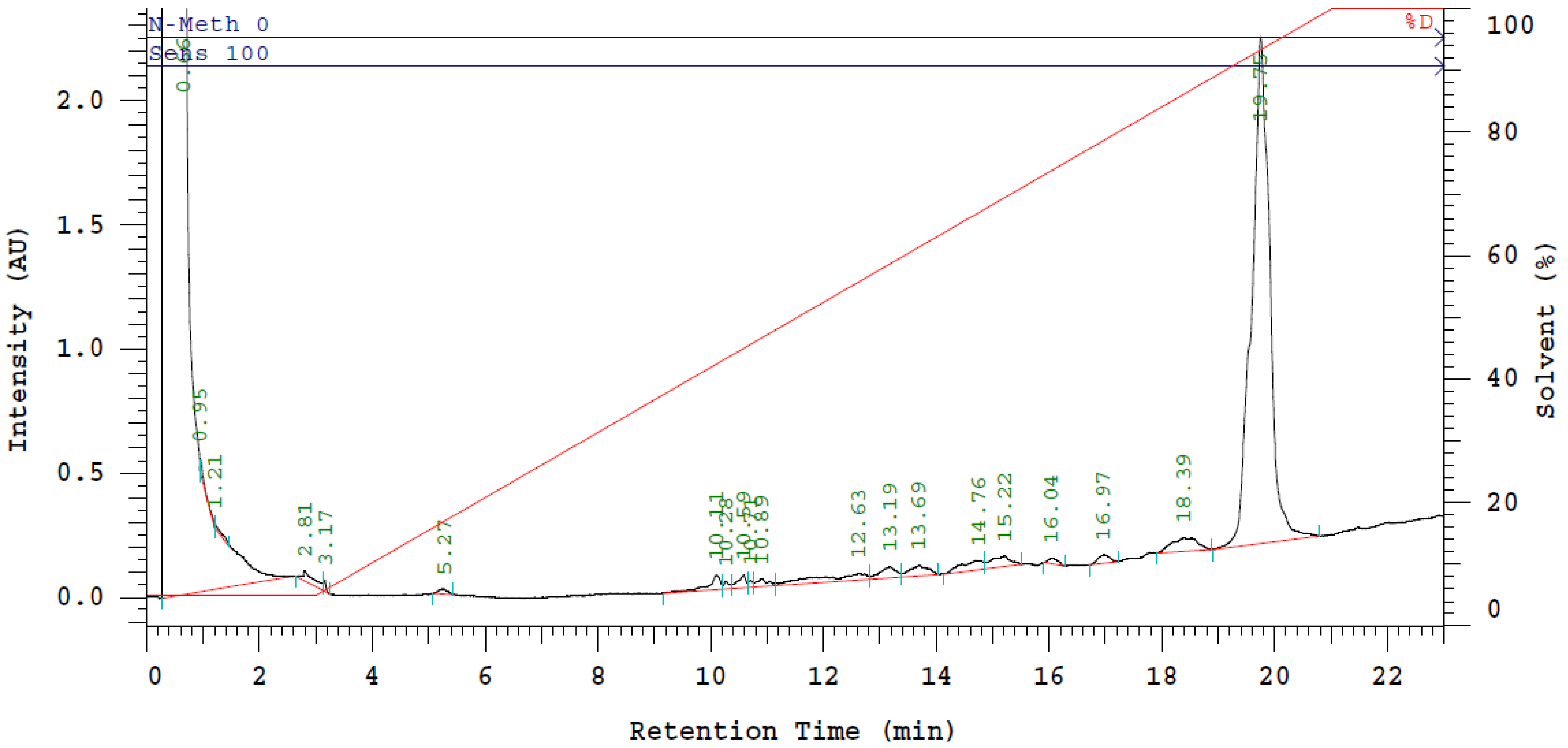

Analytical HPLC of crude **37** after trial cleavage of a small amount from the resin.

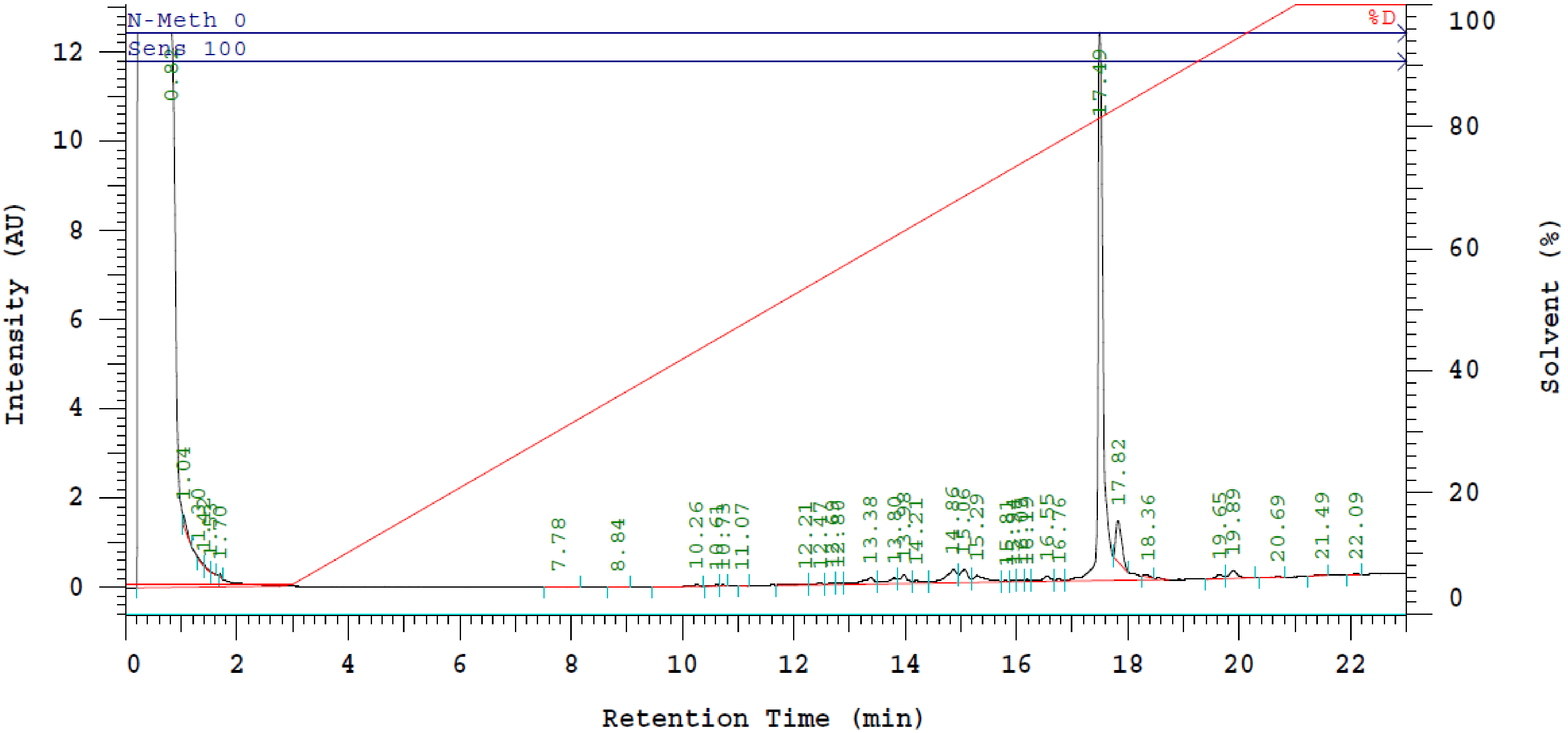

Analytical HPLC of crude linear **38**.

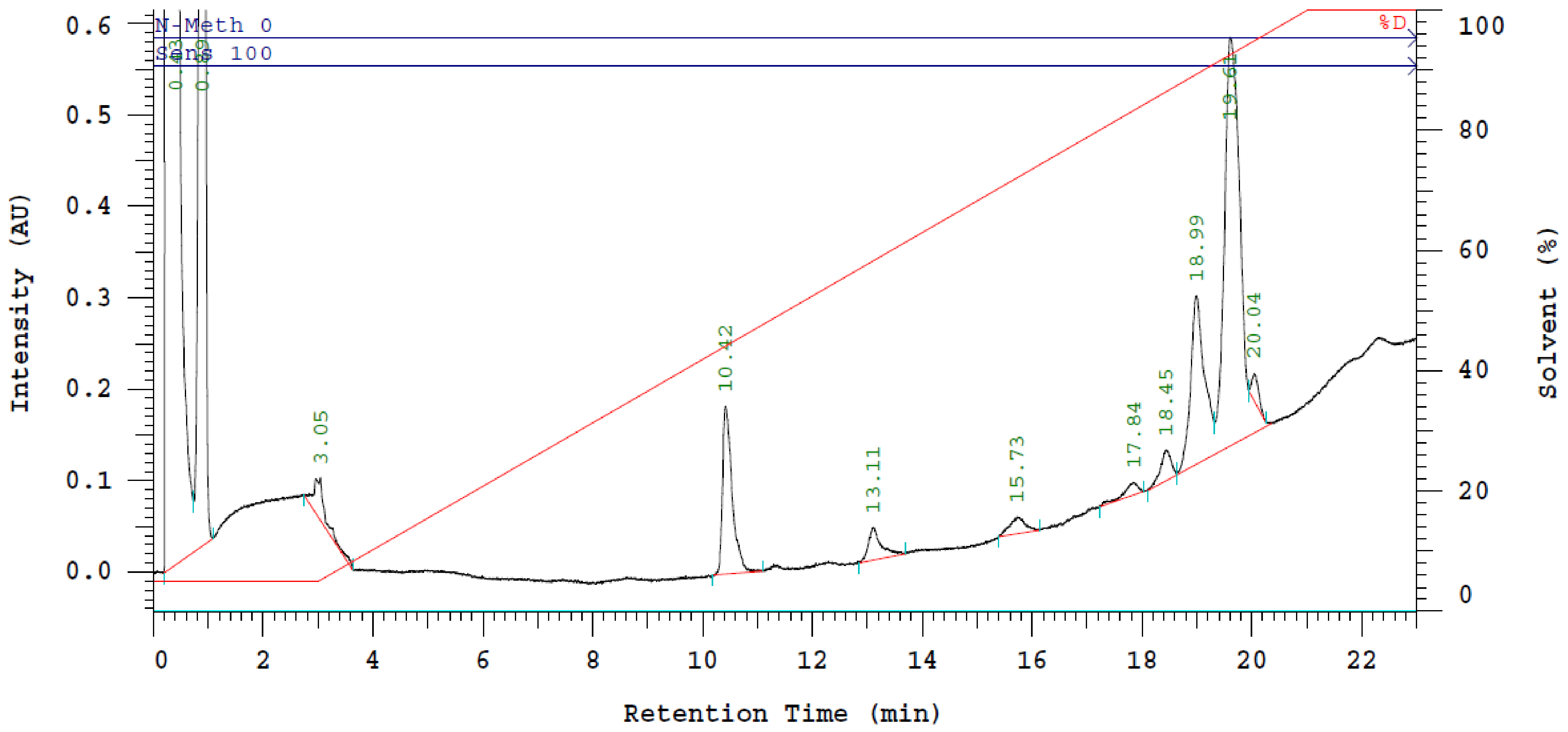

Analytical HPLC of crude cyclized **38**.

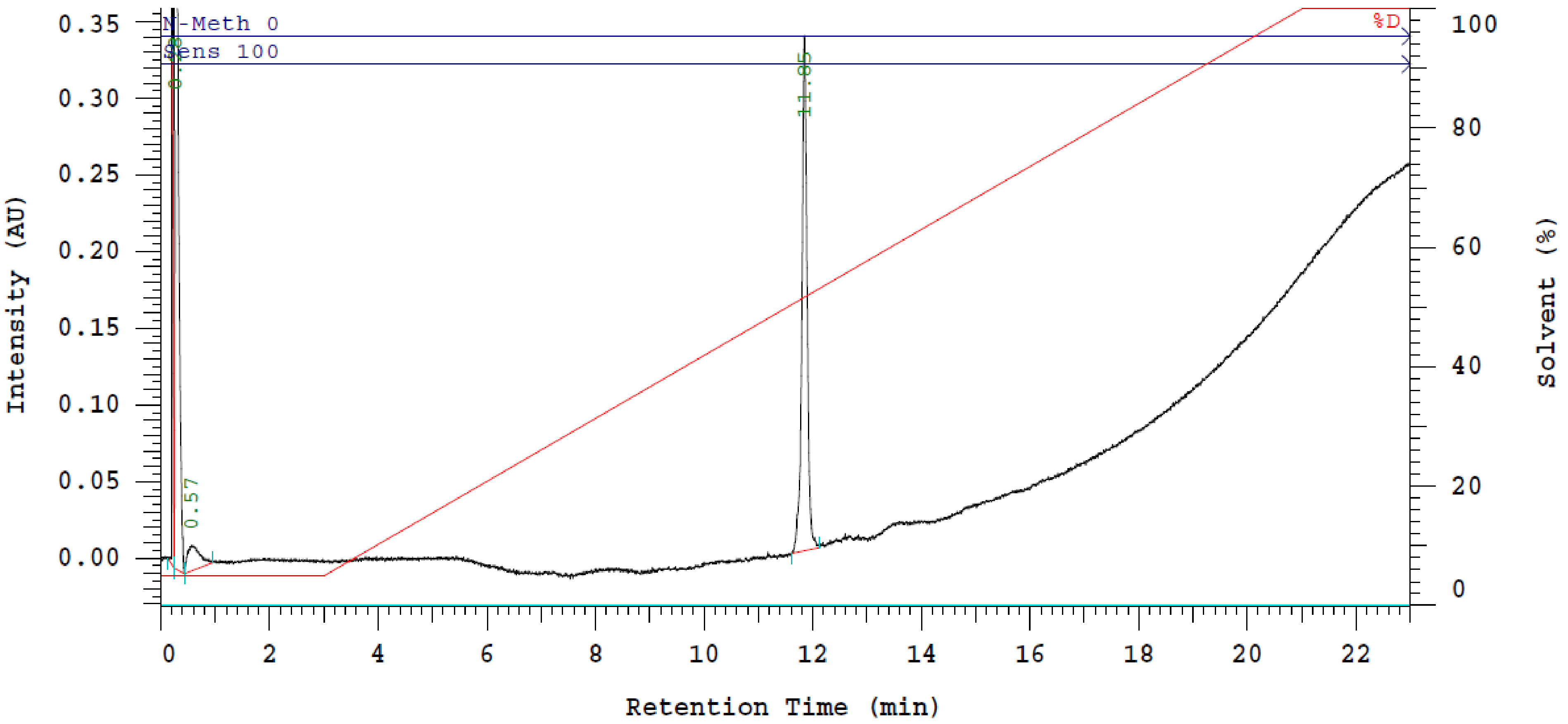

Analytical HPLC of purified **39**.

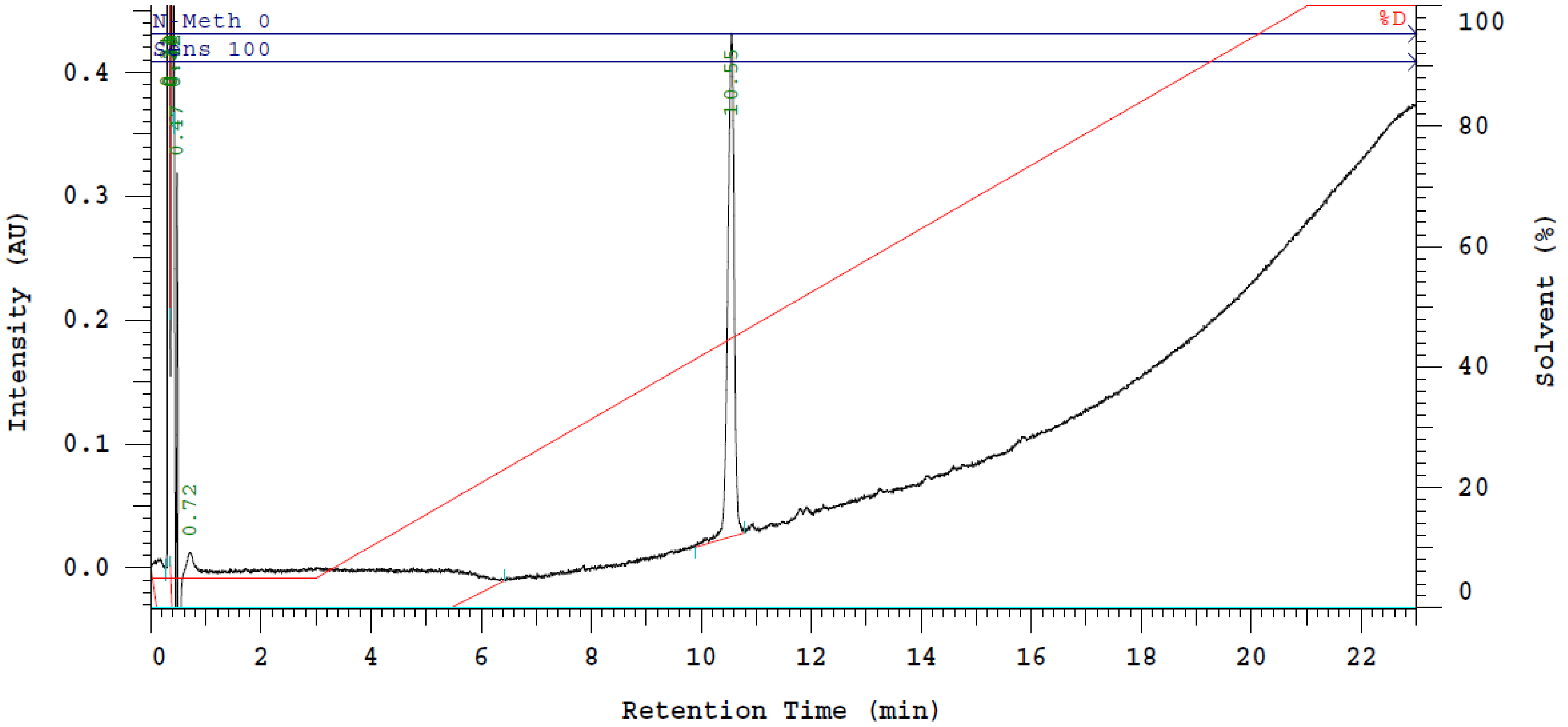

Analytical HPLC of purified **40**.

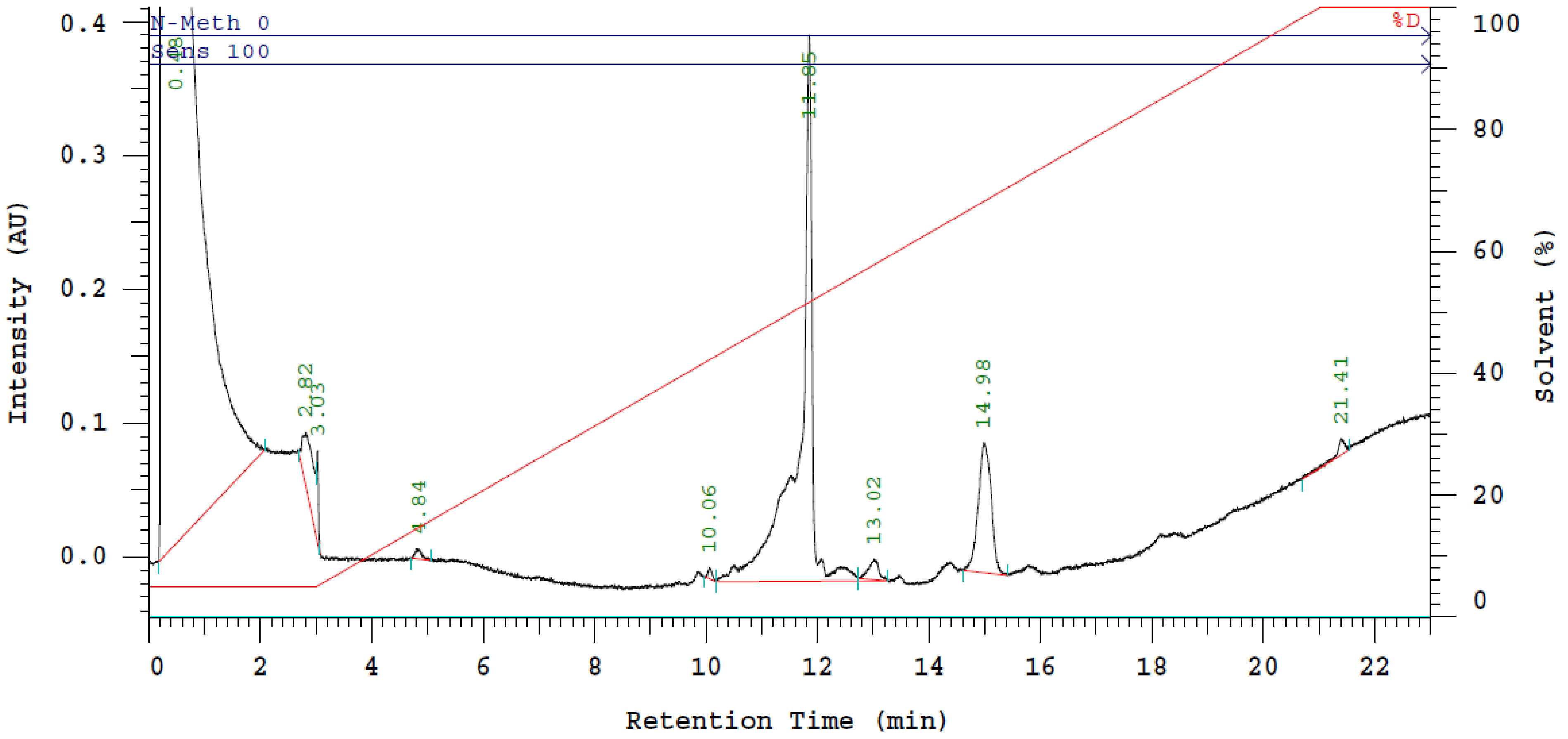

Analytical HPLC of crude **42** after removal of the Dde group and trial cleavage of a small amount from resin.

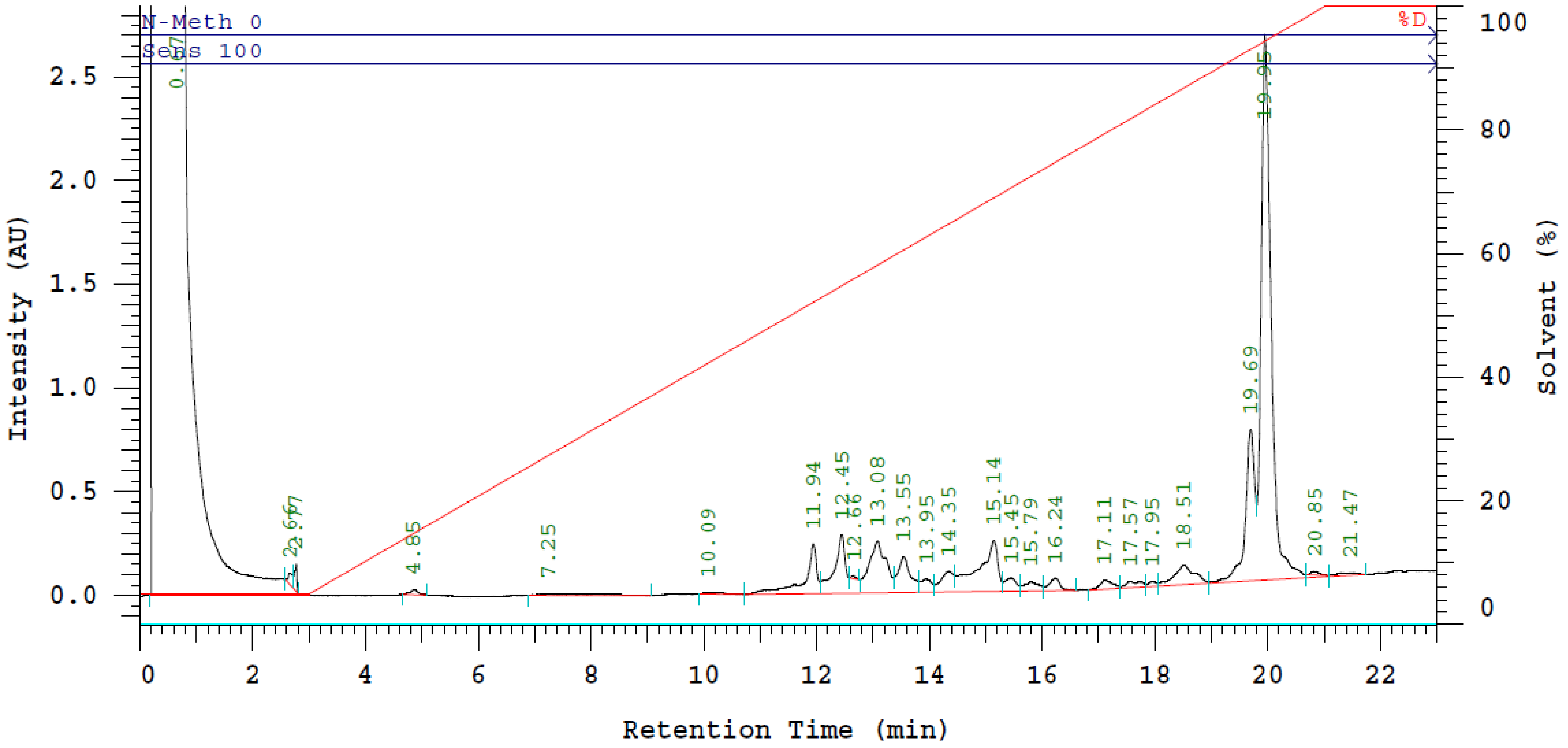

Analytical HPLC of crude **43** after trial cleavage of a small amount from the resin.

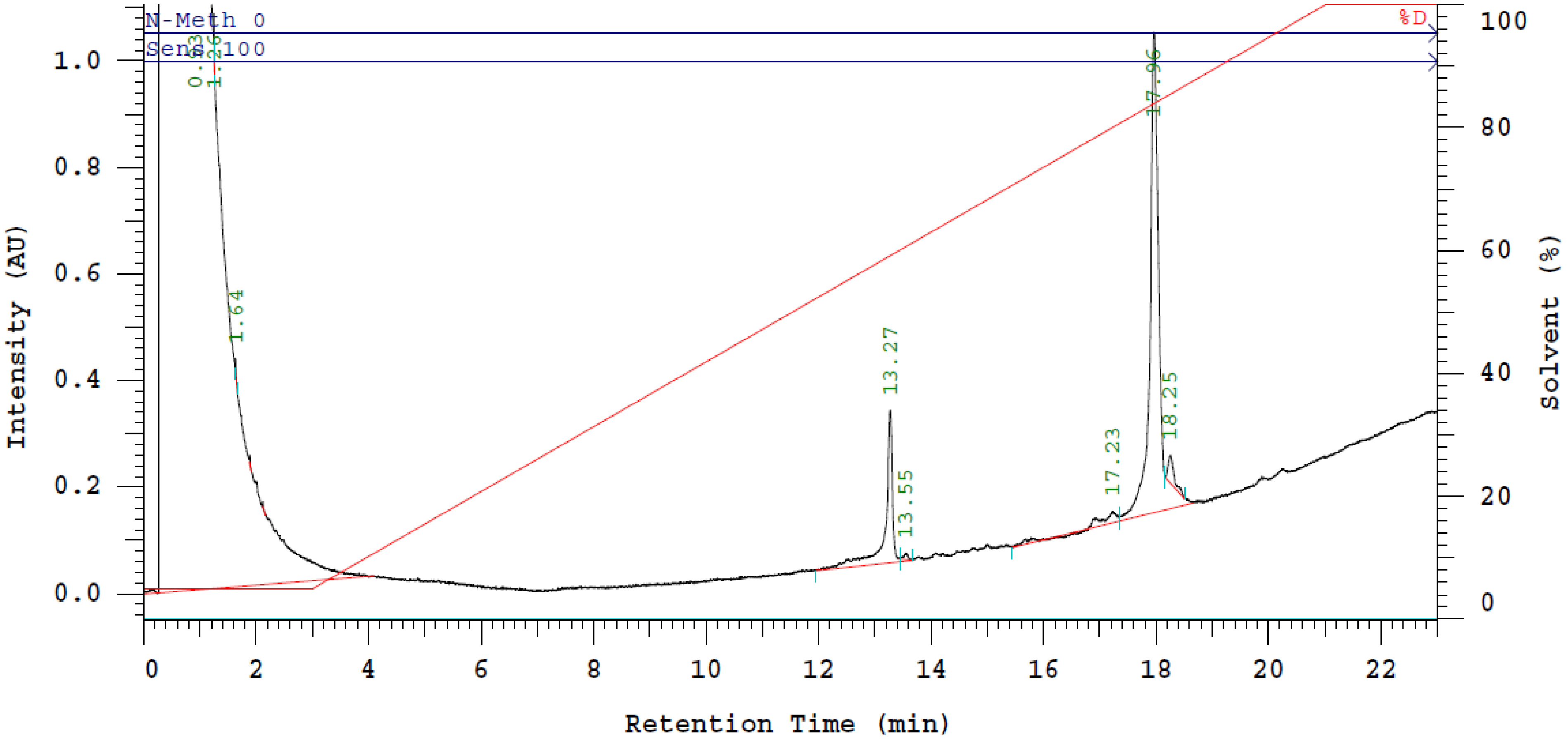

Analytical HPLC of crude linear **44**.

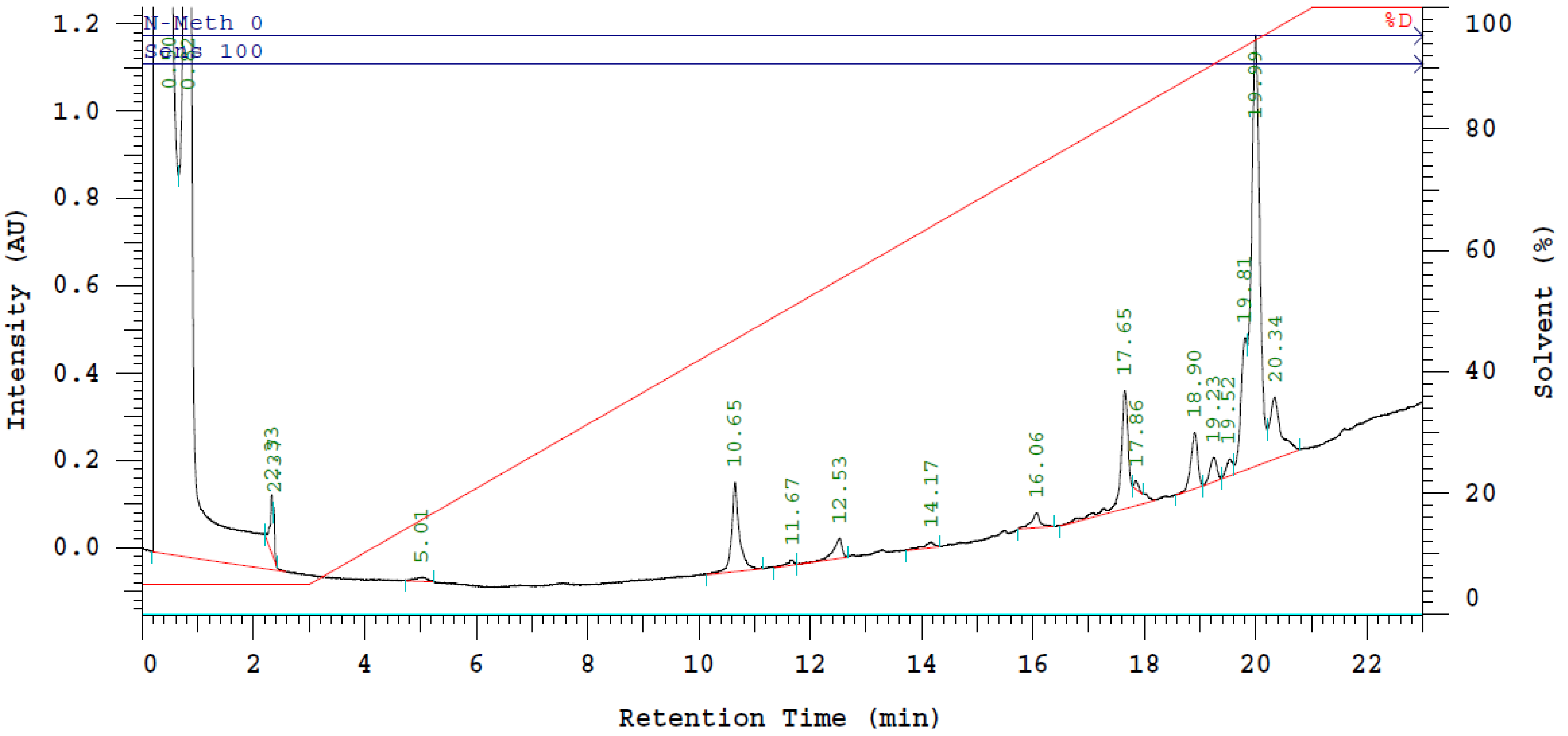

Analytical HPLC of crude cyclized **44**.

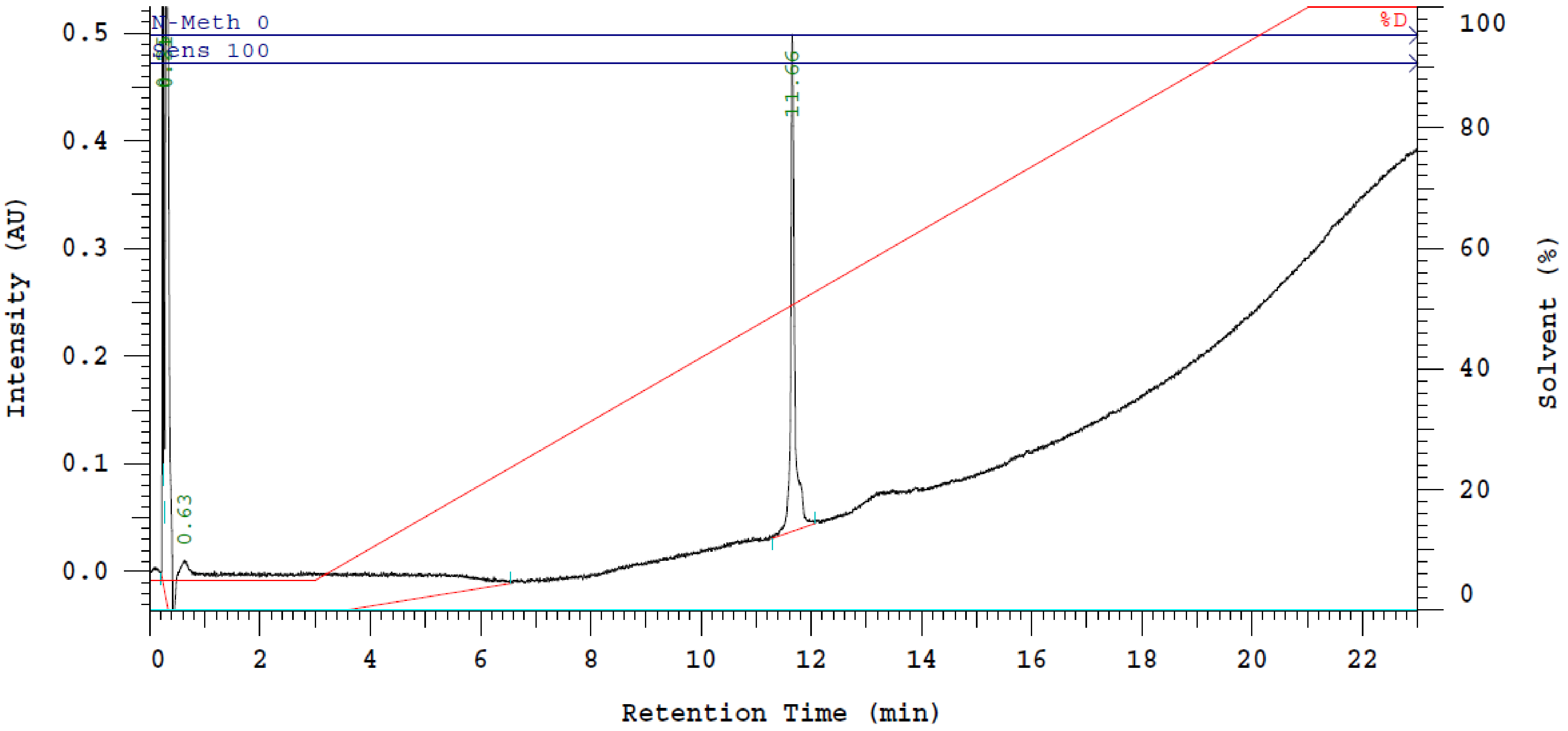

Analytical HPLC of purified **45**.

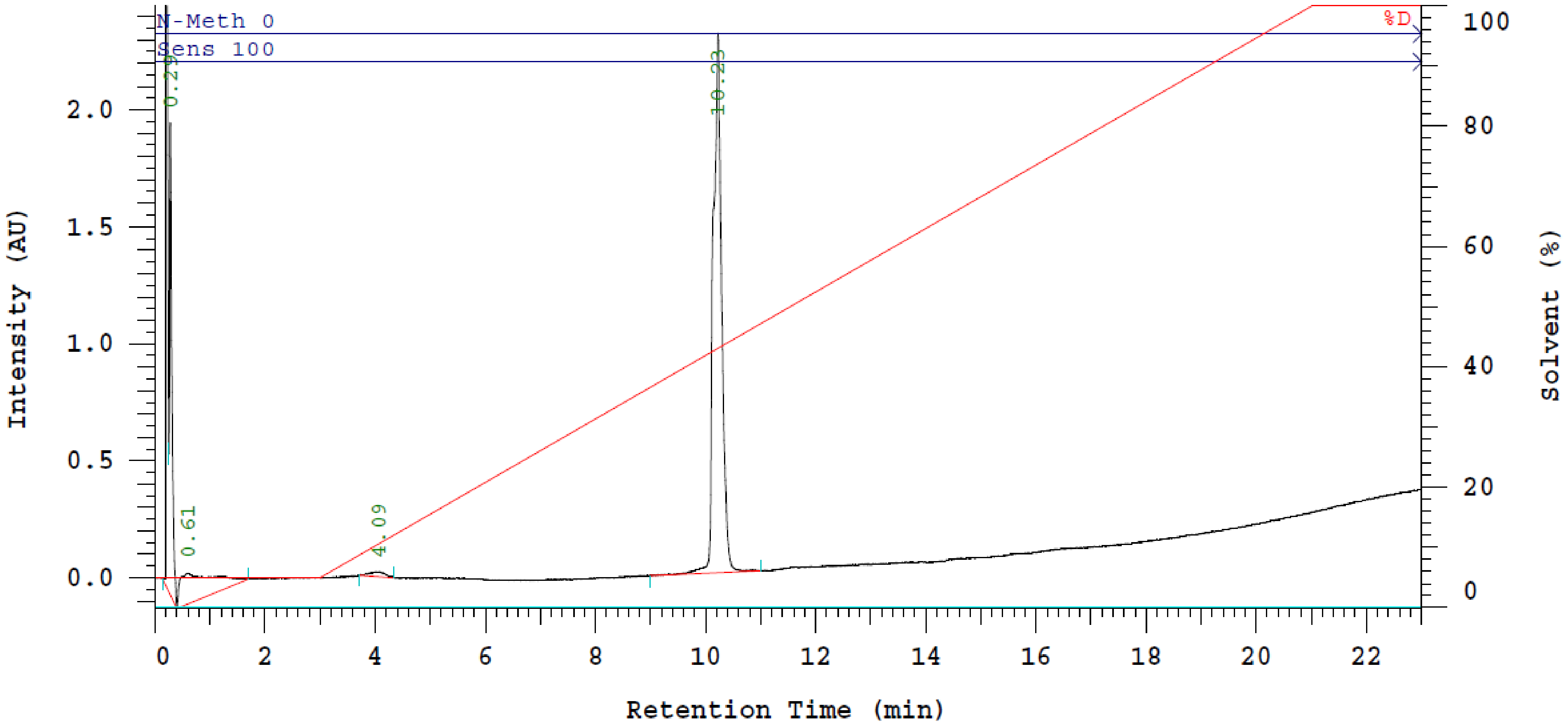

Analytical HPLC of purified **46**.

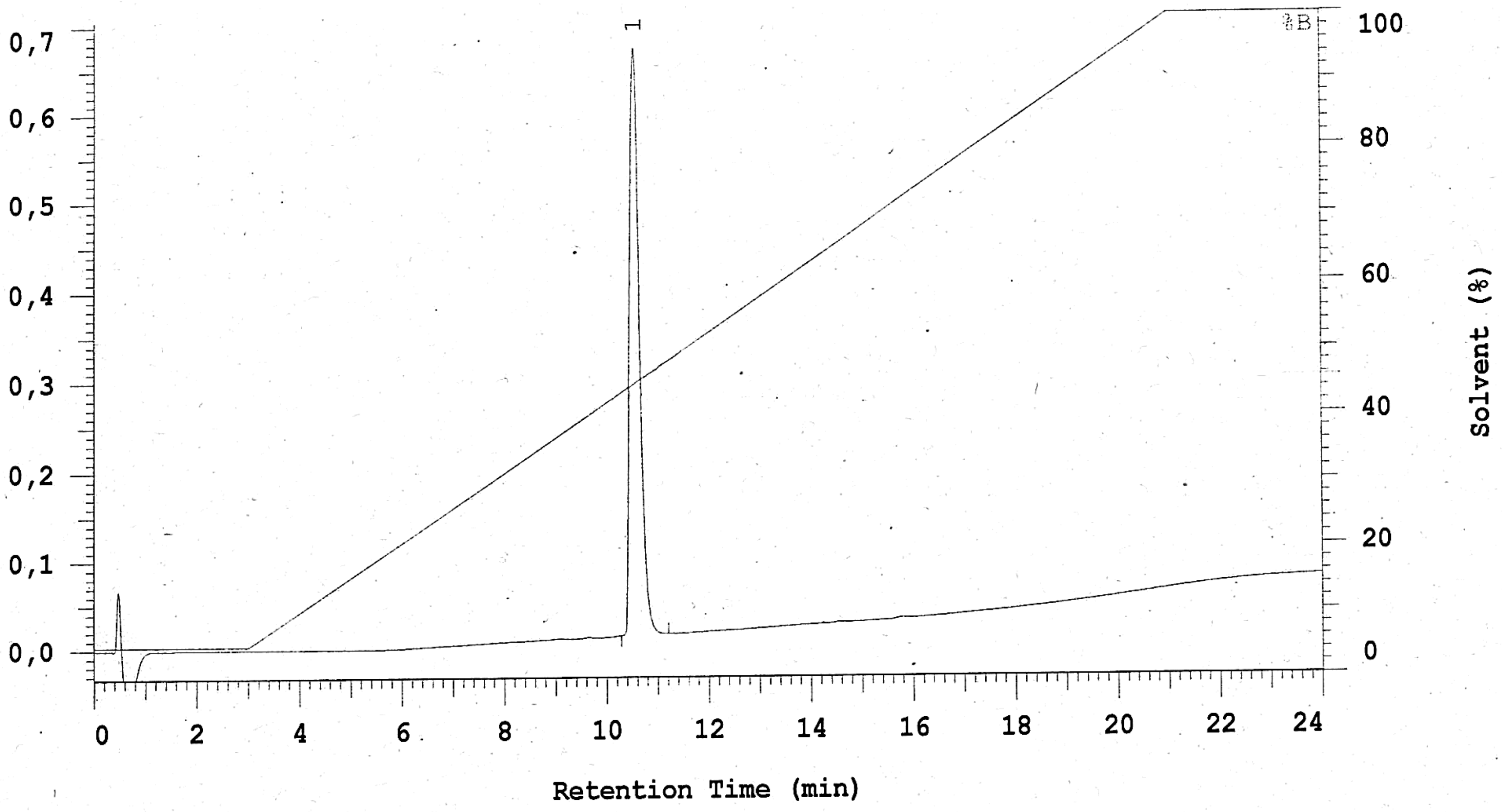

Analytical HPLC of purified **50**.

2. NMR Data

Table S1. NMR data of colistin B (**1**) in D_2O**.**

Referenced to D_2O δ_H 4.79 and TFA δ_{CF3} 116.6 ppm.

*very close carbon shift values (36.5 – 36.7 ppm and 28.5 – 28.9 ppm); see figure S23. Correlations cannot be distinguished more detailed for $CH_3CH_2NH_2$-group of 1-DAB, 3-DAB, 9-DAB).

**very close carbon shift values (24.6 - 24.7 ppm); see figure S22. Correlations cannot be distinguished more detailed for $CH(CH_3)_2$-group of 6-Leu and 7-Leu.

Colistin B (**1**)

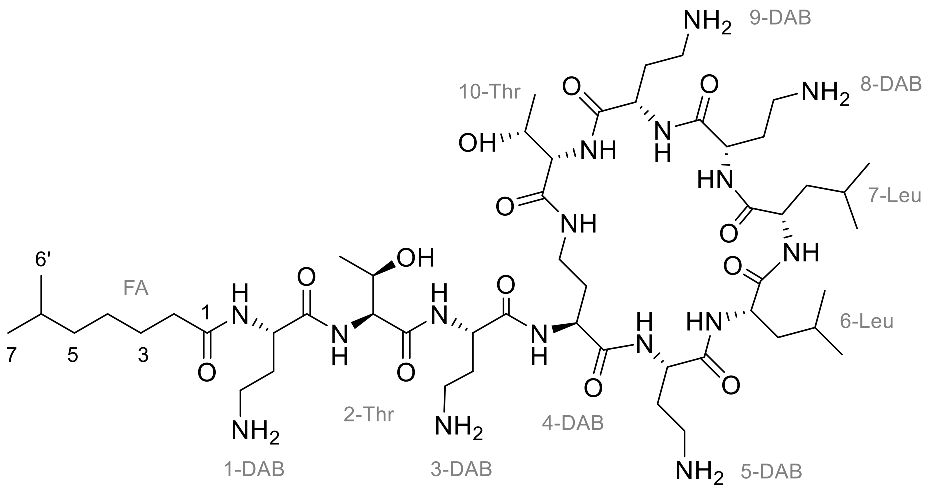

position	δ_C, mult.	δ_H (*J* in Hz)	HMBC correlations
FA			
1	178.1, C	-	-
2	35.7, CH_2	2.34 *pt* (7.2)	25.8, 26.4, 178.1
3	25.8, CH_2	1.60 *m*	26.4, 35.7, 38.1, 178.1
4	26.4, CH_2	1.31 *m*	25.8, 27.4, 35.7, 38.1
5	38.1, CH_2	1.19 *m*	22.2, 25.8, 27.4
6	27.4, CH_2	1.53 *m*	22.2, 26.4, 38.1
6‘	22.2, CH_3	0.85 *d* (6.6)	22.2, 27.4, 38.1
7	22.2, CH_3	0.85 *d* (6.6)	22.2, 27.4, 38.1
1-DAB			
1	173.3, C	-	-
2	51.4, CH	4.51 *m*	28.7*,36.6*, 173.3, 178.1
3	28.7*, CH_2	2.22, 2.10 *m*	36.6*, 51.4, 173.3
4	36.6*, CH_2	3.11 *m*	28.7*, 51.4
2-Thr			
1	172.1, C	-	-
2	59.2, CH	4.37 *d* (4.4)	19.0, 67.3, 172.1, 173.3
3	67.3, CH	4.25 *m*	19.0, 59.2, 172.1
4	19.0, CH_3	1.22 *d* (6.5)	59.2, 67.3
3-DAB			

1	172.6, C	-	-
2	51.4, CH	4.51 *m*	28.7*,36.6*, 172.1, 172.6
3	28.7*, CH_2	2.22, 2.10 *m*	36.6*, 51.4, 172.6
4	36.6*, CH_2	3.11 *m*	28.7*, 51.4
4-DAB			
1	173.1, C	-	-
2	52.1, CH	4.31 *m*	30.9, 36.4
3	30.9, CH_2	2.00, 1.90 *m*	36.4, 52.1, 173.1
4	36.4, CH_2	3.36, 3.21 *m*	30.9, 52.1, 171.7
5-DAB			
1	172.3, C	-	-
2	50.9, CH	4.58 *dd* (8.4, 3.8)	30.2, 36.4, 172.3, 173.1
3	30.2, CH_2	2.18, 2.06 *m*	36.4, 50.9, 172.3
4	36.4, CH_2	3.05 *m*	30.2, 50.9
6-Leu			
1	175.1, C	-	-
2	*53.2, CH*	4.30 *m*	*24.6**, 40.0, 172.3, 175.1*
3	*40.0,* CH_2	1.67, 1.58 *m*	*21.0, 22.2, 24.6**, 53.2, 175.1*
4	*24.6**, CH*	1.61 *m*	*21.0, 22.2, 40.0, 53.2*
4a	*22.2,* CH_3	0.94 *d* (6.2)	*21.0, 24.6**, 40.0, 53.2*
5	*21.0,* CH_3	0.90 *d* (6.2)	*22.2, 24.6**, 40.0, 53.2*
7-Leu			
1	175.3, C	-	-
2	*52.1, CH*	4.42 *dd* (11.2, 4.3)	*24.6**, 39.4, 175.1, 175.3*
3	*39.4,* CH_2	1.69 *m*	*20.3, 22.7, 24.6**, 52.1, 175.3*
4	*24.6**, CH*	1.61 *m*	*20.3, 22.7, 39.4, 52.1*
4a	*22.7,* CH_3	0.95 *d* (6.2)	*20.3, 24.6**, 39.4*
5	*20.3,* CH_3	0.88 *d* (6.2)	*22.7, 24.6**, 39.4*
8-DAB			
1	173.5, *C*	-	-
2	52.1, CH	4.32 *m*	28.1, 36.8, 173.5, 175.3
3	28.1, CH_2	2.30, 2.20 *m*	36.8, 52.1, 173.5
4	36.8, CH_2	3.15 *m*	28.1, 52.1
9-DAB			
1	*172.9, C*	-	-
2	53.1, CH	4.29 *m*	28.7*,36.6*, 172.9, 173.5
3	28.7*, CH_2	2.27, 2.20 *m*	36.6*, 53.1, 172.9
4	36.6*, CH_2	3.11 *m*	28.7*, 53.1
10-Thr			
1	171.7, C	-	-
2	59.7, CH	4.22 *d* (4.7)	19.4, 66.7, 172.7, 172.9
3	66.7, CH	4.26 *m*	19.4, 59.7, 172.7
4	19.4, CH_3	1.22 *d* (6.5)	59.7, 66.7

Table S2. NMR data of fragment of **22** in D_2O**.**

Referenced to D_2O δ_H 4.79 and FA-Me δ_{CH3} 22.2 ppm based on colistin B (**1**).

Conjugate **22**

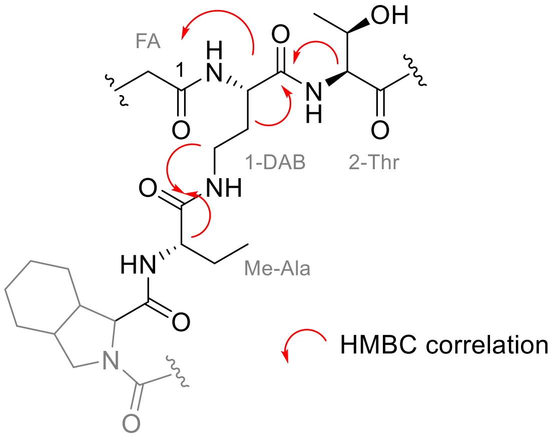

	Colistin B (**1**)		Conjugate **22** (1-DAB bound)	
position	δ_C, mult.	δ_H (*J* in Hz)	δ_C, mult.	δ_H (*J* in Hz)
FA				
1	178.1, C	-	177.8, C	-
2	35.7, CH_2	2.34 *pt (7.2)*	35.7, CH_2	2.31 *m*
…	…	…	…	…
1-DAB				
1	173.3, C	-	174.3, C	-
2	51.4, CH	4.51 *m*	51.9, CH	4.33 *m*
3	28.7, CH_2	2.22, 2.10 *m*	30.7, CH_2	2.06, 1.88 *m*
4	36.6, CH_2	3.11 *m*	36.1, CH_2	3.25, 3.39 *m*
2-Thr				
1	172.1, C	-	172.2, C	-
2	59.2, CH	4.37 d (4.4)	59.4, CH	4.36 *m*
3	67.3, CH	4.25 *m*	67.3, CH	4.26 *m*
4	19.0, CH_3	1.22 *d* (6.5)	18.7, CH_3	1.23 *m*
	D-Ubi-ElaSub (**50**)		Conjugate **22** (1-DAB bound)	
Me-Ala				
1	176.4, C	-	174.3, C	-
2	55.0, CH	4.18, *d* (6.8)	56.1, CH	4.06, *d* (6.8)
3	24.4, CH_2	1.81, 1.66, *m*	24.7, CH_2	1.74, 1.63 *m*
4	9.7, CH_3	0.89 *m*	9.9, CH_3	0.89 *m*

Chemical shifts of 1-DAB and Me-Ala (see also Figure S18) show significant changes due to amide formation. For shifted signals of DAB, HMBC correlations to FA and 2-Thr corroborate attachment to 1-DAB.

Table S3. NMR data of fragment of **28** in D_2O.

Referenced to D_2O δ_H 4.79 and FA-Me δ_{CH3} 22.2 ppm based on colistin B (**1**).

Conjugate **28**

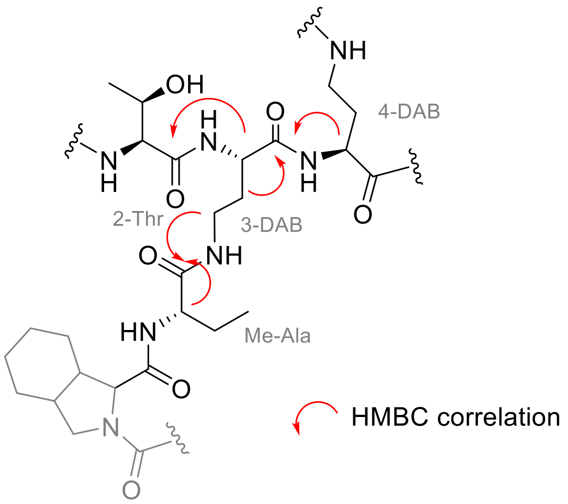

	Colistin B (**1**)		Conjugate **28** (3-DAB bound)	
position	δ_C, mult.	δ_H (*J* in Hz)	δ_C, mult.	δ_H (*J* in Hz)
2-Thr				
1	172.1, C	-	171.9, C	-
2	59.2, CH	4.37 *d* (4.4)	59.3, CH	4.36 *d* (4.2)
3	67.3, CH	4.25 *m*	67.1, CH	4.25 *m*
4	19.0, CH_3	1.22 *d* (6.5)	19.1, CH_3	1.22 *d* (6.4)
3-DAB				
1	172.6, C	-	172.8, C	-
2	51.4, CH	4.51 *m*	53.8, CH	4.26 *m*
3	28.7*, CH_2	2.22, 2.10 *m*	30.7, CH_2	2.06, 2.00 *m*
4	36.6*, CH_2	3.11 *m*	36.0, CH_2	3.24, 3.37 *m*
4-DAB				
1	173.1, C	-	173.3, C	-
2	52.1, CH	4.31 *m*	52.2, CH	4.28 *m*
3	30.9, CH_2	2.00, 1.90 *m*	30.9, CH_2	2.00, 1.90 *m*
4	36.4, CH_2	3.36, 3.21 *m*	36.3, CH_2	3.36, 3.21 *m*
	D-Ubi-ElaSub (**50**)		Conjugate **28** (3-DAB bound)	
Me-Ala				
1	176.4, C	-	174.4, C	-
2	55.0, CH	4.18, *d* (6.8)	55.9, CH	4.07, *d* (6.4)
3	24.4, CH_2	1.81, 1.66, *m*	25.0, CH_2	1.73, 1.62 *m*
4	9.7, CH_3	0.89 *m*	9.9, CH_3	0.89 *m*

For shifted signals of DAB, HMBC correlations to 2-Thr and 4-DAB corroborate attachment to 3-DAB.

Table S4. NMR data of fragment of **34** in D_2O.

Referenced to D_2O δ_H 4.79 and FA-Me δ_{CH3} 22.2 ppm based on colistin B (**1**).

Conjugate **34**

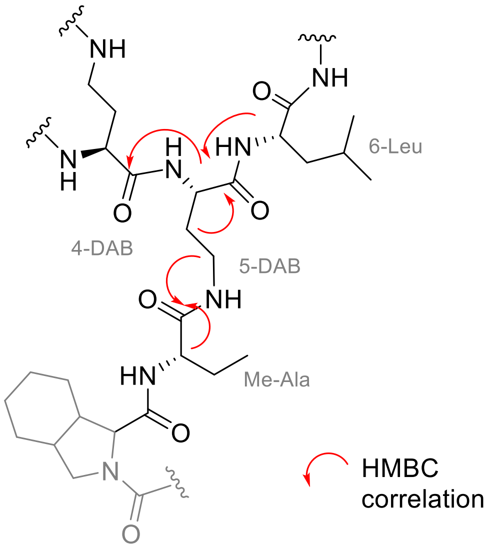

position	Colistin B (**1**) δ_C, mult.	δ_H (*J* in Hz)	Conjugate **34** (5-DAB bound) δ_C, mult.	δ_H (*J* in Hz)
4-DAB				
1	173.1, C	-	173.1, C	-
2	52.1, CH	4.31 *m*	52.1, CH	4.29 *m*
3	30.9, CH_2	2.00, 1.90 *m*	28.9, CH_2	2.23, 2.11 *m*
4	36.4, CH_2	3.36, 3.21 *m*	36.6, CH_2	3.13 *m*
5-DAB				
1	172.3, C	-	171.6, C	
2	50.9, CH	4.58 *dd* (8.4, 3.8)	51.2	4.58 *m*
3	30.2, CH_2	2.18, 2.06 *m*	30.3, CH_2	2.12, 2.07, *m*
4	36.4, CH_2	3.05 *m*	36.0, CH_2	3.42 *m*
6-Leu				
1	175.1, C	-	175.1, *C*	-
2	*53.2, CH*	4.30 *m*	52.5, *CH*	4.29 *m*
3	*40.0, CH_2*	1.67, 1.58 *m*	39.7, *CH_2*	1.68, 1.60 *m*
4	*24.6**, CH*	1.61 *m*	24.7, *CH*	1.60 *m*
	D-Ubi-ElaSub (**50**)		Conjugate **34** (5-DAB bound)	
Me-Ala				
1	176.4, C	-	175.4, C	-
2	55.0, CH	4.18, *d* (6.8)	57.2, CH	3.96, *m*
3	24.4, CH_2	1.81, 1.66, *m*	24.3, CH_2	1.73, 1.63 *m*
4	9.7, CH_3	0.89 *m*	9.9, CH_3	0.91 *m*

For shifted signals of DAB, HMBC correlations to 4-DAB and 6-Leu corroborate attachment to 5-DAB.

Table S5. NMR data of fragment of **40** in D_2O.

Referenced to D_2O δ_H 4.79 and FA-Me δ_{CH3} 22.2 ppm based on colistin B (**1**).

Conjugate **40**

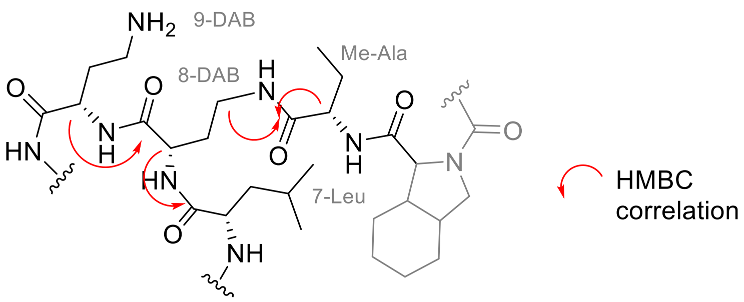

	Colistin B (**1**)		Conjugate **40** (8-DAB bound)	
position	δ_C, mult.	δ_H (*J* in Hz)	δ_C, mult.	δ_H (*J* in Hz)
7-Leu				
1	175.3, C	-	175.3, C	-
2	*52.1, CH*	4.42 *dd* (11.2, 4.3)	*52.1, CH*	4.50 *m*
3	*39.4, CH_2*	1.69 *m*	*40.1, CH_2*	1.72 *m*
4	*24.6**, CH*	1.61 *m*	*24.1, CH*	1.61 *m*
4a	*22.7,* CH_3	0.95 *d* (6.2)	*22.7,* CH_3	0.95 *m*
5	*20.3,* CH_3	0.88 *d* (6.2)	*20.3,* CH_3	0.88 *m*
8-DAB				
1	173.5, *C*	-	173.1, C	
2	52.1, CH	4.32 *m*	52.7, CH	4.30 *m*
3	28.1, CH_2	2.30, 2.20 *m*	28.6, CH_2	2.22, 2.10, *m*
4	36.8, CH_2	3.15 *m*	36.0, CH_2	3.41 *m*
9-DAB				
1	*172.9, C*	-	173.1, C	-
2	53.1, CH	4.29 *m*	53.4, CH	4.31 *m*
3	28.7, CH_2	2.27, 2.20 *m*	28.4, CH_2	2.21, 2.15 *m*
4	36.6, CH_2	3.11 *m*	36.8, CH_2	3.10 *m*
	D-Ubi-ElaSub (**50**)		Conjugate **40** (8-DAB bound)	
Me-Ala				
1	176.4, C	-	175.0, C	-
2	55.0, CH	4.18, *d* (6.8)	55.6, CH	4.14, *m*
3	24.4, CH_2	1.81, 1.66, *m*	24.8, CH_2	1.75, 1.64 *m*
4	9.7, CH_3	0.89 *m*	9.9, CH_3	0.90 *m*

For shifted signals of DAB, HMBC correlations to 7-Leu and 9-DAB corroborate attachment to 8-DAB.

Table S6. NMR data of fragment of **46** in D_2O**.**

Referenced to D_2O δ_H 4.79 and FA-Me δ_{CH3} 22.2 ppm based on colistin B (**1**).

Conjugate **46**

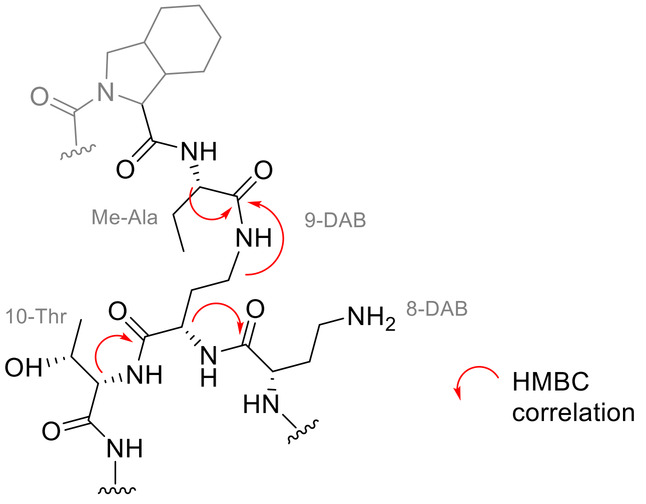

	Colistin B (**1**)		Conjugate **46** (9-DAB bound)	
position	δ_C, mult.	δ_H (*J* in Hz)	δ_C, mult.	δ_H (*J* in Hz)
8-DAB				
1	173.5, C	-	173.5, C	-
2	52.1, CH	4.32 *m*	51.3, CH	4.31 *m*
3	28.1, CH_2	2.30, 2.20 *m*	28.4, CH_2	2.29, 2.21 *m*
4	36.8, CH_2	3.15 *m*	36.8, CH_2	3.14 *m*
9-DAB				
1	*172.9, C*	-	172.7, C	-
2	53.1, CH	4.29 *m*	52.0, CH	4.36 *m*
3	28.7*, CH_2	2.27, 2.20 *m*	30.9, CH_2	2.05, 1.95 *m*
4	36.6*, CH_2	3.11 *m*	36.1, CH_2	3.37, 3.40 *m*
10-Thr				
1	171.7, C	-	171.5, C	-
2	59.7, CH	4.22 *d* (4.7)	59.4, CH	4.33, *m*
3	66.7, CH	4.26 *m*	67.5, CH	4.26 *m*
4	19.4, CH_3	1.22 *d* (6.5)	18.8, CH_3	1.20 *m*
	D-Ubi-ElaSub (**50**)		Conjugate **46** (9-DAB bound)	
Me-Ala				
1	176.4, C	-	174.8, C	-
2	55.0, CH	4.18, *d* (6.8)	56.0, CH	4.15, *d* (6.4)
3	24.4, CH_2	1.81, 1.66, *m*	25.0, CH_2	1.79, 1.73 *m*
4	9.7, CH_3	0.89 *m*	9.9, CH_3	0.89 *m*

For shifted signals of DAB, HMBC correlations to 8-DAB and 10-Thr corroborate attachment to 8-DAB.

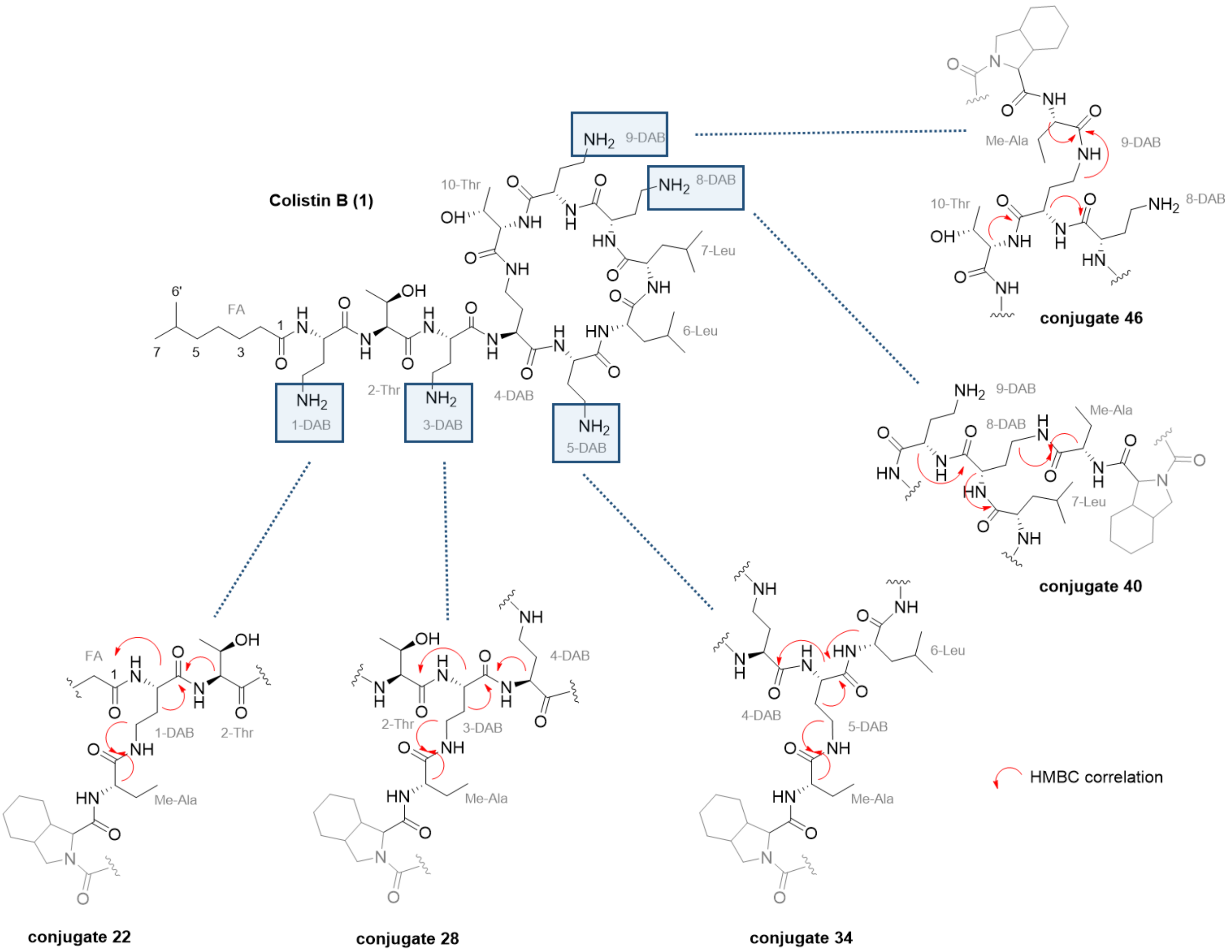

Figure S17. Sections of conjugate structures 22, 28, 34, 40 and 46. Relevant NMR HMBC correlations for proof of attachment site are illustrated as red arrows. For NMR data see Tables S1 – S6.

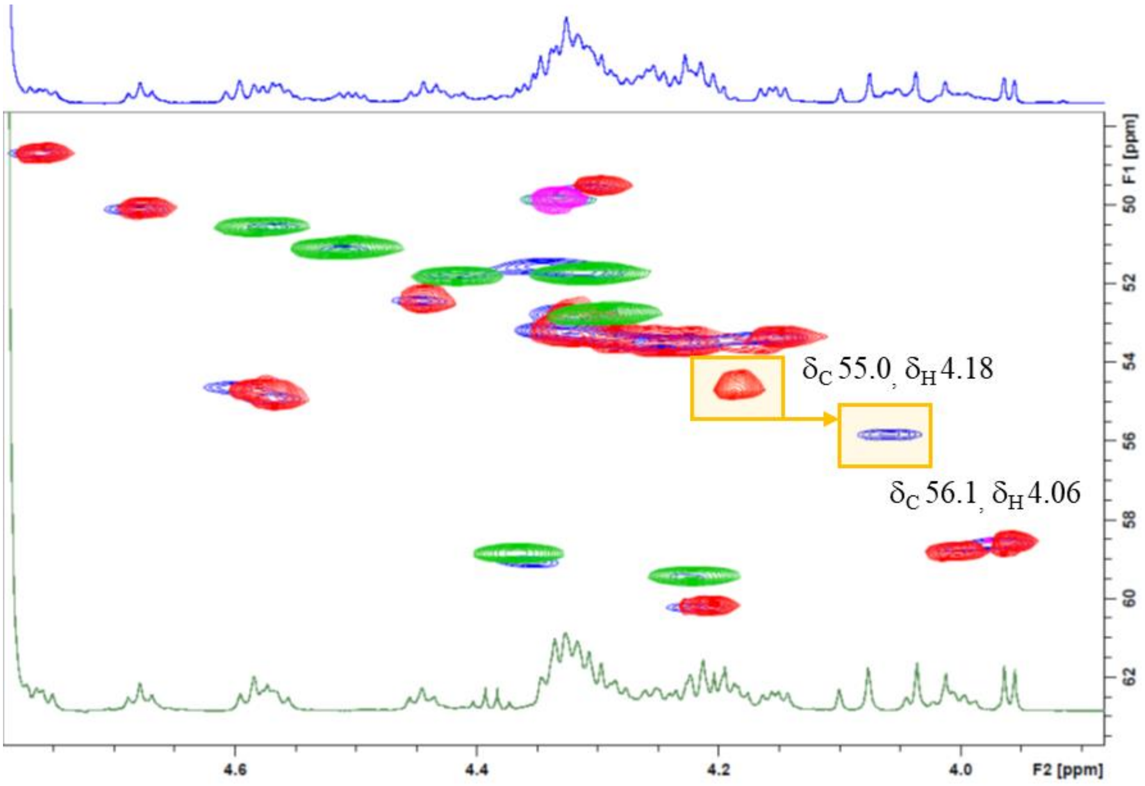

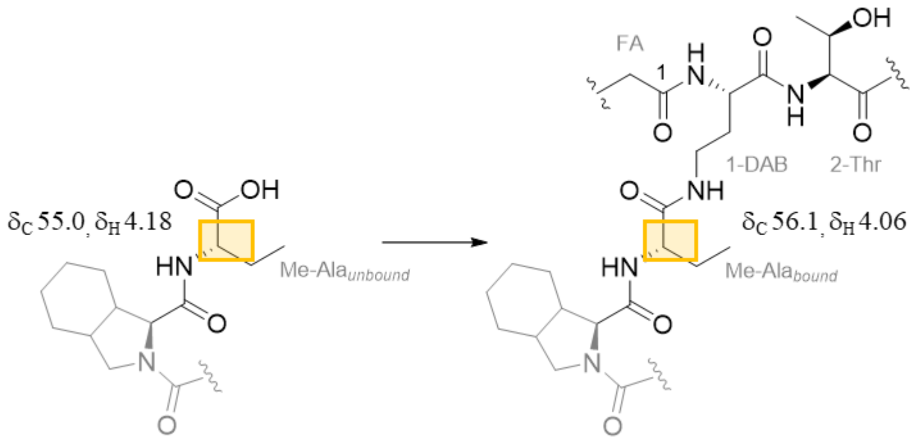

Figure S18. Overlay of HSQC spectra. HSQC spectra of D-Ubi-Ela-Sub (**50**, signals in red/pink), colistin (**1**, signals in green) and D-Ubi-Ela-Sub-1-colistin (**22**, signals in blue/mintgreen). Signal shift of Me-Ala α-CH in linker is shown framed in orange.

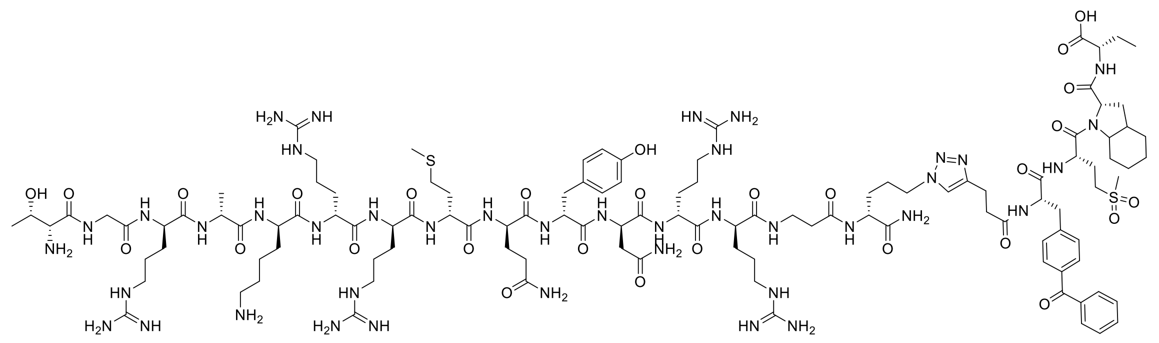

Figure S19. Structure of D-Ubi-Ela-Sub (50). D-ubiquicidin$_{29\text{-}41}$-ßAla-lys(N_3) coupled to elastase substrate peptide via N-terminal 4-pentinoyl group.

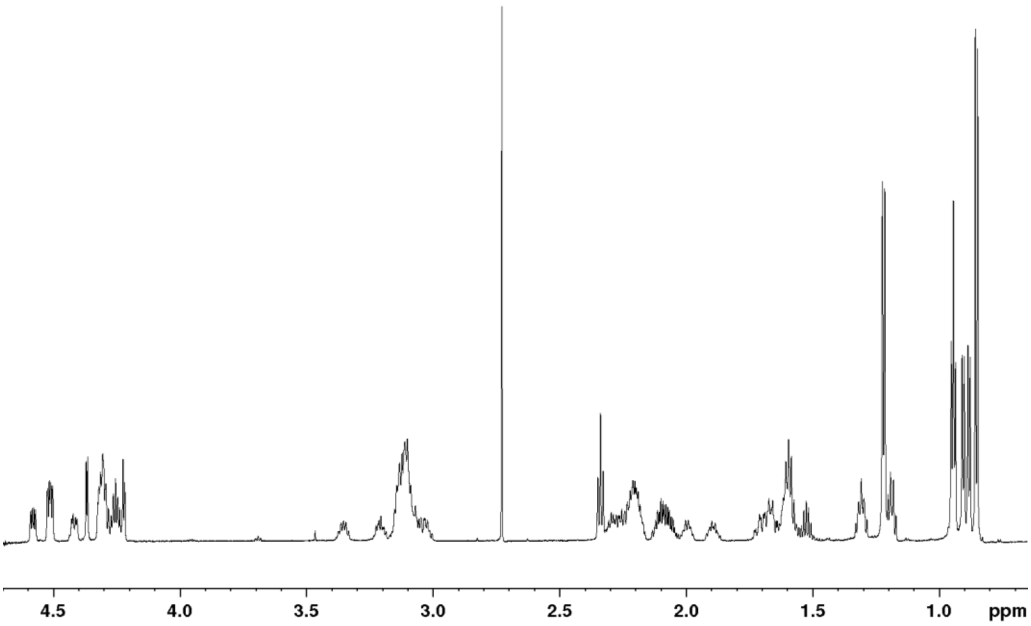

Figure S20. ^{1}H-NMR spectrum of Colistin B (Polymyxin E2) (**1**) in D_2O at 700 MHz.

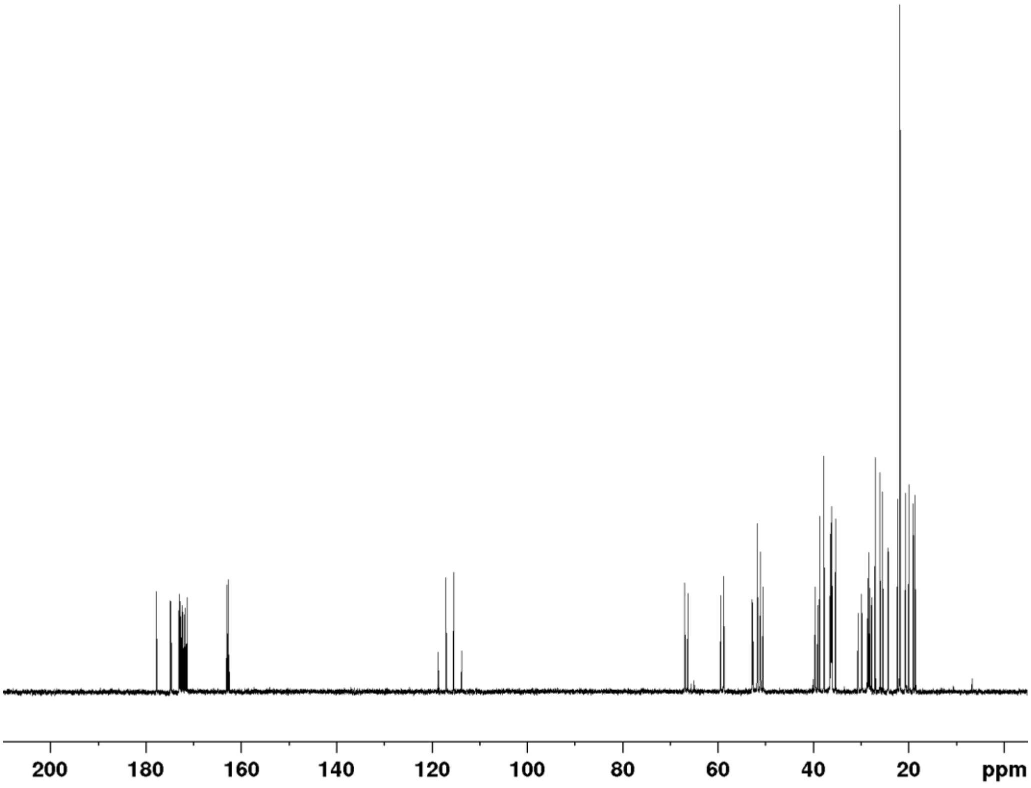

Figure S21. ^{13}C-NMR spectrum of Colistin B (Polymyxin E2) (**1**) in D_2O at 175 MHz.

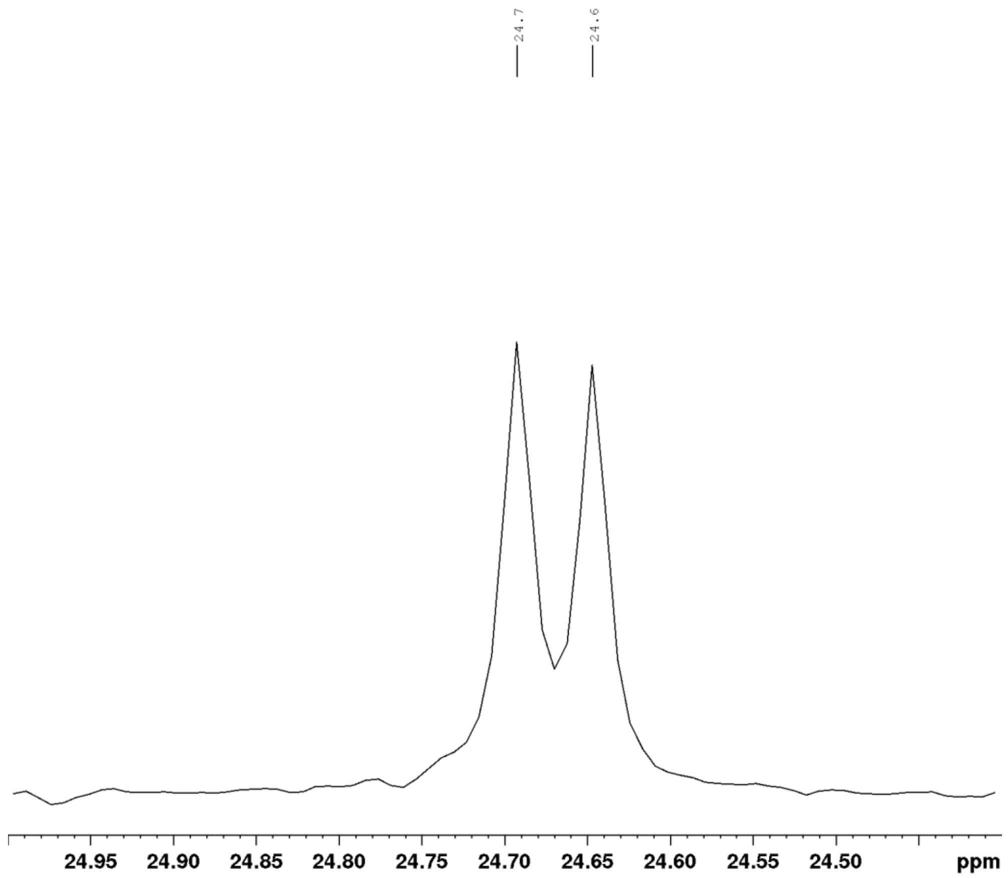

Figure S22. Section of ^{13}C-NMR spectrum of Colistin B (Polymyxin E2) (**1**) in D_2O at 175 MHz. Very close chemical shift values for $CH(CH_3)_2$-group of 6-Leu and 7-Leu.

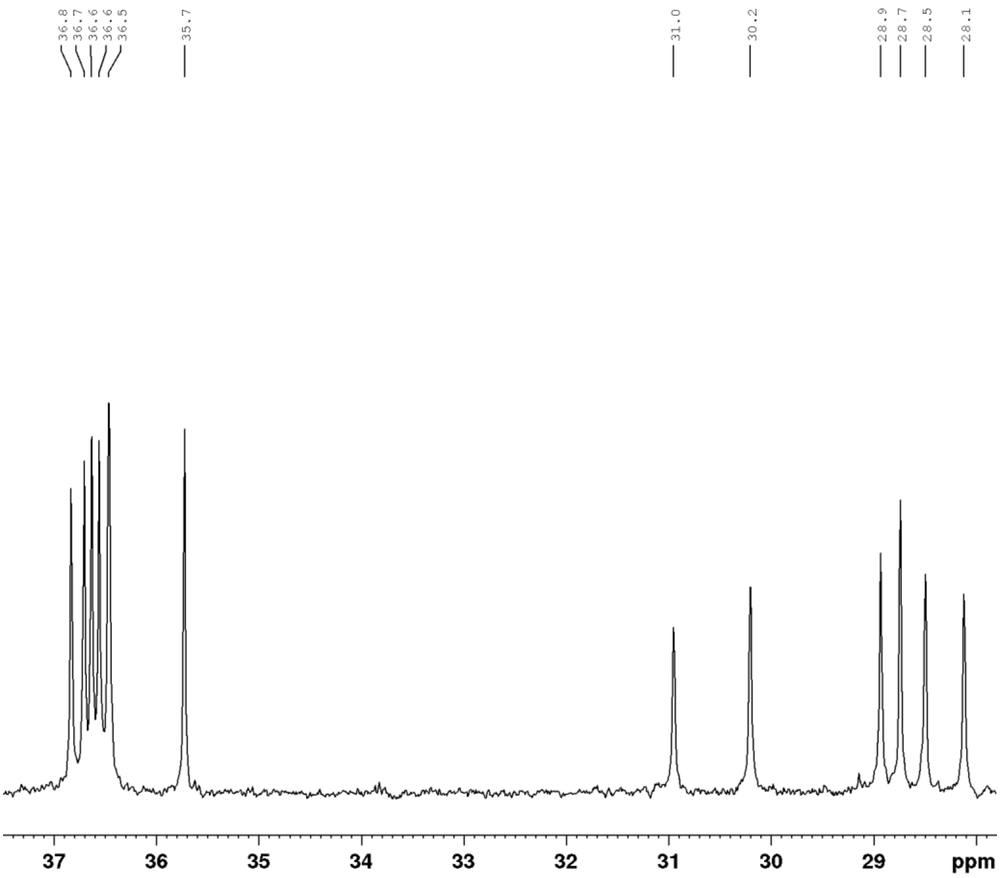

Figure S23. ^{13}C-NMR spectrum of Colistin B (Polymyxin E2) (**1**) in D_2O at 175 MHz. Very close ^{13}C-shift values for DAB CH2 groups in 1-DAB, 3-DAB, 9-DAB.

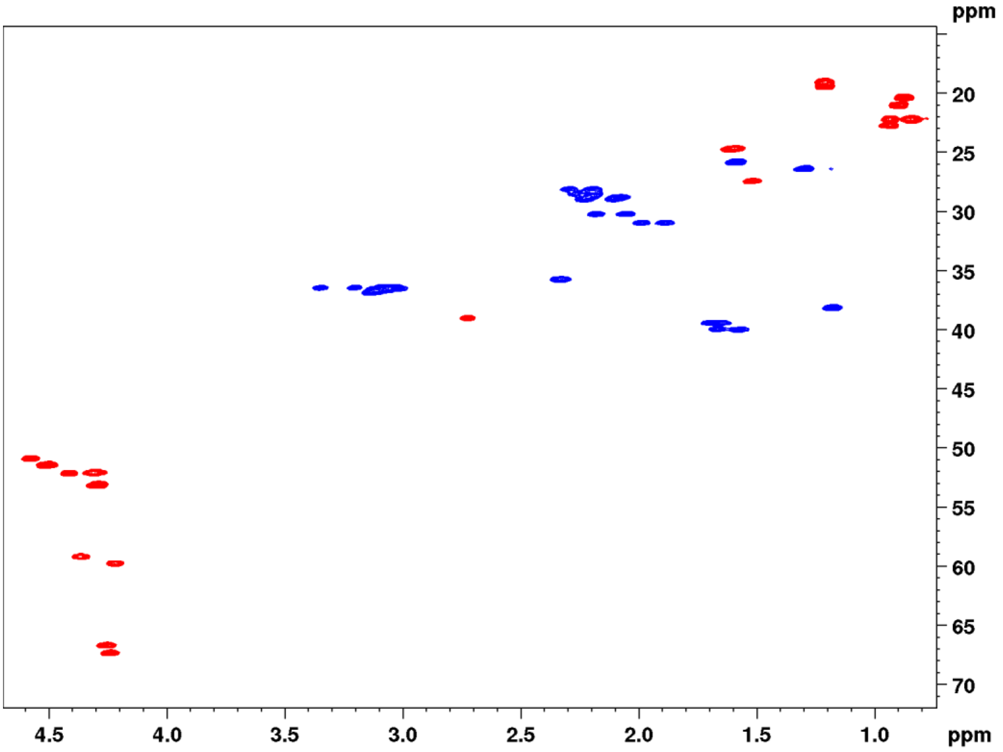

Figure S24. HSQC spectrum of Colistin B (Polymyxin E2) (**1**) in D_2O.

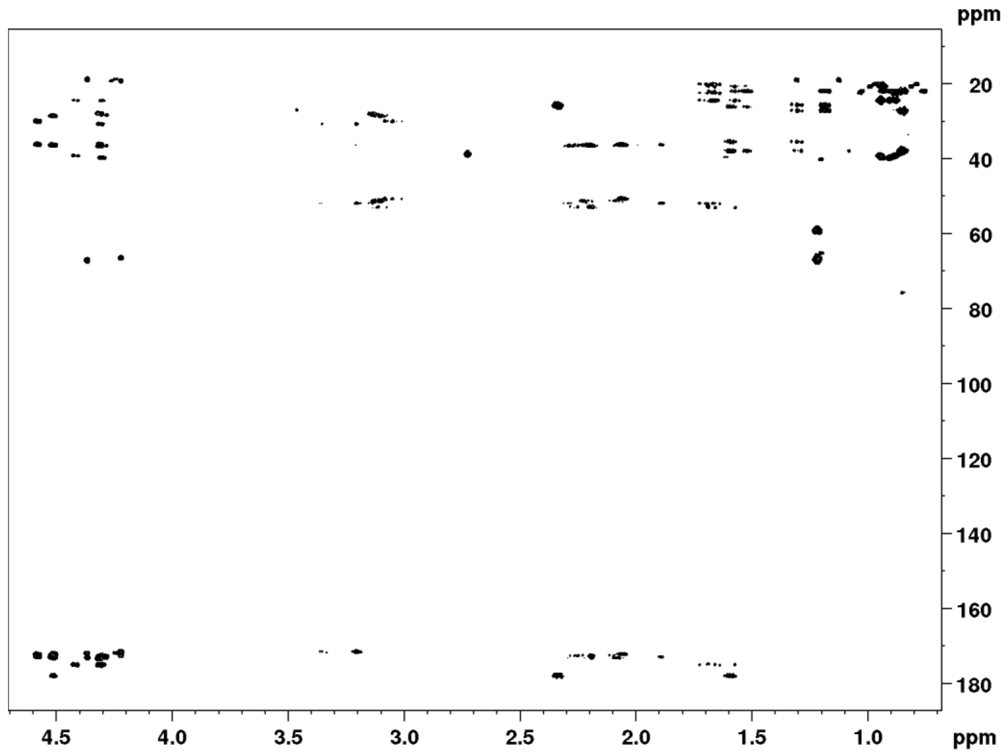

Figure S25. HMBC spectrum of Colistin B (Polymyxin E2) (**1**) in D_2O.

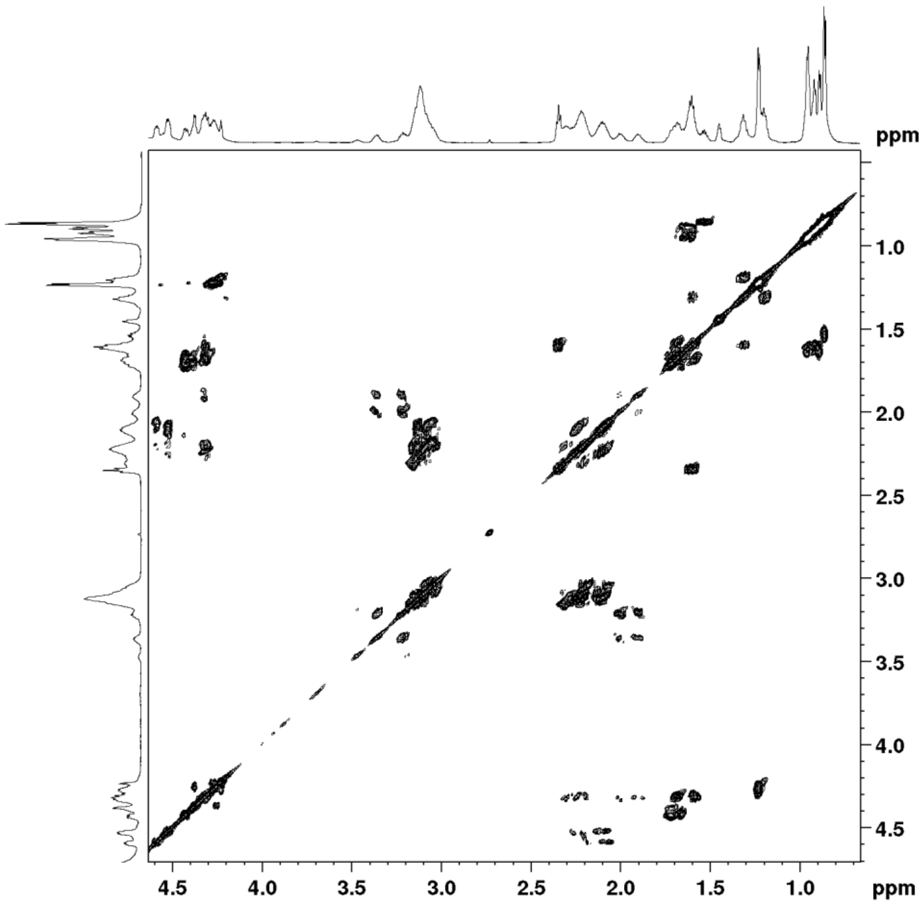

Figure S26. COSY spectrum of Colistin B (Polymyxin E2) (**1**) in D_2O.

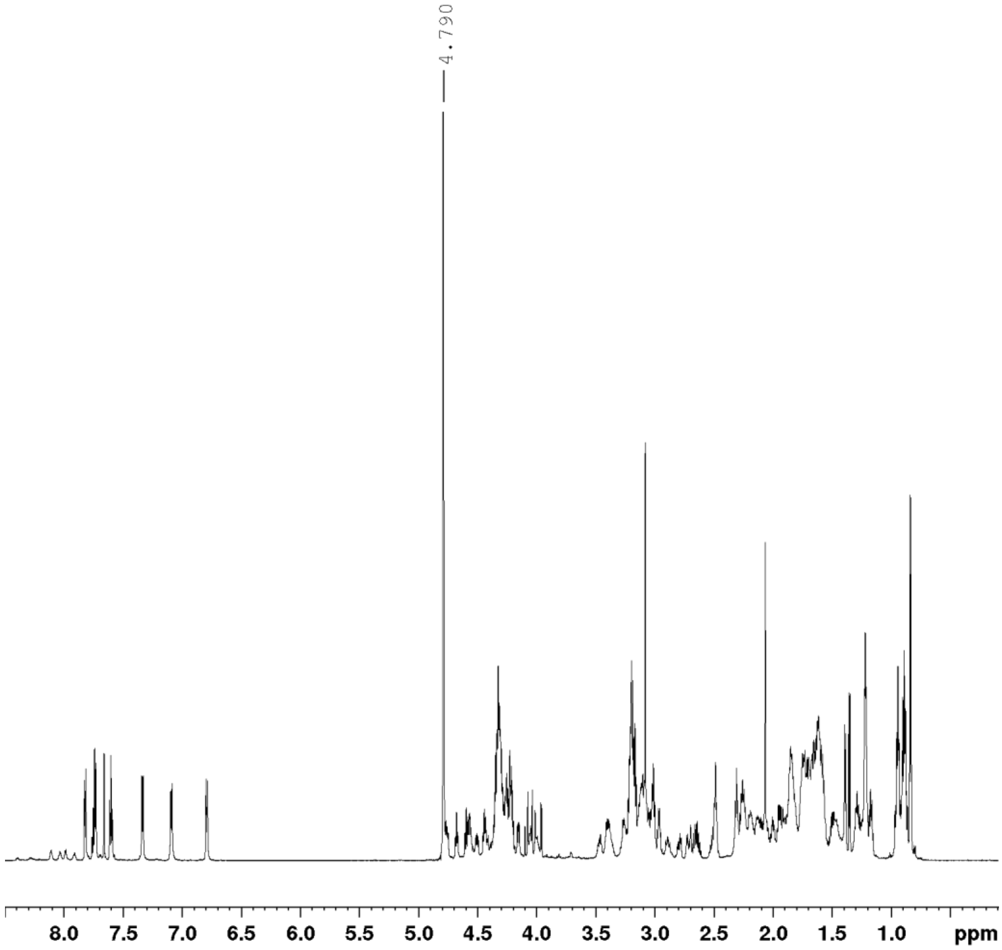

Figure S27. ^{1}H-NMR spectrum of conjugate **22** in D_2O at 700 MHz.

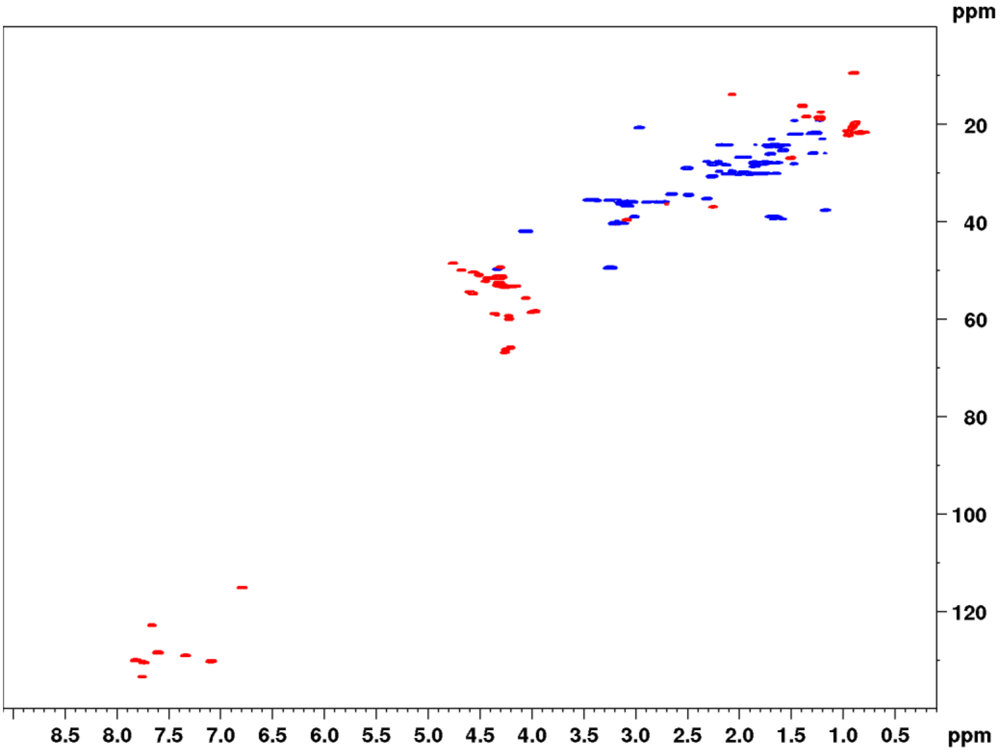

Figure S28. HSQC spectrum of conjugate **22** in D_2O.

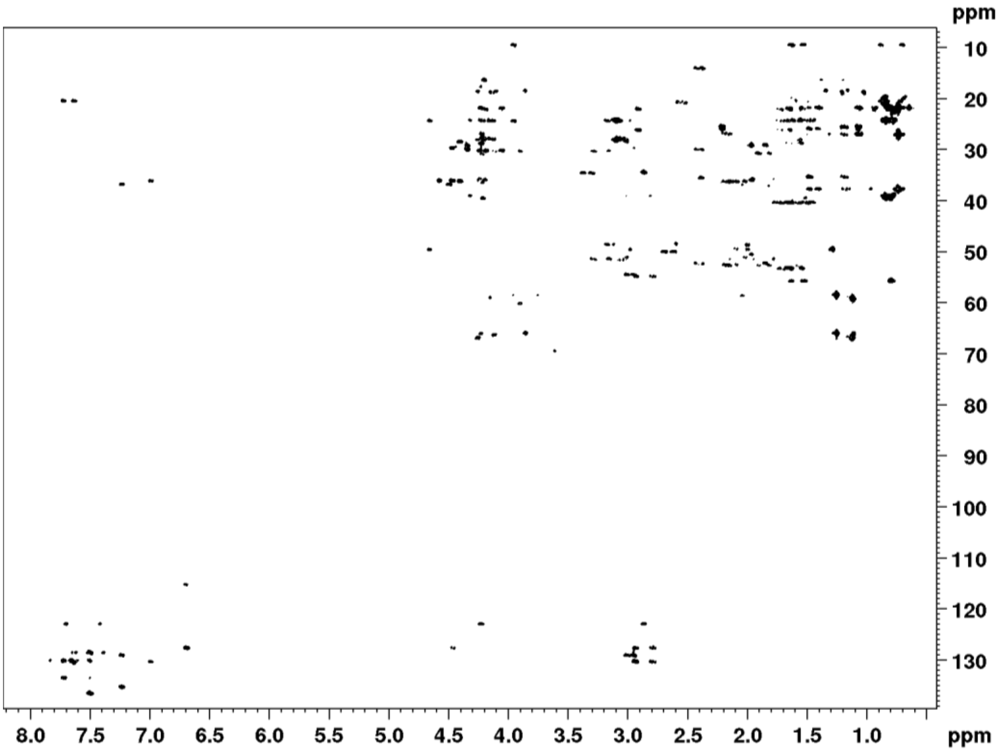

Figure S29. HMBC spectrum of conjugate **22** in D_2O.

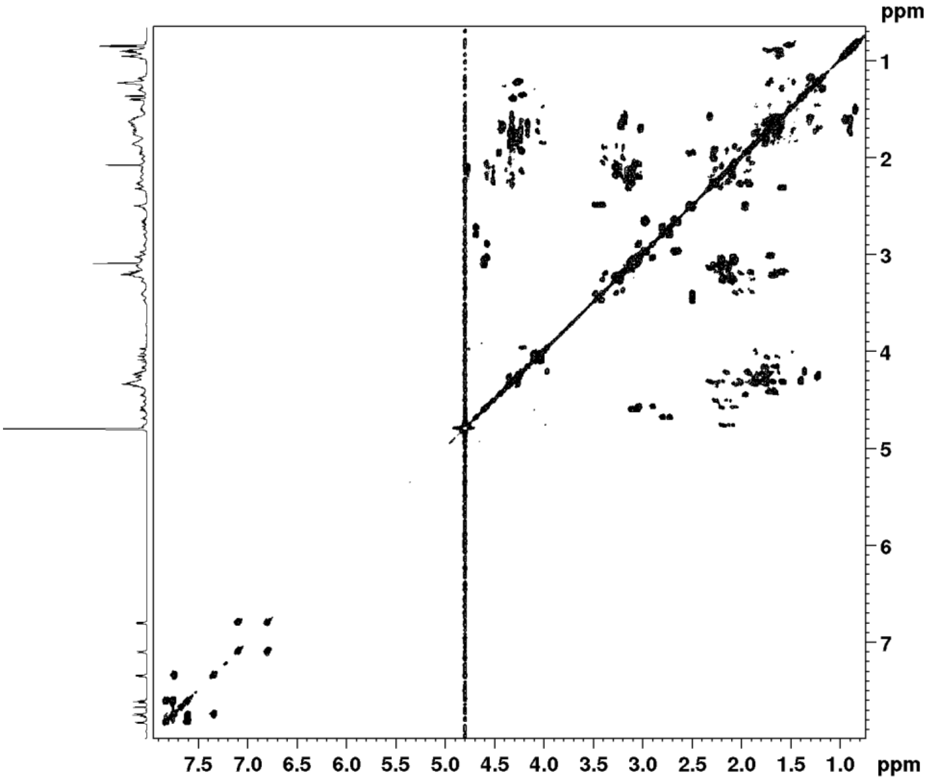

Figure S30. COSY spectrum of conjugate **22** in D_2O.

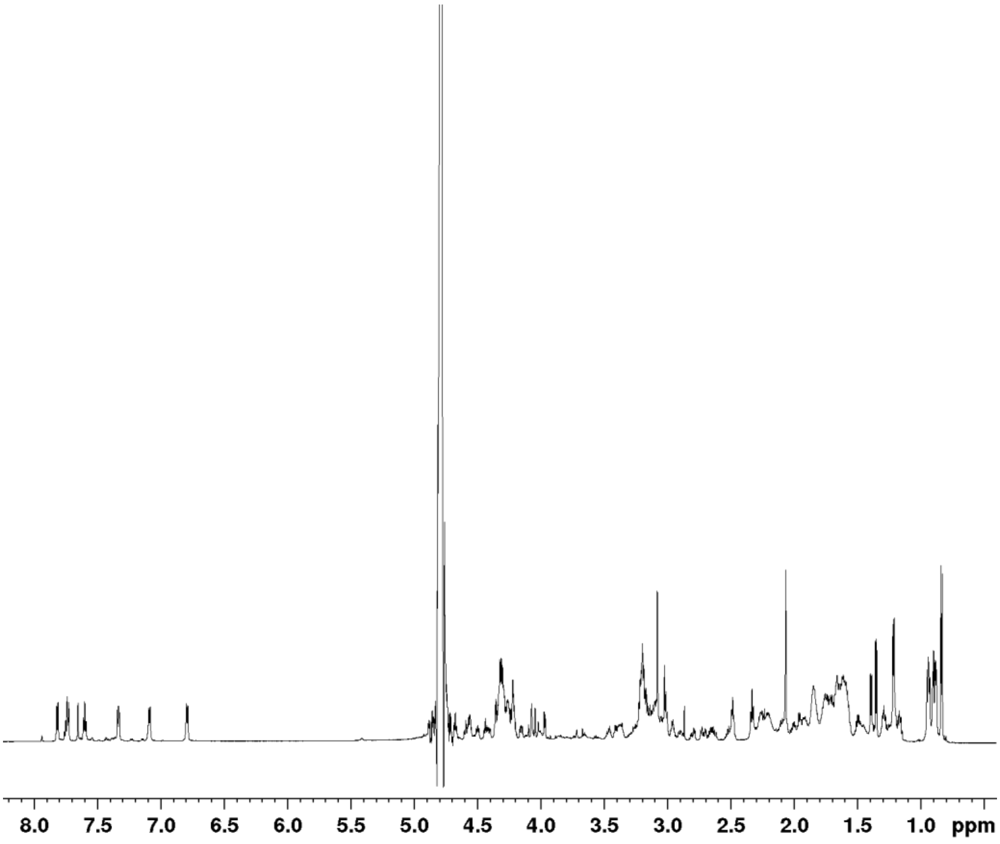

Figure S31. ^{1}H-NMR spectrum of conjugate **28** in D_2O at 700 MHz.

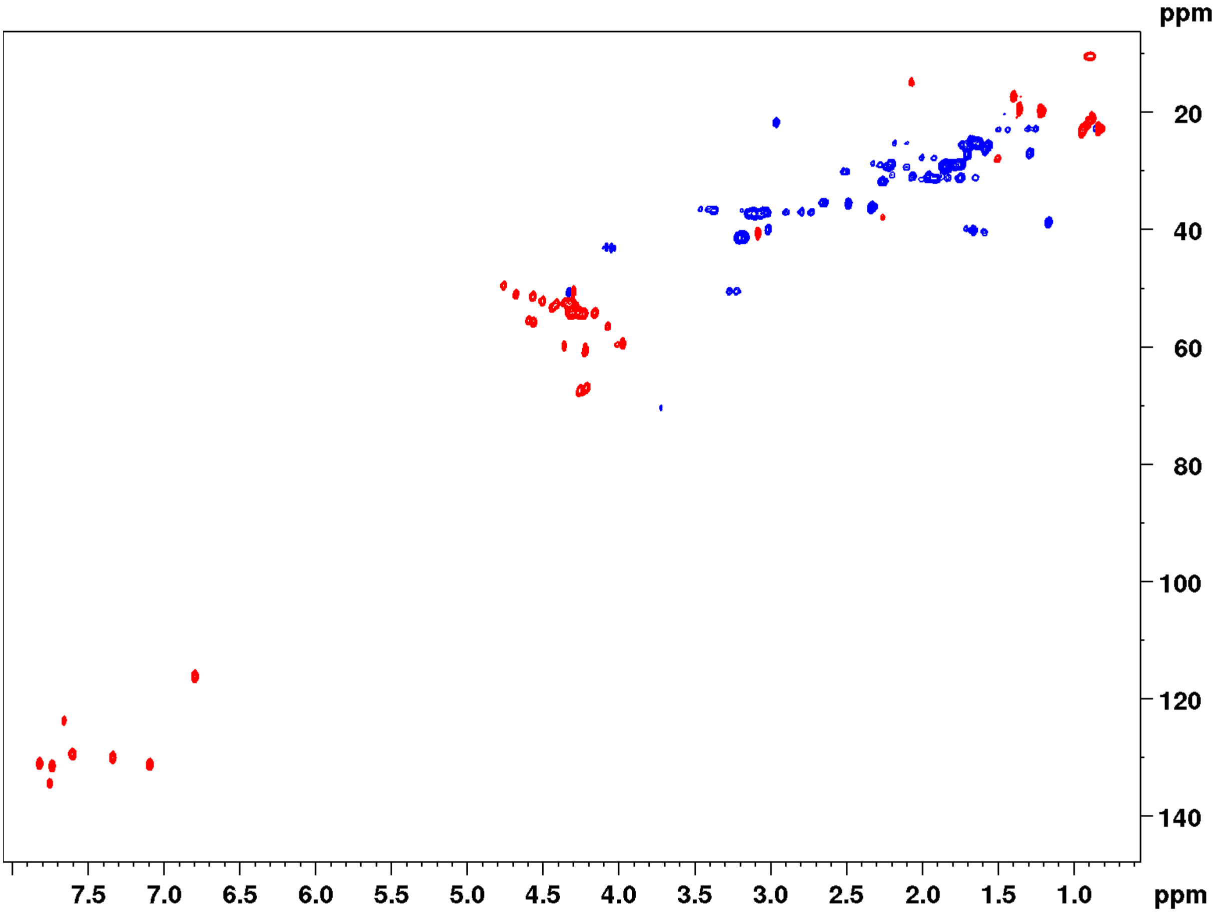

Figure S32. HSQC spectrum of conjugate **28** in D_2O.

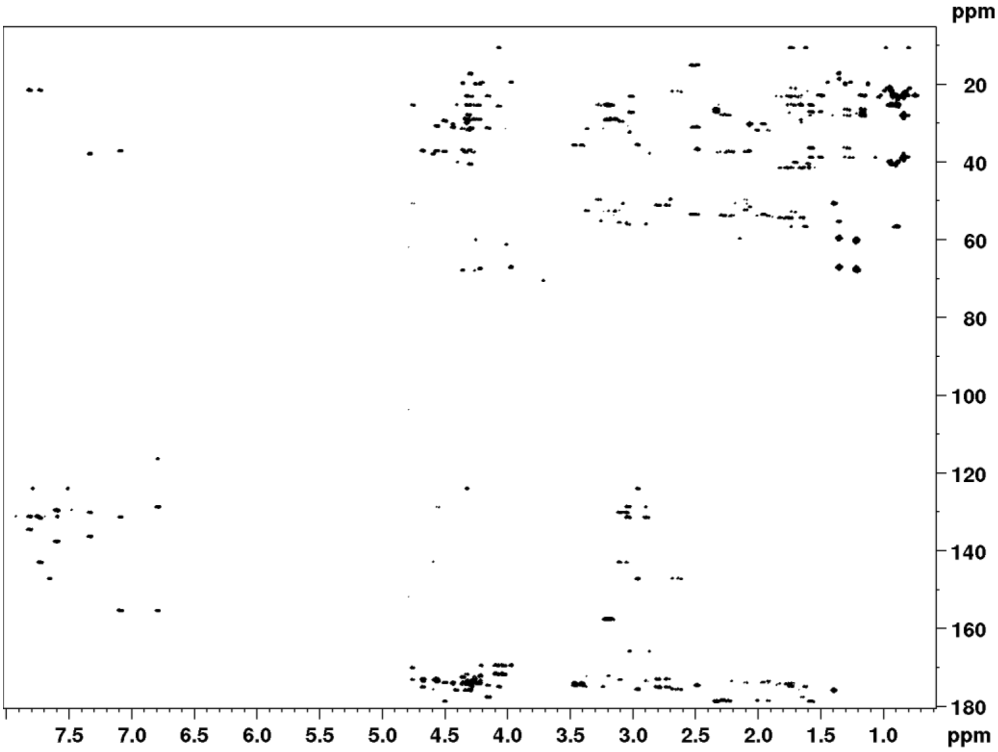

Figure S33. HMBC spectrum of conjugate **28** in D_2O.

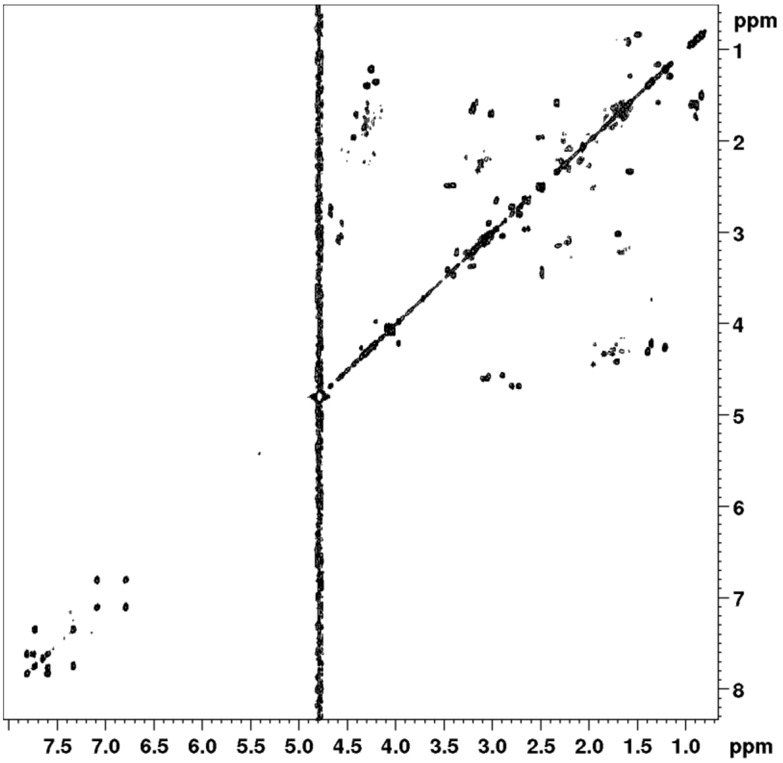

Figure S34. COSY spectrum of conjugate **28** in D_2O.

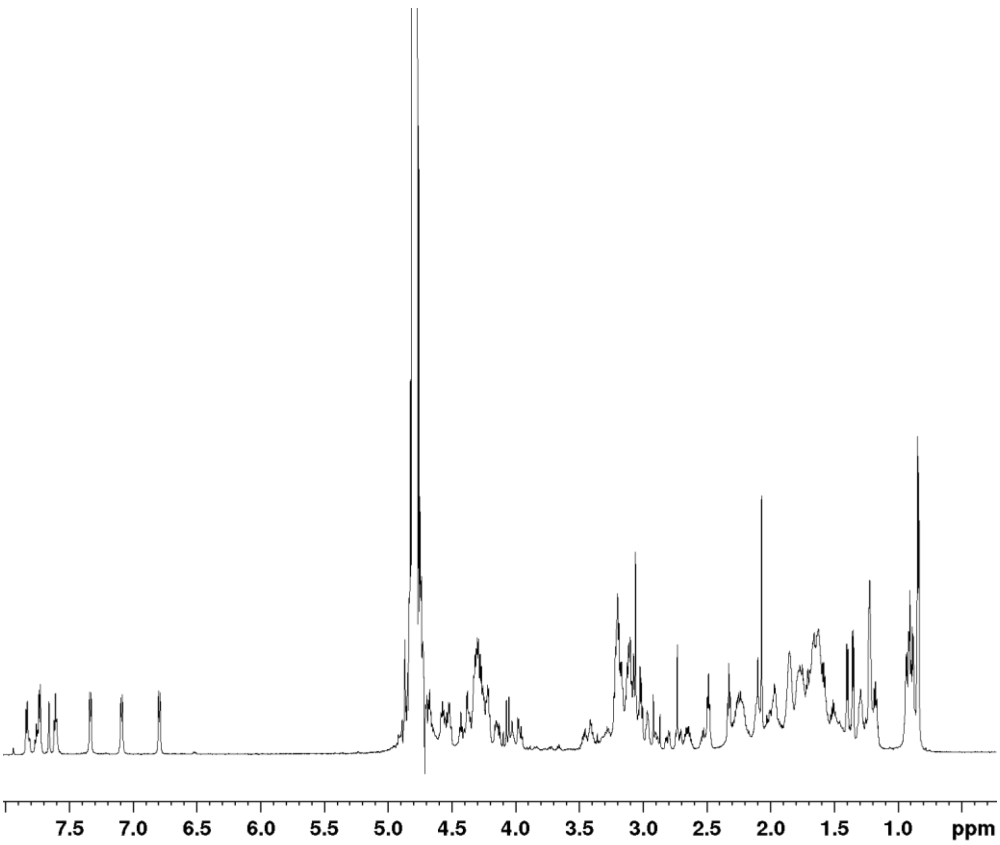

Figure S35. ^{1}H-NMR spectrum of conjugate **34** in D_2O at 700 MHz.

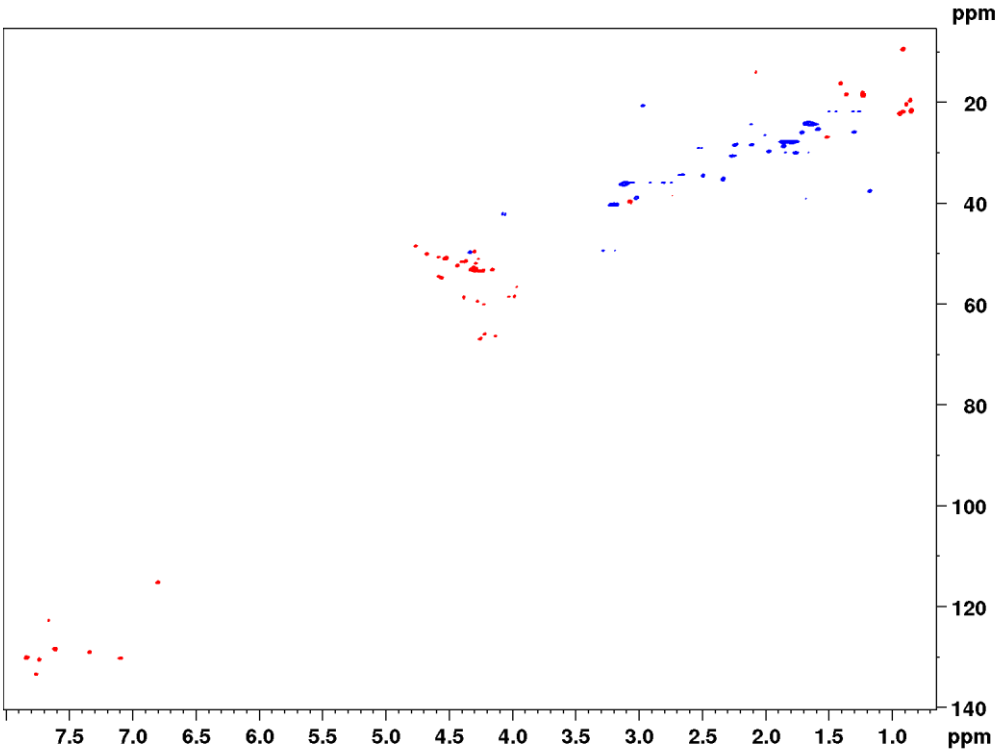

Figure S36. HSQC spectrum of conjugate **34** in D_2O.

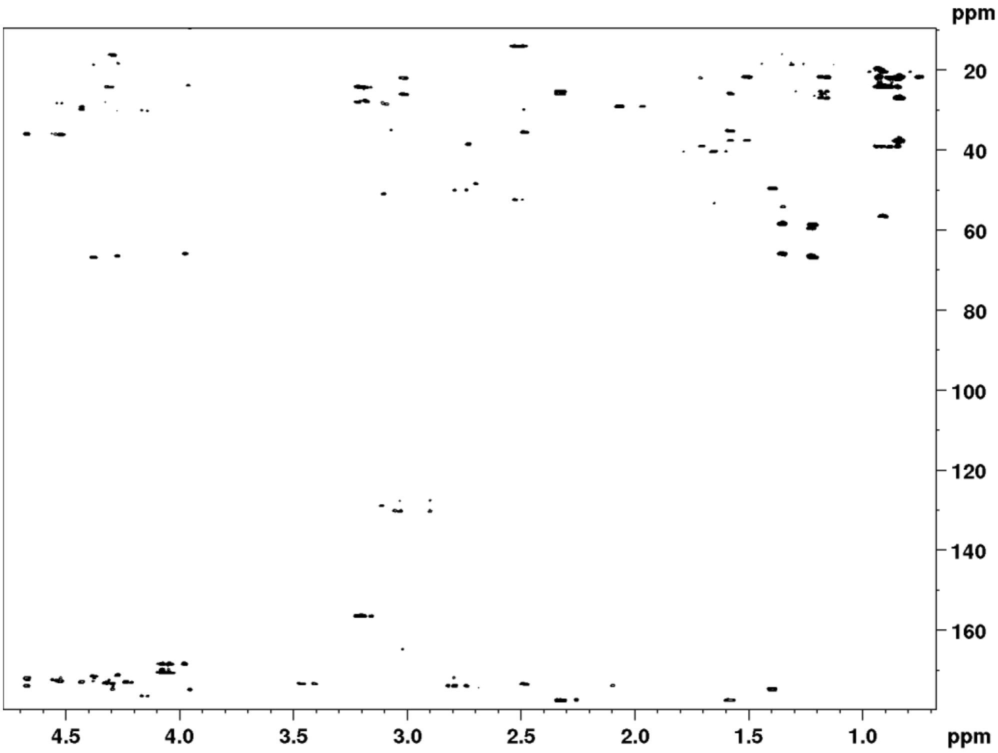

Figure S37. HMBC spectrum of conjugate **34** in D_2O.

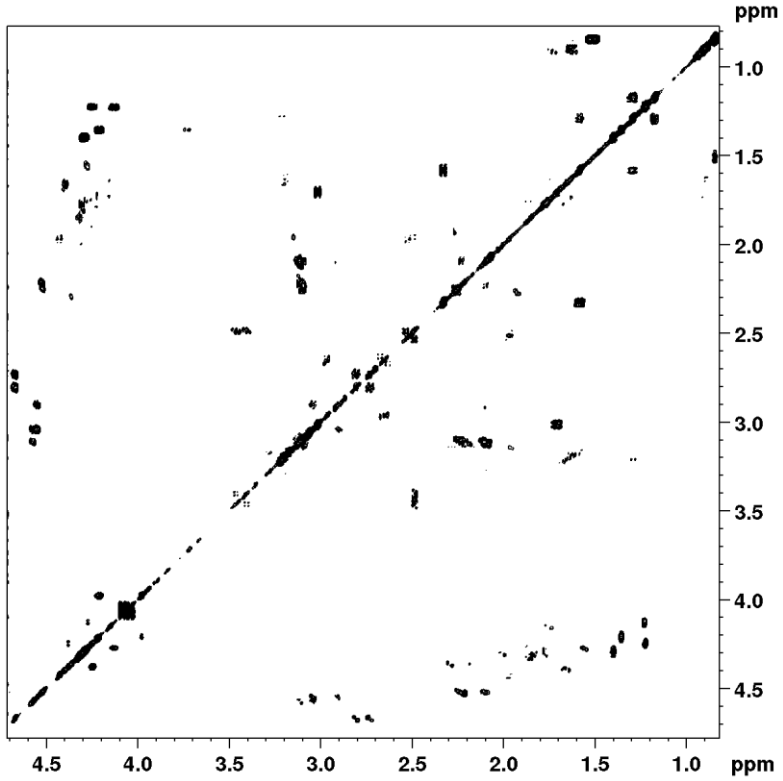

Figure S38. COSY spectrum of conjugate **34** in D_2O.

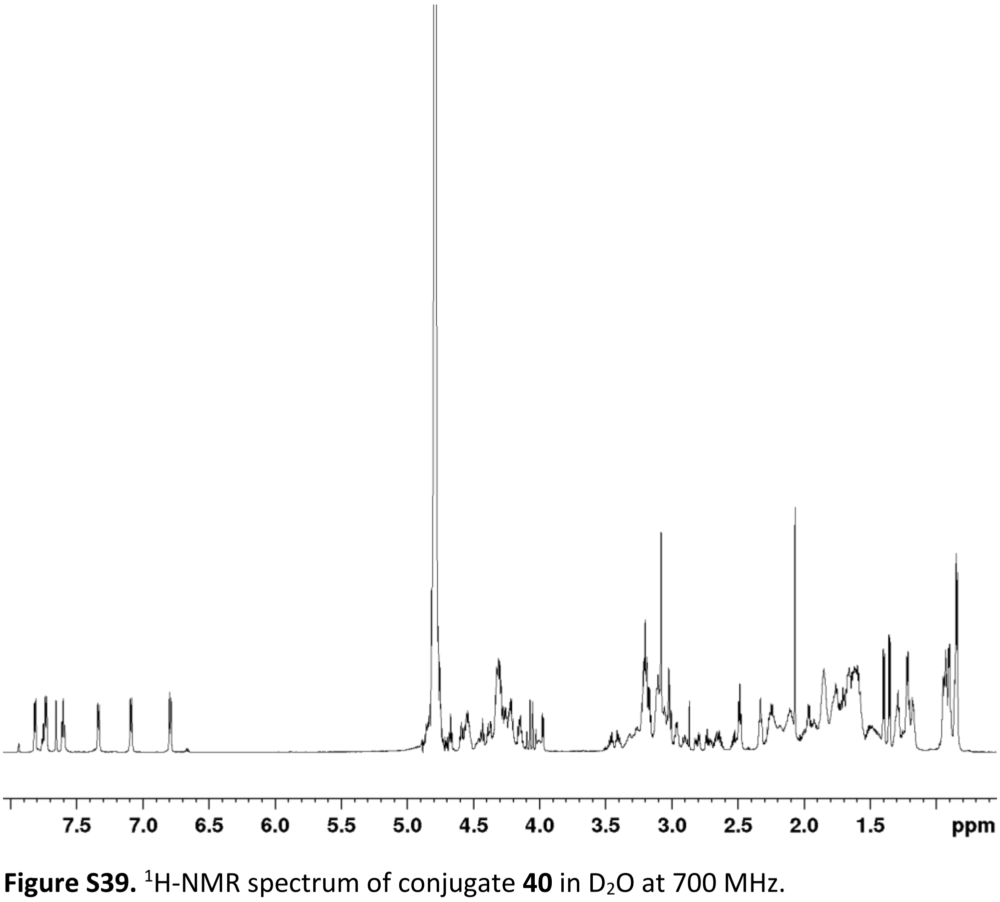

Figure S39. ^{1}H-NMR spectrum of conjugate **40** in D_2O at 700 MHz.

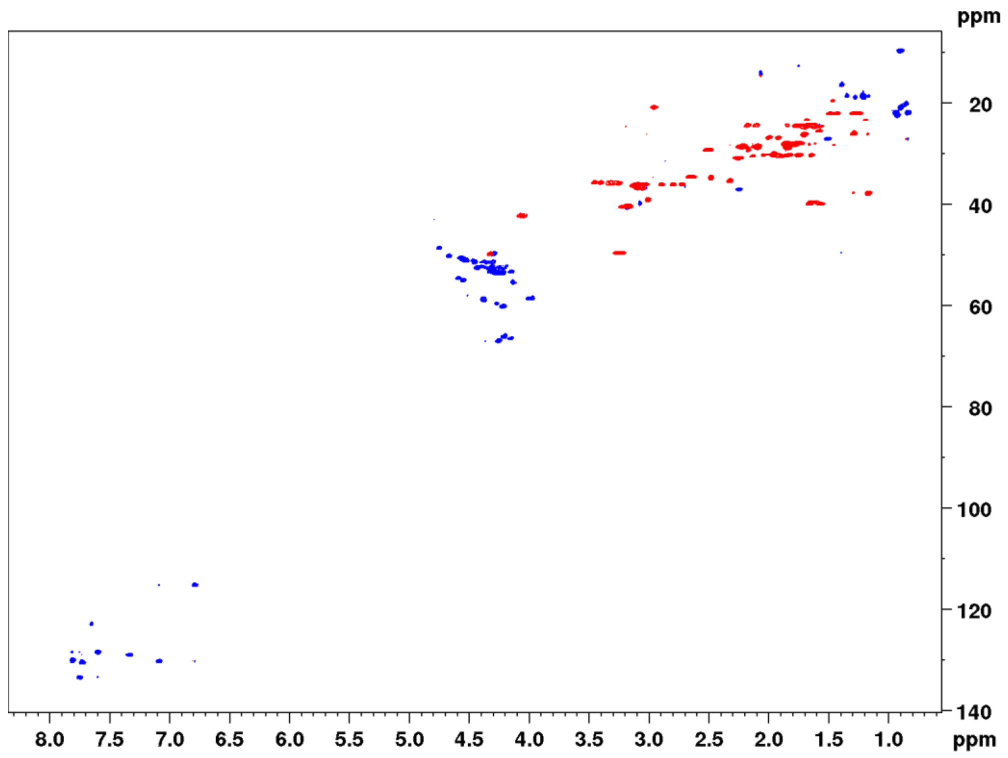

Figure S40. HSQC spectrum of conjugate **40** in D_2O.

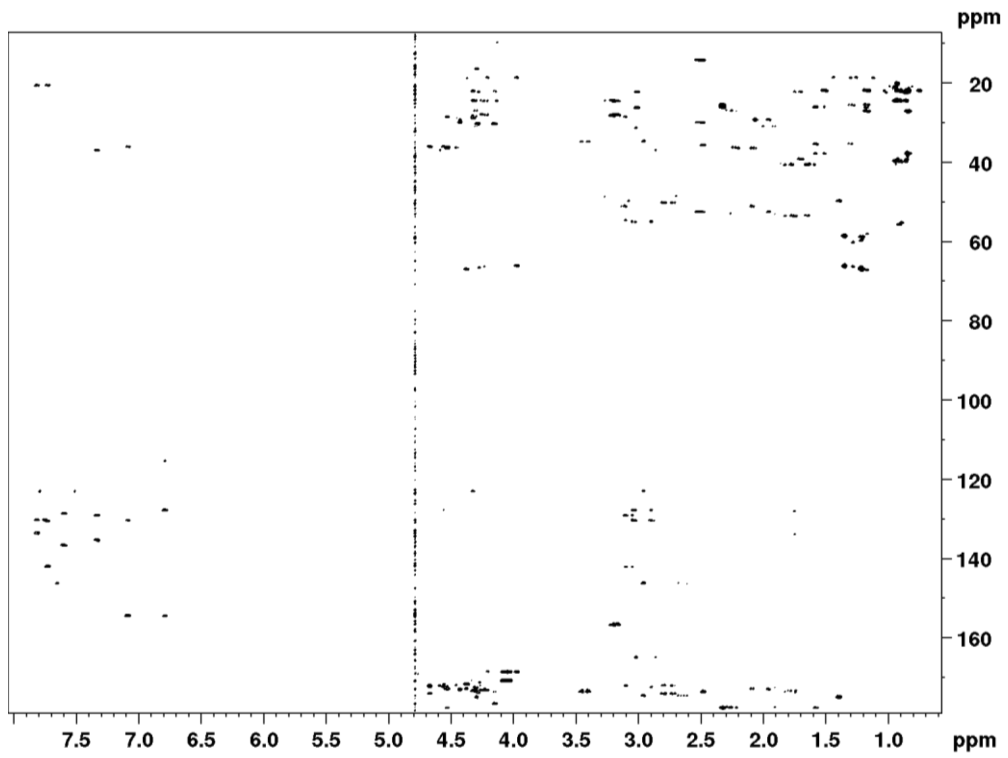

Figure S41. HMBC spectrum of conjugate **40** in D_2O.

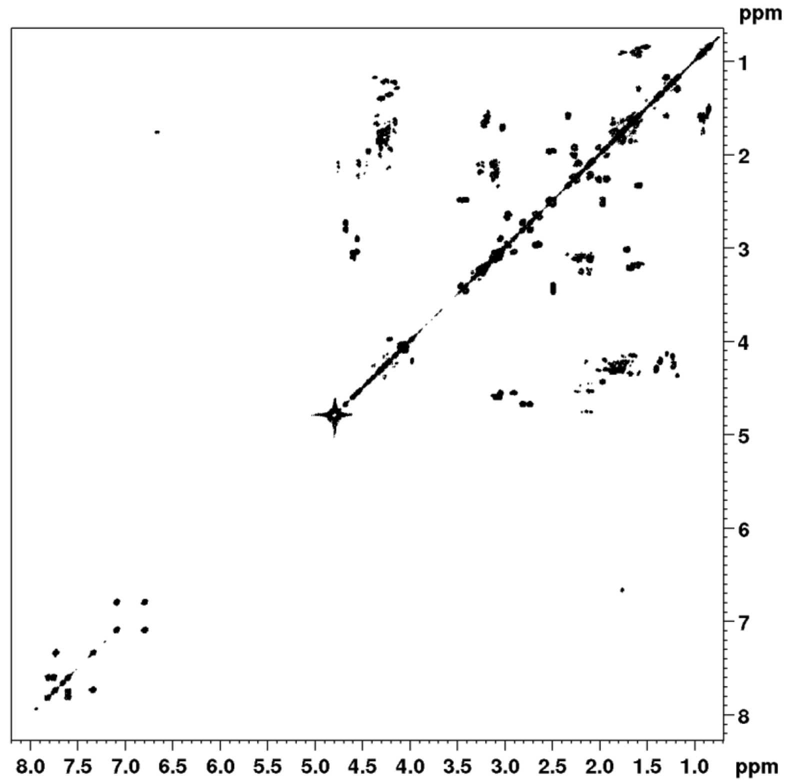

Figure S42. COSY spectrum of conjugate **40** in D_2O.

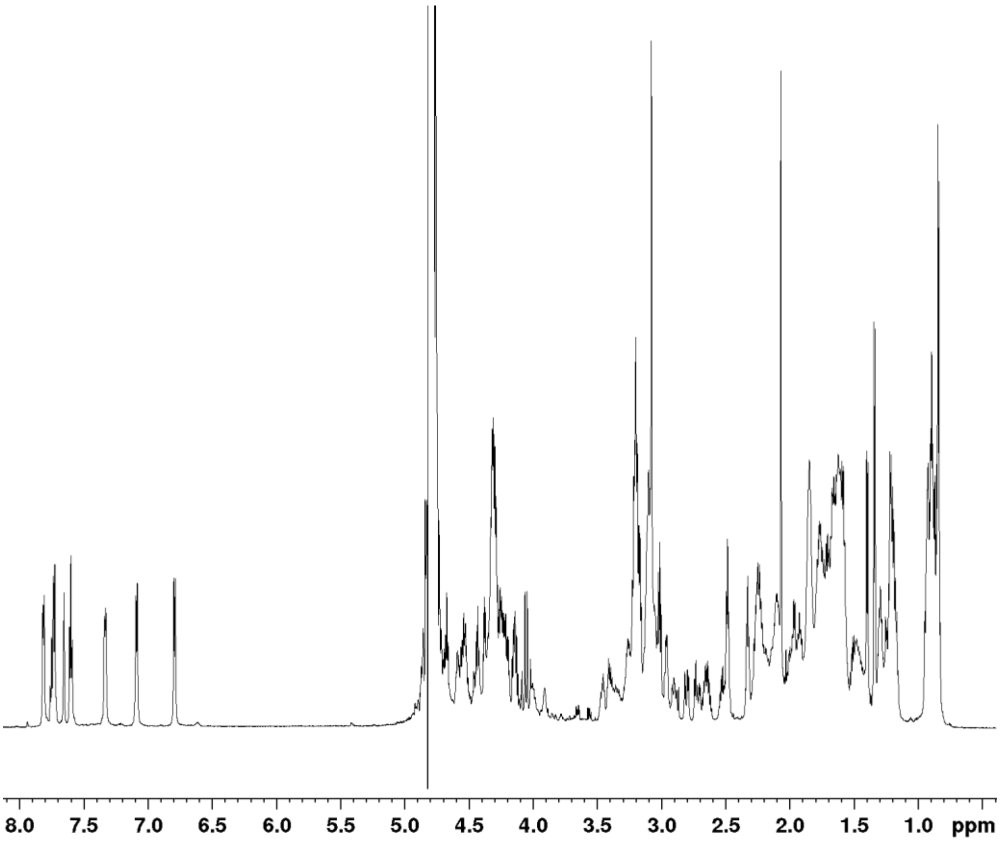

Figure S43. ^1H-NMR spectrum of conjugate **46** in D_2O at 700 MHz.

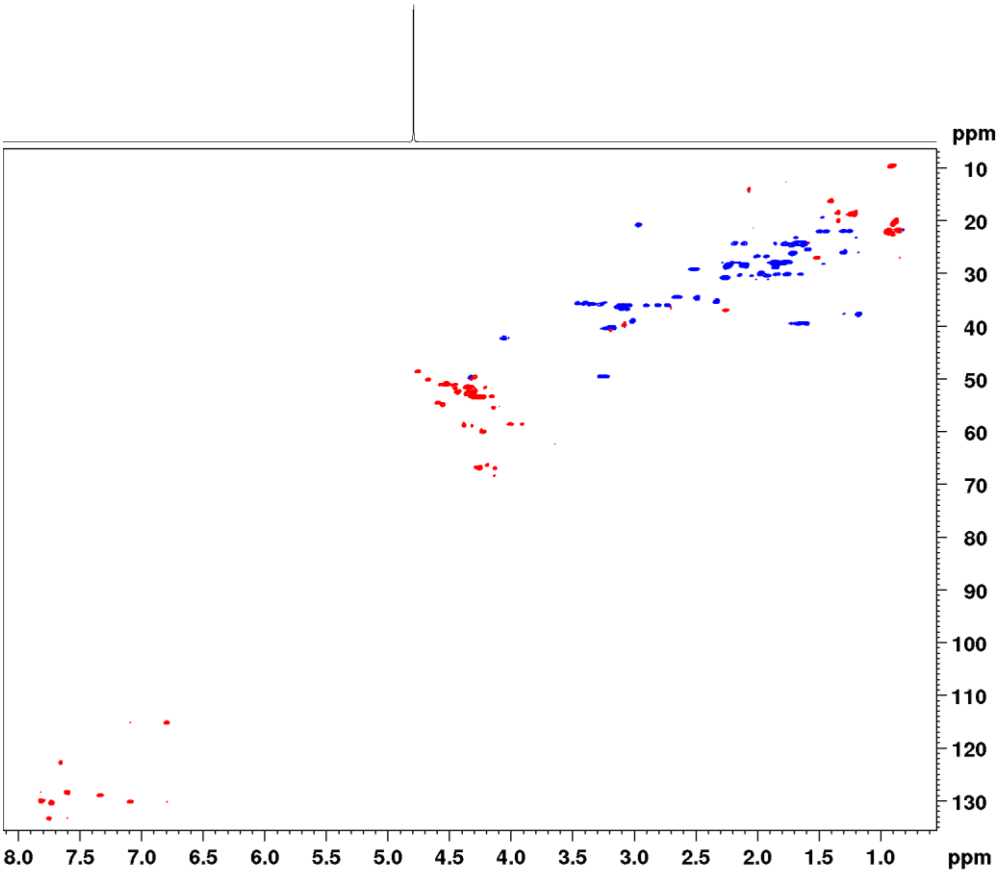

Figure S44. HSQC spectrum of conjugate **46** in D_2O.

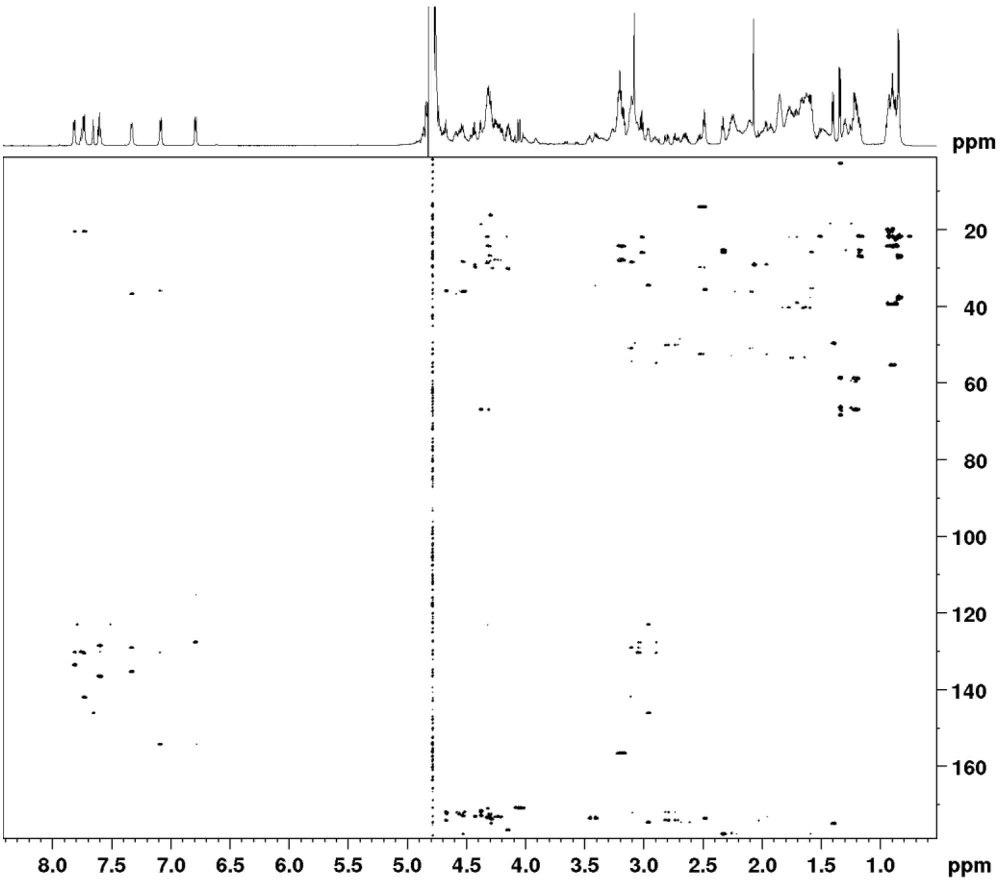

Figure S45. HMBC spectrum of conjugate **46** in D_2O.

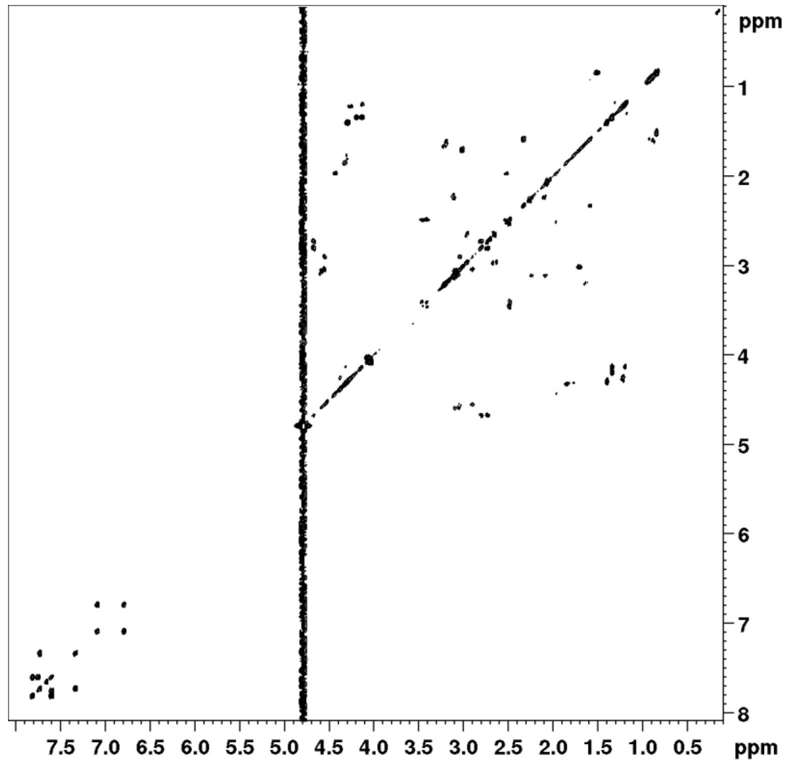

Figure S46. COSY spectrum of conjugate **46** in D_2O.
